# Supplementary material for: Fingolimod normalizes metabolic signatures associated with synaptic plasticity and memory in APP/PS1 model: Sphingosine-1-phosphate receptor a therapeutic target for Alzheimer’s
Source: Sci Rep. 2026 Mar 10;16:12835. doi: 10.1038/s41598-026-42518-8 (PMC13096423; doi:10.1038/s41598-026-42518-8)
Supplement: Supplementary file 3 — Supplementary Information 3. [file 41598_2026_42518_MOESM3_ESM.pdf]

biocrates
The future of research and health

MxP® Quant 500 XL kit

List of isobars and isomers

biocrates.com

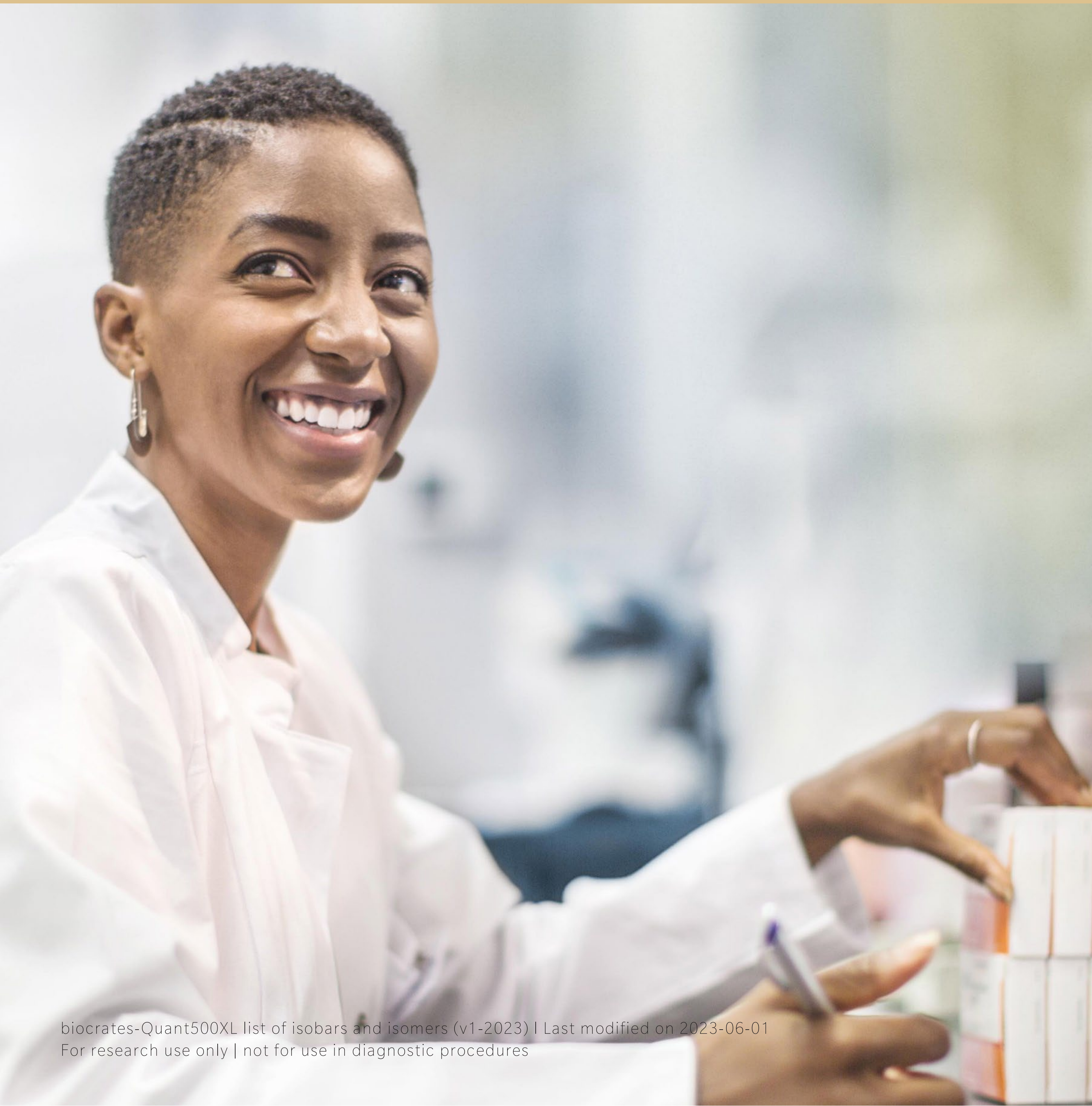

biocrates-Quant500XL list of isobars and isomers (v1-2023) I Last modified on 2023-06-01
For research use only | not for use in diagnostic procedures

Annotation of potential isobaric and isomeric lipid species analyzed using the MxP® Quant 500 XL kit

Introduction

The MxP® Quant 500 XL offers the largest combination of lipids and small molecules for targeted metabolic profiling in a single kit. Powered by biocrates' standardized, quality-controlled, and reproducible metabolomics technology, the ready-to-use kit covers more than 1,000 metabolites from various biochemical classes. The analysis of small molecules is ensured by liquid chromatography-tandem mass spectrometry (LC-MS/MS), while hexoses and lipids are analyzed by flow-injection analysis-tandem mass spectrometry (FIA-MS/MS).

The contributing isobars/isomers for each metabolite signal are listed in the following tables. While this list is comprehensive and established to the best of our knowledge, it is not exhaustive. The purpose is to provide a basis for better understanding and interpreting the results generated using the MxP® Quant 500 XL kit.

Overview of lipid isobars and isomers

Metabolite class	Number of metabolites	Page
Acylcarnitines	40	9
Lysophosphatidic acids	8	10
Phosphatidic acids	41	11
Lysophosphatidylcholines	12	13
Phosphatidylcholines	78	14
Lysophosphatidylethanolamines	43	41
Phosphatidylethanolamines	95	43
Lysophophatidylglycerols	10	63
Phosphatidylglycerols	64	63
Lysophosphatidylinositols	16	73
Phosphatidylinositols	53	74
Lysophosphatidylserines	12	86
Phosphatidylserines	18	87
Sphinganines and sphingosines	8	95
Sphinganine and sphingosine phosphates	8	95
Sphingomyelins	15	95
Ceramides	29	96
Dihydroceramides	8	97
Hexosylceramides	19	97
Dihexosylceramides	9	98
Trihexosylceramides	6	98
Cholesteryl esters	22	99
Monoglycerides	12	99
Diglycerides	44	100
Triglycerides	242	101

LC-MS/MS based metabolites

By using the MxP® Quant 500 XL kit, up to 106 small molecules from 13 biochemical classes can be quantified by LC-MS/MS analysis. The chromatographic separation provides an additional level of selectivity enabling differentiation of constitutional isomers and, to some extent, *cis-trans* stereoisomers. Enantiomeric isomers (such as D- and L-amino acids), however, are not able to be separated and instead produce a sum signal.

FIA-MS/MS based metabolites

The FIA-MS/MS analysis of the MxP® Quant 500 XL kit enables the quantification of hexoses and up to 912 lipids from 25 biochemical classes. FIA-MS/MS analysis does not provide specific information regarding either the positions or chain lengths of the fatty acid residues linked to each lipid's backbone. Consequently, the detected signal is again a sum of several isobaric/isomeric lipids (Figure 1). For example, according to the LIPID MAPS® database (lipidmaps.org [1]), the signal of PC 36:6 may arise from at least 15 different lipid species that have different compositions of fatty acid residues (e.g. PC(16:1/20:5) versus PC(18:4/18:2)), various positioning of fatty acid residues at sn-1/sn-2 (e.g. PC(18:4/18:2) versus PC(18:2/18:4)), or different double bond positions and stereochemistry in those fatty acid residues (e.g. PC(18:4(6Z,9Z,12Z,15Z)/18:2(9Z,12Z)) versus PC(18:4(9E,11E,13E,15E)/18:2(9Z,12Z))). The current annotation of the lipid signals in the MxP® Quant 500 XL kit denotes representative compounds under individually measured signals.

Annotation of acylcarnitines

- The number of carbon atoms and, if present, double bonds in the fatty acid residue are denoted as 'Cx:y', with x as the number of carbon atoms and y as the number of double bonds.
- The presence of a hydroxyl group (OH) or a (methyl-)dicarboxyl group (DC (-M)) is indicated.

Annotation of (lyso-)phosphatidic acids ((L)PAs)

- For LPAs, the fatty acid residue is bound to the glycerol backbone via an ester bond, usually at the sn-1 position.
- The number of carbon atoms and double bonds present in the fatty acid residue(s) are denoted as 'LPA x:y' and 'PA x:y_x:y', respectively, with x as the number of carbon atoms and y as the number of double bonds.
- '_' indicates that the positions (sn-1/sn-2) of the fatty acid residues are unknown.

Annotation of (lyso-)phosphatidylcholines ((L)PCs)

- For LPCs, the fatty acid residue is bound to the glycerol backbone via an ester bond, usually at the sn-1 position.
- For PCs, both fatty acid residues, at the sn-1 and sn-2 position, are bound to the glycerol backbone via ester bonds.
- 'O-' denotes that one of the moieties, either at the sn-1 or sn-2 position, is a fatty alcohol residue bound to the glycerol backbone via an ether bond.

- The total number of carbon atoms and double bonds present in the fatty acid residue(s) are denoted as ‘LPC x:y’ and ‘PC x:y’, respectively, with x as the total number of carbon atoms and y as the total number of double bonds.

Annotation of (lyso-)phosphatidylethanolamines ((L)PEs)

- For LPEs, the fatty acid residue is bound to the glycerol backbone via an ester bond, usually at the sn-1 position.
- For PEs, both fatty acid residues, at the sn-1 and sn-2 position, are bound to the glycerol backbone via ester bonds.
- ‘P-’ denotes a plasmalogen, meaning that a fatty alcohol residue is bound to the glycerol backbone via a vinyl ether bond at sn-1 position.
- The (total) number of carbon atoms and double bonds present in the fatty acid residue(s) are denoted as ‘LPE x:y’, ‘LPE P-x:y’, ‘PE x:y’, and ‘PE P-x:y/x:y’, respectively, with x as the (total) number of carbon atoms and y as the (total) number of double bonds.

Annotation of (lyso-)phosphatidylglycerols ((L)PGs)

- For LPGs, the fatty acid residue is bound to the glycerol backbone via an ester bond, usually at the sn-1 position.
- For PGs, both fatty acid residues, at the sn-1 and sn-2 position, are bound to the glycerol backbone via ester bonds.
- The number of carbon atoms and double bonds present in the fatty acid residue(s) are denoted as ‘LPG x:y’ and ‘PG x:y_x:y’, respectively, with x as the number of carbon atoms and y as the number of double bonds.
- ‘_’ indicates that the positions (sn-1/sn-2) of the fatty acid residues are unknown.

Annotation of (lyso-)phosphatidylinositols ((L)PIs)

- For LPIs, the fatty acid residue is bound to the glycerol backbone via an ester bond, usually at the sn-1 position.
- For PIs, both fatty acid residues, at the sn-1 and sn-2 position, are bound to the glycerol backbone via ester bonds.
- The number of carbon atoms and double bonds present in the fatty acid residue(s) are denoted as ‘LPI x:y’ and ‘PI x:y_x:y’, respectively, with x as the number of carbon atoms and y as the number of double bonds.
- ‘_’ indicates that the positions (sn-1/sn-2) of the fatty acid residues are unknown.

Annotation of (lyso-)phosphatidylserines ((L)PSs)

- For LPSs, the fatty acid residue is bound to the glycerol backbone via an ester bond.
- For PSs, both fatty acid residues, at the sn-1 and sn-2 position, are bound to the glycerol backbone via ester bonds.
- The total number of carbon atoms and double bonds present in the fatty acid residue(s) are denoted as ‘LPS x:y’ and ‘PS x:y’, respectively, with x as the total number of carbon atoms and y as the total number of double bonds.

Annotation of sphinganines and sphingosines and their phosphates

- The number of carbon atoms and double bonds present in the sphingoid bases (SPBs) and their phosphates (SPBPs) are denoted as 'SPB dx:y' and 'SPBP dx:y', respectively, with x as the number of carbon atoms and y as the number of double bonds.

Annotation of sphingomyelins (SMs)

- The total number of carbon atoms and double bonds present in both chains are denoted as 'SM x:y', with x as the total number of carbon atoms and y as the total number of double bonds.

Annotation of ((di-/tri-)hexosyl-/dihydro-)ceramides

- The number of carbon atoms and double bonds present in the sphingosine or sphinganine backbone (dx:y) and in the fatty acid residue (u:v) are denoted as 'Cer dx:y/u:v', 'Hex-Cer dx:y/u:v', 'Hex2Cer dx:y/u:v', and 'Hex3Cer dx:y/u:v', respectively, with x and u as the number of carbon atoms and y and v as the number of double bonds.
- The presence of a hydroxyl group (OH) is indicated.

Annotation of cholesteryl esters (CEs)

- The number of carbon atoms and double bonds present in the fatty acid residue are denoted as 'CE x:y', with x as the number of carbon atoms and y as the number of double bonds.

Annotation of mono-/di-/triglycerides (MG/DG/TG)

- The number of carbon atoms and double bonds present in the fatty acid residue(s) are denoted as 'MG x:y', 'DG x:y_x:y', and 'TG x:y_u:v', respectively, with x as the number of carbon atoms and y as the number of double bonds, and u as the total number of carbon atoms and v as the total number of double bonds of two fatty acid residues.
- '_' indicates that the positions (sn-1/sn-2/sn-3) of the fatty acid residues are unknown.

Isobars – molecules with the same nominal mass (sum of the masses of the most abundant isotopes), but differing exact masses.

Lipid species	Molecular formula	Nominal mass	Exact mass
PC 34:4	C42H76NO8P	753	753.5309
PC O-35:4	C43H80NO7P	753	753.5670

Isomers – molecules with the same molecular formula, but with different chemical structure.

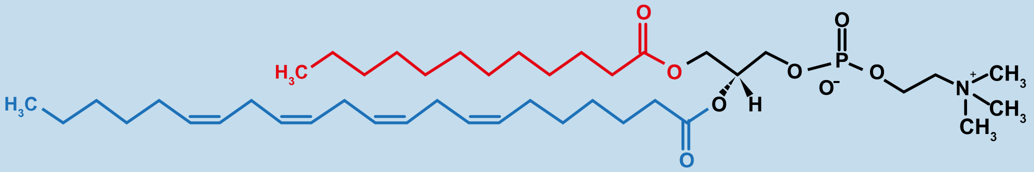

PC(12:0/22:4(7Z, 10Z, 13Z, 16Z))
LIPID MAPS® ID: LMGP01011337
Molecular formula: C42H76NO8P

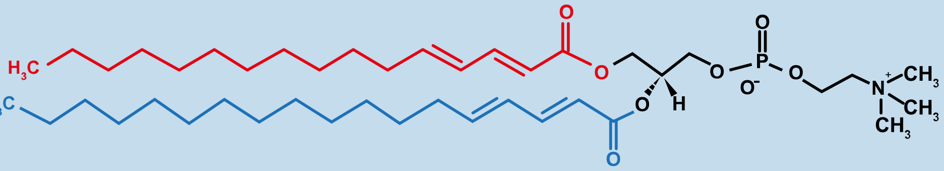

PC(16:2(2E,4E)/18:2(2E,4E))
LIPID MAPS® ID: LMGP01010699
Molecular formula: C42H76NO8P

Figure 1. Explanation of isobaric and isomeric molecules.

Commonly used lipid annotation

Presently, the terminology for lipid annotation defined by the LIPID MAPS® consortium [2, 3] is the most commonly accepted. The LIPID MAPS® nomenclature covers the full structural information of lipid molecules including fatty acyl/alkyl bond type, specific backbone positions of fatty acid/alcohol residues, and position and stereochemistry of double bonds within the fatty acid/alcohol residues.

However, commonly employed MS analyses (including triple quadrupole and high-resolution MS) do not automatically provide such structural details without additional analytical steps that are not well suited for high throughput techniques (e.g. chemical derivatization or complex separation techniques). In 2013, Liebisch et al. [4] proposed a lipid notation that aimed to add defined levels of information below the LIPID MAPS® nomenclature corresponding to the amount of structural information achievable with commonly employed MS analyses, including the MxP® Quant 500 XL kit. The measurement confidence as well as complexity of employed techniques increases with each level, hence the number of possible lipid molecules contributing to the measured signal decreases (Figure 2).

Lipid species	Bond type	Fatty acyl/alkyl composition	Fatty acyl/alkyl & double bond position
MxP® Quant 500 XL kit lipid annotation	Potential isobars within ± 0.5 Da	Potential isomers	Data base ID
PC 24:0	PC 24:0	PC 12:0_12:0	LMGP01010429
		PC 6:0_18:0	LMGP01011228
PC 26:0	PC 26:0	PC 10:0_16:0	LMGP01010388
		PC 12:0_14:0	LMGP01010432; LMGP01010475
		PC 13:0_13:0	LMGP01010456
		PC 17:0_9:0	LMGP01010725
		PC 8:0_18:0	LMGP01011243
	[^{13}C] SM 30:0	-	-

Figure 2. Examples of different confidence levels of lipid measurements.

Isobaric/isomeric lipid species measured with the MxP® Quant 500 XL kit

- All potential isobaric/isomeric lipid species (based on the LIPID MAPS® database) of particular lipid signals measured with the MxP® Quant 500 XL kit are summarized in this annotation list. The list comprises examples of potential isobars/isomers to the best of our current knowledge.
- Potential isobars are given within ± 0.5 Da range due to the typical conditions under which triple quadrupole mass spectrometers are operated for the kit and isobars are reported as sum compositions.
- The position of the fatty acid residues and double bonds are not indicated for the potential isomers in case of (lyso-)phosphatidic acids, (lyso-)phosphatidylcholines, (lyso-)phosphatidylethanolamines, (lyso-)phosphatidylglycerols, (lyso-)phosphatidylinositols, (lyso-)phosphatidylserines, monoglycerides, diglycerides, and triglycerides.
- For each isomer, examples from LIPID MAPS® structural database entries are listed (lipidmaps.org/data/databases.html [1]).
- For several lipids the prefix '[^{13}C]' indicates the possible isotope interference from another lipid species having a molecular weight with one mass unit smaller. These interferences have not been removed by the applied isotope correction algorithm due to the limitation of current MS/MS hardware in the maximal number of MRM transitions that can be monitored in parallel.

Abbreviations and separators

CE	Cholesteryl ester
Cer	Dihydro-/Ceramide
DG	Diglyceride
Gal	Galactose
Glc	Glucose
Hex-Cer	Hexosylceramide
Hex2Cer	Dihexosylceramide
Hex3Cer	Trihexosylceramide
Lac	Lactose
LPA	Lysophosphatidic acid
LPC	Lysophosphatidylcholine
LPE	Lysophosphatidylethanolamine
LPG	Lysophosphatidylglycerol
LPI	Lysophosphatidylinositol
LPS	Lysophosphatidylserine
Man	Mannose
MG	Monoglyceride
O-	O-alkyl bond
OH	Hydroxyl group
P-	Plasmalogen (vinyl ether)
PA	Phosphatidic acid
PC	Phosphatidylcholine
PE	Phosphatidylethanolamine
PG	Phosphatidylglycerol
PI	Phosphatidylinositol
PS	Phosphatidylserine
SM	Sphingomyelin
SPB	Sphingoid base
SPBP	Sphingoid base phosphate
TG	Triglyceride
[^{13}C]	Indicates possible isotope interference from another lipid species having a molecular weight with one mass unit smaller. This interference has not been corrected due to technical limitation.
_	Separator for fatty acid residues, if sn-position is not known
/	Separator for fatty acid residues, if sn-position is proven (sn1/sn2)

References

1. Sud et al. LMSD: LIPID MAPS structure database. Nucleic Acids Res 2007; 35 (Database issue):D527-32, DOI:10.1093/nar/gkl838.
2. Fahy et al. Update of the LIPID MAPS comprehensive classification system for lipids. J. Lipid Res. 2009; 50 Suppl:S9-14, DOI:10.1194/jlr.R800095-JLR200.
3. Liebisch et al. Update on LIPID MAPS classification, nomenclature, and shorthand notation for MS-derived lipid structures. J. Lipid Res. 2020; 61(12):1539–55. DOI:10.1194/jlr.S120001025.
4. Liebisch et al. Shorthand notation for lipid structures derived from mass spectrometry. J. Lipid Res. 2013; 54(6):1523–30, DOI:10.1194/jlr.M033506.

MxP® Quant 500 XL kit		
Acylcarnitines (40)		
MxP® Quant 500 XL kit lipid annotation	Potential isomer	Data base ID
C0	Carnitine	CHEBI:17126
C2	Acetyl-D-carnitine	LMFA07070049
	Acetyl-L-carnitine	LMFA07070050
	DL-Acetylcarnitine	LMFA07070060
C3	O-Propanoylcarnitine	LMFA07070005
	O-Propanoyl-D-carnitine	LMFA07070099
	Propionylcarnitine	LMFA07070105
C3-DC (C4-OH)	Malonylcarnitine	LMFA07070080
	O-Malonylcarnitine	LMFA07070093
	3-Hydroxybutyrylcarnitine	LMFA07070037
	Hydroxybutyrylcarnitine	LMFA07070071
C3-OH	Hydroxypropionylcarnitine	LMFA07070074
C3:1	Propenoylcarnitine	LMFA07070104
C4	O-Butanoylcarnitine	LMFA07070003
	Butyrylcarnitine	LMFA07070054
	Isobutyryl-L-carnitine	LMFA07070075
C4:1	Butenylcarnitine	LMFA07070053
C5	2-Methylbutyroylcarnitine	LMFA07070034
	Isovalerylcarnitine	LMFA07070076
	Isovaleryl-L-carnitine	LMFA07070077
	Pivaloylcarnitine	LMFA07070103
	Valerylcarnitine	LMFA07070111
C5-DC (C6-OH)	Glutarylcarnitine	LMFA07070066
	O-Glutarylcarnitine	LMFA07070091
	Hydroxyhexanoylcarnitine	LMFA07070072
C5-M-DC	O-Adipoylcarnitine	LMFA07070087
C5-OH (C3-DC-M)	Methylmalonylcarnitine	LMFA07070081
	O-Methylmalonylcarnitine	LMFA07070094
	O-Succinylcarnitine	LMFA07070101
	3-Hydroxyisovalerylcarnitine	LMFA07070041
	Hydroxyisovaleroylcarnitine	LMFA07070073
C5:1	2-Ethylacryloylcarnitine	LMFA07070030
	Tiglylcarnitine	LMFA07070108
C5:1-DC	Glutaconylcarnitine	LMFA07070065
	Heptanoylcarnitine	LMFA07070068
C6 (C4:1-DC)	Fumarylcarnitine	LMFA07070064
	O-Hexanoyl-R-carnitine	LMFA07070001
	Hexanoylcarnitine	LMFA07070070
C6:1	2-Hexenoylcarnitine	LMFA07070031
C7-DC	O-Pimeloylcarnitine	CHEBI:73049
C8	O-Octanoyl-R-carnitine	LMFA07070002
	O-Octanoylcarnitine	LMFA07070095
C9	3-Hydroxy-cis-5-octenoylcarnitine	LMFA07070038
	2,6 Dimethylheptanoylcarnitine	LMFA07070029
	Nonanoylcarnitine	LMFA07070082
C10	O-Decanoyl-R-carnitine	LMFA07070006
	Decanoylcarnitine	LMFA07070059
C10:1	(4Z)-Decenoylcarnitine	LMFA07070017
	9-Decenoylcarnitine	LMFA07070048
	cis-4-Decenoylcarnitine	LMFA07070056
C10:2	(2E,4Z)-Decadienoylcarnitine	LMFA07070015

MxP® Quant 500 XL kit lipid annotation	Potential isomer	Data base ID
C12	Dodecanoylcarnitine	LMFA07070062
	O-Dodecanoylcarnitine	LMFA07070090
C12-DC	O-(11-Carboxyundecanoyl)carnitine	LMFA07070083
C12:1	O-Dodecenoylcarnitine	CHEBI:86065
C14	O-Tetradecanoylcarnitine	LMFA07070102
	Tetradecanoylcarnitine	LMFA07070107
C14:1	cis-5-Tetradecenoylcarnitine	LMFA07070057
C14:1-OH	O-(Hydroxytetradecenoyl)carnitine	CHEBI:86067
	Pentadecanoylcarnitine	PubChem:123132011
C14:2	(5Z,8Z)-Tetradecadienoylcarnitine	LMFA07070020
C14:2-OH	(5Z,8Z)-3-Hydroxytetradecadienoylcarnitine	LMFA07070019
C16	(5Z)-13-Carboxytridec-5-enoylcarnitine	LMFA07070018
	Palmitoylcarnitine	LMFA07070004
	L-Palmitoylcarnitine	LMFA07070079
	O-Palmitoylcarnitine	LMFA07070098
C16-OH	2-Hydroxyhexadecanoylcarnitine	LMFA07010720
C16:1	O-Palmitoleoylcarnitine	LMFA07070097
	trans-Hexadec-2-enoylcarnitine	LMFA07070109
C16:1-OH	3-Hydroxypalmitoleoylcarnitine	LMFA07070044
	Heptadecanoylcarnitine	LMFA07070067
C16:2	(7Z,10Z)-Hexadecadienoylcarnitine	LMFA07070021
C16:2-OH	(9Z,12Z)-3-Hydroxyhexadecadienoylcarnitine	LMFA07070026
C18	Stearoylcarnitine	LMFA07070008
	Acylcarnitine C18:0	LMFA07070051
C18:1	Elaidiccarnitine	LMFA07070063
	O-Oleoylcarnitine	LMFA07070096
C18:1-OH	(9Z)-3-Hydroxyoctadecenoylcarnitine	LMFA07070025
	Nonadecanoylcarnitine	-
C18:2	9,12-Hexadecadienylcarnitine	LMFA07070009
	Linoelaidylcarnitine	LMFA07070078
	O-Linoleoylcarnitine	LMFA07070092

Lysophosphatidic acids (8)			
MxP® Quant 500 XL kit lipid annotation	Potential isobar within ± 0.5 Da	Potential isomer	Data base ID
LPA 14:0	LPA 14:0	PA(14:0/0:0)	LMGP10050007
LPA 14:1	LPA 14:1	PA(14:1(9Z)/0:0)	LMGP10050038
LPA 15:0	LPA 15:0	PA(15:0/0:0)	LMGP10050037
LPA 16:0	LPA 16:0	PA(16:0/0:0)	LMGP10050006
		PA(0:0/16:0)	LMGP10050042
LPA 18:1	LPA 18:1	PA(18:1(9Z)/0:0)	LMGP10050008
		PA(0:0/18:1(9Z))	LMGP10050014
LPA 18:2	LPA 18:2	PA(18:2(9Z,12Z)/0:0)	LMGP10050017
		PA(0:0/18:2(9Z,12Z))	LMGP10050044
LPA 22:3	LPA 22:3	LPA 22:3	PubChem:138305222
LPA 22:4	LPA 22:4	PA(22:4(7Z,10Z,13Z,16Z)/0:0)	LMGP10050020

Phosphatidic acids (41)

MxP® Quant 500 XL kit lipid annotation	Potential isobar within ± 0.5 Da	Potential isomer	Data base ID
PA 14:0_14:1	DG 34:3	DG(14:1(9Z)/20:2(11Z,14Z)/0:0)[iso2]	LMGL02010414
	PA 14:0_14:1	PA(14:0/14:1(9Z))	LMGP10010092
		PA(14:1(9Z)/14:0)	LMGP10010109
PA 16:0_18:1	DG 40:3	DG(18:1(9Z)/22:2(13Z,16Z)/0:0)[iso2]	LMGL02010193
	PA 16:0_18:1	PA(16:0/18:1(11Z))	LMGP10010007
		PA(16:0/18:1(9Z))	LMGP10010032
		PA(18:1(9Z)/16:0)	LMGP10010964
PA 16:0_18:2	DG 40:4	DG(18:2(9Z,12Z)/22:2(13Z,16Z)/0:0)[iso2]	LMGL02010203
	PA 16:0_18:2	PA(16:0/18:2(9Z,12Z))	LMGP10010023
		PA(18:2(9Z,12Z)/16:0)	LMGP10010877
PA 16:0_18:3	DG 40:5	DG(18:3(9Z,12Z,15Z)/22:2(13Z,16Z)/0:0)[iso2]	LMGL02010213
		DG(18:3(6Z,9Z,12Z)/22:2(13Z,16Z)/0:0)[iso2]	LMGL02010493
	PA 16:0_18:3	PA(18:3(6Z,9Z,12Z)/16:0)	LMGP10010370
		PA(16:0/18:3(6Z,9Z,12Z))	LMGP10010907
		PA(18:3(9Z,12Z,15Z)/16:0)	LMGP10010956
		PA(16:0/18:3(9Z,12Z,15Z))	LMGP10010975
PA 16:0_19:2	PA 16:0_19:2	-	-
PA 16:1_18:1	DG 40:4	DG(18:1(9Z)/22:3(10Z,13Z,16Z)/0:0)[iso2]	LMGL02010181
	PA 16:1_18:1	PA(18:1(9Z)/16:1(9Z))	LMGP10010881
		PA(16:1(9Z)/18:1(9Z))	LMGP10010903
PA 16:1_18:2	DG 40:5	DG(18:2(9Z,12Z)/22:3(10Z,13Z,16Z)/0:0)[iso2]	LMGL02010192
	PA 16:1_18:2	PA(18:2(9Z,12Z)/16:1(9Z))	LMGP10010344
		PA(16:1(9Z)/18:2(9Z,12Z))	LMGP10010902
PA 16:1_22:0	FAHFA 48:2;O	FAHFA(16:1/32-O-32:1)	LMFA07090161
		FAHFA(16:1/32-O-32:1)	LMFA07090159
		FAHFA(16:1/32-O-32:1)	LMFA07090160
	PG 33:3	PG(16:1(9Z)/17:2(9Z,12Z))	LMGP04010210
		PG(17:2(9Z,12Z)/16:1(9Z))	LMGP04010285
	PA 16:1_22:0	PA(16:1(9Z)/22:0)	LMGP10010220
		PA(22:0/16:1(9Z))	LMGP10010705
PA 16:2_18:1	DG 40:5	DG(18:1(9Z)/22:4(7Z,10Z,13Z,16Z)/0:0)[iso2]	LMGL02010204
	PA 16:2_18:1	-	-
PA 17:0_18:1	PA 17:0_18:1	PA(18:1(9Z)/17:0)	LMGP10010325
		PA(17:0/18:1(9Z))	LMGP10010894
	PA O-36:1	PA(O-18:0/18:1(9Z))	LMGP10020028
PA 17:0_18:2	PA 17:0_18:2	PA(17:0/18:2(9Z,12Z))	LMGP10010231
		PA(18:2(9Z,12Z)/17:0)	LMGP10010345
	PA O-36:2	PA(O-18:0/18:2(9Z,12Z))	LMGP10020029
PA 17:0_18:3	PA 17:0_18:3	PA(17:0/18:3(6Z,9Z,12Z))	LMGP10010232
		PA(17:0/18:3(9Z,12Z,15Z))	LMGP10010233
		PA(18:3(6Z,9Z,12Z)/17:0)	LMGP10010372
		PA(18:3(9Z,12Z,15Z)/17:0)	LMGP10010402
	PA O-36:3	PA(O-18:0/18:3(6Z,9Z,12Z))	LMGP10020030
		PA(O-18:0/18:3(9Z,12Z,15Z))	LMGP10020031
PA 17:1_18:1	PA 17:1_18:1	PA(17:1(9Z)/18:1(9Z))	LMGP10010257
		PA(18:1(9Z)/17:1(9Z))	LMGP10010326
	PA O-36:2	PA(P-18:0/18:1(9Z))	LMGP10030092

MxP® Quant 500 XL kit lipid annotation	Potential isobar within ± 0.5 Da	Potential isomer	Data base ID
PA 17:1_18:2	PA 17:1_18:2	PA(17:1(9Z)/18:2(9Z,12Z))	LMGP10010258
		PA(18:2(9Z,12Z)/17:1(9Z))	LMGP10010346
	PA O-36:3	PA(P-18:0/18:2(9Z,12Z))	LMGP10030040
PA 17:2_18:1	PA 17:2_18:1	PA(17:2(9Z,12Z)/18:1(9Z))	LMGP10010287
		PA(18:1(9Z)/17:2(9Z,12Z))	LMGP10010327
PA 18:0_18:1	PA 18:0_18:1	PA(18:0/18:1(9Z))	LMGP10010037
		PA(18:1(9Z)/18:0)	LMGP10010963
PA 18:0_18:2	PA 18:0_18:2	PA(18:0/18:2(9Z,12Z))	LMGP10010036
		PA(18:2(9Z,12Z)/18:0)	LMGP10010876
PA 18:0_18:3	PA 18:0_18:3	PA(18:0/18:3(6Z,9Z,12Z))	LMGP10010313
		PA(18:0/18:3(9Z,12Z,15Z))	LMGP10010314
		PA(18:3(6Z,9Z,12Z)/18:0)	LMGP10010375
		PA(18:3(9Z,12Z,15Z)/18:0)	LMGP10010405
PA 18:1_18:1	PA 18:1_18:1	PA(18:1(9Z)/18:1(9Z))	LMGP10010962
	SM 34:2;O2	SM(d16:1/18:1)	LMSP03010040
PA 18:1_18:2	PA 18:1_18:2	PA(18:2(9Z,12Z)/18:1(9Z))	LMGP10010348
		PA(18:1(9Z)/18:2(9Z,12Z))	LMGP10010961
PA 18:1_18:3	PA 18:1_18:3	PA(18:1(9Z)/18:3(6Z,9Z,12Z))	LMGP10010328
		PA(18:3(6Z,9Z,12Z)/18:1(9Z))	LMGP10010376
		PA(18:3(9Z,12Z,15Z)/18:1(9Z))	LMGP10010873
		PA(18:1(9Z)/18:3(9Z,12Z,15Z))	LMGP10010880
PA 18:1_18:4	PA 18:1_18:4	PA(18:1(9Z)/18:4(6Z,9Z,12Z,15Z))	LMGP10010329
		PA(18:4(6Z,9Z,12Z,15Z)/18:1(9Z))	LMGP10010435
PA 18:1_20:0	PA 18:1_20:0	PA(20:0/18:1(9Z))	LMGP10010953
		PA(18:1(9Z)/20:0)	LMGP10010960
	FAHFA 48:2;O	FAHFA(18:1/30-O-30:1)	LMFA07090158
	SM 36:1;O2	SM(d18:0/18:1(9Z))	LMSP03010031
PA 18:1_20:1	MGDG 32:1	MGDG(14:0/18:1(9Z))	LMGL05010055
		MGDG(18:1(9Z)/14:0)	LMGL05010075
	PA 18:1_20:1	PA(20:1(11Z)/18:1(9Z))	LMGP10010538
		PA(18:1(9Z)/20:1(11Z))	LMGP10010959
PA 18:1_20:2	PA 18:1_20:2	PA(18:1(9Z)/20:2(11Z,14Z))	LMGP10010332
		PA(20:2(11Z,14Z)/18:1(9Z))	LMGP10010568
	SM 36:3;O2	SM(d18:2/18:1)	LMSP03010047
PA 18:1_20:3	PA 18:1_20:3	PA(18:1(9Z)/20:3(8Z,11Z,14Z))	LMGP10010333
		PA(20:3(8Z,11Z,14Z)/18:1(9Z))	LMGP10010599
PA 18:1_22:0	FAHFA 50:2;O	FAHFA(18:1/32-O-32:1)	LMFA07090154
		FAHFA(18:1/32-O-32:1)	LMFA07090155
		FAHFA(18:1/32-O-32:1)	LMFA07090156
	PG 17:2_18:1	PG(17:2(9Z,12Z)/18:1(9Z))	LMGP04010289
		PG(18:1(9Z)/17:2(9Z,12Z))	LMGP04010329
	PA 18:1_22:0	PA(22:0/18:1(9Z))	LMGP10010947
		PA(18:1(9Z)/22:0)	LMGP10010958
PA 18:1_22:1	MGDG 34:1	MGDG(16:0/18:1(9Z))	LMGL05010025
	PA 18:1_22:1	PA(18:1(9Z)/22:1(11Z))	LMGP10010335
		PA(22:1(11Z)/18:1(9Z))	LMGP10010736
PA 18:1_22:2	PA 18:1_22:2	PA(18:1(9Z)/22:2(13Z,16Z))	LMGP10010336
		PA(22:2(13Z,16Z)/18:1(9Z))	LMGP10010767
	PPA 34:1	PPA(16:0/18:1(9Z))	LMGP11010001
PA 18:1_22:3	-	-	-
PA 18:2_18:2	PA 18:2_18:2	PA(18:2(9Z,12Z)/18:2(9Z,12Z))	LMGP10010957

MxP® Quant 500 XL kit lipid annotation	Potential isobar within ± 0.5 Da	Potential isomer	Data base ID
PA 18:2_18:3	PA 18:2_18:3	PA(18:2(9Z,12Z)/18:3(6Z,9Z,12Z))	LMGP10010349
		PA(18:2(9Z,12Z)/18:3(9Z,12Z,15Z))	LMGP10010350
		PA(18:3(6Z,9Z,12Z)/18:2(9Z,12Z))	LMGP10010377
		PA(18:3(9Z,12Z,15Z)/18:2(9Z,12Z))	LMGP10010406
PA 18:2_20:0	PA 18:2_20:0	PA(18:2(9Z,12Z)/20:0)	LMGP10010354
		PA(20:0/18:2(9Z,12Z))	LMGP10010952
	SM 36:2;O2	SM(d18:0/18:2)	LMSP03010049
PA 18:2_20:1	MGDG 32:2	MGDG(14:0/18:2(9Z,12Z))	LMGL05010051
		MGDG(18:2(9Z,12Z)/14:0)	LMGL05010074
	PA 18:2_20:1	PA(18:2(9Z,12Z)/20:1(11Z))	LMGP10010355
		PA(20:1(11Z)/18:2(9Z,12Z))	LMGP10010539
PA 18:2_20:2	PA 18:2_20:2	PA(18:2(9Z,12Z)/20:2(11Z,14Z))	LMGP10010356
		PA(20:2(11Z,14Z)/18:2(9Z,12Z))	LMGP10010569
PA 18:2_22:0	PG 17:2_18:2	PG(17:2(9Z,12Z)/18:2(9Z,12Z))	LMGP04010290
		PG(18:2(9Z,12Z)/17:2(9Z,12Z))	LMGP04010349
	PA 18:2_22:0	PA(18:2(9Z,12Z)/22:0)	LMGP10010360
		PA(22:0/18:2(9Z,12Z))	LMGP10010709
PA 18:2_22:1	MGDG 34:2	MGDG(16:0/18:2(9Z,12Z))	LMGL05010026
		MGDG(18:2(9Z,12Z)/16:0)	LMGL05010077
	PA 18:2_22:1	PA(18:2(9Z,12Z)/22:1(11Z))	LMGP10010361
		PA(22:1(11Z)/18:2(9Z,12Z))	LMGP10010737
PA 18:2_22:3	-	-	-
PA 18:2_22:4	PA 18:2_22:4	PA(18:2(9Z,12Z)/22:4(7Z,10Z,13Z,16Z))	LMGP10010363
		PA(22:4(7Z,10Z,13Z,16Z)/18:2(9Z,12Z))	LMGP10010799
PA 18:3_18:3	PA 18:3_18:3	PA(18:3(9Z,12Z,15Z)/18:3(9Z,12Z,15Z))	LMGP10010016
		PA(18:3(6Z,9Z,12Z)/18:3(6Z,9Z,12Z))	LMGP10010378
		PA(18:3(6Z,9Z,12Z)/18:3(9Z,12Z,15Z))	LMGP10010379
		PA(18:3(9Z,12Z,15Z)/18:3(6Z,9Z,12Z))	LMGP10010407
PA 20:0_20:4	PA 20:0_20:4	PA(20:4(5Z,8Z,11Z,14Z)/20:0)	LMGP10010636
		PA(20:0/20:4(5Z,8Z,11Z,14Z))	LMGP10010861

Lysophosphatidylcholines (12)

MxP® Quant 500 XL kit lipid annotation	Potential isobar within ± 0.5 Da	Potential isomer	Data base ID
LPC 14:0	LPC 14:0	PC 14:0_0:0	LMGP01050012; LMGP01050073
	PC O-14:0	PC O-12:0_2:0	LMGP01020009
	LPC O-15:0	PC O-15:0_0:0	LMGP01060009
LPC 16:0	LPC 16:0	PC 16:0_0:0	LMGP01050018; LMGP01050074
	PC O-16:0	PC O-14:0_2:0	LMGP01020019
	LPC O-17:0	PC O-17:0_0:0	LMGP01060013

MxP® Quant 500 XL kit lipid annotation	Potential isobar within ± 0.5 Da	Potential isomer	Data base ID
LPC 16:1	LPC 16:1	PC 16:1_0:0	LMGP01050021; LMGP01050022
	PC O-17:1	PC O-17:1_0:0	LMGP01070007
LPC 17:0	LPC 17:0	PC 17:0_0:0	LMGP01050024
	PC O-17:0	PC O-1:0_16:0	LMGP01020004; LMGP01020028; LMGP01080021
		PC O-15:0_2:0	LMGP01020024
	LPC O-18:0	PC O-18:0_0:0	LMGP01060014
	PC 16:0	PC 14:0_2:0	LMGP01010504
		PC 8:0_8:0	LMGP01011251
LPC 18:0	LPC 18:0	PC 18:0_0:0	LMGP01050026; LMGP01050076
	PC O-18:0	PC O-16:0_2:0	LMGP01020046
	LPC O-19:0	PC O-19:0_0:0	LMGP01060017
LPC 18:1	LPC 18:1	PC 18:1_0:0	LMGP01050029; LMGP01050030; LMGP01050032; LMGP01050079; LMGP01050082; LMGP01050138
	PC O-18:1	PC O-16:1_2:0	LMGP01020147; LMGP01030009
LPC 18:2	LPC 18:2	PC 18:2_0:0	LMGP01050034; LMGP01050035
	PC O-19:2	PC O-19:2_0:0	LMGP01070010
LPC 20:3	LPC 20:3	PC 20:3_0:0	LMGP01050133; LMGP01050139
LPC 20:4	LPC 20:4	PC 20:4_0:0	LMGP01050048; LMGP01050121; LMGP01050140
LPC 24:0	LPC 24:0	PC 24:0_0:0	LMGP01050057
LPC 26:0	LPC 26:0	PC 26:0_0:0	-
	PC 25:0	PC 12:0_13:0	LMGP01010001; LMGP01011338
		PC 16:0_9:0	LMGP01010677
LPC 26:1	LPC 26:1	PC 26:1_0:0	-
	PC 25:1	PC 16:1_9:0	-

Phosphatidylcholines (78)

MxP® Quant 500 XL kit lipid annotation	Potential isobars within ± 0.5 Da	Potential isomers	Data base ID
PC 24:0	PC 24:0	PC 12:0_12:0	LMGP01010429
		PC 6:0_18:0	LMGP01011228
PC 26:0	PC 26:0	PC 10:0_16:0	LMGP01010388
		PC 12:0_14:0	LMGP01010432; LMGP01010475
		PC 13:0_13:0	LMGP01010456
		PC 17:0_9:0	LMGP01010725
		PC 8:0_18:0	LMGP01011243
	[^{13}C] SM 30:0	-	-

MxP® Quant 500 XL kit lipid annotation	Potential isobars within ± 0.5 Da	Potential isomers	Data base ID
PC 28:1	PC 28:1	PC 10:0_18:1	LMGP01010392
		PC 12:0_16:1	LMGP01011319; LMGP01011473
		PC 13:0_15:1	LMGP01011341; LMGP01011432
		PC 14:0_14:1	LMGP01011364; LMGP01011381
	PC O-29:1	PC O-16:1_13:0	LMGP01030020
	[13C] SM 32:1	-	-
PC 30:0	PC 30:0	PC 10:0_20:0	LMGP01010395; LMGP01010995
		PC 11:0_19:0	LMGP01010416; LMGP01010969
		PC 12:0_18:0	LMGP01010438; LMGP01010736
		PC 13:0_17:0	LMGP01010461; LMGP01010702
		PC 14:0_16:0	LMGP01010481; LMGP01010560
		PC 15:0_15:0	LMGP01010530
		PC 9:0_21:0	LMGP01011265
	PC O-31:0	PC O-16:0_15:0	LMGP01020180
		PC O-18:0_13:0	LMGP01020194
	[13C] SM 34:0	-	-
PC 30:2	PC 30:2	PC 12:0_18:2	LMGP01011323; LMGP01011614
		PC 13:0_17:2	LMGP01011345; LMGP01011552
		PC 14:1_16:1	LMGP01011385; LMGP01011476
		PC 15:1_15:1	LMGP01011436
	PC O-31:2	PC O-16:1_15:1	LMGP01030024
PC 32:0	PC 32:0	PC 10:0_22:0	LMGP01010397; LMGP01011080
		PC 11:0_21:0	LMGP01010418; LMGP01011062
		PC 12:0_20:0	LMGP01010444; LMGP01010997
		PC 13:0_19:0	LMGP01010463; LMGP01010970
		PC 14:0_18:0	LMGP01010488; LMGP01010739
		PC 15:0_17:0	LMGP01010537; LMGP01010704
		PC 16:0_16:0	LMGP01010564
		PC 9:0_23:0	LMGP01011267
	PC O-33:0	PC O-16:0_17:0	LMGP01020031
		PC O-18:0_15:0	LMGP01020197
		PC O-20:0_13:0	LMGP01020217
	[13C] SM 36:0	-	-

MxP® Quant 500 XL kit lipid annotation	Potential isobars within ± 0.5 Da	Potential isomers	Data base ID
PC 32:1	PC 32:1	PC 14:0_18:1	LMGP01010490; LMGP01010492; LMGP01010882; LMGP01012145
		PC 16:0_16:1	LMGP01010566; LMGP01011479
		PC 12:0_20:1	LMGP01011329; LMGP01011805
		PC 13:0_19:1	LMGP01011352; LMGP01011757
		PC 14:1_18:0	LMGP01011389; LMGP01011582
		PC 15:0_17:1	LMGP01011413; LMGP01011525
		PC 15:1_17:0	LMGP01011439; LMGP01011500
	PC O-33:1	PC O-16:0_17:1	LMGP01020183
		PC O-18:0_15:1	LMGP01020198
		PC O-16:1_17:0	LMGP01030027
		PC O-18:1_15:0	LMGP01030050
		PC O-20:1_13:0	LMGP01030075
PC 32:2	PC 32:2	PC 12:0_20:2	LMGP01011330; LMGP01011835
		PC 14:0_18:2	LMGP01010494; LMGP01010496; LMGP01011616
		PC 14:1_18:1	LMGP01011390; LMGP01011597
		PC 15:0_17:2	LMGP01011414; LMGP01011555
		PC 16:1_16:1	LMGP01010682; LMGP01010684
		PC 15:1_17:1	LMGP01011440; LMGP01011526
	PC O-33:2	PC O-16:0_17:2	LMGP01020184
		PC O-16:1_17:1	LMGP01030028
		PC O-18:1_15:1	LMGP01030051
PC 32:3	PC 32:3	PC 14:0_18:3	LMGP01010497; LMGP01011369; LMGP01011642; LMGP01011673
		PC 12:0_20:3	LMGP01011331; LMGP01011866
		PC 14:1_18:2	LMGP01011391; LMGP01011617
		PC 15:1_17:2	LMGP01011441; LMGP01011556
	PC O-33:3	PC O-16:1_17:2	LMGP01030029
	[^{13}C] SM 36:3	-	-

MxP® Quant 500 XL kit lipid annotation	Potential isobars within ± 0.5 Da	Potential isomers	Data base ID
PC 34:1	PC 34:1	PC 12:0_22:1	LMGP01011335; LMGP01012004
		PC 14:0_20:1	LMGP01011372; LMGP01011807
		PC 14:1_20:0	LMGP01011397; LMGP01011787
		PC 15:0_19:1	LMGP01011419; LMGP01011760
		PC 15:1_19:0	LMGP01011448; LMGP01011732
		PC 16:0_18:1	LMGP01010005; LMGP01010575; LMGP01010576; LMGP01010578; LMGP01010581; LMGP01010884; LMGP01012146
		PC 17:0_17:1	LMGP01011503; LMGP01011529
		PC 18:0_16:1	LMGP01010744; LMGP01011483
	PC O-35:1	PC O-17:0_18:1	LMGP01020077
		PC O-16:0_19:1	LMGP01020186
		PC O-18:0_17:1	LMGP01020200
		PC O-20:0_15:1	LMGP01020221
		PC O-16:1_19:0	LMGP01030034
		PC O-18:1_17:0	LMGP01030054
		PC O-20:1_15:0	LMGP01030078
	[^{13}C] SM 38:1	-	-

MxP® Quant 500 XL kit lipid annotation	Potential isobars within ± 0.5 Da	Potential isomers	Data base ID
PC 34:2	PC 34:2	PC 12:0_22:2	LMGP01011336; LMGP01012035
		PC 14:0_20:2	LMGP01011373; LMGP01011837
		PC 14:1_20:1	LMGP01011398; LMGP01011808
		PC 15:1_19:1	LMGP01011449; LMGP01011761
		PC 16:0_18:2	LMGP01010585; LMGP01010586; LMGP01010587; LMGP01010588; LMGP01010590; LMGP01010591; LMGP01010592; LMGP01010594; LMGP01010920; LMGP01010926; LMGP01010932
		PC 16:1_18:1	LMGP01010678; LMGP01010687; LMGP01010688; LMGP01010887
		PC 17:0_17:2	LMGP01011504; LMGP01011559
		PC 17:1_17:1	LMGP01010727; LMGP01010728
		PC 18:0_16:2	LMGP01010745
	PC O-35:2	PC O-18:0_17:2	LMGP01020201
		PC O-16:1_19:1	LMGP01030035
		PC O-18:1_17:1	LMGP01030055
		PC O-20:1_15:1	LMGP01030079
	[^{13}C] SM 38:2	-	-
PC 34:3	PC 34:3	PC 14:0_20:3	LMGP01011374; LMGP01011868; LMGP01012127; LMGP01012192
		PC 14:1_20:2	LMGP01011399; LMGP01011838
		PC 16:0_18:3	LMGP01010598; LMGP01010601; LMGP01011646; LMGP01011677
		PC 16:1_18:2	LMGP01010690; LMGP01011620
		PC 17:1_17:2	LMGP01011530; LMGP01011560
	PC O-35:3	PC O-18:1_17:2	LMGP01030056

MxP® Quant 500 XL kit lipid annotation	Potential isobars within ± 0.5 Da	Potential isomers	Data base ID
PC 34:4	PC 34:4	PC 12:0_22:4	LMGP01011337; LMGP01012066
		PC 14:0_20:4	LMGP01010506; LMGP01011899; LMGP01012128; LMGP01012209
		PC 14:1_20:3	LMGP01011400; LMGP01011869; LMGP01012132; LMGP01012193
		PC 16:0_18:4	LMGP01010603; LMGP01010604; LMGP01010606; LMGP01010608; LMGP01011706
		PC 16:1_18:3	LMGP01011484; LMGP01011485; LMGP01011647; LMGP01011678
		PC 16:2_18:2	LMGP01010699
		PC 17:2_17:2	LMGP01010731
	PC O-35:4	PC O-15:0_20:4	LMGP01020026
PC 36:0	PC 36:0	PC 11:0_25:0	LMGP01010422
		PC 12:0_24:0	LMGP01010449
		PC 13:0_23:0	LMGP01010468
		PC 14:0_22:0	LMGP01010511; LMGP01011085
		PC 15:0_21:0	LMGP01010549; LMGP01011956
		PC 16:0_20:0	LMGP01010616; LMGP01011002
		PC 17:0_19:0	LMGP01011509; LMGP01011735
		PC 18:0_18:0	LMGP01010006
	PC 37:7	PC 15:1_22:6	LMGP01011461; LMGP01012102
		PC 17:2_20:5	LMGP01011574; LMGP01011936
	PC O-37:0	PC O-17:0_20:0	LMGP01020080; LMGP01020223
		PC O-16:0_21:0	LMGP01020189
		PC O-18:0_19:0	LMGP01020207
	PC O-38:7	PC O-16:1_22:6	LMGP01030015
		PC O-18:2_20:5	LMGP01090051; LMGP01090052
	[^{13}C] SM 40:0	-	-

MxP® Quant 500 XL kit lipid annotation	Potential isobars within ± 0.5 Da	Potential isomers	Data base ID
PC 36:1	PC 36:1	PC 14:0_22:1	LMGP01011376; LMGP01012006; LMGP01012129; LMGP01012218
		PC 14:1_22:0	LMGP01011404; LMGP01011980
		PC 15:1_21:0	LMGP01011456; LMGP01011957
		PC 16:0_20:1	LMGP01011468; LMGP01011811
		PC 16:1_20:0	LMGP01011488; LMGP01011790
		PC 17:0_19:1	LMGP01011510; LMGP01011764
		PC 17:1_19:0	LMGP01011537; LMGP01011736
		PC 18:0_18:1	LMGP01010750; LMGP01010751; LMGP01010753; LMGP01010754; LMGP01010758; LMGP01010759; LMGP01010761; LMGP01010840; LMGP01010888
	PC O-37:1	PC O-18:0_19:1	LMGP01020208
		PC O-20:0_17:1	LMGP01020224
		PC O-16:1_21:0	LMGP01030041
		PC O-18:1_19:0	LMGP01030062
		PC O-20:1_17:0	LMGP01030082
	[^{13}C] SM 40:1	-	-

MxP® Quant 500 XL kit lipid annotation	Potential isobars within ± 0.5 Da	Potential isomers	Data base ID
PC 36:2	PC 36:2	PC 14:0_22:2	LMGP01011377; LMGP01012037
		PC 14:1_22:1	LMGP01011405; LMGP01012007; LMGP01012219
		PC 16:0_20:2	LMGP01011469; LMGP01011841
		PC 16:1_20:1	LMGP01011489; LMGP01011812
		PC 17:1_19:1	LMGP01011538; LMGP01011765
		PC 17:2_19:0	LMGP01011567; LMGP01011737
		PC 18:0_18:2	LMGP01010764; LMGP01010765; LMGP01010766; LMGP01010768
		PC 18:1_18:1	LMGP01010836; LMGP01010837; LMGP01010841; LMGP01010849; LMGP01010853; LMGP01010855; LMGP01010857; LMGP01010858; LMGP01010860; LMGP01010862; LMGP01010865; LMGP01010866; LMGP01010868; LMGP01010871; LMGP01010873; LMGP01010876; LMGP01010890
	PC O-37:2	PC O-20:0_17:2	LMGP01020225
		PC O-18:1_19:1	LMGP01030063
		PC O-20:1_17:1	LMGP01030083
	[^{13}C] SM 40:2	-	-
PC 36:3	PC 36:3	PC 14:1_22:2	LMGP01011406; LMGP01012038
		PC 16:0_20:3	LMGP01010622; LMGP01010624; LMGP01010627; LMGP01011872
		PC 16:1_20:2	LMGP01011490; LMGP01011842
		PC 17:2_19:1	LMGP01011568; LMGP01011766
		PC 18:0_18:3	LMGP01011588; LMGP01011589; LMGP01011651; LMGP01011682
		PC 18:1_18:2	LMGP01010893; LMGP01010895; LMGP01011624; LMGP01012149
	PC O-37:3	PC O-20:1_17:2	LMGP01030084
	[^{13}C] SM 40:3	-	-

MxP® Quant 500 XL kit lipid annotation	Potential isobars within ± 0.5 Da	Potential isomers	Data base ID
PC 36:4	PC 36:4	PC 14:0_22:4	LMGP01011378; LMGP01012068
		PC 16:0_20:4	LMGP01010007; LMGP01010629; LMGP01011049; LMGP01011056
		PC 16:1_20:3	LMGP01011491; LMGP01011873; LMGP01012138; LMGP01012195
		PC 18:0_18:4	LMGP01010773; LMGP01010774; LMGP01011711
		PC 18:1_18:3	LMGP01010898; LMGP01011603; LMGP01011652; LMGP01012150; LMGP01012174; LMGP01012178
		PC 18:2_18:2	LMGP01010921; LMGP01010924; LMGP01010927; LMGP01010930
	PC O-37:4	PC O-17:0_20:4	LMGP01020081
PC 36:5	PC 36:5	PC 14:0_22:5	LMGP01012130; LMGP01012131; LMGP01012232; LMGP01012234
		PC 14:1_22:4	LMGP01011407; LMGP01012069
		PC 16:0_20:5	LMGP01010633; LMGP01011932
		PC 16:1_20:4	LMGP01010695; LMGP01011305; LMGP01011903; LMGP01012139
		PC 18:1_18:4	LMGP01011604; LMGP01011712; LMGP01012152; LMGP01012182
		PC 18:2_18:3	LMGP01011625; LMGP01011626; LMGP01011653; LMGP01011683
PC 36:6	PC 36:6	PC 14:0_22:6	LMGP01010512; LMGP01012099
		PC 14:1_22:5	LMGP01012134; LMGP01012135; LMGP01012233; LMGP01012235
		PC 16:1_20:5	LMGP01011492; LMGP01011933
		PC 18:3_18:3	LMGP01010956; LMGP01010954; LMGP01011654; LMGP01011684
		PC 18:4_18:2	LMGP01010960; LMGP01011627; LMGP01011713

MxP® Quant 500 XL kit lipid annotation	Potential isobars within ± 0.5 Da	Potential isomers	Data base ID
PC 38:0	PC 38:0	PC 12:0_26:0	LMGP01010452
		PC 13:0_25:0	LMGP01010470
		PC 14:0_24:0	LMGP01010515
		PC 16:0_22:0	LMGP01010636; LMGP01011983
		PC 17:0_21:0	LMGP01011516; LMGP01011960
		PC 17:1_22:6	LMGP01011550; LMGP01012105
		PC 18:0_20:0	LMGP01010781; LMGP01011004
		PC 19:0_19:0	LMGP01010976
	PC O-39:0	PC O-17:0_22:0	LMGP01020083
		PC O-18:0_21:0	LMGP01020212
		PC O-20:0_19:0	LMGP01020232
	PC O-38:7	PC O-18:1_22:6	LMGP01030014
	[^{13}C] SM 42:0	-	-
PC 38:1	PC 38:1	PC 14:0_24:1	LMGP01010516
		PC 16:0_22:1	LMGP01010638; LMGP01011471; LMGP01012010
		PC 16:1_22:0	LMGP01011494; LMGP01011984
		PC 17:1_21:0	LMGP01011545; LMGP01011961
		-	-
		PC 18:0_20:1	LMGP01010783; LMGP01010784; LMGP01010785; LMGP01010786; LMGP01011816
		PC 18:1_20:0	LMGP01010879; LMGP01010902; LMGP01011008; LMGP01011009
		PC 19:0_19:1	LMGP01011743; LMGP01011773
	PC 39:8	PC 17:2_22:6	LMGP01011580; LMGP01012106
	PC O-39:1	PC O-20:0_19:1	LMGP01020233
		PC O-18:1_21:0	LMGP01030069
		PC O-20:1_19:0	LMGP01030091
PC 38:3	PC 38:3	PC 16:1_22:2	LMGP01011496; LMGP01012042
		PC 18:0_20:3	LMGP01010795; LMGP01010796; LMGP01010798; LMGP01011877; LMGP01012196
		PC 18:1_20:2	LMGP01011608; LMGP01011847; LMGP01012153; LMGP01012190
		PC 18:2_20:1	LMGP01010942; LMGP01011631; LMGP01011818
		PC 18:3_20:0	LMGP01011659; LMGP01011688; LMGP01011794; LMGP01011795
	[^{13}C] SM 42:3	-	-

MxP® Quant 500 XL kit lipid annotation	Potential isobars within ± 0.5 Da	Potential isomers	Data base ID
PC 38:4	PC 38:4	PC 16:0_22:4	LMGP01010642; LMGP01012072
		PC 18:0_20:4	LMGP01010802; LMGP01010804; LMGP01012144; LMGP01011907
		PC 18:1_20:3	LMGP01010904; LMGP01011609; LMGP01011878; LMGP01012154; LMGP01012155; LMGP01012197; LMGP01012198; LMGP01012206
		PC 18:2_20:2	LMGP01011632; LMGP01011848
		PC 18:3_20:1	LMGP01011660; LMGP01011689; LMGP01011819; LMGP01011820
		PC 18:4_20:0	LMGP01011719; LMGP01011796
PC 38:5	PC 38:5	PC 16:0_22:5	LMGP01010645; LMGP01010647
		PC 16:1_22:4	LMGP01011497; LMGP01012073
		PC 18:0_20:5	LMGP01010805; LMGP01010807; LMGP01011937
		PC 18:1_20:4	LMGP01010905; LMGP01011908; LMGP01012156; LMGP01012157; LMGP01012165; LMGP01012208; LMGP01012211
		PC 18:2_20:3	LMGP01011633; LMGP01011879; LMGP01012170; LMGP01012199
		PC 18:3_20:2	LMGP01011661; LMGP01011690; LMGP01011849; LMGP01011850
		PC 18:4_20:1	LMGP01011720; LMGP01011821

MxP® Quant 500 XL kit lipid annotation	Potential isobars within ± 0.5 Da	Potential isomers	Data base ID
PC 38:6	PC 38:6	PC 16:0_22:6	LMGP01010650; LMGP01010652; LMGP01011115; LMGP01011116; LMGP01012137
		PC 16:1_22:5	LMGP01012140; LMGP01012141
		PC 18:1_20:5	LMGP01010844; LMGP01010907; LMGP01011057; LMGP01012216
		PC 18:2_20:4	LMGP01010943; LMGP01011909; LMGP01012171; LMGP01012212
		PC 18:3_20:3	LMGP01011662; LMGP01011691; LMGP01011880; LMGP01011881; LMGP01012175; LMGP01012179; LMGP01012200; LMGP01012201
		PC 18:4_20:2	LMGP01011721; LMGP01011851
PC 40:1	PC 40:1	PC 16:0_24:1	LMGP01010659
		PC 18:0_22:1	LMGP01010811; LMGP01011593; LMGP01012015
		PC 18:1_22:0	LMGP01010839; LMGP01010845; LMGP01010909; LMGP01011089; LMGP01011090; LMGP01011091; LMGP01011092; LMGP01011093; LMGP01011094
		PC 19:1_21:0	LMGP01011781; LMGP01011969
		PC 20:0_20:1	LMGP01011017; LMGP01011018; LMGP01011019; LMGP01011020; LMGP01011824
	PC O-41:1	PC O-20:1_21:0	LMGP01030099

MxP® Quant 500 XL kit lipid annotation	Potential isobars within ± 0.5 Da	Potential isomers	Data base ID
PC 40:2	PC 40:2	PC 16:1_24:1	LMGP01012142; LMGP01012239
		PC 18:0_22:2	LMGP01011594; LMGP01012046
		PC 18:1_22:1	LMGP01011611; LMGP01012016; LMGP01012158; LMGP01012166; LMGP01012220; LMGP01012221
		PC 18:2_22:0	LMGP01011636; LMGP01011988
		PC 20:0_20:2	LMGP01011021; LMGP01011854
		PC 20:1_20:1	LMGP01011036; LMGP01011038; LMGP01011042; LMGP01011043
PC 40:3	PC 40:3	PC 18:0_22:3	LMGP01010812
		PC 20:0_20:3	LMGP01011022; LMGP01011799; LMGP01011885; LMGP01012185; LMGP01012202
		PC 18:1_22:2	LMGP01011612; LMGP01012047; LMGP01012159; LMGP01012230
		PC 18:2_22:1	LMGP01011637; LMGP01012017; LMGP01012172; LMGP01012222
		PC 18:3_22:0	LMGP01011666; LMGP01011695; LMGP01011989; LMGP01011990
		PC 20:1_20:2	LMGP01011825; LMGP01011855
PC 40:4	PC 40:4	PC 18:0_22:4	LMGP01010813; LMGP01012077
		PC 18:2_22:2	LMGP01011638; LMGP01012048
		PC 18:3_22:1	LMGP01011667; LMGP01011696; LMGP01012018; LMGP01012019; LMGP01012180; LMGP01012223; LMGP01012224
		PC 18:4_22:0	LMGP01011726; LMGP01011991
		PC 20:0_20:4	LMGP01011023; LMGP01011915; LMGP01012186; LMGP01012213
		PC 20:1_20:3	LMGP01011826; LMGP01011886; LMGP01012187; LMGP01012203
		PC 20:2_20:2	LMGP01011045; LMGP01011856

MxP® Quant 500 XL kit lipid annotation	Potential isobars within ± 0.5 Da	Potential isomers	Data base ID
PC 40:5	PC 40:5	PC 18:0_22:5	LMGP01010816; LMGP01010818
PC 40:5	PC 40:5	PC 18:1_22:4	LMGP01011613; LMGP01012078; LMGP01012160; LMGP01012231
PC 40:5	PC 40:5	PC 18:3_22:2	LMGP01011668; LMGP01011697; LMGP01012049; LMGP01012050
PC 40:5	PC 40:5	PC 18:4_22:1	LMGP01011727; LMGP01012020; LMGP01012183; LMGP01012225
PC 40:5	PC 40:5	PC 20:0_20:5	LMGP01011800
PC 40:5	PC 40:5	PC 20:1_20:4	LMGP01011827; LMGP01012188; LMGP01012214
PC 40:5	PC 40:5	PC 20:2_20:3	LMGP01011857; LMGP01012191; LMGP01012204
PC 40:6	PC 40:6	PC 18:0_22:6	LMGP01010821; LMGP01010823; LMGP01012107
PC 40:6	PC 40:6	PC 18:1_22:5	LMGP01010846
PC 40:6	PC 40:6	PC 18:2_22:4	LMGP01011639; LMGP01012079
PC 40:6	PC 40:6	PC 18:4_22:2	LMGP01011728; LMGP01012051
PC 40:6	PC 40:6	PC 20:1_20:5	LMGP01011828; LMGP01011945
PC 40:6	PC 40:6	PC 20:2_20:4	LMGP01011858; LMGP01011917
PC 40:6	PC 40:6	PC 20:3_20:3	LMGP01011888
PC 42:0	PC 42:0	PC 16:0_26:0	LMGP01010663
PC 42:0	PC 42:0	PC 18:0_24:0	LMGP01010825; LMGP01011150
PC 42:0	PC 42:0	PC 20:0_22:0	LMGP01011025; LMGP01011994
PC 42:0	PC 42:0	PC 21:0_21:0	LMGP01011072
PC 42:1	PC 42:1	PC 18:0_24:1	LMGP01010826; LMGP01012240
PC 42:1	PC 42:1	PC 18:1_24:0	LMGP01010915; LMGP01012236; LMGP01011151; LMGP01011152
PC 42:1	PC 42:1	PC 20:0_22:1	LMGP01011026; LMGP01012023; LMGP01012226
PC 42:1	PC 42:1	PC 20:1_22:0	LMGP01011830; LMGP01011040; LMGP01011041; LMGP01011097

MxP® Quant 500 XL kit lipid annotation	Potential isobars within ± 0.5 Da	Potential isomers	Data base ID
PC 42:2	PC 42:2	PC 16:0_26:2	LMGP01010665; LMGP01011203
		PC 18:1_24:1	LMGP01012162; LMGP01012167; LMGP01012241; LMGP01012242
		PC 18:2_24:0	LMGP01012173; LMGP01012237
		PC 20:0_22:2	LMGP01011803; LMGP01012054
		PC 20:1_22:1	LMGP01011831; LMGP01012189; LMGP01012024; LMGP01012227
		PC 20:2_22:0	LMGP01011861; LMGP01011995
PC 42:4	PC 42:4	PC 18:3_24:1	LMGP01012177; LMGP01012181; LMGP01012243; LMGP01012244
		PC 18:4_24:0	LMGP01012184; LMGP01012238
		PC 20:0_22:4	LMGP01011804; LMGP01012085
		PC 20:2_22:2	LMGP01011863; LMGP01012056
		PC 20:3_22:1	LMGP01011893; LMGP01012205; LMGP01012207; LMGP01012026; LMGP01012228; LMGP01012229
		PC 20:4_22:0	LMGP01011921; LMGP01012215; LMGP01011997; LMGP01012217
PC 42:5	PC 42:5	PC 20:0_22:5	LMGP01011027
		PC 20:1_22:4	LMGP01011833; LMGP01012086
		PC 20:3_22:2	LMGP01011894; LMGP01012057
		PC 20:4_22:1	LMGP01011922; LMGP01012027
		PC 20:5_22:0	LMGP01011951; LMGP01011998
PC 42:6	PC 42:6	PC 20:0_22:6	LMGP01011028; LMGP01012115
		PC 20:2_22:4	LMGP01011864; LMGP01012087
		PC 20:4_22:2	LMGP01011923; LMGP01012058
		PC 20:5_22:1	LMGP01011952; LMGP01012028

MxP® Quant 500 XL kit lipid annotation	Potential isobars within ± 0.5 Da	Potential isomers	Data base ID
PC O-28:0	PC O-28:0	PC(O-16:0/12:0)	LMGP01020176
	PC 27:0	PC(17:0/10:0)	LMGP01010700
		PC(8:0/19:0)	LMGP01011244
		PC(9:0/18:0)	LMGP01011262
		PC(12:0/15:0); PC(15:0/12:0)	LMGP01011317; LMGP01011409
		PC(13:0/14:0); PC(14:0/13:0)	LMGP01011339; LMGP01011363
		PC(11:0/16:0)	LMGP01012251
	PKODiA-PC	PKODiA-PC	LMGP20010013
PC O-28:1	PC O-28:1	PC(P-16:0/12:0)	LMGP01030019
	PC 27:1	PC(12:0/15:1(9Z)); PC(15:1(9Z)/12:0)	LMGP01011318; LMGP01011431
		PC(13:0/14:1(9Z)); PC(14:1(9Z)/13:0)	LMGP01011340; LMGP01011380
	OHOHA-PC	OHOHA-PC	LMGP20010033
PC O-30:0	PC O-30:0	PC O-14:0_16:0	LMGP01020012; LMGP01020178
		PC O-18:0_12:0	LMGP01020193
	PC 29:0	PC 10:0_19:0	LMGP01010394; LMGP01010968
		PC 12:0_17:0	LMGP01011320; LMGP01011498
		PC 13:0_16:0	LMGP01011342; LMGP01011462
		PC 14:0_15:0	LMGP01010479; LMGP01011410
		PC 20:0_9:0	LMGP01011035; LMGP01011264
		PC 8:0_21:0	LMGP01011246
	[^{13}C] SM 33:0	-	-
PC O-30:1	PC O-30:1	PC O-14:0_16:1	LMGP01020014; LMGP01030021
		PC O-16:0_14:1	LMGP01020179
		PC O-18:1_12:0	LMGP01030046
	PC 29:1	PC 12:0_17:1	LMGP01011321; LMGP01011521
		PC 13:0_16:1	LMGP01011343; LMGP01011474
		PC 14:0_15:1	LMGP01011365; LMGP01011433
		PC 14:1_15:0	LMGP01011382; LMGP01011411
		PC 18:0_11:1	LMGP01010735
PC O-30:2	PC O-30:2	PC O-16:1_14:1	LMGP01030022
	PC 29:2	PC 12:0_17:2	LMGP01011322; LMGP01011551
		PC 14:1_15:1	LMGP01011383; LMGP01011434
	[^{13}C] SM 33:2	-	-

biocrates
The future of research and health

MxP® Quant 500 XL kit lipid annotation	Potential isobars within ± 0.5 Da	Potential isomers	Data base ID
PC O-32:1	PC O-32:1	PC O-14:0_18:1	LMGP01020016; LMGP01030048; LMGP01090001
		PC O-16:0_16:1	LMGP01020182; LMGP01030025
		PC O-18:0_14:1	LMGP01020196
		PC O-20:1_12:0	LMGP01030074
	PC 31:1	PC 12:0_19:1	LMGP01011328; LMGP01011756
		PC 13:0_18:1	LMGP01011347; LMGP01011596
		PC 14:0_17:1	LMGP01011367; LMGP01011523
		PC 15:0_16:1	LMGP01010535; LMGP01011477
		PC 16:0_15:1	LMGP01010002; LMGP01011437; LMGP01011464
		PC 17:0_14:1	LMGP01010008
PC O-32:2	PC O-32:2	PC O-14:0_18:2	LMGP01020017; LMGP01090002; LMGP01090003; LMGP01030133
		PC O-14:1_18:1	LMGP01030004; LMGP01090004; LMGP01030049
		PC O-16:1_16:1	LMGP01030026; LMGP01090020
	PC 31:2	PC 13:0_18:2	LMGP01011348; LMGP01011615
		PC 14:0_17:2	LMGP01011368; LMGP01011553
		PC 14:1_17:1	LMGP01011387; LMGP01011524
		PC 15:1_16:1	LMGP01011438; LMGP01011478
PC O-34:0	PC O-34:0	PC O-16:0_18:0	LMGP01020033; LMGP01020086; LMGP01080024
		PC O-17:0_17:0	LMGP01020076
		PC O-20:0_14:0	LMGP01020218
	PC 33:0	PC 10:0_23:0	LMGP01010399
		PC 11:0_22:0	LMGP01010419
		PC 13:0_20:0	LMGP01010465
		PC 15:0_18:0	LMGP01010539; LMGP01011583
		PC 16:0_17:0	LMGP01010569; LMGP01011501
		PC 19:0_14:0	LMGP01010971
		PC 21:0_12:0	LMGP01011063

MxP® Quant 500 XL kit lipid annotation	Potential isobars within ± 0.5 Da	Potential isomers	Data base ID
PC O-34:1	PC O-34:1	PC O-16:0_18:1	LMGP01020003; LMGP01020261; LMGP01020152; LMGP01030052
		PC O-18:0_16:1	LMGP01020089; LMGP01020256; LMGP01030030
		PC O-20:0_14:1	LMGP01020219
		PC O-20:1_14:0	LMGP01030076
	PC 33:1	PC 13:0_20:1	LMGP01011353; LMGP01011806
		PC 14:0_19:1	LMGP01011371; LMGP01011758
		PC 14:1_19:0	LMGP01011395; LMGP01011731
		PC 15:0_18:1	LMGP01010541; LMGP01011415; LMGP01011598
		PC 15:1_18:0	LMGP01011442; LMGP01011584
		PC 16:0_17:1	LMGP01010571; LMGP01011527
		PC 16:1_17:0	LMGP01011480; LMGP01011502
	[13C] SM 37:1	-	-
PC O-34:2	PC O-34:2	PC O-16:0_18:2	LMGP01020039; LMGP01030134; LMGP01090006; LMGP01090007
		PC O-16:1_18:1	LMGP01030006; LMGP01030053; LMGP01090009
		PC O-20:1_14:1	LMGP01030077
	PC 33:2	PC 13:0_20:2	LMGP01011354
		PC 14:1_19:1	LMGP01011396
		PC 15:0_18:2	LMGP01010543
		PC 15:1_18:1	LMGP01011443
		PC 16:0_17:2	LMGP01011465; LMGP01011557
		PC 16:1_17:1	LMGP01011481; LMGP01011528
PC O-34:3	PC O-34:3	PC O-16:0_18:3	LMGP01020041; LMGP01020042
		PC O-16:1_18:2	LMGP01020257; LMGP01030008; LMGP01090010; LMGP01030135; LMGP01090011; LMGP01030145
	PC 33:3	PC 13:0_20:3	LMGP01011355; LMGP01011867
		PC 15:0_18:3	LMGP01011416; LMGP01011417; LMGP01011644; LMGP01011675
		PC 15:1_18:2	LMGP01011444; LMGP01011619
		PC 16:1_17:2	LMGP01011482; LMGP01011558

MxP® Quant 500 XL kit lipid annotation	Potential isobars within ± 0.5 Da	Potential isomers	Data base ID
PC O-36:0	PC O-36:0	PC O-14:0_22:0	LMGP01020023
		PC O-16:0_20:0	LMGP01020051; LMGP01020122
		PC O-18:0_18:0	LMGP01020091
	PC 35:0	PC 10:0_25:0	LMGP01010401
		PC 11:0_24:0	LMGP01010421
		PC 12:0_23:0	LMGP01010448
		PC 15:0_20:0	LMGP01011420; LMGP01011788
		PC 16:0_19:0	LMGP01011466; LMGP01011733
		PC 17:0_18:0	LMGP01010709; LMGP01011585
		PC 21:0_14:0	LMGP01011065; LMGP01011375
		PC 22:0_13:0	LMGP01011084; LMGP01011358
	PC 36:7	PC 14:1_22:6	LMGP01011408; LMGP01012100
		PC 18:3_18:4	LMGP01011656; LMGP01011685; LMGP01011714; LMGP01011715
PC O-36:1	PC O-36:1	PC O-16:0_20:1	LMGP01020052; LMGP01030080; LMGP01020187
		PC O-18:0_18:1	LMGP01020202; LMGP01030057
		PC O-20:0_16:1	LMGP01020222
		PC O-16:1_20:0	LMGP01020258; LMGP01030036
	PC 35:1	PC 13:0_22:1	LMGP01011359; LMGP01012005
		PC 14:1_21:0	LMGP01011403; LMGP01011955
		PC 15:0_20:1	LMGP01011421; LMGP01011809
		PC 15:1_20:0	LMGP01011450; LMGP01011789
		PC 16:0_19:1	LMGP01011467; LMGP01011762
		PC 16:1_19:0	LMGP01011486; LMGP01011734
		PC 17:0_18:1	LMGP01010711; LMGP01011600
		PC 17:1_18:0	LMGP01011531; LMGP01011586
	PC 36:8	PC 18:4_18:4	LMGP01010961; LMGP01011716; LMGP01010964
	[^{13}C] SM 39:1	-	-

MxP® Quant 500 XL kit lipid annotation	Potential isobars within ± 0.5 Da	Potential isomers	Data base ID
PC O-36:2	PC O-36:2	PC O-16:0_20:2	LMGP01020188
		PC O-18:0_18:2	LMGP01020203; LMGP01030136; LMGP01030146
		PC O-18:1_18:1	LMGP01020263; LMGP01030013; LMGP01030130; LMGP01090013
		PC O-16:1_20:1	LMGP01030037; LMGP01090036; LMGP01030081
	PC 35:2	PC 13:0_22:2	LMGP01011360; LMGP01012036
		PC 15:0_20:2	LMGP01011422; LMGP01011839
		PC 15:1_20:1	LMGP01011451; LMGP01011810
		PC 16:1_19:1	LMGP01011487; LMGP01011763
		PC 17:0_18:2	LMGP01011505; LMGP01011621
		PC 17:1_18:1	LMGP01011532; LMGP01011601
		PC 17:2_18:0	LMGP01011561; LMGP01011587
	[^{13}C] SM 39:2	-	-
PC O-36:3	PC O-36:3	PC O-16:0_20:3	LMGP01020053
		PC O-18:0_18:3	LMGP01020204; LMGP01020205
		PC O-18:1_18:2	LMGP01020262; LMGP01090014; LMGP01090015; LMGP01090017; LMGP01090016; LMGP01030137; LMGP01020264; LMGP01030058
		PC O-16:1_20:2	LMGP01030038; LMGP01090037
	PC 35:3	PC 15:0_20:3	LMGP01011423; LMGP01011870
		PC 15:1_20:2	LMGP01011452; LMGP01011840
		PC 17:0_18:3	LMGP01011506; LMGP01011507; LMGP01011648; LMGP01011679
		PC 17:1_18:2	LMGP01011533; LMGP01011622
		PC 17:2_18:1	LMGP01011562; LMGP01011602

MxP® Quant 500 XL kit lipid annotation	Potential isobars within ± 0.5 Da	Potential isomers	Data base ID
PC O-36:4	PC O-36:4	PC O-16:0_20:4	LMGP01020054; LMGP01020056; LMGP01020245
		PC O-16:1_20:3	LMGP01030039; LMGP01090040; LMGP01090044; LMGP01030129
		PC O-18:0_18:4	LMGP01020206
		PC O-18:1_18:3	LMGP01030059; LMGP01090026; LMGP01090028; LMGP01030060
		PC O-18:2_18:2	LMGP01020270; LMGP01090022; LMGP01090023
	PC 35:4	PC 13:0_22:4	LMGP01011361; LMGP01012067
		PC 15:0_20:4	LMGP01011424; LMGP01011901
		PC 15:1_20:3	LMGP01011453; LMGP01011871
		PC 17:0_18:4	LMGP01011508; LMGP01011708
		PC 17:1_18:3	LMGP01011534; LMGP01011535; LMGP01011649; LMGP01011680
		PC 17:2_18:2	LMGP01011563; LMGP01010931; LMGP01011623
PC O-36:5	PC O-36:5	PC O-16:0_20:5	LMGP01020058
		PC O-16:1_20:4	LMGP01020259; LMGP01030010
		PC O-18:1_18:4	LMGP01030061; LMGP01090032
		PC O-18:2_18:3	LMGP01090024; LMGP01090025; LMGP01090030; LMGP01090031
	PC 35:5	PC 15:0_20:5	LMGP01011425; LMGP01011930
		PC 15:1_20:4	LMGP01011454; LMGP01011902
		PC 17:1_18:4	LMGP01011536; LMGP01011709
		PC 17:2_18:3	LMGP01011564; LMGP01011565; LMGP01011650; LMGP01011681

MxP® Quant 500 XL kit lipid annotation	Potential isobars within ± 0.5 Da	Potential isomers	Data base ID
PC O-38:0	PC O-38:0	PC O-16:0_22:0	LMGP01020060
		PC O-18:0_20:0	LMGP01020097; LMGP01020226
	PC 37:0	PC 15:0_22:0	LMGP01011426; LMGP01011981
		PC 16:0_21:0	LMGP01011470; LMGP01011958
		PC 17:0_20:0	LMGP01011511; LMGP01011791
		PC 18:0_19:0	LMGP01011590; LMGP01010975
	PC 38:7	PC 16:1_22:6	LMGP01010696; LMGP01011306; LMGP01012103
		PC 18:2_20:5	LMGP01011634; LMGP01011938
		PC 18:3_20:4	LMGP01011663; LMGP01011692; LMGP01011910; LMGP01011911
		PC 18:4_20:3	LMGP01011722; LMGP01011882
PC O-38:1	PC O-38:1	PC O-16:0_22:1	LMGP01020190
		PC O-16:1_22:0	LMGP01020260; LMGP01030042
		PC O-18:0_20:1	LMGP01020099; LMGP01020209; LMGP01030085
		PC O-18:1_20:0	LMGP01020265; LMGP01030064; LMGP01020227
	PC 37:1	PC 15:0_22:1	LMGP01011427; LMGP01012008
		PC 15:1_22:0	LMGP01011457; LMGP01011982
		PC 16:1_21:0	LMGP01011493; LMGP01011959
		PC 17:0_20:1	LMGP01011512; LMGP01011813
		PC 17:1_20:0	LMGP01011539; LMGP01011792
		PC 18:0_19:1	LMGP01011591; LMGP01011767
		PC 18:1_19:0	LMGP01011605; LMGP01011738
	PC 38:8	PC 18:3_20:5	LMGP01011664; LMGP01011693; LMGP01011939; LMGP01011940
		PC 18:4_20:4	LMGP01011723; LMGP01011912

MxP® Quant 500 XL kit lipid annotation	Potential isobars within ± 0.5 Da	Potential isomers	Data base ID
PC O-38:2	PC O-38:2	PC O-16:0_22:2	LMGP01020191
		PC O-18:0_20:2	LMGP01020210
		PC O-18:1_20:1	LMGP01020266; LMGP01030065; LMGP01030086
		PC O-18:2_20:0	LMGP01020271; LMGP01020228
		PC O-16:1_22:1	LMGP01030043
	PC 37:2	PC 15:0_22:2	LMGP01011428; LMGP01012039
		PC 15:1_22:1	LMGP01011458; LMGP01012009
		PC 17:0_20:2	LMGP01011513; LMGP01011843
		PC 17:1_20:1	LMGP01011540; LMGP01011814
		PC 17:2_20:0	LMGP01011569; LMGP01011793
		PC 18:1_19:1	LMGP01011606; LMGP01011768
		PC 18:2_19:0	LMGP01011628; LMGP01011739
	PC 38:9	PC 18:4_20:5	LMGP01011724; LMGP01011941
PC O-38:3	PC O-38:3	PC O-18:0_20:3	LMGP01020211
		PC O-16:1_22:2	LMGP01030044
		PC O-18:1_20:2	LMGP01030066
		PC O-18:2_20:1	LMGP01030087
		P O-18:3_20:0	LMGP01020229; LMGP01020230
	PC 37:3	PC 15:1_22:2	LMGP01011459; LMGP01012040
		PC 17:0_20:3	LMGP01011514; LMGP01011874
		PC 17:1_20:2	LMGP01011541; LMGP01011844
		PC 17:2_20:1	LMGP01011570; LMGP01011815
		PC 18:2_19:1	LMGP01011629; LMGP01011769
		PC 18:3_19:0	LMGP01011657; LMGP01011686; LMGP01011740; LMGP01011741

MxP® Quant 500 XL kit lipid annotation	Potential isobars within ± 0.5 Da	Potential isomers	Data base ID
PC O-38:4	PC O-38:4	PC O-16:0_22:4	LMGP01020192
		PC O-18:0_20:4	LMGP01020100; LMGP01020102; LMGP01020247
		PC O-20:0_18:4	LMGP01020231
		PC O-18:1_20:3	LMGP01030067; LMGP01090041; LMGP01090045; LMGP01030131
		PC O-18:2_20:2	LMGP01030138; LMGP01090038; LMGP01090039
		PC O-20:1_18:3	LMGP01030088; LMGP01030089
	PC 37:4	PC 15:0_22:4	LMGP01011429; LMGP01012070
		PC 17:0_20:4	LMGP01010003; LMGP01011904
		PC 17:1_20:3	LMGP01011542; LMGP01011875
		PC 17:2_20:2	LMGP01011571; LMGP01011845
		PC 18:3_19:1	LMGP01011658; LMGP01011770; LMGP01011771
		PC 18:4_19:0	LMGP01011717; LMGP01011742
PC O-38:5	PC O-38:5	PC O-16:0_22:5	LMGP01020066
		PC O-16:1_22:4	LMGP01030045
		PC O-18:0_20:5	LMGP01020104
		PC O-18:1_20:4	LMGP01020157; LMGP01020267; LMGP01030012
		PC O-18:2_20:3	LMGP01090042; LMGP01090043; LMGP01090046; LMGP01090047
		PC O-18:4_20:1	LMGP01030090
	PC 37:5	PC 15:1_22:4	LMGP01011460; LMGP01012071
		PC 17:0_20:5	LMGP01011515; LMGP01011934
		PC 17:1_20:4	LMGP01011543; LMGP01011905
		PC 17:2_20:3	LMGP01011572; LMGP01011876
		PC 18:4_19:1	LMGP01011718; LMGP01011772
PC O-38:6	PC O-38:6	PC O-16:0_22:6	LMGP01020064
		PC O-18:1_20:5	LMGP01030068
		PC O-18:2_20:5	LMGP01090048; LMGP01090049
	PC 37:6	PC 15:0_22:6	LMGP01011430; LMGP01012101
		PC 17:1_20:5	LMGP01011544; LMGP01011935
		PC 17:2_20:4	LMGP01011573; LMGP01011906

MxP® Quant 500 XL kit lipid annotation	Potential isobars within ± 0.5 Da	Potential isomers	Data base ID
PC O-40:1	PC O-40:1	PC O-18:0_22:1	LMGP01020213
		PC O-18:1_22:0	LMGP01020268; LMGP01030070
		PC O-20:0_20:1	LMGP01020235; LMGP01030093
	PC 39:1	PC 17:0_22:1	LMGP01011518; LMGP01012012
		PC 17:1_22:0	LMGP01011546; LMGP01011986
		PC 18:1_21:0	LMGP01011610; LMGP01011963
		PC 19:0_20:1	LMGP01011745; LMGP01011822
		PC 19:1_20:0	LMGP01011775; LMGP01011798
	PC 40:8	PC 18:2_22:6	LMGP01010947; LMGP01012109
		PC 18:4_22:4	LMGP01011729; LMGP01012082
		PC 20:3_20:5	LMGP01011890; LMGP01011947
		PC 20:4_20:4	LMGP01011047; LMGP01011052
PC O-40:2	PC O-40:2	PC O-18:0_22:2	LMGP01020214
		PC O-18:1_22:1	LMGP01030071
		PC O-18:2_22:0	LMGP01020272
		PC O-20:0_20:2	LMGP01020236
		PC O-20:1_20:1	LMGP01030094
	PC 39:2	PC 17:0_22:2	LMGP01011519; LMGP01012043
		PC 17:1_22:1	LMGP01011547; LMGP01012013
		PC 17:2_22:0	LMGP01011576; LMGP01011987
		PC 18:2_21:0	LMGP01011635; LMGP01011964
		PC 19:0_20:2	LMGP01011746; LMGP01011852
		PC 19:1_20:1	LMGP01011776; LMGP01011823
	PC 40:9	PC 18:3_22:6	LMGP01011670; LMGP01011699; LMGP01012110; LMGP01012111
		PC 20:4_20:5	LMGP01011919; LMGP01011948
	[^{13}C] SM 43:2	-	-

MxP® Quant 500 XL kit lipid annotation	Potential isobars within ± 0.5 Da	Potential isomers	Data base ID
PC O-40:3	PC O-40:3	PC O-18:1_22:2	LMGP01030072; LMGP01090055
PC O-40:3	PC O-40:3	PC O-18:2_22:1	LMGP01030139; LMGP01090053; LMGP01090054; LMGP01030147
PC O-40:3	PC O-40:3	PC O-20:0_20:3	LMGP01020237
PC O-40:3	PC O-40:3	PC O-20:1_20:2	LMGP01030095
PC O-40:3	PC O-40:3	PC O-22:0_18:3	LMGP01020249
PC O-40:3	PC 39:3	PC 17:1_22:2	LMGP01011548; LMGP01012044
PC O-40:3	PC 39:3	PC 17:2_22:1	LMGP01011577; LMGP01012014
PC O-40:3	PC 39:3	PC 18:3_21:0	LMGP01011665; LMGP01011694; LMGP01011965; LMGP01011966
PC O-40:3	PC 39:3	PC 19:0_20:3	LMGP01011747; LMGP01011883
PC O-40:3	PC 39:3	PC 19:1_20:2	LMGP01011777; LMGP01011853
PC O-40:3	PC 40:10	PC 18:4_22:6	LMGP01011730; LMGP01012112
PC O-40:3	PC 40:10	PC 20:5_20:5	LMGP01011949
PC O-40:4	PC O-40:4	PC O-18:0_22:4	LMGP01020215
PC O-40:4	PC O-40:4	PC O-18:2_22:2	LMGP01030140; LMGP01090056; LMGP01090057; LMGP01030148
PC O-40:4	PC O-40:4	PC O-20:0_20:4	LMGP01020238; LMGP01020248
PC O-40:4	PC O-40:4	PC O-20:1_20:3	LMGP01030096
PC O-40:4	PC 39:4	PC 17:0_22:4	LMGP01011520; LMGP01012074
PC O-40:4	PC 39:4	PC 17:2_22:2	LMGP01011578; LMGP01012045
PC O-40:4	PC 39:4	PC 18:4_21:0	LMGP01011725; LMGP01011967
PC O-40:4	PC 39:4	PC 19:0_20:4	LMGP01011748; LMGP01011913
PC O-40:4	PC 39:4	PC 19:1_20:3	LMGP01011778; LMGP01011884
PC O-40:5	PC O-40:5	PC O-18:0_22:5	LMGP01020107; LMGP01020109
PC O-40:5	PC O-40:5	PC O-18:1_22:4	LMGP01030073; LMGP01090058
PC O-40:5	PC O-40:5	PC O-20:0_20:5	LMGP01020239
PC O-40:5	PC O-40:5	PC O-20:1_20:4	LMGP01020274; LMGP01030097
PC O-40:5	PC 39:5	PC 16:0_23:5	LMGP01010656
PC O-40:5	PC 39:5	PC 17:0_22:5	LMGP01010719
PC O-40:5	PC 39:5	PC 17:1_22:4	LMGP01011549; LMGP01012075
PC O-40:5	PC 39:5	PC 19:0_20:5	LMGP01011749; LMGP01011942
PC O-40:5	PC 39:5	PC 19:1_20:4	LMGP01011779; LMGP01011914

MxP® Quant 500 XL kit lipid annotation	Potential isobars within ± 0.5 Da	Potential isomers	Data base ID
PC O-40:6	PC O-40:6	PC O-18:0_22:6	LMGP01020110
		PC O-18:2_22:4	LMGP01090059
		PC O-20:1_20:5	LMGP01030098
	PC 39:6	PC 17:0_22:6	LMGP01010720; LMGP01012104
		PC 17:2_22:4	LMGP01011579; LMGP01012076
		PC 19:1_20:5	LMGP01011780; LMGP01011943
PC O-42:0	PC O-42:0	PC O-20:0_22:0	LMGP01020125
	PC 41:0	PC 19:0_22:0	LMGP01011751; LMGP01011992
		PC 20:0_21:0	LMGP01011801; LMGP01011970
		PC 23:0_18:0	LMGP01011130
	PC 42:7	PC 20:1_22:6	LMGP01011834; LMGP01012116
		PC 20:3_22:4	LMGP01011895; LMGP01012088
		PC 20:5_22:2	LMGP01011953; LMGP01012059
PC O-42:1	PC O-42:1	PC O-20:0_22:1	LMGP01020241
		PC O-18:1_24:0	LMGP01020269
		PC O-20:1_22:0	LMGP01030100; LMGP01020250
	PC 40:1	PC 19:0_22:1	LMGP01011752; LMGP01012021
		PC 19:1_22:0	LMGP01011782; LMGP01011993
		PC 20:1_21:0	LMGP01011829; LMGP01011971
	PC 42:8	PC 20:2_22:6	LMGP01011865; LMGP01012117
		PC 20:4_22:4	LMGP01011924; LMGP01012089
PC O-42:2	PC O-42:2	PC O-20:0_22:2	LMGP01020242
		PC O-18:2_24:0	LMGP01020273; LMGP01030141; LMGP01030149; LMGP01090060; LMGP01090061
		PC O-20:1_22:1	LMGP01030101
		PC O-18:1_24:1	LMGP01030132; LMGP01090062
	PC 41:2	PC 19:0_22:2	LMGP01011753; LMGP01012052
		PC 19:1_22:1	LMGP01011783; LMGP01012022
		PC 20:2_21:0	LMGP01011860; LMGP01011972
	PC 42:9	PC 20:3_22:6	LMGP01011896; LMGP01012118
		PC 20:5_22:4	LMGP01011954; LMGP01012090

MxP® Quant 500 XL kit lipid annotation	Potential isobars within ± 0.5 Da	Potential isomers	Data base ID
PC O-42:3	PC O-42:3	PC O-24:0_18:3	LMGP01020252; LMGP01020253
		PC O-20:1_22:2	LMGP01030102
		PC O- 18:2_24:1	LMGP01030142; LMGP01030150; LMGP01090063; LMGP01090064
	PC 41:3	PC 21:0_20:3	LMGP01011973; LMGP01011891
		PC 19:1_22:2	LMGP01011784; LMGP01012053
		PC 20:3_21:0	LMGP01011891; LMGP01011973
	PC 42:10	PC 20:5_22:5	LMGP01011058
		PC 20:4_22:6	LMGP01011925; LMGP01012119
PC O-42:4	PC O-42:4	PC O-20:0_22:4	LMGP01020243
	PC 41:4	PC 19:0_22:4	LMGP01011754; LMGP01012083
		PC 20:4_21:0	LMGP01011920; LMGP01011974
	PC 42:11	PC 20:5_22:6	LMGP01011059; LMGP01012120
PC O-42:5	PC O-42:5	PC O-20:1_22:4	LMGP01030103
	PC 41:5	PC 19:1_22:4	LMGP01011785; LMGP01012084
		PC 20:5_21:0	LMGP01011950; LMGP01011975
PC O-44:3	PC O-44:3	PC O-22:0_22:3	LMGP01020251
		PC O-22:1_22:2	LMGP01020275
	PC 44:10	PC 22:4_22:6	LMGP01012096; LMGP01012125
PC O-44:4	PC O-44:4	PC O-24:0_20:4	LMGP01020254
		PC O-22:1_22:3	LMGP01020276
		PC O-22:2_22:2	LMGP01020277
	PC 43:4	PC 21:0_22:4	LMGP01011979; LMGP01012091
PC O-44:5	PC O-44:5	PC O-22:2_22:3	LMGP01020278
	PC 44:12	PC 22:6_22:6	LMGP01011119
PC O-44:6	PC O-44:6	PC O-22:2_22:4	-
	PC 43:6	PC 21:0_22:6	LMGP01010004; LMGP01012121

Lysophosphatidylethanolamines (43)			
MxP® Quant 500 XL kit lipid annotation	Potential isobars within ± 0.5 Da	Potential isomers	Data base ID
LPE 12:0	LPE 12:0	PE(12:0/0:0)	LMGP02050005
LPE 14:0	LPE 14:0	PE(14:0/0:0)	LMGP02050003
		PE(0:0/14:0)	LMGP02050033
LPE 14:1	LPE 14:1	PE(14:1(9Z)/0:0)	LMGP02050032
		PE(0:0/14:1(9Z))	LMGP02050034

MxP® Quant 500 XL kit lipid annotation	Potential isobars within ± 0.5 Da	Potential isomers	Data base ID
LPE 15:0	LPE 15:0	PE(15:0/0:0)	LMGP02050031
		PE(0:0/15:0)	LMGP02050035
		PE(14:0(13Me)/0:0)	LMGP02050074
	LPE O-16:0	PE(O-16:0/0:0)	LMGP02060002
	LPE O-15:1;O	1-(2-methoxy-6Z-tetradecenyl)-sn-glycero-3-phosphoethanolamine	LMGP02060008
LPE 16:0	LPE 16:0	PE(16:0/0:0)	LMGP02050002
		PE(0:0/16:0)	LMGP02050036
LPE 16:1	LPE 16:1	PE(16:1(9Z)/0:0)	LMGP02050010
		PE(0:0/16:1(9Z))	LMGP02050037
LPE 17:0	PE 16:0	PE(8:0/8:0)	LMGP02010102
	LPE 17:0	PE(17:0/0:0)	LMGP02050030
	LPE O-18:0	PE(O-18:0/0:0)	LMGP02060003
LPE 17:1	LPE 17:1	PE(17:1(9Z)/0:0)	LMGP02050008
	LPE O-18:1	PE(O-18:1(9Z)/0:0)	LMGP02060004
		PE(P-18:0/0:0)	LMGP02070002
LPE 18:0	LPE 18:0	PE(18:0/0:0)	LMGP02050001
		PE(0:0/18:0)	LMGP02050038
LPE 18:1	LPE 18:1	PE(18:1(9Z)/0:0)	LMGP02050004
		PE(0:0/18:1(11Z))	LMGP02050039
		PE(0:0/18:1(9Z))	LMGP02050040
		PE(18:1(11Z)/0:0)	LMGP02050064
LPE 18:2	LPE 18:2	PE(18:2(9Z,12Z)/0:0)	LMGP02050011
		PE(0:0/18:2(9Z,12Z))	LMGP02050041
	LPE O-19:2	PE(P-19:1(12Z)/0:0)	LMGP02070003
LPE 18:3	LPE 18:3	PE(18:3(6Z,9Z,12Z)/0:0)	LMGP02050017
		PE(18:3(9Z,12Z,15Z)/0:0)	LMGP02050029
		PE(0:0/18:3(6Z,9Z,12Z))	LMGP02050042
		PE(0:0/18:3(9Z,12Z,15Z))	LMGP02050043
LPE 19:0	LPE 19:0	PE(19:0/0:0)	LMGP02050028
	LPE O-20:0	PE(O-20:0/0:0)	LMGP02060005
LPE 19:1	LPE 19:1	PE(19:1(9Z)/0:0)	LMGP02050019
	LPE O-20:1	PE(P-20:0/0:0)	LMGP02070004
LPE 19:2	LPE 19:2	LPE 19:2	PubChem:138300243
LPE 20:0	LPE 20:0	PE(20:0/0:0)	LMGP02050012
		PE(0:0/20:0)	LMGP02050045
LPE 20:1	LPE 20:1	PE(20:1(11Z)/0:0)	LMGP02050020
		PE(0:0/20:1(11Z))	LMGP02050046
LPE 20:2	LPE 20:2	PE(20:2(11Z,14Z)/0:0)	LMGP02050021
		PE(0:0/20:2(11Z,14Z))	LMGP02050047
LPE 20:3	LPE 20:3	PE(20:3(8Z,11Z,14Z)/0:0)	LMGP02050022
		PE(0:0/20:3(11Z,14Z,17Z))	LMGP02050048
		PE(0:0/20:3(5Z,8Z,11Z))	LMGP02050049
		PE(0:0/20:3(8Z,11Z,14Z))	LMGP02050050
		PE(20:3(11Z,14Z,17Z)/0:0)	LMGP02050065
		PE(20:3(5Z,8Z,11Z)/0:0)	LMGP02050066
LPE 20:4	LPE 20:4	PE(20:4(5Z,8Z,11Z,14Z)/0:0)	LMGP02050009
		PE(0:0/20:4(5Z,8Z,11Z,14Z))	LMGP02050051
		PE(0:0/20:4(8Z,11Z,14Z,17Z))	LMGP02050052
		PE(20:4(8Z,11Z,14Z,17Z)/0:0)	LMGP02050067
LPE 20:5	LPE 20:5	PE(20:5(5Z,8Z,11Z,14Z,17Z)/0:0)	LMGP02050027
		PE(0:0/20:5(5Z,8Z,11Z,14Z,17Z))	LMGP02050053

MxP® Quant 500 XL kit lipid annotation	Potential isobars within ± 0.5 Da	Potential isomers	Data base ID
LPE 22:0	LPE 22:0	PE(22:0/0:0)	LMGP02050025
		PE(0:0/22:0)	LMGP02050054
	PE 20:1;O	POB-PE	LMGP20020032
LPE 22:1	LPE 22:1	PE(22:1(11Z)/0:0)	LMGP02050023
		PE(0:0/22:1(13Z))	LMGP02050055
		PE(22:1(13Z)/0:0)	LMGP02050068
LPE 22:4	LPE 22:4	PE(22:4(7Z,10Z,13Z,16Z)/0:0)	LMGP02050014
		PE(0:0/22:4(7Z,10Z,13Z,16Z))	LMGP02050057
LPE 22:5	LPE 22:5	PE(0:0/22:5(4Z,7Z,10Z,13Z,16Z))	LMGP02050058
		PE(0:0/22:5(7Z,10Z,13Z,16Z,19Z))	LMGP02050059
		PE(22:5(4Z,7Z,10Z,13Z,16Z)/0:0)	LMGP02050069
		PE(22:5(7Z,10Z,13Z,16Z,19Z)/0:0)	LMGP02050070
LPE 22:6	LPE 22:6	PE(22:6(4Z,7Z,10Z,13Z,16Z,19Z)/0:0)	LMGP02050013
		PE(0:0/22:6(4Z,7Z,10Z,13Z,16Z,19Z))	LMGP02050060
LPE 24:0	LPE 24:0	PE(0:0/24:0)	LMGP02050061
		PE(24:0/0:0)	LMGP02050071
LPE P-14:0	-	-	-
LPE P-15:0	-	-	-
LPE P-16:0	LPE O-16:1	PE(P-16:0/0:0)	LMGP02070001
LPE P-17:0	-	-	-
LPE P-18:0	LPE O-18:1	PE(P-18:0/0:0)	LMGP02070002
LPE P-18:1	-	-	-
LPE P-18:2	-	-	-
LPE P-20:0	LPE O-20:1	PE(P-20:0/0:0)	LMGP02070004
LPE P-20:1	-	-	-
LPE P-20:4	-	-	-
LPE P-20:5	-	-	-
LPE P-22:0	-	-	-
LPE P-22:1	-	-	-
LPE P-22:4	-	-	-
LPE P-22:5	-	-	-
LPE P-22:6	-	-	-

Phosphatidylethanolamines (95)

MxP® Quant 500 XL kit lipid annotation	Potential isobars within ± 0.5 Da	Potential isomers	Data base ID
PE 20:0	PE 20:0	PE(10:0/10:0)	LMGP02010101
	LPE 21:0	PE(21:0/0:0)	LMGP02050026
PE 28:0	PE 28:0	PE(18:0/10:0)	LMGP02010021
		PE(16:0/12:0)	LMGP02010308
		PE(14:0/14:0)	LMGP02010352
		PE(15:0/13:0)	LMGP02011237
		PE(13:0/15:0)	LMGP02011256
		PE(12:0/16:0)	LMGP02011262
		PE(12:0(11Me)/14:0(13Me))	LMGP02011271
	PE O-29:0	PE(O-16:0/13:0)	LMGP02020022
	PE 25:3;O3	OHHdiA-PE	LMGP20020043

MxP® Quant 500 XL kit lipid annotation	Potential isobars within ± 0.5 Da	Potential isomers	Data base ID
PE 28:1	PE 28:1	PE(12:0/16:1(9Z))	LMGP02010362
		PE(13:0/15:1(9Z))	LMGP02010384
		PE(14:0/14:1(9Z))	LMGP02010407
		PE(14:1(9Z)/14:0)	LMGP02010424
		PE(15:1(9Z)/13:0)	LMGP02010475
		PE(16:1(9Z)/12:0)	LMGP02010514
	PE O-29:1	PE(P-16:0/13:0)	LMGP02030008
	PE 27:2;O	OON-PE	LMGP20020038
	PE 25:4;O3	OKHdiA-PE	LMGP20020041
	PE 26:3;O2	OHOOA-PE	LMGP20020046
PE 30:0	PE 30:0	PE(15:0/15:0)	LMGP02010106
		PE(16:0/14:0)	LMGP02010297
		PE(14:0/16:0)	LMGP02010302
		PE(18:0/12:0)	LMGP02011207
		PE(17:0/13:0)	LMGP02011215
		PE(13:0/17:0)	LMGP02011255
		PE(12:0/18:0)	LMGP02011261
		PE(14:0(13Me)/14:0(13Me))	LMGP02011274
	PE O-31:0	PE(O-16:0/15:0)	LMGP02020025
		PE(O-18:0/13:0)	LMGP02020039
	PE 28:2;O2	PHODA-PE	LMGP20020027
PE 30:1	PE 30:1	PE(13:0/17:1(9Z))	LMGP02010387
		PE(14:1(9Z)/16:0)	LMGP02010427
		PE(15:0/15:1(9Z))	LMGP02010455
		PE(15:1(9Z)/15:0)	LMGP02010478
		PE(16:0/14:1(9Z))	LMGP02010506
		PE(16:1(9Z)/14:0)	LMGP02010516
		PE(17:1(9Z)/13:0)	LMGP02010562
		PE(18:1(9Z)/12:0)	LMGP02010635
		PE(14:0/16:1(9Z))	LMGP02011249
		PE(12:0/18:1(9Z))	LMGP02011260
	PE O-31:1	PE(O-16:0/15:1(9Z))	LMGP02020026
		PE(P-16:0/15:0)	LMGP02030011
		PE(P-18:0/13:0)	LMGP02030035
	PE 28:3;O2	PKODA-PE	LMGP20020026
PE 31:0	PE 31:0	PE(12:0/19:0)	LMGP02010370
		PE(13:0/18:0)	LMGP02010389
		PE(14:0/17:0)	LMGP02010409
		PE(17:0/14:0)	LMGP02010540
		PE(18:0/13:0)	LMGP02010621
		PE(19:0/12:0)	LMGP02011188
		PE(16:0/15:0)	LMGP02011229
		PE(15:0/16:0)	LMGP02011236
	PE O-32:0	PE(O-18:0/14:0)	LMGP02020040
		PE(O-20:0/12:0)	LMGP02020061
		PE(O-16:0/16:0)	LMGP02020103

MxP® Quant 500 XL kit lipid annotation	Potential isobars within ± 0.5 Da	Potential isomers	Data base ID
PE 32:0	PE 32:0	PE(16:0/16:0)	LMGP02010037
		PE(20:0/12:0)	LMGP02011182
		PE(19:0/13:0)	LMGP02011187
		PE(18:0/14:0)	LMGP02011206
		PE(17:0/15:0)	LMGP02011214
		PE(15:0/17:0)	LMGP02011234
		PE(14:0/18:0)	LMGP02011248
		PE(13:0/19:0)	LMGP02011254
		PE(12:0/20:0)	LMGP02011259
		PE(16:0(15Me)/14:0(13Me))	LMGP02011276
	PE O-33:0	PE(O-18:0/15:0)	LMGP02020042
		PE(O-20:0/13:0)	LMGP02020062
		PE(O-16:0/17:0)	LMGP02020102
PE 32:1	PE 32:1	PE(12:0/20:1(11Z))	LMGP02010372
		PE(13:0/19:1(9Z))	LMGP02010395
		PE(14:1(9Z)/18:0)	LMGP02010432
		PE(15:0/17:1(9Z))	LMGP02010456
		PE(15:1(9Z)/17:0)	LMGP02010482
		PE(16:1(9Z)/16:0)	LMGP02010520
		PE(17:0/15:1(9Z))	LMGP02010541
		PE(17:1(9Z)/15:0)	LMGP02010565
		PE(18:0/14:1(9Z))	LMGP02010622
		PE(19:1(9Z)/13:0)	LMGP02010794
		PE(20:1(11Z)/12:0)	LMGP02010841
		PE(18:1(9Z)/14:0)	LMGP02011199
		PE(16:0/16:1(9Z))	LMGP02011228
		PE(14:0/18:1(9Z))	LMGP02011247
		PE(15:0/16:0(9Cp))	LMGP02011268
	PE O-33:1	PE(O-16:0/17:1(9Z))	LMGP02020028
		PE(O-18:0/15:1(9Z))	LMGP02020043
		PE(P-16:0/17:0)	LMGP02030015
		PE(P-18:0/15:0)	LMGP02030038
		PE(P-20:0/13:0)	LMGP02030063
	PE 30:3;O2	OHODA-PE	LMGP20020050
PE 32:2	PE 32:2	PE(16:1(9Z)/16:1(9Z))	LMGP02010108
		PE(16:1(11Z)/16:1(11Z))	LMGP02010354
		PE(16:1(5Z)/16:1(5Z))	LMGP02010356
		PE(12:0/20:2(11Z,14Z))	LMGP02010373
		PE(14:1(9Z)/18:1(9Z))	LMGP02010433
		PE(15:0/17:2(9Z,12Z))	LMGP02010457
		PE(15:1(9Z)/17:1(9Z))	LMGP02010483
		PE(17:1(9Z)/15:1(9Z))	LMGP02010566
		PE(17:2(9Z,12Z)/15:0)	LMGP02010595
		PE(18:1(9Z)/14:1(9Z))	LMGP02010637
		PE(18:2(9Z,12Z)/14:0)	LMGP02010655
		PE(20:2(11Z,14Z)/12:0)	LMGP02010871
		PE(14:0/18:2(9Z,12Z))	LMGP02011246
	PE O-33:2	PE(O-16:0/17:2(9Z,12Z))	LMGP02020029
		PE(P-16:0/17:1(9Z))	LMGP02030016
		PE(P-18:0/15:1(9Z))	LMGP02030039
	PE 30:4;O2	OKODA-PE	LMGP20020048

MxP® Quant 500 XL kit lipid annotation	Potential isobars within ± 0.5 Da	Potential isomers	Data base ID
PE 33:0	PE-NMe 32:0	PE-NMe(16:0/16:0)	LMGP02010337
	PE 33:0	PE(12:0/21:0)	LMGP02010377
		PE(14:0/19:0)	LMGP02010413
		PE(17:0/16:0)	LMGP02010542
		PE(18:0/15:0)	LMGP02010623
		PE(21:0/12:0)	LMGP02011170
		PE(20:0/13:0)	LMGP02011181
		PE(19:0/14:0)	LMGP02011186
		PE(16:0/17:0)	LMGP02011227
		PE(15:0/18:0)	LMGP02011233
		PE(13:0/20:0)	LMGP02011253
	PE O-34:0	PE(O-20:0/14:0)	LMGP02020063
		PE(O-18:0/16:0)	LMGP02020094
		PE(O-16:0/18:0)	LMGP02020101
	PE 30:3;O3	OHDdiA-PE	LMGP20020051
PE 33:1	PE 33:1	PE(13:0/20:1(11Z))	LMGP02010396
		PE(14:0/19:1(9Z))	LMGP02010414
		PE(14:1(9Z)/19:0)	LMGP02010438
		PE(15:0/18:1(9Z))	LMGP02010458
		PE(15:1(9Z)/18:0)	LMGP02010485
		PE(16:1(9Z)/17:0)	LMGP02010521
		PE(17:0/16:1(9Z))	LMGP02010543
		PE(17:1(9Z)/16:0)	LMGP02010567
		PE(18:0/15:1(9Z))	LMGP02010624
		PE(18:1(9Z)/15:0)	LMGP02010638
		PE(19:0/14:1(9Z))	LMGP02010769
		PE(19:1(9Z)/14:0)	LMGP02010795
		PE(20:1(11Z)/13:0)	LMGP02010842
		PE(16:0/17:1(9Z))	LMGP02011226
		PE(16:0/16:0(9Cp))	LMGP02011266
	PE O-34:1	PE(O-16:0/18:1(9Z))	LMGP02020018
		PE(O-20:0/14:1(9Z))	LMGP02020064
		PE(O-18:0/16:1(9Z))	LMGP02020093
		PE(P-16:0/18:0)	LMGP02030018
		PE(P-18:0/16:0)	LMGP02030040
		PE(P-20:0/14:0)	LMGP02030064
	PE 30:4;O3	OKDdiA-PE	LMGP20020049
PE 33:2	PE 33:2	PE(13:0/20:2(11Z,14Z))	LMGP02010397
		PE(14:1(9Z)/19:1(9Z))	LMGP02010439
		PE(15:1(9Z)/18:1(9Z))	LMGP02010486
		PE(16:0/17:2(9Z,12Z))	LMGP02010508
		PE(16:1(9Z)/17:1(9Z))	LMGP02010522
		PE(17:1(9Z)/16:1(9Z))	LMGP02010568
		PE(17:2(9Z,12Z)/16:0)	LMGP02010597
		PE(18:1(9Z)/15:1(9Z))	LMGP02010639
		PE(18:2(9Z,12Z)/15:0)	LMGP02010657
		PE(19:1(9Z)/14:1(9Z))	LMGP02010796
		PE(20:2(11Z,14Z)/13:0)	LMGP02010872
		PE(15:0/18:2(9Z,12Z))	LMGP02011232
	PE O-34:2	PE(O-16:0/18:2(9Z,12Z))	LMGP02020100
		PE(P-18:0/16:1(9Z))	LMGP02030041
		PE(P-20:0/14:1(9Z))	LMGP02030065
		PE(P-16:0/18:1(9Z))	LMGP02030095

MxP® Quant 500 XL kit lipid annotation	Potential isobars within ± 0.5 Da	Potential isomers	Data base ID
PE 34:0	PE 34:0	PE(22:0/12:0)	LMGP02011165
		PE(21:0/13:0)	LMGP02011169
		PE(20:0/14:0)	LMGP02011180
		PE(19:0/15:0)	LMGP02011185
		PE(18:0/16:0)	LMGP02011205
		PE(17:0/17:0)	LMGP02011213
		PE(16:0/18:0)	LMGP02011225
		PE(15:0/19:0)	LMGP02011231
		PE(14:0/20:0)	LMGP02011243
		PE(13:0/21:0)	LMGP02011252
		PE(12:0/22:0)	LMGP02011258
	PE O-35:0	PE(O-18:0/17:0)	LMGP02020044
		PE(O-20:0/15:0)	LMGP02020065
		PE(O-16:0/19:0)	LMGP02020097
PE 34:1	PE 34:1	PE(16:0/18:1(9Z))	LMGP02010009
		PE(16:0/18:1(11Z))	LMGP02010010
		PE(18:1(9Z)/16:0)	LMGP02010099
		PE(16:0/18:1(7Z))	LMGP02010311
		PE(12:0/22:1(11Z))	LMGP02010378
		PE(14:0/20:1(11Z))	LMGP02010415
		PE(14:1(9Z)/20:0)	LMGP02010440
		PE(15:0/19:1(9Z))	LMGP02010462
		PE(15:1(9Z)/19:0)	LMGP02010491
		PE(16:1(9Z)/18:0)	LMGP02010524
		PE(17:0/17:1(9Z))	LMGP02010544
		PE(17:1(9Z)/17:0)	LMGP02010569
		PE(19:0/15:1(9Z))	LMGP02010770
		PE(19:1(9Z)/15:0)	LMGP02010797
		PE(20:0/14:1(9Z))	LMGP02010824
		PE(20:1(11Z)/14:0)	LMGP02010843
		PE(22:1(11Z)/12:0)	LMGP02011040
		PE(18:0/16:1(9Z))	LMGP02011204
	PE O-35:1	PE(O-16:0/19:1(9Z))	LMGP02020031
		PE(O-18:0/17:1(9Z))	LMGP02020045
		PE(O-20:0/15:1(9Z))	LMGP02020066
		PE(P-16:0/19:0)	LMGP02030022
		PE(P-18:0/17:0)	LMGP02030042
		PE(P-20:0/15:0)	LMGP02030066

MxP® Quant 500 XL kit lipid annotation	Potential isobars within ± 0.5 Da	Potential isomers	Data base ID
PE 34:2	PE 34:2	PE(16:0/18:2(9Z,12Z))	LMGP02010042
		PE(12:0/22:2(13Z,16Z))	LMGP02010379
		PE(14:0/20:2(11Z,14Z))	LMGP02010416
		PE(14:1(9Z)/20:1(11Z))	LMGP02010441
		PE(15:1(9Z)/19:1(9Z))	LMGP02010492
		PE(17:0/17:2(9Z,12Z))	LMGP02010545
		PE(17:2(9Z,12Z)/17:0)	LMGP02010599
		PE(19:1(9Z)/15:1(9Z))	LMGP02010798
		PE(20:1(11Z)/14:1(9Z))	LMGP02010844
		PE(20:2(11Z,14Z)/14:0)	LMGP02010873
		PE(22:2(13Z,16Z)/12:0)	LMGP02011071
		PE(18:2(9Z,12Z)/16:0)	LMGP02011194
		PE(18:1(9Z)/16:1(9Z))	LMGP02011198
		PE(17:1(9Z)/17:1(9Z))	LMGP02011209
		PE(16:1(9Z)/18:1(9Z))	LMGP02011220
		PE(16:0(9Cp)/16:0(9Cp))	LMGP02011269
	PE O-35:2	PE(O-18:0/17:2(9Z,12Z))	LMGP02020046
		PE(P-16:0/19:1(9Z))	LMGP02030023
		PE(P-18:0/17:1(9Z))	LMGP02030043
		PE(P-20:0/15:1(9Z))	LMGP02030067
PE 34:3	PE 34:3	PE(16:0/18:3(9Z,12Z,15Z))	LMGP02010041
		PE(18:3(9Z,12Z,15Z)/16:0)	LMGP02010047
		PE(14:0/20:3(8Z,11Z,14Z))	LMGP02010417
		PE(14:1(9Z)/20:2(11Z,14Z))	LMGP02010442
		PE(17:1(9Z)/17:2(9Z,12Z))	LMGP02010570
		PE(17:2(9Z,12Z)/17:1(9Z))	LMGP02010600
		PE(18:2(9Z,12Z)/16:1(9Z))	LMGP02010659
		PE(18:3(6Z,9Z,12Z)/16:0)	LMGP02010685
		PE(20:2(11Z,14Z)/14:1(9Z))	LMGP02010874
		PE(20:3(8Z,11Z,14Z)/14:0)	LMGP02010904
		PE(16:1(9Z)/18:2(9Z,12Z))	LMGP02011219
		PE(16:0/18:3(6Z,9Z,12Z))	LMGP02011224
	PE O-35:3	PE(P-18:0/17:2(9Z,12Z))	LMGP02030044
PE 34:4	PE 34:4	PE(12:0/22:4(7Z,10Z,13Z,16Z))	LMGP02010380
		PE(14:1(9Z)/20:3(8Z,11Z,14Z))	LMGP02010443
		PE(16:1(9Z)/18:3(6Z,9Z,12Z))	LMGP02010525
		PE(16:1(9Z)/18:3(9Z,12Z,15Z))	LMGP02010526
		PE(18:3(6Z,9Z,12Z)/16:1(9Z))	LMGP02010686
		PE(18:3(9Z,12Z,15Z)/16:1(9Z))	LMGP02010716
		PE(18:4(6Z,9Z,12Z,15Z)/16:0)	LMGP02010744
		PE(20:3(8Z,11Z,14Z)/14:1(9Z))	LMGP02010905
		PE(20:4(5Z,8Z,11Z,14Z)/14:0)	LMGP02010935
		PE(22:4(7Z,10Z,13Z,16Z)/12:0)	LMGP02011102
		PE(17:2(9Z,12Z)/17:2(9Z,12Z))	LMGP02011208
		PE(16:0/18:4(6Z,9Z,12Z,15Z))	LMGP02011223
		PE(14:0/20:4(5Z,8Z,11Z,14Z))	LMGP02011242

MxP® Quant 500 XL kit lipid annotation	Potential isobars within ± 0.5 Da	Potential isomers	Data base ID
PE 35:1	PE 35:1	PE(13:0/22:1(11Z))	LMGP02010402
		PE(14:1(9Z)/21:0)	LMGP02010446
		PE(15:0/20:1(11Z))	LMGP02010464
		PE(15:1(9Z)/20:0)	LMGP02010493
		PE(16:0/19:1(9Z))	LMGP02010509
		PE(16:1(9Z)/19:0)	LMGP02010527
		PE(17:1(9Z)/18:0)	LMGP02010571
		PE(18:0/17:1(9Z))	LMGP02010626
		PE(18:1(9Z)/17:0)	LMGP02010640
		PE(19:0/16:1(9Z))	LMGP02010771
		PE(19:1(9Z)/16:0)	LMGP02010799
		PE(20:0/15:1(9Z))	LMGP02010826
		PE(20:1(11Z)/15:0)	LMGP02010845
		PE(21:0/14:1(9Z))	LMGP02010991
		PE(22:1(11Z)/13:0)	LMGP02011041
		PE(17:0/18:1(9Z))	LMGP02011211
		PE(16:0/18:0(11Cp))	LMGP02011265
	PE 36:8	PE(18:4(6Z,9Z,12Z,15Z)/18:4(6Z,9Z,12Z,15Z))	LMGP02010754
	PE O-36:1	PE(O-16:0/20:1(11Z))	LMGP02020032
		PE(O-18:0/18:1(9Z))	LMGP02020047
		PE(O-20:0/16:1(9Z))	LMGP02020067
		PE(P-16:0/20:0)	LMGP02030024
		PE(P-18:0/18:0)	LMGP02030045
		PE(P-20:0/16:0)	LMGP02030068
PE 35:2	PE 35:2	PE(13:0/22:2(13Z,16Z))	LMGP02010403
		PE(15:0/20:2(11Z,14Z))	LMGP02010465
		PE(15:1(9Z)/20:1(11Z))	LMGP02010494
		PE(16:1(9Z)/19:1(9Z))	LMGP02010528
		PE(17:0/18:2(9Z,12Z))	LMGP02010546
		PE(17:1(9Z)/18:1(9Z))	LMGP02010572
		PE(17:2(9Z,12Z)/18:0)	LMGP02010601
		PE(18:0/17:2(9Z,12Z))	LMGP02010627
		PE(18:1(9Z)/17:1(9Z))	LMGP02010641
		PE(18:2(9Z,12Z)/17:0)	LMGP02010660
		PE(19:1(9Z)/16:1(9Z))	LMGP02010800
		PE(20:1(11Z)/15:1(9Z))	LMGP02010846
		PE(20:2(11Z,14Z)/15:0)	LMGP02010875
		PE(22:2(13Z,16Z)/13:0)	LMGP02011072
	PE O-36:2	PE(O-16:0/20:2(11Z,14Z))	LMGP02020033
		PE(O-18:0/18:2(9Z,12Z))	LMGP02020048
		PE(P-18:0/18:1(9Z))	LMGP02030004
		PE(P-16:0/20:1(11Z))	LMGP02030025
		PE(P-20:0/16:1(9Z))	LMGP02030069

MxP® Quant 500 XL kit lipid annotation	Potential isobars within ± 0.5 Da	Potential isomers	Data base ID
PE 35:3	PE 35:3	PE(15:0/20:3(8Z,11Z,14Z))	LMGP02010466
		PE(15:1(9Z)/20:2(11Z,14Z))	LMGP02010495
		PE(17:0/18:3(6Z,9Z,12Z))	LMGP02010547
		PE(17:0/18:3(9Z,12Z,15Z))	LMGP02010548
		PE(17:1(9Z)/18:2(9Z,12Z))	LMGP02010573
		PE(17:2(9Z,12Z)/18:1(9Z))	LMGP02010602
		PE(18:1(9Z)/17:2(9Z,12Z))	LMGP02010642
		PE(18:2(9Z,12Z)/17:1(9Z))	LMGP02010661
		PE(18:3(6Z,9Z,12Z)/17:0)	LMGP02010687
		PE(18:3(9Z,12Z,15Z)/17:0)	LMGP02010717
		PE(20:2(11Z,14Z)/15:1(9Z))	LMGP02010876
		PE(20:3(8Z,11Z,14Z)/15:0)	LMGP02010906
	PE O-36:3	PE(O-18:0/18:3(6Z,9Z,12Z))	LMGP02020049
		PE(O-18:0/18:3(9Z,12Z,15Z))	LMGP02020050
		PE(O-16:0/20:3(8Z,11Z,14Z))	LMGP02020096
		PE(P-16:0/20:2(11Z,14Z))	LMGP02030026
		PE(P-18:0/18:2(9Z,12Z))	LMGP02030046
PE 36:0	PE 36:0	PE(18:0/18:0)	LMGP02010097
		PE(16:0/20:0)	LMGP02010270
		PE(17:0/19:0)	LMGP02010550
		PE(19:0/17:0)	LMGP02010772
		PE(21:0/15:0)	LMGP02010992
		PE(22:0/14:0)	LMGP02011163
		PE(20:0/16:0)	LMGP02011179
		PE(15:0/21:0)	LMGP02011230
		PE(14:0/22:0)	LMGP02011240
	PE 37:7	PE(15:1(9Z)/22:6(4Z,7Z,10Z,13Z,16Z,19Z))	LMGP02010504
		PE(17:2(9Z,12Z)/20:5(5Z,8Z,11Z,14Z,17Z))	LMGP02010614
		PE(20:5(5Z,8Z,11Z,14Z,17Z)/17:2(9Z,12Z))	LMGP02010972
		PE(22:6(4Z,7Z,10Z,13Z,16Z,19Z)/15:1(9Z))	LMGP02011137
	PE O-37:0	PE(O-16:0/21:0)	LMGP02020034
		PE(O-18:0/19:0)	LMGP02020052
		PE(O-20:0/17:0)	LMGP02020068
		PE(P-16:0/22:6(4Z,7Z,10Z,13Z,16Z,19Z))	LMGP02030001

MxP® Quant 500 XL kit lipid annotation	Potential isobars within ± 0.5 Da	Potential isomers	Data base ID
PE 36:1	PE 36:1	PE(18:0/18:1(9Z))	LMGP02010036
		PE(18:1(9Z)/18:0)	LMGP02010050
		PE(18:0/18:1(7Z))	LMGP02010301
		PE(16:0/20:1(11Z))	LMGP02010312
		PE(14:0/22:1(11Z))	LMGP02010419
		PE(14:1(9Z)/22:0)	LMGP02010447
		PE(15:1(9Z)/21:0)	LMGP02010499
		PE(16:1(9Z)/20:0)	LMGP02010529
		PE(17:0/19:1(9Z))	LMGP02010551
		PE(17:1(9Z)/19:0)	LMGP02010577
		PE(19:0/17:1(9Z))	LMGP02010773
		PE(19:1(9Z)/17:0)	LMGP02010801
		PE(20:0/16:1(9Z))	LMGP02010827
		PE(20:1(11Z)/16:0)	LMGP02010847
		PE(21:0/15:1(9Z))	LMGP02010993
		PE(22:0/14:1(9Z))	LMGP02011016
		PE(22:1(11Z)/14:0)	LMGP02011042
	PE O-37:1	PE(O-18:0/19:1(9Z))	LMGP02020053
		PE(O-20:0/17:1(9Z))	LMGP02020069
		PE(P-16:0/21:0)	LMGP02030029
		PE(P-18:0/19:0)	LMGP02030050
		PE(P-20:0/17:0)	LMGP02030070
PE 36:2	PE 36:2	PE(18:1(9E)/18:1(9E))	LMGP02010039
		PE(18:0/18:2(9Z,12Z))	LMGP02010044
		PE(18:1(9Z)/18:1(9Z))	LMGP02010052
		PE(18:1(6Z)/18:1(6Z))	LMGP02010109
		PE(14:0/22:2(13Z,16Z))	LMGP02010420
		PE(14:1(9Z)/22:1(11Z))	LMGP02010448
		PE(16:0/20:2(11Z,14Z))	LMGP02010510
		PE(16:1(9Z)/20:1(11Z))	LMGP02010530
		PE(17:1(9Z)/19:1(9Z))	LMGP02010578
		PE(17:2(9Z,12Z)/19:0)	LMGP02010607
		PE(19:0/17:2(9Z,12Z))	LMGP02010774
		PE(19:1(9Z)/17:1(9Z))	LMGP02010802
		PE(20:1(11Z)/16:1(9Z))	LMGP02010848
		PE(20:2(11Z,14Z)/16:0)	LMGP02010877
		PE(22:1(11Z)/14:1(9Z))	LMGP02011043
		PE(22:2(13Z,16Z)/14:0)	LMGP02011073
		PE(18:2(9Z,12Z)/18:0)	LMGP02011193
		PE(18:0(11Cp)/16:0(9Cp))	LMGP02011270
	PE O-37:2	PE(O-20:0/17:2(9Z,12Z))	LMGP02020070
		PE(P-18:0/19:1(9Z))	LMGP02030051
		PE(P-20:0/17:1(9Z))	LMGP02030071

MxP® Quant 500 XL kit lipid annotation	Potential isobars within ± 0.5 Da	Potential isomers	Data base ID
PE 36:3	PE 36:3	PE(18:1(9Z)/18:2(9Z,12Z))	LMGP02010048
		PE(14:1(9Z)/22:2(13Z,16Z))	LMGP02010449
		PE(16:1(9Z)/20:2(11Z,14Z))	LMGP02010531
		PE(17:2(9Z,12Z)/19:1(9Z))	LMGP02010608
		PE(18:0/18:3(6Z,9Z,12Z))	LMGP02010628
		PE(18:0/18:3(9Z,12Z,15Z))	LMGP02010629
		PE(18:2(9Z,12Z)/18:1(9Z))	LMGP02010663
		PE(18:3(6Z,9Z,12Z)/18:0)	LMGP02010690
		PE(18:3(9Z,12Z,15Z)/18:0)	LMGP02010720
		PE(19:1(9Z)/17:2(9Z,12Z))	LMGP02010803
		PE(20:2(11Z,14Z)/16:1(9Z))	LMGP02010878
		PE(20:3(8Z,11Z,14Z)/16:0)	LMGP02010908
		PE(22:2(13Z,16Z)/14:1(9Z))	LMGP02011074
		PE(16:0/20:3(8Z,11Z,14Z))	LMGP02011222
	PE O-37:3	PE(P-20:0/17:2(9Z,12Z))	LMGP02030072
PE 36:4	PE 36:4	PE(16:0/20:4(5Z,8Z,11Z,14Z))	LMGP02010096
		PE(18:2(9Z,12Z)/18:2(9Z,12Z))	LMGP02010111
		PE(18:2(6Z,9Z)/18:2(6Z,9Z))	LMGP02010112
		PE(14:0/22:4(7Z,10Z,13Z,16Z))	LMGP02010421
		PE(16:1(9Z)/20:3(8Z,11Z,14Z))	LMGP02010532
		PE(18:1(9Z)/18:3(6Z,9Z,12Z))	LMGP02010643
		PE(18:3(6Z,9Z,12Z)/18:1(9Z))	LMGP02010691
		PE(18:4(6Z,9Z,12Z,15Z)/18:0)	LMGP02010749
		PE(20:3(8Z,11Z,14Z)/16:1(9Z))	LMGP02010909
		PE(22:4(7Z,10Z,13Z,16Z)/14:0)	LMGP02011104
		PE(20:4(5Z,8Z,11Z,14Z)/16:0)	LMGP02011174
		PE(18:3(9Z,12Z,15Z)/18:1(9Z))	LMGP02011190
		PE(18:1(9Z)/18:3(9Z,12Z,15Z))	LMGP02011197
		PE(18:0/18:4(6Z,9Z,12Z,15Z))	LMGP02011203
	PE O-36:5;O	PE(P-16:0/20:4(5Z,8Z,10E,14Z)(12OH[S]))	LMGP20020005
		PE(P-16:0/20:4(5Z,8Z,11Z,13E)(15OH[S]))	LMGP20020008
		PE(P-16:0/20:4(6E,8Z,11Z,14Z)(5OH[S]))	LMGP20020011

MxP® Quant 500 XL kit lipid annotation	Potential isobars within ± 0.5 Da	Potential isomers	Data base ID
PE 36:5	PE 36:5	PE(14:1(9Z)/22:4(7Z,10Z,13Z,16Z))	LMGP02010450
		PE(18:1(9Z)/18:4(6Z,9Z,12Z,15Z))	LMGP02010644
		PE(18:2(9Z,12Z)/18:3(6Z,9Z,12Z))	LMGP02010664
		PE(18:2(9Z,12Z)/18:3(9Z,12Z,15Z))	LMGP02010665
		PE(18:3(6Z,9Z,12Z)/18:2(9Z,12Z))	LMGP02010692
		PE(18:3(9Z,12Z,15Z)/18:2(9Z,12Z))	LMGP02010721
		PE(18:4(6Z,9Z,12Z,15Z)/18:1(9Z))	LMGP02010750
		PE(20:4(5Z,8Z,11Z,14Z)/16:1(9Z))	LMGP02010939
		PE(20:5(5Z,8Z,11Z,14Z,17Z)/16:0)	LMGP02010968
		PE(22:4(7Z,10Z,13Z,16Z)/14:1(9Z))	LMGP02011105
		PE(16:1(9Z)/20:4(5Z,8Z,11Z,14Z))	LMGP02011217
		PE(16:0/20:5(5Z,8Z,11Z,14Z,17Z))	LMGP02011221
PE 36:6	PE 36:6	PE(16:1(9Z)/20:5(5Z,8Z,11Z,14Z,17Z))	LMGP02010533
		PE(18:2(9Z,12Z)/18:4(6Z,9Z,12Z,15Z))	LMGP02010666
		PE(18:3(6Z,9Z,12Z)/18:3(6Z,9Z,12Z))	LMGP02010693
		PE(18:3(6Z,9Z,12Z)/18:3(9Z,12Z,15Z))	LMGP02010694
		PE(18:3(9Z,12Z,15Z)/18:3(6Z,9Z,12Z))	LMGP02010722
		PE(18:4(6Z,9Z,12Z,15Z)/18:2(9Z,12Z))	LMGP02010751
		PE(20:5(5Z,8Z,11Z,14Z,17Z)/16:1(9Z))	LMGP02010969
		PE(22:6(4Z,7Z,10Z,13Z,16Z,19Z)/14:0)	LMGP02011134
		PE(18:3(9Z,12Z,15Z)/18:3(9Z,12Z,15Z))	LMGP02011189
		PE(14:0/22:6(4Z,7Z,10Z,13Z,16Z,19Z))	LMGP02011239
PE 38:0	PE 38:0	PE(18:0/20:0)	LMGP02010069
		PE(20:0/18:0)	LMGP02010131
		PE(16:0/22:0)	LMGP02010132
		PE(17:0/21:0)	LMGP02010556
		PE(17:1(9Z)/22:6(4Z,7Z,10Z,13Z,16Z,19Z))	LMGP02010590
		PE(21:0/17:0)	LMGP02010996
		PE(22:0/16:0)	LMGP02011019
		PE(22:6(4Z,7Z,10Z,13Z,16Z,19Z)/17:1(9Z))	LMGP02011140
		PE(19:0/19:0)	LMGP02011183
	PE O-39:0	PE(O-18:0/21:0)	LMGP02020057
		PE(O-20:0/19:0)	LMGP02020077
	PE O-40:7	PE(P-18:0/22:6(4Z,7Z,10Z,13Z,16Z,19Z))	LMGP02030005

MxP® Quant 500 XL kit lipid annotation	Potential isobars within ± 0.5 Da	Potential isomers	Data base ID
PE 38:1	PE 38:1	PE(20:0/18:1(9Z))	LMGP02010135
		PE(16:0/22:1(13Z))	LMGP02010136
		PE(20:0/18:1(13Z))	LMGP02010137
		PE(20:0/18:1(11Z))	LMGP02010138
		PE(20:0/18:1(7Z))	LMGP02010139
		PE(18:0/20:1(11Z))	LMGP02010141
		PE(18:1(9Z)/20:0)	LMGP02010143
		PE(16:0/22:1(11Z))	LMGP02010512
		PE(16:1(9Z)/22:0)	LMGP02010535
		PE(17:1(9Z)/21:0)	LMGP02010585
		PE(17:2(9Z,12Z)/22:6(4Z,7Z,10Z,13Z,16Z,19Z))	LMGP02010620
		PE(19:0/19:1(9Z))	LMGP02010780
		PE(19:1(9Z)/19:0)	LMGP02010810
		PE(20:1(11Z)/18:0)	LMGP02010852
		PE(21:0/17:1(9Z))	LMGP02010997
		PE(22:0/16:1(9Z))	LMGP02011020
		PE(22:1(11Z)/16:0)	LMGP02011046
		PE(22:6(4Z,7Z,10Z,13Z,16Z,19Z)/17:2(9Z,12Z))	LMGP02011141
	PE O-39:1	PE(O-20:0/19:1(9Z))	LMGP02020078
		PE(P-18:0/21:0)	LMGP02030057
		PE(P-20:0/19:0)	LMGP02030079
	PE O-40:8	PE(P-18:1(9Z)/22:6(4Z,7Z,10Z,13Z,16Z,19Z))	LMGP02030006
PE 38:2	PE 38:2	PE(20:2(5Z,8Z)/18:0)	LMGP02010122
		PE(18:0/20:2(11Z,14Z))	LMGP02010124
		PE(20:0/18:2(9Z,12Z))	LMGP02010125
		PE(18:1(9Z)/20:1(11Z))	LMGP02010126
		PE(16:0/22:2(13Z,16Z))	LMGP02010513
		PE(16:1(9Z)/22:1(11Z))	LMGP02010536
		PE(17:2(9Z,12Z)/21:0)	LMGP02010615
		PE(18:2(9Z,12Z)/20:0)	LMGP02010669
		PE(19:1(9Z)/19:1(9Z))	LMGP02010811
		PE(20:1(11Z)/18:1(9Z))	LMGP02010853
		PE(20:2(11Z,14Z)/18:0)	LMGP02010882
		PE(21:0/17:2(9Z,12Z))	LMGP02010998
		PE(22:1(11Z)/16:1(9Z))	LMGP02011047
		PE(22:2(13Z,16Z)/16:0)	LMGP02011077
	PE O-39:2	PE(P-20:0/19:1(9Z))	LMGP02030080
PE 38:3	PE 38:3	PE(16:1(9Z)/22:2(13Z,16Z))	LMGP02010537
		PE(18:1(9Z)/20:2(11Z,14Z))	LMGP02010647
		PE(18:2(9Z,12Z)/20:1(11Z))	LMGP02010670
		PE(18:3(6Z,9Z,12Z)/20:0)	LMGP02010698
		PE(18:3(9Z,12Z,15Z)/20:0)	LMGP02010726
		PE(20:0/18:3(6Z,9Z,12Z))	LMGP02010831
		PE(20:0/18:3(9Z,12Z,15Z))	LMGP02010832
		PE(20:1(11Z)/18:2(9Z,12Z))	LMGP02010854
		PE(20:2(11Z,14Z)/18:1(9Z))	LMGP02010883
		PE(20:3(8Z,11Z,14Z)/18:0)	LMGP02010913
		PE(22:2(13Z,16Z)/16:1(9Z))	LMGP02011078
		PE(18:0/20:3(8Z,11Z,14Z))	LMGP02011202

MxP® Quant 500 XL kit lipid annotation	Potential isobars within ± 0.5 Da	Potential isomers	Data base ID
PE 38:4	PE 38:4	PE(20:0/18:4(6Z,9Z,12Z,15Z))	LMGP02010114
		PE(18:1(9Z)/20:3(5Z,8Z,11Z))	LMGP02010115
		PE(16:0/22:4(7Z,10Z,13Z,16Z))	LMGP02010116
		PE(18:0/20:4(5Z,8Z,11Z,14Z))	LMGP02010118
		PE(18:0/20:4(5E,8E,11E,14E))	LMGP02010129
		PE(18:1(9Z)/20:3(8Z,11Z,14Z))	LMGP02010648
		PE(18:2(9Z,12Z)/20:2(11Z,14Z))	LMGP02010671
		PE(18:3(6Z,9Z,12Z)/20:1(11Z))	LMGP02010699
		PE(18:3(9Z,12Z,15Z)/20:1(11Z))	LMGP02010727
		PE(18:4(6Z,9Z,12Z,15Z)/20:0)	LMGP02010757
		PE(20:1(11Z)/18:3(6Z,9Z,12Z))	LMGP02010855
		PE(20:1(11Z)/18:3(9Z,12Z,15Z))	LMGP02010856
		PE(20:2(11Z,14Z)/18:2(9Z,12Z))	LMGP02010884
		PE(20:3(8Z,11Z,14Z)/18:1(9Z))	LMGP02010914
		PE(20:4(5Z,8Z,11Z,14Z)/18:0)	LMGP02010943
		PE(22:4(7Z,10Z,13Z,16Z)/16:0)	LMGP02011108
	PE O-38:5;O	PE(P-18:0/20:4(5Z,8Z,10E,14Z)(12OH[S]))	LMGP20020007
		PE(P-18:0/20:4(5Z,8Z,11Z,13E)(15OH[S]))	LMGP20020010
		PE(P-18:0/20:4(6E,8Z,11Z,14Z)(5OH[S]))	LMGP20020013

MxP® Quant 500 XL kit lipid annotation	Potential isobars within ± 0.5 Da	Potential isomers	Data base ID
PE 38:5	PE 38:5	PE(16:1(9Z)/22:4(7Z,10Z,13Z,16Z))	LMGP02010538
		PE(18:2(9Z,12Z)/20:3(8Z,11Z,14Z))	LMGP02010672
		PE(18:3(6Z,9Z,12Z)/20:2(11Z,14Z))	LMGP02010700
		PE(18:3(9Z,12Z,15Z)/20:2(11Z,14Z))	LMGP02010728
		PE(18:4(6Z,9Z,12Z,15Z)/20:1(11Z))	LMGP02010758
		PE(20:1(11Z)/18:4(6Z,9Z,12Z,15Z))	LMGP02010857
		PE(20:2(11Z,14Z)/18:3(6Z,9Z,12Z))	LMGP02010885
		PE(20:2(11Z,14Z)/18:3(9Z,12Z,15Z))	LMGP02010886
		PE(20:3(8Z,11Z,14Z)/18:2(9Z,12Z))	LMGP02010915
		PE(20:4(5Z,8Z,11Z,14Z)/18:1(9Z))	LMGP02010944
		PE(20:5(5Z,8Z,11Z,14Z,17Z)/18:0)	LMGP02010973
		PE(22:4(7Z,10Z,13Z,16Z)/16:1(9Z))	LMGP02011109
		PE(18:1(9Z)/20:4(5Z,8Z,11Z,14Z))	LMGP02011196
		PE(18:0/20:5(5Z,8Z,11Z,14Z,17Z))	LMGP02011201
	PE O-38:6;O	PE(P-18:1(9Z)/20:4(5Z,8Z,10E,14Z)(12OH[S]))	LMGP20020006
		PE(P-18:1(9Z)/20:4(5Z,8Z,11Z,13E)(15OH[S]))	LMGP20020009
		PE(P-18:1(9Z)/20:4(6E,8Z,11Z,14Z)(5OH[S]))	LMGP20020012

MxP® Quant 500 XL kit lipid annotation	Potential isobars within ± 0.5 Da	Potential isomers	Data base ID
PE 38:6	PE 38:6	PE(16:0/22:6(4Z,7Z,10Z,13Z,16Z,19Z))	LMGP02010095
		PE(18:3(6Z,9Z,12Z)/20:3(8Z,11Z,14Z))	LMGP02010701
		PE(18:3(9Z,12Z,15Z)/20:3(8Z,11Z,14Z))	LMGP02010729
		PE(18:4(6Z,9Z,12Z,15Z)/20:2(11Z,14Z))	LMGP02010759
		PE(20:2(11Z,14Z)/18:4(6Z,9Z,12Z,15Z))	LMGP02010887
		PE(20:3(8Z,11Z,14Z)/18:3(6Z,9Z,12Z))	LMGP02010916
		PE(20:3(8Z,11Z,14Z)/18:3(9Z,12Z,15Z))	LMGP02010917
		PE(20:4(5Z,8Z,11Z,14Z)/18:2(9Z,12Z))	LMGP02010945
		PE(22:6(4Z,7Z,10Z,13Z,16Z,19Z)/16:0)	LMGP02011161
		PE(20:5(5Z,8Z,11Z,14Z,17Z)/18:1(9Z))	LMGP02011172
		PE(18:2(9Z,12Z)/20:4(5Z,8Z,11Z,14Z))	LMGP02011192
		PE(18:1(9Z)/20:5(5Z,8Z,11Z,14Z,17Z))	LMGP02011195
	PE O-38:7;O	PE(P-16:0/22:6(4Z,7Z,10Z,12E,16Z,19Z)(14OH))	LMGP20020014
PE 38:7	PE 37:0	PE(17:0/20:0)	LMGP02010067
		PE(15:0/22:0)	LMGP02010469
		PE(16:0/21:0)	LMGP02010511
		PE(18:0/19:0)	LMGP02010630
		PE(20:0/17:0)	LMGP02010828
		PE(21:0/16:0)	LMGP02010994
		PE(22:0/15:0)	LMGP02011017
		PE(19:0/18:0)	LMGP02011184
	PE 38:7	PE(18:2(9Z,12Z)/20:5(5Z,8Z,11Z,14Z,17Z))	LMGP02010673
		PE(18:3(6Z,9Z,12Z)/20:4(5Z,8Z,11Z,14Z))	LMGP02010702
		PE(18:3(9Z,12Z,15Z)/20:4(5Z,8Z,11Z,14Z))	LMGP02010730
		PE(18:4(6Z,9Z,12Z,15Z)/20:3(8Z,11Z,14Z))	LMGP02010760
		PE(20:3(8Z,11Z,14Z)/18:4(6Z,9Z,12Z,15Z))	LMGP02010918
		PE(20:4(5Z,8Z,11Z,14Z)/18:3(6Z,9Z,12Z))	LMGP02010946
		PE(20:4(5Z,8Z,11Z,14Z)/18:3(9Z,12Z,15Z))	LMGP02010947
		PE(20:5(5Z,8Z,11Z,14Z,17Z)/18:2(9Z,12Z))	LMGP02010974
		PE(22:6(4Z,7Z,10Z,13Z,16Z,19Z)/16:1(9Z))	LMGP02011138
		PE(16:1(9Z)/22:6(4Z,7Z,10Z,13Z,16Z,19Z))	LMGP02011216
	PE O-38:0	PE(O-18:0/20:0)	LMGP02020014
		PE(O-16:0/22:0)	LMGP02020015
		PE(O-20:0/18:0)	LMGP02020071

MxP® Quant 500 XL kit lipid annotation	Potential isobars within ± 0.5 Da	Potential isomers	Data base ID
PE 40:1	PE 40:1	PE(18:0/22:1(13Z))	LMGP02010151
		PE(20:0/20:1(11Z))	LMGP02010152
		PE(22:0/18:1(13Z))	LMGP02010153
		PE(22:0/18:1(11Z))	LMGP02010154
		PE(22:0/18:1(9Z))	LMGP02010155
		PE(22:0/18:1(7Z))	LMGP02010156
		PE(20:1(13E)/20:0)	LMGP02010157
		PE(18:1(11E)/22:0)	LMGP02010158
		PE(20:1(13Z)/20:0)	LMGP02010159
		PE(18:1(11Z)/22:0)	LMGP02010160
		PE(18:1(9Z)/22:0)	LMGP02010161
		PE(18:0/22:1(11Z))	LMGP02010633
		PE(19:1(9Z)/21:0)	LMGP02010818
		PE(20:1(11Z)/20:0)	LMGP02010860
		PE(21:0/19:1(9Z))	LMGP02011005
		PE(22:1(11Z)/18:0)	LMGP02011051
	PE O-41:1	PE(P-20:0/21:0)	LMGP02030087
PE 40:3	PE 40:3	PE(18:1(9Z)/22:2(13Z,16Z))	LMGP02010651
		PE(18:2(9Z,12Z)/22:1(11Z))	LMGP02010676
		PE(18:3(6Z,9Z,12Z)/22:0)	LMGP02010705
		PE(18:3(9Z,12Z,15Z)/22:0)	LMGP02010733
		PE(20:0/20:3(8Z,11Z,14Z))	LMGP02010835
		PE(20:1(11Z)/20:2(11Z,14Z))	LMGP02010861
		PE(20:2(11Z,14Z)/20:1(11Z))	LMGP02010891
		PE(20:3(8Z,11Z,14Z)/20:0)	LMGP02010921
		PE(22:0/18:3(6Z,9Z,12Z))	LMGP02011025
		PE(22:0/18:3(9Z,12Z,15Z))	LMGP02011026
		PE(22:1(11Z)/18:2(9Z,12Z))	LMGP02011053
		PE(22:2(13Z,16Z)/18:1(9Z))	LMGP02011083
PE 40:4	PE 40:4	PE(18:2(9Z,12Z)/22:2(13Z,16Z))	LMGP02010677
		PE(18:3(6Z,9Z,12Z)/22:1(11Z))	LMGP02010706
		PE(18:3(9Z,12Z,15Z)/22:1(11Z))	LMGP02010734
		PE(18:4(6Z,9Z,12Z,15Z)/22:0)	LMGP02010764
		PE(20:1(11Z)/20:3(8Z,11Z,14Z))	LMGP02010862
		PE(20:2(11Z,14Z)/20:2(11Z,14Z))	LMGP02010892
		PE(20:3(8Z,11Z,14Z)/20:1(11Z))	LMGP02010922
		PE(20:4(5Z,8Z,11Z,14Z)/20:0)	LMGP02010951
		PE(22:0/18:4(6Z,9Z,12Z,15Z))	LMGP02011027
		PE(22:1(11Z)/18:3(6Z,9Z,12Z))	LMGP02011054
		PE(22:1(11Z)/18:3(9Z,12Z,15Z))	LMGP02011055
		PE(22:2(13Z,16Z)/18:2(9Z,12Z))	LMGP02011084
		PE(22:4(7Z,10Z,13Z,16Z)/18:0)	LMGP02011113
		PE(20:0/20:4(5Z,8Z,11Z,14Z))	LMGP02011177
		PE(18:0/22:4(7Z,10Z,13Z,16Z))	LMGP02011200

MxP® Quant 500 XL kit lipid annotation	Potential isobars within ± 0.5 Da	Potential isomers	Data base ID
PE 40:5	PE 40:5	PE(18:1(9Z)/22:4(7Z,10Z,13Z,16Z))	LMGP02010652
		PE(18:3(6Z,9Z,12Z)/22:2(13Z,16Z))	LMGP02010707
		PE(18:3(9Z,12Z,15Z)/22:2(13Z,16Z))	LMGP02010735
		PE(18:4(6Z,9Z,12Z,15Z)/22:1(11Z))	LMGP02010765
		PE(20:0/20:5(5Z,8Z,11Z,14Z,17Z))	LMGP02010836
		PE(20:1(11Z)/20:4(5Z,8Z,11Z,14Z))	LMGP02010863
		PE(20:2(11Z,14Z)/20:3(8Z,11Z,14Z))	LMGP02010893
		PE(20:3(8Z,11Z,14Z)/20:2(11Z,14Z))	LMGP02010923
		PE(20:4(5Z,8Z,11Z,14Z)/20:1(11Z))	LMGP02010952
		PE(20:5(5Z,8Z,11Z,14Z,17Z)/20:0)	LMGP02010980
		PE(22:1(11Z)/18:4(6Z,9Z,12Z,15Z))	LMGP02011056
		PE(22:2(13Z,16Z)/18:3(6Z,9Z,12Z))	LMGP02011085
		PE(22:2(13Z,16Z)/18:3(9Z,12Z,15Z))	LMGP02011086
		PE(22:4(7Z,10Z,13Z,16Z)/18:1(9Z))	LMGP02011114
PE 40:6	PE 40:6	PE(18:0/22:6(4Z,7Z,10Z,13Z,16Z,19Z))	LMGP02010094
		PE(18:2(9Z,12Z)/22:4(7Z,10Z,13Z,16Z))	LMGP02010678
		PE(18:4(6Z,9Z,12Z,15Z)/22:2(13Z,16Z))	LMGP02010766
		PE(20:1(11Z)/20:5(5Z,8Z,11Z,14Z,17Z))	LMGP02010864
		PE(20:2(11Z,14Z)/20:4(5Z,8Z,11Z,14Z))	LMGP02010894
		PE(20:3(8Z,11Z,14Z)/20:3(8Z,11Z,14Z))	LMGP02010924
		PE(20:4(5Z,8Z,11Z,14Z)/20:2(11Z,14Z))	LMGP02010953
		PE(20:5(5Z,8Z,11Z,14Z,17Z)/20:1(11Z))	LMGP02010981
		PE(22:2(13Z,16Z)/18:4(6Z,9Z,12Z,15Z))	LMGP02011087
		PE(22:4(7Z,10Z,13Z,16Z)/18:2(9Z,12Z))	LMGP02011115
		PE(22:6(4Z,7Z,10Z,13Z,16Z,19Z)/18:0)	LMGP02011142
	PE O-40:7;O	PE(P-18:0/22:6(4Z,7Z,10Z,12E,16Z,19Z)(14OH))	LMGP20020015

MxP® Quant 500 XL kit lipid annotation	Potential isobars within ± 0.5 Da	Potential isomers	Data base ID
PE 40:7	PE 39:0	PE(17:0/22:0)	LMGP02010557
		PE(18:0/21:0)	LMGP02010632
		PE(19:0/20:0)	LMGP02010781
		PE(20:0/19:0)	LMGP02010833
		PE(22:0/17:0)	LMGP02011021
		PE(21:0/18:0)	LMGP02011167
	PE 40:7	PE(18:3(6Z,9Z,12Z)/22:4(7Z,10Z,13Z,16Z))	LMGP02010708
		PE(18:3(9Z,12Z,15Z)/22:4(7Z,10Z,13Z,16Z))	LMGP02010736
		PE(20:2(11Z,14Z)/20:5(5Z,8Z,11Z,14Z,17Z))	LMGP02010895
		PE(20:3(8Z,11Z,14Z)/20:4(5Z,8Z,11Z,14Z))	LMGP02010925
		PE(20:4(5Z,8Z,11Z,14Z)/20:3(8Z,11Z,14Z))	LMGP02010954
		PE(20:5(5Z,8Z,11Z,14Z,17Z)/20:2(11Z,14Z))	LMGP02010982
		PE(22:4(7Z,10Z,13Z,16Z)/18:3(6Z,9Z,12Z))	LMGP02011116
		PE(22:4(7Z,10Z,13Z,16Z)/18:3(9Z,12Z,15Z))	LMGP02011117
		PE(22:6(4Z,7Z,10Z,13Z,16Z,19Z)/18:1(9Z))	LMGP02011143
		PE(18:1(9Z)/22:6(4Z,7Z,10Z,13Z,16Z,19Z))	LMGP02011264
	PE O-40:0	PE(O-18:0/22:0)	LMGP02020017
		PE(O-20:0/20:0)	LMGP02020079
PE 40:8	PE 39:1	PE(17:0/22:1(11Z))	LMGP02010558
		PE(17:1(9Z)/22:0)	LMGP02010586
		PE(18:1(9Z)/21:0)	LMGP02010649
		PE(19:0/20:1(11Z))	LMGP02010782
		PE(19:1(9Z)/20:0)	LMGP02010812
		PE(20:0/19:1(9Z))	LMGP02010834
		PE(20:1(11Z)/19:0)	LMGP02010858
		PE(21:0/18:1(9Z))	LMGP02010999
		PE(22:0/17:1(9Z))	LMGP02011022
		PE(22:1(11Z)/17:0)	LMGP02011048
	PE 40:8	PE(18:4(6Z,9Z,12Z,15Z)/22:4(7Z,10Z,13Z,16Z))	LMGP02010767
		PE(20:3(8Z,11Z,14Z)/20:5(5Z,8Z,11Z,14Z,17Z))	LMGP02010926
		PE(20:5(5Z,8Z,11Z,14Z,17Z)/20:3(8Z,11Z,14Z))	LMGP02010983
		PE(22:4(7Z,10Z,13Z,16Z)/18:4(6Z,9Z,12Z,15Z))	LMGP02011118
		PE(22:6(4Z,7Z,10Z,13Z,16Z,19Z)/18:2(9Z,12Z))	LMGP02011144
		PE(20:4(5Z,8Z,11Z,14Z)/20:4(5Z,8Z,11Z,14Z))	LMGP02011173
		PE(18:2(9Z,12Z)/22:6(4Z,7Z,10Z,13Z,16Z,19Z))	LMGP02011191
	PE O-40:1	PE(O-18:0/22:1(11Z))	LMGP02020058
		PE(O-20:0/20:1(11Z))	LMGP02020080
		PE(P-18:0/22:0)	LMGP02030058
		PE(P-20:0/20:0)	LMGP02030081

MxP® Quant 500 XL kit lipid annotation	Potential isobars within ± 0.5 Da	Potential isomers	Data base ID
PE 42:7	PE 41:0	PE(19:0/22:0)	LMGP02010788
		PE(20:0/21:0)	LMGP02010837
		PE(21:0/20:0)	LMGP02011006
		PE(22:0/19:0)	LMGP02011028
	PE 42:7	PE(20:1(11Z)/22:6(4Z,7Z,10Z,13Z,16Z,19Z))	LMGP02010870
		PE(20:3(8Z,11Z,14Z)/22:4(7Z,10Z,13Z,16Z))	LMGP02010931
		PE(20:5(5Z,8Z,11Z,14Z,17Z)/22:2(13Z,16Z))	LMGP02010989
		PE(22:2(13Z,16Z)/20:5(5Z,8Z,11Z,14Z,17Z))	LMGP02011095
		PE(22:4(7Z,10Z,13Z,16Z)/20:3(8Z,11Z,14Z))	LMGP02011124
		PE(22:6(4Z,7Z,10Z,13Z,16Z,19Z)/20:1(11Z))	LMGP02011151
	PE O-42:0	PE(O-20:0/22:0)	LMGP02020090
PE 42:8	PE 41:1	PE(19:0/22:1(11Z))	LMGP02010789
		PE(19:1(9Z)/22:0)	LMGP02010819
		PE(20:1(11Z)/21:0)	LMGP02010865
		PE(21:0/20:1(11Z))	LMGP02011007
		PE(22:0/19:1(9Z))	LMGP02011029
		PE(22:1(11Z)/19:0)	LMGP02011057
	PE 42:8	PE(20:2(11Z,14Z)/22:6(4Z,7Z,10Z,13Z,16Z,19Z))	LMGP02010901
		PE(20:4(5Z,8Z,11Z,14Z)/22:4(7Z,10Z,13Z,16Z))	LMGP02010960
		PE(22:4(7Z,10Z,13Z,16Z)/20:4(5Z,8Z,11Z,14Z))	LMGP02011125
		PE(22:6(4Z,7Z,10Z,13Z,16Z,19Z)/20:2(11Z,14Z))	LMGP02011152
	PE O-42:1	PE(O-20:0/22:1(11Z))	LMGP02020086
		PE(P-20:0/22:0)	LMGP02030088
PE 44:6	PE 44:6	PE(22:0/22:6(4Z,7Z,10Z,13Z,16Z,19Z))	LMGP02011039
		PE(22:2(13Z,16Z)/22:4(7Z,10Z,13Z,16Z))	LMGP02011100
		PE(22:4(7Z,10Z,13Z,16Z)/22:2(13Z,16Z))	LMGP02011130
		PE(22:6(4Z,7Z,10Z,13Z,16Z,19Z)/22:0)	LMGP02011157
PE 44:7	PE 43:0	PE(21:0/22:0)	LMGP02011012
		PE(22:0/21:0)	LMGP02011035
	PE 44:7	PE(22:1(11Z)/22:6(4Z,7Z,10Z,13Z,16Z,19Z))	LMGP02011070
		PE(22:6(4Z,7Z,10Z,13Z,16Z,19Z)/22:1(11Z))	LMGP02011158
PE 44:11	PE 44:11	PE(22:5(7Z,10Z,13Z,16Z,19Z)/22:6(4Z,7Z,10Z,13Z,16Z,19Z))	Pubchem:53479968
		PE(22:5(4Z,7Z,10Z,13Z,16Z)/22:6(4Z,7Z,10Z,13Z,16Z,19Z))	Pubchem:53479935
	PE 43:4	PE(21:0/22:4(7Z,10Z,13Z,16Z))	LMGP02011015
		PE(22:4(7Z,10Z,13Z,16Z)/21:0)	LMGP02011127
PE 44:12	PE 44:12	PE(22:6(4Z,7Z,10Z,13Z,16Z,19Z)/22:6(4Z,7Z,10Z,13Z,16Z,19Z))	LMGP02010093
	PE-GlcDG P-16:0/16:1	PE-GlcDG(P-16:0/16:1(9Z))	LMGL05020001
PE P-16:0/14:0	PE P-16:0/14:0	PE(P-16:0/14:0)	LMGP02030009
PE P-16:0/15:0	PE P-16:0/15:0	PE(P-16:0/15:0)	LMGP02030011
PE P-16:0/16:0	PE P-16:0/16:0	PE(P-16:0/16:0)	LMGP02030013

MxP® Quant 500 XL kit lipid annotation	Potential isobars within ± 0.5 Da	Potential isomers	Data base ID
PE P-16:0/16:1	PE P-16:0/16:1	PE(P-16:0/16:1(9Z))	LMGP02030014
PE P-16:0/18:1	PE P-16:0/18:1	PE(P-16:0/18:1(9Z))	LMGP02030095
PE P-16:0/18:2	PE P-16:0/18:2	PE(P-16:0/18:2(9Z,12Z))	LMGP02030094
PE P-16:0/18:3	PE P-16:0/18:3	PE(P-16:0/18:3(6Z,9Z,12Z))	LMGP02030019
		PE(P-16:0/18:3(9Z,12Z,15Z))	LMGP02030020
PE P-16:0/20:3	PE P-16:0/20:3	PE(P-16:0/20:3(8Z,11Z,14Z))	LMGP02030027
PE P-16:0/20:4	PE P-16:0/20:4	PE(P-16:0/20:4(5Z,8Z,11Z,14Z))	LMGP02030093
PE P-16:0/20:5	PE P-16:0/20:5	PE(P-16:0/20:5(5Z,8Z,11Z,14Z,17Z))	LMGP02030028
PE P-16:0/22:4	PE P-16:0/22:4	PE(P-16:0/22:4(7Z,10Z,13Z,16Z))	LMGP02030033
PE P-16:0/22:5	PE P-16:0/22:5	PE(P-16:0/22:5(4Z,7Z,10Z,13Z,16Z))	PubChem:53480849
		PE(P-16:0/22:5(7Z,10Z,13Z,16Z,19Z))	PubChem:53480850
PE P-16:0/22:6	PE P-16:0/22:6	PE(P-16:0/22:6(4Z,7Z,10Z,13Z,16Z,19Z))	LMGP02030001
PE P-18:0/14:0	PE P-18:0/14:0	PE(P-18:0/14:0)	LMGP02030036
PE P-18:0/16:0	PE P-18:0/16:0	PE(P-18:0/16:0)	LMGP02030040
PE P-18:0/16:1	PE P-18:0/16:1	PE(P-18:0/16:1(9Z))	LMGP02030041
PE P-18:0/17:1	PE P-18:0/17:1	PE(P-18:0/17:1(9Z))	LMGP02030043
PE P-18:0/18:0	PE P-18:0/18:0	PE(P-18:0/18:0)	LMGP02030045
PE P-18:0/18:1	PE P-18:0/18:1	PE(P-18:0/18:1(9Z))	LMGP02030004
PE P-18:0/18:2	PE P-18:0/18:2	PE(P-18:0/18:2(9Z,12Z))	LMGP02030046
PE P-18:0/18:3	PE P-18:0/18:3	PE(P-18:0/18:3(6Z,9Z,12Z))	LMGP02030047
		PE(P-18:0/18:3(9Z,12Z,15Z))	LMGP02030048
PE P-18:0/19:1	PE P-18:0/19:1	PE(P-18:0/19:1(9Z))	LMGP02030051
PE P-18:0/20:1	PE P-18:0/20:1	PE(P-18:0/20:1(11Z))	LMGP02030053
PE P-18:0/20:2	PE P-18:0/20:2	PE(P-18:0/20:2(11Z,14Z))	LMGP02030054
PE P-18:0/20:3	PE P-18:0/20:3	PE(P-18:0/20:3(8Z,11Z,14Z))	LMGP02030055
PE P-18:0/20:4	PE P-18:0/20:4	PE(P-18:0/20:4(5Z,8Z,11Z,14Z))	LMGP02030003
PE P-18:0/20:5	PE P-18:0/20:5	PE(P-18:0/20:5(5Z,8Z,11Z,14Z,17Z))	LMGP02030056
PE P-18:0/22:1	PE P-18:0/22:1	PE(P-18:0/22:1(11Z))	LMGP02030059
PE P-18:0/22:2	PE P-18:0/22:2	PE(P-18:0/22:2(13Z,16Z))	LMGP02030060
PE P-18:0/22:3	PE P-18:0/22:3	-	-
PE P-18:0/22:4	PE P-18:0/22:4	PE(P-18:0/22:4(7Z,10Z,13Z,16Z))	LMGP02030061
PE P-18:0/22:5	PE P-18:0/22:5	PE(P-18:0/22:5(7Z,10Z,13Z,16Z,19Z))	PubChem:53480858
		PE(P-18:0/22:5(4Z,7Z,10Z,13Z,16Z))	PubChem:53480857
		PE(P-18:0/20:5(5Z,8Z,11Z,14Z,17Z))	LMGP02030056
PE P-18:0/22:6	PE P-18:0/22:6	PE(P-18:0/22:6(4Z,7Z,10Z,13Z,16Z,19Z))	LMGP02030005
PE P-18:1/18:1	PE P-18:1/18:1	PE(P-18:1/18:1)	PubChem:85342034
PE P-18:1/18:2	PE P-18:1/18:2	PE(P-18:1(11Z)/18:2(9Z,12Z))	PubChem:53480869
PE P-18:1/20:4	PE P-18:1/20:4	PE(P-18:1/20:4)	PubChem:85366510
		PE P-18:1/20:4	PubChem:242084114
PE P-18:1/20:5	PE P-18:1/20:5	-	-

MxP® Quant 500 XL kit lipid annotation	Potential isobars within ± 0.5 Da	Potential isomers	Data base ID
PE P-18:1/22:6	PE P-18:1/22:6	PE(P-18:1(9Z)/22:6(4Z,7Z,10Z,13Z,16Z,19Z))	LMGP02030006
PE P-20:0/14:0	PE P-20:0/14:0	PE(P-20:0/14:0)	LMGP02030064
PE P-20:0/16:0	PE P-20:0/16:0	PE(P-20:0/16:0)	LMGP02030068
PE P-20:0/16:1	PE P-20:0/16:1	PE(P-20:0/16:1(9Z))	LMGP02030069
PE P-20:0/17:1	PE P-20:0/17:1	PE(P-20:0/17:1(9Z))	LMGP02030071
PE P-20:0/18:1	PE P-20:0/18:1	PE(P-20:0/18:1(9Z))	LMGP02030074
PE P-20:0/18:2	PE P-20:0/18:2	PE(P-20:0/18:2(9Z,12Z))	LMGP02030075
PE P-20:0/20:0	PE P-20:0/20:0	PE(P-20:0/20:0)	LMGP02030081
PE P-20:0/20:4	PE P-20:0/20:4	PE(P-20:0/20:4(5Z,8Z,11Z,14Z))	LMGP02030085
PE P-20:0/20:5	PE P-20:0/20:5	PE(P-20:0/20:5(5Z,8Z,11Z,14Z,17Z))	LMGP02030086

Lysophosphatidylglycerols (10)			
MxP® Quant 500 XL kit lipid annotation	Potential isobars within ± 0.5 Da	Potential isomers	Data base ID
LPG 14:0	LPG 14:0	PG(14:0/0:0)	LMGP04050012
LPG 14:1	LPG 14:1	PG(14:1(9Z)/0:0)	LMGP04050035
LPG 16:0	LPG 16:0	PG(16:0/0:0)	LMGP04050008
LPG 16:1	LPG 16:1	PG(16:1(9Z)/0:0)	LMGP04050013
LPG 17:0	LPG 17:0	PG(17:0/0:0)	LMGP04050033
LPG 17:1	LPG 17:1	PG(17:1(9Z)/0:0)	LMGP04050036
LPG 18:0	LPG 18:0	PG(18:0/0:0)	LMGP04050009
LPG 18:1	LPG 18:1	PG(18:1(9Z)/0:0)	LMGP04050006
LPG 18:2	LPG 18:2	PG(18:2(9Z,12Z)/0:0)	LMGP04050014
LPG 20:1	LPG 20:1	PG(20:1(11Z)/0:0)	LMGP04050023

Phosphatidylglycerols (64)			
MxP® Quant 500 XL kit lipid annotation	Potential isobars within ± 0.5 Da	Potential isomers	Data base ID
PG 14:0_16:0	TG 16:0_24:0	TG(12:0/12:0/16:0)[iso3]	LMGL03012632
	PG 14:0_16:0	PG(14:0/16:0)	LMGP04010022
		PG(16:0/14:0)	LMGP04010974
	PA 16:0_20:5	PA(20:5(5Z,8Z,11Z,14Z,17Z)/16:0)	LMGP10010653
		PA(16:0/20:5(5Z,8Z,11Z,14Z,17Z))	LMGP10010904
PG 15:0_18:1	TG 18:1:25:0	TG(12:0/13:0/18:1(9Z))[iso6]	LMGL03013187
	PG 15:0_18:1	PG(15:0/18:1(9Z))	LMGP04010145
		PG(18:1(9Z)/15:0)	LMGP04010325
	PG O-16:0/18:1	PG(O-16:0/18:1(9Z))	LMGP04020092
PG 16:0_16:0	TG 16:0_26:0	TG(13:0/13:0/16:0)[iso3]	LMGL03012695
		TG(12:0/14:0/16:0)[iso6]	LMGL03013211
	PG 16:0_16:0	PG(16:0/16:0)	LMGP04010986

MxP® Quant 500 XL kit lipid annotation	Potential isobars within ± 0.5 Da	Potential isomers	Data base ID
PG 16:0_16:1	TG 16:0_26:1	TG(12:0/14:1(9Z)/16:0)[iso6]	LMGL03013240
	PG 16:0_16:1	PG(16:1(9Z)/16:0)	LMGP04010207
		PG(16:0/16:1(9Z))	LMGP04010910
		PG(15:0/16:0(9Cp))	LMGP04010992
	PA 16:0_22:6	PA(16:0/22:6(4Z,7Z,10Z,13Z,16Z,19Z))	LMGP10010038
		PA(22:6(4Z,7Z,10Z,13Z,16Z,19Z)/16:0)	LMGP10010845
	As-PL 16:0/0:0	As-PL(16:0/0:0)	LMGP14040001
PG 16:0_18:1	TG 18:1_26:0	TG(13:0/13:0/18:1(9Z))[iso3]	LMGL03012701
		TG(12:0/14:0/18:1(9Z))[iso6]	LMGL03013217
	PG 16:0_18:1	PG(16:0/18:1(9Z))	LMGP04010002
		PG(16:0/18:1(11Z))	LMGP04010008
		PG(18:1(9Z)/16:0)	LMGP04010962
		PG(16:0/18:1(9Z))	LMGP04010987
	LBPA 16:0_18:1	LBPA(16:0/18:1(9Z))	LMGP04100002
PG 16:0_18:2	TG 16:0_28:2	TG(14:1(9Z)/14:1(9Z)/16:0)[iso3]	LMGL03012815
	PG 16:0_18:2	PG(18:2(9Z,12Z)/16:0)	LMGP04010877
		PG(16:0/18:2(9Z,12Z))	LMGP04010983
PG 16:0_18:3	PG 16:0_18:3	PG(18:3(6Z,9Z,12Z)/16:0)	LMGP04010372
		PG(16:0/18:3(6Z,9Z,12Z))	LMGP04010906
		PG(18:3(9Z,12Z,15Z)/16:0)	LMGP04010955
		PG(16:0/18:3(9Z,12Z,15Z))	LMGP04010973
PG 16:0_19:1	TG 16:0_29:1	TG(12:0/16:0/17:1(9Z))[iso6]	LMGL03013324
		TG(13:0/16:0/16:1(9Z))[iso6]	LMGL03013786
		TG(14:0/15:1(9Z)/16:0)[iso6]	LMGL03014194
		TG(14:1(9Z)/15:0/16:0)[iso6]	LMGL03014573
	PG 16:0_19:1	PG(16:0/19:1(9Z))	LMGP04010196
		PG(19:1(9Z)/16:0)	LMGP04010486
		PG(16:0/18:0(11Cp))	LMGP04010989
	PG P-20:0/16:0	PG(P-20:0/16:0)	LMGP04030062
PG 16:0_20:3	TG 16:0_30:3	TG(12:0/16:0/18:3(6Z,9Z,12Z))[iso6]	LMGL03013328
		TG(12:0/16:0/18:3(9Z,12Z,15Z))[iso6]	LMGL03013329
	PG 16:0_20:3	PG(20:3(8Z,11Z,14Z)/16:0)	LMGP04010595
		PG(16:0/20:3(8Z,11Z,14Z))	LMGP04010904
PG 16:0_20:4	TG 16:0_30:4	TG(12:0/16:0/18:4(6Z,9Z,12Z,15Z))[iso6]	LMGL03013330
	GlcADG 18:1_16:0	alpha-GlcADAG(18:1/16:0)	LMGL05010032
	PG 16:0_20:4	PG(16:0/20:4(5Z,8Z,11Z,14Z))	LMGP04010036
		PG(20:4(5Z,8Z,11Z,14Z)/16:0)	LMGP04010859
PG 16:0_20:5	PG 36:5	PG(20:5(5Z,8Z,11Z,14Z,17Z)/16:0)	LMGP04010654
		PG(16:0/20:5(5Z,8Z,11Z,14Z,17Z))	LMGP04010903
	PI 29:0	PI(13:0/16:0)	LMGP06010040
		PI(16:0/13:0)	LMGP06010160

MxP® Quant 500 XL kit lipid annotation	Potential isobars within ± 0.5 Da	Potential isomers	Data base ID
PG 16:0_22:1	TG 16:0_32:1	TG(16:0/16:0/16:1(9Z))[iso3]	LMGL03010017
		TG(12:0/16:0/20:1(11Z))[iso6]	LMGL03013334
		TG(13:0/16:0/19:1(9Z))[iso6]	LMGL03013797
		TG(14:0/16:0/18:1(9Z))[iso6]	LMGL03014226
		TG(14:1(9Z)/16:0/18:0)[iso6]	LMGL03014631
		TG(15:0/16:0/17:1(9Z))[iso6]	LMGL03015007
		TG(15:1(9Z)/16:0/17:0)[iso6]	LMGL03015357
	PG 16:0_22:1	PG(16:0/22:1(11Z))	LMGP04010199
		PG(22:1(11Z)/16:0)	LMGP04010731
PG 16:0_22:2	TG 16:0_32:2	TG(16:0/16:1(9Z)/16:1(9Z))[iso3]	LMGL03010018
		TG(12:0/16:0/20:2(11Z,14Z))[iso6]	LMGL03013335
		TG(14:0/16:0/18:2(9Z,12Z))[iso6]	LMGL03014227
		TG(14:1(9Z)/16:0/18:1(9Z))[iso6]	LMGL03014632
		TG(15:0/16:0/17:2(9Z,12Z))[iso6]	LMGL03015008
		TG(15:1(9Z)/16:0/17:1(9Z))[iso6]	LMGL03015358
	PG 16:0_22:2	PG(16:0/22:2(13Z,16Z))	LMGP04010200
		PG(22:2(13Z,16Z)/16:0)	LMGP04010762
PG 16:1_16:1	TG 16:1_26:1	TG(12:0/14:1(9Z)/16:1(9Z))[iso6]	LMGL03013241
	PG 16:1_16:1	PG(16:1(9Z)/16:1(9Z))	LMGP04010967
	PA 16:1_22:6	PA(22:6(4Z,7Z,10Z,13Z,16Z,19Z)/16:1(9Z))	LMGP10010822
		PA(16:1(9Z)/22:6(4Z,7Z,10Z,13Z,16Z,19Z))	LMGP10010899
PG 16:1_18:0	TG 18:0_26:1	TG(12:0/14:1(9Z)/18:0)[iso6]	LMGL03013245
	PG 16:1_18:0	PG(16:1(9Z)/18:0)	LMGP04010211
		PG(18:0/16:1(9Z))	LMGP04010887
	PA 18:0_22:6	PA(18:0/22:6(4Z,7Z,10Z,13Z,16Z,19Z))	LMGP10010039
		PA(22:6(4Z,7Z,10Z,13Z,16Z,19Z)/18:0)	LMGP10010826
PG 16:1_18:1	TG 18:1_26:1	TG(12:0/14:1(9Z)/18:1(9Z))[iso6]	LMGL03013246
	PG 16:1_18:1	PG(18:1(9Z)/16:1(9Z))	LMGP04010881
		PG(16:1(9Z)/18:1(9Z))	LMGP04010902
	LBPA 16:1_18_1	LBPA(16:1(9Z)/18:1(9Z))	LMGP04100004
	PA 18:1_22:6	PA(18:1(9Z)/22:6(4Z,7Z,10Z,13Z,16Z,19Z))	LMGP10010040
		PA(22:6(4Z,7Z,10Z,13Z,16Z,19Z)/18:1(9Z))	LMGP10010827
PG 16:1_18:2	TG 18:2_26:1	TG(12:0/14:1(9Z)/18:2(9Z,12Z))[iso6]	LMGL03013247
	PG 16:1_18:2	PG(18:2(9Z,12Z)/16:1(9Z))	LMGP04010346
		PG(16:1(9Z)/18:2(9Z,12Z))	LMGP04010901
	PA 18:2_22:6	PA(22:6(4Z,7Z,10Z,13Z,16Z,19Z)/18:2(9Z,12Z))	LMGP10010828
		PA(18:2(9Z,12Z)/22:6(4Z,7Z,10Z,13Z,16Z,19Z))	LMGP10010874

MxP® Quant 500 XL kit lipid annotation	Potential isobars within ± 0.5 Da	Potential isomers	Data base ID
PG 16:1_20:4	TG 20:4_26:1	TG(12:0/14:1(9Z)/20:4(5Z,8Z,11Z,14Z))[iso6]	LMGL03013257
	PG 16:1_20:4	PG(20:4(5Z,8Z,11Z,14Z)/16:1(9Z))	LMGP04010626
		PG(16:1(9Z)/20:4(5Z,8Z,11Z,14Z))	LMGP04010899
	PA 20:4_22:6	PA(20:4(5Z,8Z,11Z,14Z)/22:6(4Z,7Z,10Z,13Z,16Z,19Z))	LMGP10010646
		PA(22:6(4Z,7Z,10Z,13Z,16Z,19Z)/20:4(5Z,8Z,11Z,14Z))	LMGP10010838
PG 16:1_22:1	TG 16:1_32:1	TG(16:0/16:1(9Z)/16:1(9Z))[iso3]	LMGL03010018
		TG(12:0/16:1(9Z)/20:1(11Z))[iso6]	LMGL03013359
		TG(13:0/16:1(9Z)/19:1(9Z))[iso6]	LMGL03013822
		TG(14:0/16:1(9Z)/18:1(9Z))[iso6]	LMGL03014251
		TG(14:1(9Z)/16:1(9Z)/18:0)[iso6]	LMGL03014656
		TG(15:0/16:1(9Z)/17:1(9Z))[iso6]	LMGL03015032
		TG(15:1(9Z)/16:1(9Z)/17:0)[iso6]	LMGL03015382
	PG 16:1_22:1	PG(16:1(9Z)/22:1(11Z))	LMGP04010223
		PG(22:1(11Z)/16:1(9Z))	LMGP04010732
PG 16:2_18:1	PG 16:2_18:1	PA(18:1(9Z)/21:0)	LMGP10010334
		PA(21:0/18:1(9Z))	LMGP10010684
	[3-[[(2S)-2,3-dihydroxypropoxy]-hydroxyphosphoryl]oxy-2-[(4E,7E)-hexadeca-4,7-dienoyl]oxypropyl] (E)-octadec-11-enoate	[3-[[(2S)-2,3-dihydroxypropoxy]-hydroxyphosphoryl]oxy-2-[(4E,7E)-hexadeca-4,7-dienoyl]oxypropyl] (E)-octadec-11-enoate	PubChem:134724191
PG 16:2_18:2	PG 16:2_18:2	PA(18:2(9Z,12Z)/21:0)	LMGP10010359
		PA(21:0/18:2(9Z,12Z))	LMGP10010685
	[(2R)-1-[[(2S)-2,3-dihydroxypropoxy]-hydroxyphosphoryl]oxy-3-dodecanoyloxypropan-2-yl] (7Z,10Z,13Z,16Z)-docosa-7,10,13,16-tetraenoate	[(2R)-1-[[(2S)-2,3-dihydroxypropoxy]-hydroxyphosphoryl]oxy-3-dodecanoyloxypropan-2-yl] (7Z,10Z,13Z,16Z)-docosa-7,10,13,16-tetraenoate	PubChem:52926332
PG 16:3_18:1	PG 16:3_18:1	-	-
PG 17:0_18:1	TG 18:1_27:0	TG(12:0/15:0/18:1(9Z))[iso6]	LMGL03013274
		TG(13:0/14:0/18:1(9Z))[iso6]	LMGL03013681
	PG 17:0_18:1	PG(18:1(9Z)/17:0)	LMGP04010327
		PG(17:0/18:1(9Z))	LMGP04010893
	PG O-18:0/18:1	PG(O-18:0/18:1(9Z))	LMGP04020027
PG 17:0_18:2	TG 18:2_27:0	TG(12:0/15:0/18:2(9Z,12Z))[iso6]	LMGL03013275
		TG(13:0/14:0/18:2(9Z,12Z))[iso6]	LMGL03013682
	PG 17:0_18:2	PG(17:0/18:2(9Z,12Z))	LMGP04010233
		PG(18:2(9Z,12Z)/17:0)	LMGP04010347
	PG O-18:0/18:2	PG(O-18:0/18:2(9Z,12Z))	LMGP04020028

MxP® Quant 500 XL kit lipid annotation	Potential isobars within ± 0.5 Da	Potential isomers	Data base ID
PG 17:1_18:1	TG 18:1_27:1	TG(12:0/15:1(9Z)/18:1(9Z))[iso6]	LMGL03013301
		TG(13:0/14:1(9Z)/18:1(9Z))[iso6]	LMGL03013710
	PG 17:1_18:1	PG(17:1(9Z)/18:1(9Z))	LMGP04010259
		PG(18:1(9Z)/17:1(9Z))	LMGP04010328
	PG P-18:0/18:1	PG(P-18:0/18:1(9Z))	LMGP04030091
	LPIM1 18:1	LPIM1(18:1(9Z)/0:0)	LMGP15040004
PG 18:0_18:1	TG 18:1_28:0	TG(14:0/14:0/18:1(9Z))[iso3]	LMGL03012762
		TG(12:0/16:0/18:1(9Z))[iso6]	LMGL03013326
		TG(13:0/15:0/18:1(9Z))[iso6]	LMGL03013738
	PG 18:0_18:1	PG(18:0/18:1(9Z))	LMGP04010037
		PG(18:1(9Z)/18:0)	LMGP04010961
PG 18:0_18:2	TG 18:2_28:0	TG(14:0/14:0/18:2(9Z,12Z))[iso3]	LMGL03012763
		TG(12:0/16:0/18:2(9Z,12Z))[iso6]	LMGL03013327
		TG(13:0/15:0/18:2(9Z,12Z))[iso6]	LMGL03013739
	PG 18:0_18:2	PG(18:2(9Z,12Z)/18:0)	LMGP04010876
		PG(18:0/18:2(9Z,12Z))	LMGP04010982
PG 18:0_18:3	PG 18:0_18:3	PG(18:0/18:3(6Z,9Z,12Z))	LMGP04010315
		PG(18:0/18:3(9Z,12Z,15Z))	LMGP04010316
		PG(18:3(6Z,9Z,12Z)/18:0)	LMGP04010377
		PG(18:3(9Z,12Z,15Z)/18:0)	LMGP04010407
PG 18:0_22:1	TG 18:0_32:1	TG(16:0/16:1(9Z)/18:0)[iso6]	LMGL03010036
		TG(14:1(9Z)/18:0/18:0)[iso3]	LMGL03012849
		TG(12:0/18:0/20:1(11Z))[iso6]	LMGL03013449
		TG(13:0/18:0/19:1(9Z))[iso6]	LMGL03013912
		TG(14:0/18:0/18:1(9Z))[iso6]	LMGL03014341
		TG(15:0/17:1(9Z)/18:0)[iso6]	LMGL03015081
		TG(15:1(9Z)/17:0/18:0)[iso6]	LMGL03015409
	PG 18:0_22:1	PG(18:0/22:1(11Z))	LMGP04010320
		PG(22:1(11Z)/18:0)	LMGP04010736
PG 18:1_18:1	TG 18:1_28:1	TG(12:0/16:1(9Z)/18:1(9Z))[iso6]	LMGL03013351
		TG(13:0/15:1(9Z)/18:1(9Z))[iso6]	LMGL03013765
		TG(14:0/14:1(9Z)/18:1(9Z))[iso6]	LMGL03014145
	PG 18:1_18:1	PG(18:1(9E)/18:1(9E))	LMGP04010033
		PG(18:1(9Z)/18:1(9Z))	LMGP04010985
	LBPA 36:2	LBPA(18:1(9Z)/18:1(9Z))	LMGP04100003
PG 18:1_18:2	TG 18:2_28:1	TG(12:0/16:1(9Z)/18:2(9Z,12Z))[iso6]	LMGL03013352
		TG(13:0/15:1(9Z)/18:2(9Z,12Z))[iso6]	LMGL03013766
		TG(14:0/14:1(9Z)/18:2(9Z,12Z))[iso6]	LMGL03014146
	PG 18:1_18:2	PG(18:2(9Z,12Z)/18:1(9Z))	LMGP04010350
		PG(18:1(9Z)/18:2(9Z,12Z))	LMGP04010960
PG 18:1_18:3	GlcADG 18:1_16:0	alpha-GlcADAG(18:1/16:0)	LMGL05010032
	PG 18:1_18:3	PG(18:1(9Z)/18:3(6Z,9Z,12Z))	LMGP04010330
		PG(18:3(6Z,9Z,12Z)/18:1(9Z))	LMGP04010378
		PG(18:3(9Z,12Z,15Z)/18:1(9Z))	LMGP04010874
		PG(18:1(9Z)/18:3(9Z,12Z,15Z))	LMGP04010880

MxP® Quant 500 XL kit lipid annotation	Potential isobars within ± 0.5 Da	Potential isomers	Data base ID
PG 18:1_20:0	TG 18:1_30:0	TG(15:0/15:0/18:1(9Z))[iso3]	LMGL03012878
		TG(12:0/18:0/18:1(9Z))[iso6]	LMGL03013441
		TG(13:0/17:0/18:1(9Z))[iso6]	LMGL03013840
		TG(14:0/16:0/18:1(9Z))[iso6]	LMGL03014226
	PG 18:1_20:0	PG(20:0/18:1(9Z))	LMGP04010952
		PG(18:1(9Z)/20:0)	LMGP04010959
PG 18:1_20:1	TG 18:1_30:1	TG(12:0/18:1(9Z)/18:1(9Z))[iso3]	LMGL03012670
		TG(13:0/17:1(9Z)/18:1(9Z))[iso6]	LMGL03013863
		TG(14:0/16:1(9Z)/18:1(9Z))[iso6]	LMGL03014251
		TG(14:1(9Z)/16:0/18:1(9Z))[iso6]	LMGL03014632
		TG(15:0/15:1(9Z)/18:1(9Z))[iso6]	LMGL03014984
	PG 18:1_20:1	PG(20:1(11Z)/18:1(9Z))	LMGP04010540
		PG(18:1(9Z)/20:1(11Z))	LMGP04010958
PG 18:1_20:2	TG 18:1_30:2	TG(15:1(9Z)/15:1(9Z)/18:1(9Z))[iso3]	LMGL03012933
		TG(12:0/18:1(9Z)/18:2(9Z,12Z))[iso6]	LMGL03013462
		TG(13:0/17:2(9Z,12Z)/18:1(9Z))[iso6]	LMGL03013885
		TG(14:1(9Z)/16:1(9Z)/18:1(9Z))[iso6]	LMGL03014657
	PG 18:1_20:2	PG(18:1(9Z)/20:2(11Z,14Z))	LMGP04010334
		PG(20:2(11Z,14Z)/18:1(9Z))	LMGP04010570
PG 18:1_20:3	TG 18:1_30:3	TG(12:0/18:1(9Z)/18:3(6Z,9Z,12Z))[iso6]	LMGL03013463
		TG(12:0/18:1(9Z)/18:3(9Z,12Z,15Z))[iso6]	LMGL03013464
	PG 18:1_20:3	PG(18:1(9Z)/20:3(8Z,11Z,14Z))	LMGP04010335
		PG(20:3(8Z,11Z,14Z)/18:1(9Z))	LMGP04010601
PG 18:1_20:4	TG 18:1_30:4	TG(12:0/18:1(9Z)/18:4(6Z,9Z,12Z,15Z))[iso6]	LMGL03013465
	PG 18:1_20:4	PG(20:4(5Z,8Z,11Z,14Z)/18:1(9Z))	LMGP04010631
		PG(18:1(9Z)/20:4(5Z,8Z,11Z,14Z))	LMGP04010879
PG 18:1_20:5	PG 18:1_20:5	PG(20:5(5Z,8Z,11Z,14Z,17Z)/18:1(9Z))	LMGP04010857
		PG(18:1(9Z)/20:5(5Z,8Z,11Z,14Z,17Z))	LMGP04010878
	PI 18:1:13:0	PI(13:0/18:1(9Z))	LMGP06010045
		PI(18:1(9Z)/13:0)	LMGP06010291
PG 18:1_22:0	TG 18:1_32:0	TG(16:0/16:0/18:1(11E))	LMGL03010005
		TG(16:0/16:0/18:1(9Z))	LMGL03010006
		TG(12:0/18:1(9Z)/20:0)[iso6]	LMGL03013468
		TG(13:0/18:1(9Z)/19:0)[iso6]	LMGL03013931
		TG(14:0/18:0/18:1(9Z))[iso6]	LMGL03014341
		TG(15:0/17:0/18:1(9Z))[iso6]	LMGL03015059
	PG 18:1_22:0	PG(22:0/18:1(9Z))	LMGP04010946
		PG(18:1(9Z)/22:0)	LMGP04010957

MxP® Quant 500 XL kit lipid annotation	Potential isobars within ± 0.5 Da	Potential isomers	Data base ID
PG 18:1_22:1	TG 18:1_32:1	TG(16:0/16:1(9Z)/18:1(9Z))[iso6]	LMGL03010043
		TG(14:0/18:1(9Z)/18:1(9Z))[iso3]	LMGL03012792
		TG(12:0/18:1(9Z)/20:1(11Z))[iso6]	LMGL03013469
		TG(13:0/18:1(9Z)/19:1(9Z))[iso6]	LMGL03013932
		TG(14:1(9Z)/18:0/18:1(9Z))[iso6]	LMGL03014747
		TG(15:0/17:1(9Z)/18:1(9Z))[iso6]	LMGL03015082
		TG(15:1(9Z)/17:0/18:1(9Z))[iso6]	LMGL03015410
	PG 18:1_22:1	PG(18:1(9Z)/22:1(11Z))	LMGP04010337
		PG(22:1(11Z)/18:1(9Z))	LMGP04010737
PG 18:1_22:2	TG 18:1_32:2	TG(16:1(9Z)/16:1(9Z)/18:1(9Z))[iso3]	LMGL03010052
		TG(14:1(9Z)/18:1(9Z)/18:1(9Z))[iso3]	LMGL03012850
		TG(12:0/18:1(9Z)/20:2(11Z,14Z))[iso6]	LMGL03013470
		TG(14:0/18:1(9Z)/18:2(9Z,12Z))[iso6]	LMGL03014362
		TG(15:0/17:2(9Z,12Z)/18:1(9Z))[iso6]	LMGL03015104
		TG(15:1(9Z)/17:1(9Z)/18:1(9Z))[iso6]	LMGL03015433
	PG 18:1_22:2	PG(18:1(9Z)/22:2(13Z,16Z))	LMGP04010338
		PG(22:2(13Z,16Z)/18:1(9Z))	LMGP04010768
	PGP 16:0_18:1	PGP(16:0/18:1(9Z))	LMGP05010001
		PGP(16:0/18:1(11Z))	LMGP05010002
PG 18:1_22:3	TG 18:1_32:3	TG(12:0/18:1(9Z)/20:3(8Z,11Z,14Z))[iso6]	LMGL03013471
		TG(14:0/18:1(9Z)/18:3(6Z,9Z,12Z))[iso6]	LMGL03014363
		TG(14:0/18:1(9Z)/18:3(9Z,12Z,15Z))[iso6]	LMGL03014364
		TG(14:1(9Z)/18:1(9Z)/18:2(9Z,12Z))[iso6]	LMGL03014768
		TG(15:1(9Z)/17:2(9Z,12Z)/18:1(9Z))[iso6]	LMGL03015455
	18:1-Glc-Campesterol	18:1-Glc-Campesterol	LMST01031134
PG 18:1_22:4	TG 18:1_32:4	TG(12:0/18:1(9Z)/20:4(5Z,8Z,11Z,14Z))[iso6]	LMGL03013472
		TG(14:0/18:1(9Z)/18:4(6Z,9Z,12Z,15Z))[iso6]	LMGL03014365
		TG(14:1(9Z)/18:1(9Z)/18:3(6Z,9Z,12Z))[iso6]	LMGL03014769
		TG(14:1(9Z)/18:1(9Z)/18:3(9Z,12Z,15Z))[iso6]	LMGL03014770
	PG 18:1_22:4	PG(18:1(9Z)/22:4(7Z,10Z,13Z,16Z))	LMGP04010339
		PG(22:4(7Z,10Z,13Z,16Z)/18:1(9Z))	LMGP04010799

MxP® Quant 500 XL kit lipid annotation	Potential isobars within ± 0.5 Da	Potential isomers	Data base ID
PG 18:1_22:5	TG 18:1_32:5	TG(12:0/18:1(9Z)/20:5(5Z,8Z,11Z,14Z,17Z))[iso6]	LMGL03013473
		TG(14:1(9Z)/18:1(9Z)/18:4(6Z,9Z,12Z,15Z))[iso6]	LMGL03014771
	PI 15:0_18:1	PI(15:0/18:1(9Z))	LMGP06010113
		PI(18:1(9Z)/15:0)	LMGP06010293
	PI O-34:1	PI(O-16:0/18:1(9Z))	LMGP06020093
	PG 18:1_22:5	PG(18:1(11Z)/22:5(4Z,7Z,10Z,13Z,16Z))	PubChem:53480626
		PG(18:1(9Z)/22:5(4Z,7Z,10Z,13Z,16Z))	PubChem:53480631
		PG(18:1(11Z)/22:5(7Z,10Z,13Z,16Z,19Z))	PubChem:53480627
		PG(18:1(9Z)/22:5(7Z,10Z,13Z,16Z,19Z))	PubChem:53480632
PG 18:2_18:2	TG 46:4	TG(14:1(9Z)/14:1(9Z)/18:2(9Z,12Z))[iso3]	LMGL03012822
	PG 36:4	PG(18:2(9Z,12Z)/18:2(9Z,12Z))	LMGP04010956
PG 18:2_18:3	PG 36:5	PG(18:2(9Z,12Z)/18:3(6Z,9Z,12Z))	LMGP04010351
		PG(18:2(9Z,12Z)/18:3(9Z,12Z,15Z))	LMGP04010352
		PG(18:3(6Z,9Z,12Z)/18:2(9Z,12Z))	LMGP04010379
		PG(18:3(9Z,12Z,15Z)/18:2(9Z,12Z))	LMGP04010408
PG 18:2_18:4	PG 18:2_18:4	PG(18:2(9Z,12Z)/18:4(6Z,9Z,12Z,15Z))	LMGP04010353
		PG(18:4(6Z,9Z,12Z,15Z)/18:2(9Z,12Z))	LMGP04010438
PG 18:2_20:0	TG 18:2_30:0	TG(15:0/15:0/18:2(9Z,12Z))[iso3]	LMGL03012879
		TG(12:0/18:0/18:2(9Z,12Z))[iso6]	LMGL03013442
		TG(13:0/17:0/18:2(9Z,12Z))[iso6]	LMGL03013841
		TG(14:0/16:0/18:2(9Z,12Z))[iso6]	LMGL03014227
	PG 18:2_20:0	PG(18:2(9Z,12Z)/20:0)	LMGP04010356
		PG(20:0/18:2(9Z,12Z))	LMGP04010951
PG 18:2_20:2	TG 18:2_30:2	TG(12:0/18:2(9Z,12Z)/18:2(9Z,12Z))[iso3]	LMGL03012671
		TG(15:1(9Z)/15:1(9Z)/18:2(9Z,12Z))[iso3]	LMGL03012934
		TG(13:0/17:2(9Z,12Z)/18:2(9Z,12Z))[iso6]	LMGL03013886
		TG(14:1(9Z)/16:1(9Z)/18:2(9Z,12Z))[iso6]	LMGL03014658
	PG 18:2_20:2	PG(18:2(9Z,12Z)/20:2(11Z,14Z))	LMGP04010358
		PG(20:2(11Z,14Z)/18:2(9Z,12Z))	LMGP04010571
PG 18:2_20:3	TG 18:2_30:3	TG(12:0/18:2(9Z,12Z)/18:3(6Z,9Z,12Z))[iso6]	LMGL03013482
		TG(12:0/18:2(9Z,12Z)/18:3(9Z,12Z,15Z))[iso6]	LMGL03013483
		PG(18:2(9Z,12Z)/20:3(8Z,11Z,14Z))	LMGP04010359
		PG(20:3(8Z,11Z,14Z)/18:2(9Z,12Z))	LMGP04010602

MxP® Quant 500 XL kit lipid annotation	Potential isobars within ± 0.5 Da	Potential isomers	Data base ID
PG 18:2_20:4	TG 18:2_30:4	TG(12:0/18:2(9Z,12Z)/18:4(6Z,9Z,12Z,15Z))[iso6]	LMGL03013484
	PG 18:2_20:4	PG(20:4(5Z,8Z,11Z,14Z)/18:2(9Z,12Z))	LMGP04010632
		PG(18:2(9Z,12Z)/20:4(5Z,8Z,11Z,14Z))	LMGP04010875
PG 18:2_20:5	PG 18:2_20:5	PG(18:2(9Z,12Z)/20:5(5Z,8Z,11Z,14Z,17Z))	LMGP04010360
		PG(20:5(5Z,8Z,11Z,14Z,17Z)/18:2(9Z,12Z))	LMGP04010660
	PI 18:2_13:0	PI(13:0/18:2(9Z,12Z))	LMGP06010046
		PI(18:2(9Z,12Z)/13:0)	LMGP06010309
PG 18:2_22:0	TG 18:2_32:0	TG(16:0/16:0/18:2(9Z,12Z))[iso3]	LMGL03010044
		TG(12:0/18:2(9Z,12Z)/20:0)[iso6]	LMGL03013487
		TG(13:0/18:2(9Z,12Z)/19:0)[iso6]	LMGL03013950
		TG(14:0/18:0/18:2(9Z,12Z))[iso6]	LMGL03014342
		TG(15:0/17:0/18:2(9Z,12Z))[iso6]	LMGL03015060
	PG 18:2_22:0	PG(18:2(9Z,12Z)/22:0)	LMGP04010362
		PG(22:0/18:2(9Z,12Z))	LMGP04010710
PG 18:2_22:1	TG 18:2_32:1	TG(16:0/16:1(9Z)/18:2(9Z,12Z))[iso6]	LMGL03010053
		TG(12:0/18:2(9Z,12Z)/20:1(11Z))[iso6]	LMGL03013488
		TG(13:0/18:2(9Z,12Z)/19:1(9Z))[iso6]	LMGL03013951
		TG(14:0/18:1(9Z)/18:2(9Z,12Z))[iso6]	LMGL03014362
		TG(14:1(9Z)/18:0/18:2(9Z,12Z))[iso6]	LMGL03014748
		TG(15:0/17:1(9Z)/18:2(9Z,12Z))[iso6]	LMGL03015083
		TG(15:1(9Z)/17:0/18:2(9Z,12Z))[iso6]	LMGL03015411
	PG 18:2_22:1	PG(18:2(9Z,12Z)/22:1(11Z))	LMGP04010363
		PG(22:1(11Z)/18:2(9Z,12Z))	LMGP04010738
PG 18:2_22:3	TG 18:2_32:3	TG(14:1(9Z)/18:2(9Z,12Z)/18:2(9Z,12Z))[iso3]	LMGL03012851
		TG(12:0/18:2(9Z,12Z)/20:3(8Z,11Z,14Z))[iso6]	LMGL03013490
		TG(14:0/18:2(9Z,12Z)/18:3(6Z,9Z,12Z))[iso6]	LMGL03014382
		TG(14:0/18:2(9Z,12Z)/18:3(9Z,12Z,15Z))[iso6]	LMGL03014383
		TG(15:1(9Z)/17:2(9Z,12Z)/18:2(9Z,12Z))[iso6]	LMGL03015456
	18:2-Glc-Campesterol	18:2-Glc-Campesterol	LMST01031135

MxP® Quant 500 XL kit lipid annotation	Potential isobars within ± 0.5 Da	Potential isomers	Data base ID
PG 18:2_22:4	TG 18:2_32:4	TG(12:0/18:2(9Z,12Z)/20:4(5Z,8Z,11Z,14Z))[iso6]	LMGL03013491
		TG(14:0/18:2(9Z,12Z)/18:4(6Z,9Z,12Z,15Z))[iso6]	LMGL03014384
		TG(14:1(9Z)/18:2(9Z,12Z)/18:3(6Z,9Z,12Z))[iso6]	LMGL03014788
		TG(14:1(9Z)/18:2(9Z,12Z)/18:3(9Z,12Z,15Z))[iso6]	LMGL03014789
	PG 18:2_22:4	PG(18:2(9Z,12Z)/22:4(7Z,10Z,13Z,16Z))	LMGP04010365
		PG(22:4(7Z,10Z,13Z,16Z)/18:2(9Z,12Z))	LMGP04010800
PG 20:3_20:4	TG 20:4_30:3	TG(12:0/18:3(6Z,9Z,12Z)/20:4(5Z,8Z,11Z,14Z))[iso6]	LMGL03013509
		TG(12:0/18:3(9Z,12Z,15Z)/20:4(5Z,8Z,11Z,14Z))[iso6]	LMGL03013526
	PG 20:3_20:4	PG(20:3(8Z,11Z,14Z)/20:4(5Z,8Z,11Z,14Z))	LMGP04010612
		PG(20:4(5Z,8Z,11Z,14Z)/20:3(8Z,11Z,14Z))	LMGP04010641
PG 20:4_20:4	TG 20:4_30:4	TG(12:0/18:4(6Z,9Z,12Z,15Z)/20:4(5Z,8Z,11Z,14Z))[iso6]	LMGL03013542
	PG 20:4_20:4	PG(20:4(5Z,8Z,11Z,14Z)/20:4(5Z,8Z,11Z,14Z))	LMGP04010858
	LBPA 20:4_20:4	LBPA(20:4(5Z,8Z,11Z,14Z)/20:4(5Z,8Z,11Z,14Z))	LMGP04100001
PG 20:4_22:1	TG 20:4_32:1	TG(16:0/16:1(9Z)/20:4(5Z,8Z,11Z,14Z))[iso6]	LMGL03010185
		TG(12:0/20:1(11Z)/20:4(5Z,8Z,11Z,14Z))[iso6]	LMGL03013596
		TG(13:0/19:1(9Z)/20:4(5Z,8Z,11Z,14Z))[iso6]	LMGL03014036
		TG(14:0/18:1(9Z)/20:4(5Z,8Z,11Z,14Z))[iso6]	LMGL03014372
		TG(14:1(9Z)/18:0/20:4(5Z,8Z,11Z,14Z))[iso6]	LMGL03014758
		TG(15:0/17:1(9Z)/20:4(5Z,8Z,11Z,14Z))[iso6]	LMGL03015093
		TG(15:1(9Z)/17:0/20:4(5Z,8Z,11Z,14Z))[iso6]	LMGL03015421
	PG 20:4_22:1	PG(20:4(5Z,8Z,11Z,14Z)/22:1(11Z))	LMGP04010645
		PG(22:1(11Z)/20:4(5Z,8Z,11Z,14Z))	LMGP04010748
PG 20:4_22:3	TG 20:4_32:3	TG(12:0/20:3(8Z,11Z,14Z)/20:4(5Z,8Z,11Z,14Z))[iso6]	LMGL03013617
		TG(14:0/18:3(6Z,9Z,12Z)/20:4(5Z,8Z,11Z,14Z))[iso6]	LMGL03014409
		TG(14:0/18:3(9Z,12Z,15Z)/20:4(5Z,8Z,11Z,14Z))[iso6]	LMGL03014426
		TG(14:1(9Z)/18:2(9Z,12Z)/20:4(5Z,8Z,11Z,14Z))[iso6]	LMGL03014797
		TG(15:1(9Z)/17:2(9Z,12Z)/20:4(5Z,8Z,11Z,14Z))[iso6]	LMGL03015466

MxP® Quant 500 XL kit lipid annotation	Potential isobars within ± 0.5 Da	Potential isomers	Data base ID
PG 20:4_22:4	TG 20:4_32:4	TG(12:0/20:4(5Z,8Z,11Z,14Z)/20:4(5Z,8Z,11Z,14Z))[iso3]	LMGL03012681
		TG(14:0/18:4(6Z,9Z,12Z,15Z)/20:4(5Z,8Z,11Z,14Z))[iso6]	LMGL03014442
		TG(14:1(9Z)/18:3(6Z,9Z,12Z)/20:4(5Z,8Z,11Z,14Z))[iso6]	LMGL03014815
		TG(14:1(9Z)/18:3(9Z,12Z,15Z)/20:4(5Z,8Z,11Z,14Z))[iso6]	LMGL03014832
	PG 20:4_22:4	PG(20:4(5Z,8Z,11Z,14Z)/22:4(7Z,10Z,13Z,16Z))	LMGP04010647
		PG(22:4(7Z,10Z,13Z,16Z)/20:4(5Z,8Z,11Z,14Z))	LMGP04010810
PG 22:4_22:6	PG 22:4_22:6	PG(22:6(4Z,7Z,10Z,13Z,16Z,19Z)/22:4(7Z,10Z,13Z,16Z))	LMGP04010845
		PG(22:4(7Z,10Z,13Z,16Z)/22:6(4Z,7Z,10Z,13Z,16Z,19Z))	LMGP04010978
	PI 15:1_22:4	PI(15:1(9Z)/22:4(7Z,10Z,13Z,16Z))	LMGP06010158
		PI(22:4(7Z,10Z,13Z,16Z)/15:1(9Z))	LMGP06010761
	PI P-16:0/22:4	PI(P-16:0/22:4(7Z,10Z,13Z,16Z))	LMGP06030028
PG 22:5_22:6	PG 22:5_22:6	PG(22:5/22:6)	PubChem:154573539
PG 22:6_22:6	PG 22:6_22:6	PG(22:6(4Z,7Z,10Z,13Z,16Z,19Z)/22:6(4Z,7Z,10Z,13Z,16Z,19Z))	LMGP04010977
	PI 15:1_22:6	PI(15:1(9Z)/22:6(4Z,7Z,10Z,13Z,16Z,19Z))	LMGP06010159
		PI(22:6(4Z,7Z,10Z,13Z,16Z,19Z)/15:1(9Z))	LMGP06010791
	PI P-16:0/22:6	PI(P-16:0/22:6(4Z,7Z,10Z,13Z,16Z,19Z))	LMGP06030093

Lysophosphatidylinositols (16)			
MxP® Quant 500 XL kit lipid annotation	Potential isobars within ± 0.5 Da	Potential isomers	Data base ID
LPI 14:0	LPI 14:0	PI(14:0/0:0)	LMGP06050008
LPI 14:1	LPI 14:1	PI(14:1(9Z)/0:0)	LMGP06050031
LPI 15:0	LPI 15:0	PI(15:0/0:0)	LMGP06050030
LPI 16:0	LPI 16:0	PI(16:0/0:0)	LMGP06050002
LPI 16:1	LPI 16:1	PI(16:1(9Z)/0:0)	LMGP06050009
LPI 17:0	LPI 17:0	PI(17:0/0:0)	LMGP06050029
LPI 17:1	LPI 17:1	PI(17:1(10Z)/0:0)	LMGP06050003
		PI(17:1(9Z)/0:0)	LMGP06050032
LPI 18:0	LPI 18:0	PI(18:0/0:0)	LMGP06050004
	Glc-GP 18:0	Glc-GP(18:0/0:0)	LMGP14040003
LPI 18:1	LPI 18:1	PI(18:1(9Z)/0:0)	LMGP06050005
LPI 18:2	LPI 18:2	PI(18:2(9Z,12Z)/0:0)	LMGP06050010
LPI 18:3	LPI 18:3	PI(18:3(6Z,9Z,12Z)/0:0)	LMGP06050016
		PI(18:3(9Z,12Z,15Z)/0:0)	LMGP06050028
LPI 19:0	LPI 19:0	PI(19:0/0:0)	LMGP06050027
LPI 20:1	LPI 20:1	PI(20:1(11Z)/0:0)	LMGP06050019
LPI 20:4	LPI 20:4	PI(20:4(5Z,8Z,11Z,14Z)/0:0)	LMGP06050006
LPI 22:0	LPI 22:0	PI(22:0/0:0)	LMGP06050024
LPI 22:1	LPI 22:1	PI(22:1(11Z)/0:0)	LMGP06050022

Phosphatidylinositols (53)			
MxP® Quant 500 XL kit lipid annotation	Potential isobars within ± 0.5 Da	Potential isomers	Data base ID
PI 14:0_18:1	PI 14:0_18:1	PI(18:1(9Z)/14:0)	LMGP06010852
		PI(14:0/18:1(9Z))	LMGP06010960
PI 14:0_18:2	PI 14:0_18:2	PI(18:2(9Z,12Z)/14:0)	LMGP06010310
		PI(14:0/18:2(9Z,12Z))	LMGP06010899
PI 15:0_16:0	TG 16:0_32:5	TG(12:0/16:0/20:5(5Z,8Z,11Z,14Z,17Z))[iso6]	LMGL03013338
		TG(14:1(9Z)/16:0/18:4(6Z,9Z,12Z,15Z))[iso6]	LMGL03014636
	PI 15:0_16:0	PI(16:0/15:0)	LMGP06010882
		PI(15:0/16:0)	LMGP06010889
PI 15:1_16:0	SQDG 16:0_16:0	SQDG(16:0/16:0)	LMGL05010004
	PG 16:0_22:6	PG(16:0/22:6(4Z,7Z,10Z,13Z,16Z,19Z))	LMGP04010041
		PG(22:6(4Z,7Z,10Z,13Z,16Z,19Z)/16:0)	LMGP04010846
	PI 15:1_16:0	PI(15:1(9Z)/16:0)	LMGP06010135
		PI(16:0/15:1(9Z))	LMGP06010162
PI 16:0_16:0	TG 16:0_33:5	TG(13:0/16:0/20:5(5Z,8Z,11Z,14Z,17Z))[iso6]	LMGL03013803
		TG(15:1(9Z)/16:0/18:4(6Z,9Z,12Z,15Z))[iso6]	LMGL03015365
	PI 16:0_16:0	PI(16:0/16:0)	LMGP06010007
PI 16:0_17:0	TG 16:0_34:5	TG(12:0/16:0/22:5(7Z,10Z,13Z,16Z,19Z))[iso6]	LMGL03013345
		TG(14:0/16:0/20:5(5Z,8Z,11Z,14Z,17Z))[iso6]	LMGL03014238
		TG(14:1(9Z)/16:0/20:4(5Z,8Z,11Z,14Z))[iso6]	LMGL03014643
		TG(16:0/16:1(9Z)/18:4(6Z,9Z,12Z,15Z))[iso6]	LMGL03015708
	PI 16:0_17:0	PI(17:0/16:0)	LMGP06010197
		PI(16:0/17:0)	LMGP06010880
	PI O-18:0/16:0	PI(O-18:0/16:0)	LMGP06020074
PI 16:0_17:1	TG 16:0_34:6	TG(12:0/16:0/22:6(4Z,7Z,10Z,13Z,16Z,19Z))[iso6]	LMGL03013346
		TG(14:1(9Z)/16:0/20:5(5Z,8Z,11Z,14Z,17Z))[iso6]	LMGL03014644
	PI 16:0_17:1	PI(17:1(9Z)/16:0)	LMGP06010222
		PI(16:0/17:1(9Z))	LMGP06010879
	PI P-18:0/16:0	PI(P-18:0/16:0)	LMGP06030035
PI 16:0_17:2	TG 16:0_33:0	TG(16:0/16:0/17:0)[iso3]	LMGL03010019
		TG(12:0/16:0/21:0)[iso6]	LMGL03013339
		TG(13:0/16:0/20:0)[iso6]	LMGL03013798
		TG(14:0/16:0/19:0)[iso6]	LMGL03014231
		TG(15:0/16:0/18:0)[iso6]	LMGL03015009
	PI 16:0_17:2	PI(16:0/17:2(9Z,12Z))	LMGP06010163
		PI(17:2(9Z,12Z)/16:0)	LMGP06010252
PI 16:0_18:1	TG 18:1_33:5	TG(13:0/18:1(9Z)/20:5(5Z,8Z,11Z,14Z,17Z))[iso6]	LMGL03013938
		TG(15:1(9Z)/18:1(9Z)/18:4(6Z,9Z,12Z,15Z))[iso6]	LMGL03015500
	PI 16:0_18:1	PI(16:0/18:1(9Z))	LMGP06010001
		PI(18:1(9Z)/16:0)	LMGP06010933

MxP® Quant 500 XL kit lipid annotation	Potential isobars within ± 0.5 Da	Potential isomers	Data base ID
PI 16:0_18:2	TG 18:2_33:5	TG(13:0/18:2(9Z,12Z)/20:5(5Z,8Z,11Z,14Z,17Z))[iso6]	LMGL03013957
		TG(15:1(9Z)/18:2(9Z,12Z)/18:4(6Z,9Z,12Z,15Z))[iso6]	LMGL03015519
	PI 16:0_18:2	PI(18:2(9Z,12Z)/16:0)	LMGP06010847
		PI(16:0/18:2(9Z,12Z))	LMGP06010959
PI 16:0_18:3	TG 16:0_34:1	TG(16:0/16:0/18:1(11E))	LMGL03010005
		TG(16:0/16:0/18:1(9Z))	LMGL03010006
		TG(16:0/17:0/17:1(9Z))[iso6]	LMGL03010028
		TG(16:0/16:1(9Z)/18:0)[iso6]	LMGL03010036
		TG(12:0/16:0/22:1(11Z))[iso6]	LMGL03013341
		TG(14:0/16:0/20:1(11Z))[iso6]	LMGL03014234
		TG(14:1(9Z)/16:0/20:0)[iso6]	LMGL03014639
		TG(15:0/16:0/19:1(9Z))[iso6]	LMGL03015016
		TG(15:1(9Z)/16:0/19:0)[iso6]	LMGL03015366
	PI 16:0_18:3	PI(18:3(6Z,9Z,12Z)/16:0)	LMGP06010340
		PI(16:0/18:3(6Z,9Z,12Z))	LMGP06010877
		PI(18:3(9Z,12Z,15Z)/16:0)	LMGP06010926
		PI(16:0/18:3(9Z,12Z,15Z))	LMGP06010944
PI 16:0_20:0	TG 16:0_37:5	TG(16:0/17:2(9Z,12Z)/20:3(8Z,11Z,14Z))[iso6]	LMGL03010241
		TG(16:0/17:1(9Z)/20:4(5Z,8Z,11Z,14Z))[iso6]	LMGL03010244
		TG(16:0/17:0/20:5(5Z,8Z,11Z,14Z,17Z))[iso6]	LMGL03010246
		TG(15:0/16:0/22:5(7Z,10Z,13Z,16Z,19Z))[iso6]	LMGL03015029
		TG(15:1(9Z)/16:0/22:4(7Z,10Z,13Z,16Z))[iso6]	LMGL03015379
		TG(16:0/18:4(6Z,9Z,12Z,15Z)/19:1(9Z))[iso6]	LMGL03015757
	PI 16:0_20:0	PI(20:0/16:0)	LMGP06010833
		PI(16:0/20:0)	LMGP06010942
PI 16:0_20:3	TG 16:0_36:1	TG(16:0/18:0/18:1(9Z))[iso6]	LMGL03010085
		TG(16:0/16:1(9Z)/20:0)[iso6]	LMGL03010095
		TG(16:0/16:0/20:1(11Z))[iso3]	LMGL03010096
		TG(16:0/17:1(9Z)/19:0)[iso6]	LMGL03010110
		TG(14:0/16:0/22:1(11Z))[iso6]	LMGL03014241
		TG(14:1(9Z)/16:0/22:0)[iso6]	LMGL03014646
		TG(15:1(9Z)/16:0/21:0)[iso6]	LMGL03015374
		TG(16:0/17:0/19:1(9Z))[iso6]	LMGL03015713
	PI 16:0_20:3	PI(20:3(8Z,11Z,14Z)/16:0)	LMGP06010563
		PI(16:0/20:3(8Z,11Z,14Z))	LMGP06010875

MxP® Quant 500 XL kit lipid annotation	Potential isobars within ± 0.5 Da	Potential isomers	Data base ID
PI 16:0_20:4	TG 16:0_36:2	TG(16:0/18:1(9Z)/18:1(9Z))[iso3]	LMGL03010100
		TG(16:0/18:0/18:2(9Z,12Z))[iso6]	LMGL03010104
		TG(16:0/16:1(9Z)/20:1(11Z))[iso6]	LMGL03010114
		TG(16:0/16:0/20:2(11Z,14Z))[iso3]	LMGL03010115
		TG(16:0/17:2(9Z,12Z)/19:0)[iso6]	LMGL03010128
		TG(14:0/16:0/22:2(13Z,16Z))[iso6]	LMGL03014242
		TG(14:1(9Z)/16:0/22:1(11Z))[iso6]	LMGL03014647
		TG(16:0/17:1(9Z)/19:1(9Z))[iso6]	LMGL03015717
	PI 16:0_20:4	PI(20:4(5Z,8Z,11Z,14Z)/16:0)	LMGP06010828
		PI(16:0/20:4(5Z,8Z,11Z,14Z))	LMGP06010958
PI 16:0_22:1	TG 16:0_39:6	TG(16:0/17:2(9Z,12Z)/22:4(7Z,10Z,13Z,16Z))[iso6]	LMGL03010643
		TG(16:0/17:1(9Z)/22:5(7Z,10Z,13Z,16Z,19Z))[iso6]	LMGL03010646
		TG(16:0/17:0/22:6(4Z,7Z,10Z,13Z,16Z,19Z))[iso6]	LMGL03010648
		TG(16:0/19:1(9Z)/20:5(5Z,8Z,11Z,14Z,17Z))[iso6]	LMGL03015779
	PI 16:0_22:1	PI(16:0/22:1(11Z))	LMGP06010167
		PI(22:1(11Z)/16:0)	LMGP06010700
PI 16:1_18:0	PI 16:1_18:0	PI(16:1(9Z)/18:0)	LMGP06010179
		PI(18:0/16:1(9Z))	LMGP06010857
PI 16:1_18:1	PI 16:1_18:1	PI(18:1(9Z)/16:1(9Z))	LMGP06010851
		PI(16:1(9Z)/18:1(9Z))	LMGP06010873
PI 16:1_18:2	PI 16:1_18:2	PI(18:2(9Z,12Z)/16:1(9Z))	LMGP06010314
		PI(16:1(9Z)/18:2(9Z,12Z))	LMGP06010872
PI 17:0_18:1	TG 18:1_34:5	TG(12:0/18:1(9Z)/22:5(7Z,10Z,13Z,16Z,19Z))[iso6]	LMGL03013480
		TG(14:0/18:1(9Z)/20:5(5Z,8Z,11Z,14Z,17Z))[iso6]	LMGL03014373
		TG(14:1(9Z)/18:1(9Z)/20:4(5Z,8Z,11Z,14Z))[iso6]	LMGL03014778
		TG(16:1(9Z)/18:1(9Z)/18:4(6Z,9Z,12Z,15Z))[iso6]	LMGL03015819
	PI 17:0_18:1	PI(18:1(9Z)/17:0)	LMGP06010295
		PI(17:0/18:1(9Z))	LMGP06010864
	PI O-18:0_18:1	PI(O-18:0/18:1(9Z))	LMGP06020028
PI 17:1_18:1	TG 18:1_34:6	TG(12:0/18:1(9Z)/22:6(4Z,7Z,10Z,13Z,16Z,19Z))[iso6]	LMGL03013481
		TG(14:1(9Z)/18:1(9Z)/20:5(5Z,8Z,11Z,14Z,17Z))[iso6]	LMGL03014779
	PI 17:1_18:1	PI(17:1(9Z)/18:1(9Z))	LMGP06010227
		PI(18:1(9Z)/17:1(9Z))	LMGP06010296
	PI P-18:0/18:1	PI(P-18:0/18:1(9Z))	LMGP06030092
PI 17:1_18:2	TG 18:2_34:6	TG(12:0/18:2(9Z,12Z)/22:6(4Z,7Z,10Z,13Z,16Z,19Z))[iso6]	LMGL03013500
		TG(14:1(9Z)/18:2(9Z,12Z)/20:5(5Z,8Z,11Z,14Z,17Z))[iso6]	LMGL03014798
	PI 17:1_18:2	PI(17:1(9Z)/18:2(9Z,12Z))	LMGP06010228
		PI(18:2(9Z,12Z)/17:1(9Z))	LMGP06010316
	PI P-18:0/18:2	PI(P-18:0/18:2(9Z,12Z))	LMGP06030041

MxP® Quant 500 XL kit lipid annotation	Potential isobars within ± 0.5 Da	Potential isomers	Data base ID
PI 18:0_18:0	TG 18:0_35:5	TG(17:2(9Z,12Z)/18:0/18:3(9Z,12Z,15Z))[iso6]	LMGL03010224
		TG(13:0/18:0/22:5(7Z,10Z,13Z,16Z,19Z))[iso6]	LMGL03013925
		TG(15:0/18:0/20:5(5Z,8Z,11Z,14Z,17Z))[iso6]	LMGL03015137
		TG(15:1(9Z)/18:0/20:4(5Z,8Z,11Z,14Z))[iso6]	LMGL03015487
		TG(17:1(9Z)/18:0/18:4(6Z,9Z,12Z,15Z))[iso6]	LMGL03015985
		TG(17:2(9Z,12Z)/18:0/18:3(6Z,9Z,12Z))[iso6]	LMGL03016063
	PI 18:0_18:0	PI(18:0/18:0)	LMGP06010008
	Glc-GP 18:0_18:0	Glc-GP(18:0/18:0)	LMGP14010003
PI 18:0_18:1	TG 18:1_35:5	TG(17:2(9Z,12Z)/18:1(9Z)/18:3(9Z,12Z,15Z))[iso6]	LMGL03010255
		TG(13:0/18:1(9Z)/22:5(7Z,10Z,13Z,16Z,19Z))[iso6]	LMGL03013945
		TG(15:0/18:1(9Z)/20:5(5Z,8Z,11Z,14Z,17Z))[iso6]	LMGL03015157
		TG(15:1(9Z)/18:1(9Z)/20:4(5Z,8Z,11Z,14Z))[iso6]	LMGL03015507
		TG(17:1(9Z)/18:1(9Z)/18:4(6Z,9Z,12Z,15Z))[iso6]	LMGL03015989
		TG(17:2(9Z,12Z)/18:1(9Z)/18:3(6Z,9Z,12Z))[iso6]	LMGL03016067
	PI 18:0_18:1	PI(18:1(9Z)/18:0)	LMGP06010932
		PI(18:0/18:1(9Z))	LMGP06010957
PI 18:0_18:2	TG 18:2_35:5	TG(17:2(9Z,12Z)/18:2(9Z,12Z)/18:3(9Z,12Z,15Z))[iso6]	LMGL03010290
		TG(13:0/18:2(9Z,12Z)/22:5(7Z,10Z,13Z,16Z,19Z))[iso6]	LMGL03013964
		TG(15:0/18:2(9Z,12Z)/20:5(5Z,8Z,11Z,14Z,17Z))[iso6]	LMGL03015176
		TG(15:1(9Z)/18:2(9Z,12Z)/20:4(5Z,8Z,11Z,14Z))[iso6]	LMGL03015526
		TG(17:1(9Z)/18:2(9Z,12Z)/18:4(6Z,9Z,12Z,15Z))[iso6]	LMGL03015993
		TG(17:2(9Z,12Z)/18:2(9Z,12Z)/18:3(6Z,9Z,12Z))[iso6]	LMGL03016071
	PI 18:0_18:2	PI(18:2(9Z,12Z)/18:0)	LMGP06010846
		PI(18:0/18:2(9Z,12Z))	LMGP06010956
PI 18:0_18:3	TG 18:0_34:1	TG(17:0/17:1(9Z)/18:0)[iso6]	LMGL03010071
		TG(16:1(9Z)/18:0/18:0)[iso3]	LMGL03010082
		TG(16:0/18:0/18:1(9Z))[iso6]	LMGL03010085
		TG(12:0/18:0/22:1(11Z))[iso6]	LMGL03013456
		TG(14:0/18:0/20:1(11Z))[iso6]	LMGL03014349
		TG(14:1(9Z)/18:0/20:0)[iso6]	LMGL03014754
		TG(15:0/18:0/19:1(9Z))[iso6]	LMGL03015131
		TG(15:1(9Z)/18:0/19:0)[iso6]	LMGL03015481
	PI 18:0_18:3	PI(18:0/18:3(6Z,9Z,12Z))	LMGP06010283
		PI(18:0/18:3(9Z,12Z,15Z))	LMGP06010284
		PI(18:3(6Z,9Z,12Z)/18:0)	LMGP06010345
		PI(18:3(9Z,12Z,15Z)/18:0)	LMGP06010375

MxP® Quant 500 XL kit lipid annotation	Potential isobars within ± 0.5 Da	Potential isomers	Data base ID
PI 18:0_20:0	TG 18:0_37:5	TG(17:2(9Z,12Z)/18:0/20:3(8Z,11Z,14Z))[iso6]	LMGL03010445
		TG(17:1(9Z)/18:0/20:4(5Z,8Z,11Z,14Z))[iso6]	LMGL03010449
		TG(17:0/18:0/20:5(5Z,8Z,11Z,14Z,17Z))[iso6]	LMGL03010453
		TG(15:0/18:0/22:5(7Z,10Z,13Z,16Z,19Z))[iso6]	LMGL03015144
		TG(15:1(9Z)/18:0/22:4(7Z,10Z,13Z,16Z))[iso6]	LMGL03015494
		TG(18:0/18:4(6Z,9Z,12Z,15Z)/19:1(9Z))[iso6]	LMGL03016172
	PI 18:0_20:0	PI(20:0/18:0)	LMGP06010924
		PI(18:0/20:0)	LMGP06010936
	Glc-GP 18:0_20:0	Glc-GP(18:0/20:0)	LMGP14010002
PI 18:0_20:3	TG 18:0_36:1	TG(18:0/18:0/18:1(9Z))[iso3]	LMGL03010188
		TG(16:1(9Z)/18:0/20:0)[iso6]	LMGL03010202
		TG(16:0/18:0/20:1(11Z))[iso6]	LMGL03010205
		TG(17:1(9Z)/18:0/19:0)[iso6]	LMGL03010228
		TG(14:0/18:0/22:1(11Z))[iso6]	LMGL03014356
		TG(14:1(9Z)/18:0/22:0)[iso6]	LMGL03014761
		TG(15:1(9Z)/18:0/21:0)[iso6]	LMGL03015489
		TG(17:0/18:0/19:1(9Z))[iso6]	LMGL03015903
	PI 18:0_20:3	PI(20:3(8Z,11Z,14Z)/18:0)	LMGP06010568
		PI(18:0/20:3(8Z,11Z,14Z))	LMGP06010855
PI 18:0_20:4	TG 18:0_36:2	TG(18:0/18:1(9Z)/18:1(9Z))[iso3]	LMGL03010217
		TG(18:0/18:0/18:2(9Z,12Z))[iso3]	LMGL03010220
		TG(16:1(9Z)/18:0/20:1(11Z))[iso6]	LMGL03010235
		TG(16:0/18:0/20:2(11Z,14Z))[iso6]	LMGL03010238
		TG(17:2(9Z,12Z)/18:0/19:0)[iso6]	LMGL03010261
		TG(14:0/18:0/22:2(13Z,16Z))[iso6]	LMGL03014357
		TG(14:1(9Z)/18:0/22:1(11Z))[iso6]	LMGL03014762
		TG(17:1(9Z)/18:0/19:1(9Z))[iso6]	LMGL03015986
	PI 18:0_20:4	PI(18:0/20:4(5Z,8Z,11Z,14Z))	LMGP06010010
		PI(20:4(5Z,8Z,11Z,14Z)/18:0)	LMGP06010598
	Glc-GP 18:0_20:4	Glc-GP(18:0/20:4(5Z,8Z,11Z,14Z))	LMGP14010001
PI 18:0_22:0	TG 18:0_39:5	TG(17:2(9Z,12Z)/18:0/22:3(10Z,13Z,16Z))[iso6]	LMGL03010839
		TG(18:0/19:0/20:5(5Z,8Z,11Z,14Z,17Z))[iso6]	LMGL03010882
		TG(17:1(9Z)/18:0/22:4(7Z,10Z,13Z,16Z))[iso6]	LMGL03010919
		TG(17:0/18:0/22:5(7Z,10Z,13Z,16Z,19Z))[iso6]	LMGL03010923
		TG(18:0/19:1(9Z)/20:4(5Z,8Z,11Z,14Z))[iso6]	LMGL03016193
	PI 18:0_22:0	PI(22:0/18:0)	LMGP06010816
		PI(18:0/22:0)	LMGP06010955

MxP® Quant 500 XL kit lipid annotation	Potential isobars within ± 0.5 Da	Potential isomers	Data base ID
PI 18:1_18:1	TG 18:1_35:6	TG(13:0/18:1(9Z)/22:6(4Z,7Z,10Z,13Z,16Z,19Z))[iso6]	LMGL03013946
		TG(15:1(9Z)/18:1(9Z)/20:5(5Z,8Z,11Z,14Z,17Z))[iso6]	LMGL03015508
		TG(17:2(9Z,12Z)/18:1(9Z)/18:4(6Z,9Z,12Z,15Z))[iso6]	LMGL03016068
	PI 18:1_18:1	PI(18:1(9Z)/18:1(9Z))	PubChem:5771759
		PI(18:1/18:1)	PubChem:52927556
PI 18:1_18:2	TG 18:1_34:0	TG(17:0/17:0/18:1(9Z))[iso3]	LMGL03010074
		TG(16:0/18:0/18:1(9Z))[iso6]	LMGL03010085
		TG(12:0/18:1(9Z)/22:0)[iso6]	LMGL03013475
		TG(13:0/18:1(9Z)/21:0)[iso6]	LMGL03013939
		TG(14:0/18:1(9Z)/20:0)[iso6]	LMGL03014368
		TG(15:0/18:1(9Z)/19:0)[iso6]	LMGL03015150
	PI 18:1_18:2	PI(18:2(9Z,12Z)/18:1(9Z))	LMGP06010318
		PI(18:1(9Z)/18:2(9Z,12Z))	LMGP06010931
PI 18:1_18:3	TG 18:1_34:1	TG(17:0/17:1(9Z)/18:1(9Z))[iso6]	LMGL03010087
		TG(16:0/18:1(9Z)/18:1(9Z))[iso3]	LMGL03010100
		TG(16:1(9Z)/18:0/18:1(9Z))[iso6]	LMGL03010101
		TG(12:0/18:1(9Z)/22:1(11Z))[iso6]	LMGL03013476
		TG(14:0/18:1(9Z)/20:1(11Z))[iso6]	LMGL03014369
		TG(14:1(9Z)/18:1(9Z)/20:0)[iso6]	LMGL03014774
		TG(15:0/18:1(9Z)/19:1(9Z))[iso6]	LMGL03015151
		TG(15:1(9Z)/18:1(9Z)/19:0)[iso6]	LMGL03015501
	PI 18:1_18:3	PI(18:1(9Z)/18:3(6Z,9Z,12Z))	LMGP06010298
		PI(18:3(6Z,9Z,12Z)/18:1(9Z))	LMGP06010346
		PI(18:3(9Z,12Z,15Z)/18:1(9Z))	LMGP06010843
		PI(18:1(9Z)/18:3(9Z,12Z,15Z))	LMGP06010850
PI 18:1_20:0	TG 18:1_37:5	TG(17:2(9Z,12Z)/18:1(9Z)/20:3(8Z,11Z,14Z))[iso6]	LMGL03010495
		TG(17:1(9Z)/18:1(9Z)/20:4(5Z,8Z,11Z,14Z))[iso6]	LMGL03010500
		TG(17:0/18:1(9Z)/20:5(5Z,8Z,11Z,14Z,17Z))[iso6]	LMGL03010504
		TG(15:0/18:1(9Z)/22:5(7Z,10Z,13Z,16Z,19Z))[iso6]	LMGL03015164
		TG(15:1(9Z)/18:1(9Z)/22:4(7Z,10Z,13Z,16Z))[iso6]	LMGL03015514
		TG(18:1(9Z)/18:4(6Z,9Z,12Z,15Z)/19:1(9Z))[iso6]	LMGL03016243
	PI 18:1_20:0	PI(20:0/18:1(9Z))	LMGP06010923
		PI(18:1(9Z)/20:0)	LMGP06010930

MxP® Quant 500 XL kit lipid annotation	Potential isobars within ± 0.5 Da	Potential isomers	Data base ID
PI 18:1_20:1	TG 18:1_37:6	TG(17:2(9Z,12Z)/18:1(9Z)/20:4(5Z,8Z,11Z,14Z))[iso6]	LMGL03010556
		TG(17:1(9Z)/18:1(9Z)/20:5(5Z,8Z,11Z,14Z,17Z))[iso6]	LMGL03010561
		TG(15:0/18:1(9Z)/22:6(4Z,7Z,10Z,13Z,16Z,19Z))[iso6]	LMGL03015165
		TG(15:1(9Z)/18:1(9Z)/22:5(7Z,10Z,13Z,16Z,19Z))[iso6]	LMGL03015515
	DGDG 14:0_18:1	DGDG(18:1(9Z)/14:0)	LMGL05010082
	PI 18:1_20:1	PI(20:1(11Z)/18:1(9Z))	LMGP06010508
		PI(18:1(9Z)/20:1(11Z))	LMGP06010929
PI 18:1_20:2	TG 18:1_36:0	TG(18:0/18:0/18:1(9Z))[iso3]	LMGL03010188
		TG(16:0/18:1(9Z)/20:0)[iso6]	LMGL03010201
		TG(17:0/18:1(9Z)/19:0)[iso6]	LMGL03010227
		TG(14:0/18:1(9Z)/22:0)[iso6]	LMGL03014375
		TG(15:0/18:1(9Z)/21:0)[iso6]	LMGL03015158
	TG 18:1_37:7	TG(17:2(9Z,12Z)/18:1(9Z)/20:5(5Z,8Z,11Z,14Z,17Z))[iso6]	LMGL03010620
		TG(15:1(9Z)/18:1(9Z)/22:6(4Z,7Z,10Z,13Z,16Z,19Z))[iso6]	LMGL03015516
	PI 18:1_20:2	PI(18:1(9Z)/20:2(11Z,14Z))	LMGP06010302
		PI(20:2(11Z,14Z)/18:1(9Z))	LMGP06010538
PI 18:1_20:3	TG 18:1_36:1	TG(18:0/18:1(9Z)/18:1(9Z))[iso3]	LMGL03010217
		TG(16:1(9Z)/18:1(9Z)/20:0)[iso6]	LMGL03010231
		TG(16:0/18:1(9Z)/20:1(11Z))[iso6]	LMGL03010234
		TG(17:1(9Z)/18:1(9Z)/19:0)[iso6]	LMGL03010260
		TG(14:0/18:1(9Z)/22:1(11Z))[iso6]	LMGL03014376
		TG(14:1(9Z)/18:1(9Z)/22:0)[iso6]	LMGL03014781
		TG(15:1(9Z)/18:1(9Z)/21:0)[iso6]	LMGL03015509
		TG(17:0/18:1(9Z)/19:1(9Z))[iso6]	LMGL03015907
	PI 18:1_20:3	PI(18:1(9Z)/20:3(8Z,11Z,14Z))	LMGP06010303
		PI(20:3(8Z,11Z,14Z)/18:1(9Z))	LMGP06010569

MxP® Quant 500 XL kit lipid annotation	Potential isobars within ± 0.5 Da	Potential isomers	Data base ID
PI 18:1_20:4	TG 18:1_36:2	TG(18:1(9Z)/18:1(9Z)/18:1(9Z))	LMGL03010250
		TG(18:0/18:1(9Z)/18:2(9Z,12Z))[iso6]	LMGL03010252
		TG(16:1(9Z)/18:1(9Z)/20:1(11Z))[iso6]	LMGL03010268
		TG(16:0/18:1(9Z)/20:2(11Z,14Z))[iso6]	LMGL03010271
		TG(17:2(9Z,12Z)/18:1(9Z)/19:0)[iso6]	LMGL03010295
		TG(18:1(9Z)/18:1(6Z)/18:1(9Z))	LMGL03012612
		TG(18:1(6Z)/18:1(9Z)/18:1(6Z))	LMGL03012613
		TG(14:0/18:1(9Z)/22:2(13Z,16Z))[iso6]	LMGL03014377
		TG(14:1(9Z)/18:1(9Z)/22:1(11Z))[iso6]	LMGL03014782
		TG(17:1(9Z)/18:1(9Z)/19:1(9Z))[iso6]	LMGL03015990
	PI 18:1_20:4	PI(20:4(5Z,8Z,11Z,14Z)/18:1(9Z))	LMGP06010599
		PI(18:1(9Z)/20:4(5Z,8Z,11Z,14Z))	LMGP06010849
PI 18:1_20:5	TG 18:1_36:3	TG(18:1(9Z)/18:1(9Z)/18:2(9Z,12Z))[iso3]	LMGL03010288
		TG(18:0/18:1(9Z)/18:3(9Z,12Z,15Z))[iso6]	LMGL03010291
		TG(16:1(9Z)/18:1(9Z)/20:2(11Z,14Z))[iso6]	LMGL03010308
		TG(16:0/18:1(9Z)/20:3(8Z,11Z,14Z))[iso6]	LMGL03010311
		TG(14:0/18:1(9Z)/22:3(10Z,13Z,16Z))[iso6]	LMGL03014378
		TG(14:1(9Z)/18:1(9Z)/22:2(13Z,16Z))[iso6]	LMGL03014783
		TG(17:2(9Z,12Z)/18:1(9Z)/19:1(9Z))[iso6]	LMGL03016069
		TG(18:0/18:1(9Z)/18:3(6Z,9Z,12Z))[iso6]	LMGL03016142
	PI 18:1_20:5	PI(20:5(5Z,8Z,11Z,14Z,17Z)/18:1(9Z))	LMGP06010826
		PI(18:1(9Z)/20:5(5Z,8Z,11Z,14Z,17Z))	LMGP06010848
PI 18:1_22:0	TG 18:1_39:5	TG(17:2(9Z,12Z)/18:1(9Z)/22:3(10Z,13Z,16Z))[iso6]	LMGL03010909
		TG(18:1(9Z)/19:0/20:5(5Z,8Z,11Z,14Z,17Z))[iso6]	LMGL03010953
		TG(17:1(9Z)/18:1(9Z)/22:4(7Z,10Z,13Z,16Z))[iso6]	LMGL03010992
		TG(17:0/18:1(9Z)/22:5(7Z,10Z,13Z,16Z,19Z))[iso6]	LMGL03010996
		TG(18:1(9Z)/19:1(9Z)/20:4(5Z,8Z,11Z,14Z))[iso6]	LMGL03016264
	PI 18:1_22:0	PI(22:0/18:1(9Z))	LMGP06010917
		PI(18:1(9Z)/22:0)	LMGP06010928

MxP® Quant 500 XL kit lipid annotation	Potential isobars within ± 0.5 Da	Potential isomers	Data base ID
PI 18:1_22:1	TG 18:1_39:6	TG(17:2(9Z,12Z)/18:1(9Z)/22:4(7Z,10Z,13Z,16Z))[iso6]	LMGL03011067
		TG(17:1(9Z)/18:1(9Z)/22:5(7Z,10Z,13Z,16Z,19Z))[iso6]	LMGL03011072
		TG(17:0/18:1(9Z)/22:6(4Z,7Z,10Z,13Z,16Z,19Z))[iso6]	LMGL03011076
		TG(18:1(9Z)/19:1(9Z)/20:5(5Z,8Z,11Z,14Z,17Z))[iso6]	LMGL03016265
	PI 18:1_22:1	PI(18:1(9Z)/22:1(11Z))	LMGP06010305
		PI(22:1(11Z)/18:1(9Z))	LMGP06010706
PI 18:1_22:2	TG 18:1:38:0	TG(18:0/18:1(9Z)/20:0)[iso6]	LMGL03010382
		TG(16:0/18:1(9Z)/22:0)[iso6]	LMGL03010511
		TG(18:1(9Z)/19:0/19:0)[iso3]	LMGL03010528
		TG(17:0/18:1(9Z)/21:0)[iso6]	LMGL03010565
	TG 18:1_39:7	TG(17:2(9Z,12Z)/18:1(9Z)/22:5(7Z,10Z,13Z,16Z,19Z))[iso6]	LMGL03011149
		TG(17:1(9Z)/18:1(9Z)/22:6(4Z,7Z,10Z,13Z,16Z,19Z))[iso6]	LMGL03011154
	PI 18:1_22:2	PI(18:1(9Z)/22:2(13Z,16Z))	LMGP06010306
		PI(22:2(13Z,16Z)/18:1(9Z))	LMGP06010737
	PIP 16:0_18:1	PIP[3'](16:0/18:1(9Z))	LMGP07010001
PI 18:1_22:3	TG 18:1_38:1	TG(18:1(9Z)/18:1(9Z)/20:0)[iso3]	LMGL03010428
		TG(18:0/18:1(9Z)/20:1(11Z))[iso6]	LMGL03010434
		TG(16:1(9Z)/18:1(9Z)/22:0)[iso6]	LMGL03010569
		TG(16:0/18:1(9Z)/22:1(13Z))[iso6]	LMGL03010572
		TG(17:1(9Z)/18:1(9Z)/21:0)[iso6]	LMGL03010625
		TG(17:2(9Z,12Z)/18:1(9Z)/22:6(4Z,7Z,10Z,13Z,16Z,19Z))[iso6]	LMGL03011232
		TG(16:0/18:1(9Z)/22:1(11Z))[iso6]	LMGL03015730
		TG(18:1(9Z)/19:0/19:1(9Z))[iso6]	LMGL03016258
PI 18:1_22:4	TG 18:1_38:2	TG(18:1(9Z)/18:2(9Z,12Z)/20:0)[iso6]	LMGL03010478
		TG(18:1(9Z)/18:1(9Z)/20:1(11Z))[iso3]	LMGL03010484
		TG(18:0/18:1(9Z)/20:2(11Z,14Z))[iso6]	LMGL03010490
		TG(16:1(9Z)/18:1(9Z)/22:1(13Z))[iso6]	LMGL03010633
		TG(17:2(9Z,12Z)/18:1(9Z)/21:0)[iso6]	LMGL03010688
		TG(16:0/18:1(9Z)/22:2(13Z,16Z))[iso6]	LMGL03010704
		TG(18:1(9Z)/19:1(9Z)/19:1(9Z))[iso3]	LMGL03013035
		TG(16:1(9Z)/18:1(9Z)/22:1(11Z))[iso6]	LMGL03015821
	PI 18:1_22:4	PI(18:1(9Z)/22:4(7Z,10Z,13Z,16Z))	LMGP06010307
		PI(22:4(7Z,10Z,13Z,16Z)/18:1(9Z))	LMGP06010768

MxP® Quant 500 XL kit lipid annotation	Potential isobars within ± 0.5 Da	Potential isomers	Data base ID
PI 18:1_22:5	TG 18:1_38:3	TG(18:1(9Z)/18:3(9Z,12Z,15Z)/20:0)[iso6]	LMGL03010532
		TG(18:1(9Z)/18:2(9Z,12Z)/20:1(11Z))[iso6]	LMGL03010538
		TG(18:1(9Z)/18:1(9Z)/20:2(11Z,14Z))[iso3]	LMGL03010545
		TG(18:0/18:1(9Z)/20:3(8Z,11Z,14Z))[iso6]	LMGL03010551
		TG(16:0/18:1(9Z)/22:3(10Z,13Z,16Z))[iso6]	LMGL03010636
		TG(16:1(9Z)/18:1(9Z)/22:2(13Z,16Z))[iso6]	LMGL03010772
		TG(18:1(9Z)/18:3(6Z,9Z,12Z)/20:0)[iso6]	LMGL03016225
	PI 18:1_22:5	[1-[hydroxy-(2,3,4,5,6-pentahydroxycyclohexyl)oxyphosphoryl]oxy-3-octadec-9-enoyloxypropan-2-yl] docosa-7,10,13,16,19-pentaenoate	PubChem:133034343
PI 18:1_22:6	TG 18:1_38:4	TG(18:1(9Z)/18:3(9Z,12Z,15Z)/20:1(11Z))[iso6]	LMGL03010595
		TG(18:1(9Z)/18:2(9Z,12Z)/20:2(11Z,14Z))[iso6]	LMGL03010602
		TG(18:1(9Z)/18:1(9Z)/20:3(8Z,11Z,14Z))[iso3]	LMGL03010609
		TG(18:0/18:1(9Z)/20:4(5Z,8Z,11Z,14Z))[iso6]	LMGL03010615
		TG(16:1(9Z)/18:1(9Z)/22:3(10Z,13Z,16Z))[iso6]	LMGL03010701
		TG(16:0/18:1(9Z)/22:4(7Z,10Z,13Z,16Z))[iso6]	LMGL03010775
		TG(18:1(9Z)/18:3(6Z,9Z,12Z)/20:1(11Z))[iso6]	LMGL03016226
		TG(18:1(9Z)/18:4(6Z,9Z,12Z,15Z)/20:0)[iso6]	LMGL03016244
	PI 18:1_22:6	PI(18:1(9Z)/22:6(4Z,7Z,10Z,13Z,16Z,19Z))	LMGP06010013
		PI(22:6(4Z,7Z,10Z,13Z,16Z,19Z)/18:1(9Z))	LMGP06010797

MxP® Quant 500 XL kit lipid annotation	Potential isobars within ± 0.5 Da	Potential isomers	Data base ID
PI 18:2_18:3	TG 18:2_34:1	TG(17:0/17:1(9Z)/18:2(9Z,12Z))[iso6]	LMGL03010106
		TG(16:0/18:1(9Z)/18:2(9Z,12Z))[iso6]	LMGL03010121
		TG(16:1(9Z)/18:0/18:2(9Z,12Z))[iso6]	LMGL03010122
		TG(12:0/18:2(9Z,12Z)/22:1(11Z))[iso6]	LMGL03013495
		TG(14:0/18:2(9Z,12Z)/20:1(11Z))[iso6]	LMGL03014388
		TG(14:1(9Z)/18:2(9Z,12Z)/20:0)[iso6]	LMGL03014793
		TG(15:0/18:2(9Z,12Z)/19:1(9Z))[iso6]	LMGL03015170
		TG(15:1(9Z)/18:2(9Z,12Z)/19:0)[iso6]	LMGL03015520
	PI 18:2_18:3	PI(18:2(9Z,12Z)/18:3(6Z,9Z,12Z))	LMGP06010319
		PI(18:2(9Z,12Z)/18:3(9Z,12Z,15Z))	LMGP06010320
		PI(18:3(6Z,9Z,12Z)/18:2(9Z,12Z))	LMGP06010347
		PI(18:3(9Z,12Z,15Z)/18:2(9Z,12Z))	LMGP06010376
PI 18:2_20:0	TG 18:2_37:5	TG(17:2(9Z,12Z)/18:2(9Z,12Z)/20:3(8Z,11Z,14Z))[iso6]	LMGL03010550
		TG(17:1(9Z)/18:2(9Z,12Z)/20:4(5Z,8Z,11Z,14Z))[iso6]	LMGL03010555
		TG(17:0/18:2(9Z,12Z)/20:5(5Z,8Z,11Z,14Z,17Z))[iso6]	LMGL03010560
		TG(15:0/18:2(9Z,12Z)/22:5(7Z,10Z,13Z,16Z,19Z))[iso6]	LMGL03015183
		TG(15:1(9Z)/18:2(9Z,12Z)/22:4(7Z,10Z,13Z,16Z))[iso6]	LMGL03015533
		TG(18:2(9Z,12Z)/18:4(6Z,9Z,12Z,15Z)/19:1(9Z))[iso6]	LMGL03016310
	PI 18:2_20:0	PI(18:2(9Z,12Z)/20:0)	LMGP06010324
		PI(20:0/18:2(9Z,12Z))	LMGP06010922
PI 18:2_20:1	TG 18:2_37:6	TG(17:2(9Z,12Z)/18:2(9Z,12Z)/20:4(5Z,8Z,11Z,14Z))[iso6]	LMGL03010614
		TG(17:1(9Z)/18:2(9Z,12Z)/20:5(5Z,8Z,11Z,14Z,17Z))[iso6]	LMGL03010619
		TG(15:0/18:2(9Z,12Z)/22:6(4Z,7Z,10Z,13Z,16Z,19Z))[iso6]	LMGL03015184
		TG(15:1(9Z)/18:2(9Z,12Z)/22:5(7Z,10Z,13Z,16Z,19Z))[iso6]	LMGL03015534
	DGDG 14:0_18:2	DGDG(18:2(9Z,12Z)/14:0)	LMGL05010080
	PI 18:2_20:1	PI(18:2(9Z,12Z)/20:1(11Z))	LMGP06010325
		PI(20:1(11Z)/18:2(9Z,12Z))	LMGP06010509

MxP® Quant 500 XL kit lipid annotation	Potential isobars within ± 0.5 Da	Potential isomers	Data base ID
PI 18:2_20:4	TG 18:2_36:2	TG(18:0/18:2(9Z,12Z)/18:2(9Z,12Z))[iso3]	LMGL03010287
		TG(18:1(9Z)/18:1(9Z)/18:2(9Z,12Z))[iso3]	LMGL03010288
		TG(16:1(9Z)/18:2(9Z,12Z)/20:1(11Z))[iso6]	LMGL03010303
		TG(16:0/18:2(9Z,12Z)/20:2(11Z,14Z))[iso6]	LMGL03010307
		TG(17:2(9Z,12Z)/18:2(9Z,12Z)/19:0)[iso6]	LMGL03010333
		TG(14:0/18:2(9Z,12Z)/22:2(13Z,16Z))[iso6]	LMGL03014396
		TG(14:1(9Z)/18:2(9Z,12Z)/22:1(11Z))[iso6]	LMGL03014801
		TG(17:1(9Z)/18:2(9Z,12Z)/19:1(9Z))[iso6]	LMGL03015994
	PI 18:2_20:4	PI(20:4(5Z,8Z,11Z,14Z)/18:2(9Z,12Z))	LMGP06010600
		PI(18:2(9Z,12Z)/20:4(5Z,8Z,11Z,14Z))	LMGP06010845
PI 18:2_20:5	TG 18:2_36:3	TG(18:1(9Z)/18:2(9Z,12Z)/18:2(9Z,12Z))[iso3]	LMGL03010327
		TG(18:0/18:2(9Z,12Z)/18:3(9Z,12Z,15Z))[iso6]	LMGL03010329
		TG(16:1(9Z)/18:2(9Z,12Z)/20:2(11Z,14Z))[iso6]	LMGL03010347
		TG(16:0/18:2(9Z,12Z)/20:3(8Z,11Z,14Z))[iso6]	LMGL03010351
		TG(14:0/18:2(9Z,12Z)/22:3(10Z,13Z,16Z))[iso6]	LMGL03014397
		TG(14:1(9Z)/18:2(9Z,12Z)/22:2(13Z,16Z))[iso6]	LMGL03014802
		TG(17:2(9Z,12Z)/18:2(9Z,12Z)/19:1(9Z))[iso6]	LMGL03016073
		TG(18:0/18:2(9Z,12Z)/18:3(6Z,9Z,12Z))[iso6]	LMGL03016146
	PI 18:2_20:5	PI(18:2(9Z,12Z)/20:5(5Z,8Z,11Z,14Z,17Z))	LMGP06010328
		PI(20:5(5Z,8Z,11Z,14Z,17Z)/18:2(9Z,12Z))	LMGP06010629
PI 18:2_22:0	TG 18:2_39:5	TG(17:2(9Z,12Z)/18:2(9Z,12Z)/22:3(10Z,13Z,16Z))[iso6]	LMGL03010981
		TG(18:2(9Z,12Z)/19:0/20:5(5Z,8Z,11Z,14Z,17Z))[iso6]	LMGL03011025
		TG(17:1(9Z)/18:2(9Z,12Z)/22:4(7Z,10Z,13Z,16Z))[iso6]	LMGL03011066
		TG(17:0/18:2(9Z,12Z)/22:5(7Z,10Z,13Z,16Z,19Z))[iso6]	LMGL03011071
		TG(18:2(9Z,12Z)/19:1(9Z)/20:4(5Z,8Z,11Z,14Z))[iso6]	LMGL03016331
	PI 18:2_22:0	PI(18:2(9Z,12Z)/22:0)	LMGP06010330
		PI(22:0/18:2(9Z,12Z))	LMGP06010679

MxP® Quant 500 XL kit lipid annotation	Potential isobars within ± 0.5 Da	Potential isomers	Data base ID
PI 18:2_22:1	TG 18:2_39:6	TG(17:2(9Z,12Z)/18:2(9Z,12Z)/22:4(7Z,10Z,13Z,16Z))[iso6]	LMGL03011143
		TG(17:1(9Z)/18:2(9Z,12Z)/22:5(7Z,10Z,13Z,16Z,19Z))[iso6]	LMGL03011148
		TG(17:0/18:2(9Z,12Z)/22:6(4Z,7Z,10Z,13Z,16Z,19Z))[iso6]	LMGL03011153
		TG(18:2(9Z,12Z)/19:1(9Z)/20:5(5Z,8Z,11Z,14Z,17Z))[iso6]	LMGL03016332
	PI 18:2_22:1	PI(18:2(9Z,12Z)/22:1(11Z))	LMGP06010331
		PI(22:1(11Z)/18:2(9Z,12Z))	LMGP06010707
PI 18:2_22:6	TG 18:2_38:4	TG(18:2(9Z,12Z)/18:3(9Z,12Z,15Z)/20:1(11Z))[iso6]	LMGL03010657
		TG(18:2(9Z,12Z)/18:2(9Z,12Z)/20:2(11Z,14Z))[iso3]	LMGL03010663
		TG(18:1(9Z)/18:2(9Z,12Z)/20:3(8Z,11Z,14Z))[iso6]	LMGL03010670
		TG(18:0/18:2(9Z,12Z)/20:4(5Z,8Z,11Z,14Z))[iso6]	LMGL03010676
		TG(16:1(9Z)/18:2(9Z,12Z)/22:3(10Z,13Z,16Z))[iso6]	LMGL03010767
		TG(16:0/18:2(9Z,12Z)/22:4(7Z,10Z,13Z,16Z))[iso6]	LMGL03010845
		TG(18:2(9Z,12Z)/18:3(6Z,9Z,12Z)/20:1(11Z))[iso6]	LMGL03016293
		TG(18:2(9Z,12Z)/18:4(6Z,9Z,12Z,15Z)/20:0)[iso6]	LMGL03016311
	PI 18:2_22:6	PI(22:6(4Z,7Z,10Z,13Z,16Z,19Z)/18:2(9Z,12Z))	LMGP06010798
		PI(18:2(9Z,12Z)/22:6(4Z,7Z,10Z,13Z,16Z,19Z))	LMGP06010844

Lysophosphatidylserines (12)			
MxP® Quant 500 XL kit lipid annotation	Potential isobars within ± 0.5 Da	Potential isomers	Data base ID
LPS 16:0	LPS 16:0	PS(16:0/0:0)	LMGP03050002
	LPS O-16:1;O	1-(2-methoxy-13-methyl-6Z-tetradecenyl)-sn-glycero-3-phosphoserine	LMGP03060006
		1-(2-methoxy-6Z-pentadecenyl)-sn-glycero-3-phosphoserine	LMGP03060011
LPS 16:1	LPS 16:1	PS(16:1(9Z)/0:0)	LMGP03050010
LPS 18:0	LPS 18:0	PS(18:0/0:0)	LMGP03050006
	LPS O-18:1;O	1-(2-methoxy-6Z-heptadecenyl)-sn-glycero-3-phosphoserine	LMGP03060015
LPS 18:1	LPS 18:1	PS(18:1(9Z)/0:0)	LMGP03050001
LPS 18:2	LPS 18:2	PS(18:2(9Z,12Z)/0:0)	LMGP03050011
LPS 18:3	LPS 18:3	PS(18:3(6Z,9Z,12Z)/0:0)	LMGP03050017
		PS(18:3(9Z,12Z,15Z)/0:0)	LMGP03050029
LPS 20:0	LPS 20:0	PS(20:0/0:0)	LMGP03050012
LPS 20:1	LPS 20:1	PS(20:1(11Z)/0:0)	LMGP03050020
LPS 20:4	LPS 20:4	PS(20:4(5Z,8Z,11Z,14Z)/0:0)	LMGP03050007
LPS 20:5	LPS 20:5	PS(20:5(5Z,8Z,11Z,14Z,17Z)/0:0)	LMGP03050027
LPS 22:0	LPS 22:0	PS(22:0/0:0)	LMGP03050025
	PS 20:1;O	POB-PS	LMGP20040017

MxP® Quant 500 XL kit lipid annotation	Potential isobars within ± 0.5 Da	Potential isomers	Data base ID
LPS 22:6	LPS 22:6	PS(22:6(4Z,7Z,10Z,13Z,16Z,19Z)/0:0)	LMGP03050013
	LPS O-21:0;O	1-(2-methoxy-eicosanyl)-sn-glycero-3-phosphoserine	LMGP03060019

Phosphatidylserines (18)			
**MxP® Quant 500 XL kit lipid annotation**	**Potential isobars within ± 0.5 Da**	**Potential isomers**	**Data base ID**
PS 30:0	PS 30:0	PS(18:0/12:0)	LMGP03010889
		PS(17:0/13:0)	LMGP03010897
		PS(13:0/17:0)	LMGP03010935
		PS(12:0/18:0)	LMGP03010941
		PS(16:0/14:0)	LMGP03010970
		PS(15:0/15:0)	LMGP03010982
		PS(14:0/16:0)	LMGP03010983
	PS O-31:0	PS(O-16:0/15:0)	LMGP03020005
		PS(O-18:0/13:0)	LMGP03020019
	PS 28:2;O2	PHODA-PS	LMGP20040001
PS 32:0	PS 32:0	PS(16:0/16:0)	LMGP03010029
		PS(20:0/12:0)	LMGP03010867
		PS(19:0/13:0)	LMGP03010872
		PS(17:0/15:0)	LMGP03010896
		PS(15:0/17:0)	LMGP03010915
		PS(14:0/18:0)	LMGP03010928
		PS(13:0/19:0)	LMGP03010934
		PS(12:0/20:0)	LMGP03010939
		PS(18:0/14:0)	LMGP03010980
	PS O-33:0	PS(O-18:0/15:0)	LMGP03020022
		PS(O-20:0/13:0)	LMGP03020042
		PS(O-16:0/17:0)	LMGP03020081
PS 34:1	PS 34:1	PS(16:0/18:1(11Z))	LMGP03010007
		PS(16:0/18:1(9Z))	LMGP03010024
		PS(12:0/22:1(11Z))	LMGP03010065
		PS(14:0/20:1(11Z))	LMGP03010102
		PS(14:1(9Z)/20:0)	LMGP03010127
		PS(15:0/19:1(9Z))	LMGP03010149
		PS(15:1(9Z)/19:0)	LMGP03010178
		PS(16:1(9Z)/18:0)	LMGP03010211
		PS(17:0/17:1(9Z))	LMGP03010231
		PS(17:1(9Z)/17:0)	LMGP03010256
		PS(19:0/15:1(9Z))	LMGP03010457
		PS(19:1(9Z)/15:0)	LMGP03010484
		PS(20:0/14:1(9Z))	LMGP03010511
		PS(20:1(11Z)/14:0)	LMGP03010530
		PS(22:1(11Z)/12:0)	LMGP03010725
		PS(18:0/16:1(9Z))	LMGP03010887
		PS(18:1(9Z)/16:0)	LMGP03010959
	PS O-35:1	PS(O-16:0/19:1(9Z))	LMGP03020011
		PS(O-18:0/17:1(9Z))	LMGP03020025
		PS(O-20:0/15:1(9Z))	LMGP03020046
		PS(P-16:0/19:0)	LMGP03030016
		PS(P-18:0/17:0)	LMGP03030036
		PS(P-20:0/15:0)	LMGP03030060

MxP® Quant 500 XL kit lipid annotation	Potential isobars within ± 0.5 Da	Potential isomers	Data base ID
PS 34:2	PS 34:2	PS(12:0/22:2(13Z,16Z))	LMGP03010066
		PS(14:0/20:2(11Z,14Z))	LMGP03010103
		PS(14:1(9Z)/20:1(11Z))	LMGP03010128
		PS(15:1(9Z)/19:1(9Z))	LMGP03010179
		PS(17:0/17:2(9Z,12Z))	LMGP03010232
		PS(17:2(9Z,12Z)/17:0)	LMGP03010286
		PS(19:1(9Z)/15:1(9Z))	LMGP03010485
		PS(20:1(11Z)/14:1(9Z))	LMGP03010531
		PS(20:2(11Z,14Z)/14:0)	LMGP03010560
		PS(22:2(13Z,16Z)/12:0)	LMGP03010756
		PS(18:2(9Z,12Z)/16:0)	LMGP03010877
		PS(18:1(9Z)/16:1(9Z))	LMGP03010881
		PS(17:1(9Z)/17:1(9Z))	LMGP03010891
		PS(16:1(9Z)/18:1(9Z))	LMGP03010901
		PS(16:0/18:2(9Z,12Z))	LMGP03010976
	PS O-35:2	PS(O-18:0/17:2(9Z,12Z))	LMGP03020026
		PS(P-16:0/19:1(9Z))	LMGP03030017
		PS(P-18:0/17:1(9Z))	LMGP03030037
		PS(P-20:0/15:1(9Z))	LMGP03030061
PS 36:1	PS 36:1	PS(18:0/18:1(9Z))	LMGP03010025
		PS(18:1(9Z)/18:0)	LMGP03010034
		PS(14:0/22:1(11Z))	LMGP03010106
		PS(14:1(9Z)/22:0)	LMGP03010134
		PS(15:1(9Z)/21:0)	LMGP03010186
		PS(16:1(9Z)/20:0)	LMGP03010216
		PS(17:0/19:1(9Z))	LMGP03010238
		PS(17:1(9Z)/19:0)	LMGP03010264
		PS(19:0/17:1(9Z))	LMGP03010460
		PS(19:1(9Z)/17:0)	LMGP03010488
		PS(20:0/16:1(9Z))	LMGP03010514
		PS(20:1(11Z)/16:0)	LMGP03010534
		PS(21:0/15:1(9Z))	LMGP03010679
		PS(22:0/14:1(9Z))	LMGP03010702
		PS(22:1(11Z)/14:0)	LMGP03010727
		PS(16:0/20:1(11Z))	LMGP03010967
	PS O-37:1	PS(O-18:0/19:1(9Z))	LMGP03020033
		PS(O-20:0/17:1(9Z))	LMGP03020049
		PS(P-16:0/21:0)	LMGP03030023
		PS(P-18:0/19:0)	LMGP03030044
		PS(P-20:0/17:0)	LMGP03030064

MxP® Quant 500 XL kit lipid annotation	Potential isobars within ± 0.5 Da	Potential isomers	Data base ID
PS 36:2	PS 36:2	PS(18:1(9Z)/18:1(9Z))	LMGP03010030
		PS(18:0/18:2(9Z,12Z))	LMGP03010031
		PS(14:0/22:2(13Z,16Z))	LMGP03010107
		PS(14:1(9Z)/22:1(11Z))	LMGP03010135
		PS(16:0/20:2(11Z,14Z))	LMGP03010197
		PS(16:1(9Z)/20:1(11Z))	LMGP03010217
		PS(17:1(9Z)/19:1(9Z))	LMGP03010265
		PS(17:2(9Z,12Z)/19:0)	LMGP03010294
		PS(19:0/17:2(9Z,12Z))	LMGP03010461
		PS(19:1(9Z)/17:1(9Z))	LMGP03010489
		PS(20:1(11Z)/16:1(9Z))	LMGP03010535
		PS(20:2(11Z,14Z)/16:0)	LMGP03010564
		PS(22:1(11Z)/14:1(9Z))	LMGP03010728
		PS(22:2(13Z,16Z)/14:0)	LMGP03010758
		PS(18:2(9Z,12Z)/18:0)	LMGP03010876
	PS O-37:2	PS(O-20:0/17:2(9Z,12Z))	LMGP03020050
		PS(P-18:0/19:1(9Z))	LMGP03030045
		PS(P-20:0/17:1(9Z))	LMGP03030065
PS 36:3	PS 36:3	PS(14:1(9Z)/22:2(13Z,16Z))	LMGP03010136
		PS(16:1(9Z)/20:2(11Z,14Z))	LMGP03010218
		PS(17:2(9Z,12Z)/19:1(9Z))	LMGP03010295
		PS(18:0/18:3(6Z,9Z,12Z))	LMGP03010315
		PS(18:0/18:3(9Z,12Z,15Z))	LMGP03010316
		PS(18:2(9Z,12Z)/18:1(9Z))	LMGP03010350
		PS(18:3(6Z,9Z,12Z)/18:0)	LMGP03010377
		PS(18:3(9Z,12Z,15Z)/18:0)	LMGP03010407
		PS(19:1(9Z)/17:2(9Z,12Z))	LMGP03010490
		PS(20:2(11Z,14Z)/16:1(9Z))	LMGP03010565
		PS(20:3(8Z,11Z,14Z)/16:0)	LMGP03010595
		PS(22:2(13Z,16Z)/14:1(9Z))	LMGP03010759
		PS(16:0/20:3(8Z,11Z,14Z))	LMGP03010903
		PS(18:1(9Z)/18:2(9Z,12Z))	LMGP03010958
	PS O-37:3	PS(P-20:0/17:2(9Z,12Z))	LMGP03030066
PS 36:4	PS 36:4	PS(18:2(9Z,12Z)/18:2(9Z,12Z))	LMGP03010023
		PS(16:0/20:4(5Z,8Z,11Z,14Z))	LMGP03010038
		PS(14:0/22:4(7Z,10Z,13Z,16Z))	LMGP03010108
		PS(16:1(9Z)/20:3(8Z,11Z,14Z))	LMGP03010219
		PS(18:1(9Z)/18:3(6Z,9Z,12Z))	LMGP03010330
		PS(18:3(6Z,9Z,12Z)/18:1(9Z))	LMGP03010378
		PS(18:4(6Z,9Z,12Z,15Z)/18:0)	LMGP03010436
		PS(20:3(8Z,11Z,14Z)/16:1(9Z))	LMGP03010596
		PS(22:4(7Z,10Z,13Z,16Z)/14:0)	LMGP03010789
		PS(20:4(5Z,8Z,11Z,14Z)/16:0)	LMGP03010859
		PS(18:3(9Z,12Z,15Z)/18:1(9Z))	LMGP03010874
		PS(18:1(9Z)/18:3(9Z,12Z,15Z))	LMGP03010880
		PS(18:0/18:4(6Z,9Z,12Z,15Z))	LMGP03010886

MxP® Quant 500 XL kit lipid annotation	Potential isobars within ± 0.5 Da	Potential isomers	Data base ID
PS 36:5	PS 36:5	PS(14:1(9Z)/22:4(7Z,10Z,13Z,16Z))	LMGP03010137
		PS(18:1(9Z)/18:4(6Z,9Z,12Z,15Z))	LMGP03010331
		PS(18:2(9Z,12Z)/18:3(6Z,9Z,12Z))	LMGP03010351
		PS(18:2(9Z,12Z)/18:3(9Z,12Z,15Z))	LMGP03010352
		PS(18:3(6Z,9Z,12Z)/18:2(9Z,12Z))	LMGP03010379
		PS(18:3(9Z,12Z,15Z)/18:2(9Z,12Z))	LMGP03010408
		PS(18:4(6Z,9Z,12Z,15Z)/18:1(9Z))	LMGP03010437
		PS(20:4(5Z,8Z,11Z,14Z)/16:1(9Z))	LMGP03010626
		PS(20:5(5Z,8Z,11Z,14Z,17Z)/16:0)	LMGP03010654
		PS(22:4(7Z,10Z,13Z,16Z)/14:1(9Z))	LMGP03010790
		PS(16:1(9Z)/20:4(5Z,8Z,11Z,14Z))	LMGP03010898
		PS(16:0/20:5(5Z,8Z,11Z,14Z,17Z))	LMGP03010902
PS 38:4	PS 38:4	PS(18:0/20:4(5Z,8Z,11Z,14Z))	LMGP03010039
		PS(18:1(9Z)/20:3(8Z,11Z,14Z))	LMGP03010335
		PS(18:2(9Z,12Z)/20:2(11Z,14Z))	LMGP03010358
		PS(18:3(6Z,9Z,12Z)/20:1(11Z))	LMGP03010386
		PS(18:3(9Z,12Z,15Z)/20:1(11Z))	LMGP03010414
		PS(18:4(6Z,9Z,12Z,15Z)/20:0)	LMGP03010444
		PS(20:1(11Z)/18:3(6Z,9Z,12Z))	LMGP03010542
		PS(20:1(11Z)/18:3(9Z,12Z,15Z))	LMGP03010543
		PS(20:2(11Z,14Z)/18:2(9Z,12Z))	LMGP03010571
		PS(20:3(8Z,11Z,14Z)/18:1(9Z))	LMGP03010601
		PS(20:4(5Z,8Z,11Z,14Z)/18:0)	LMGP03010630
		PS(22:4(7Z,10Z,13Z,16Z)/16:0)	LMGP03010793
		PS(20:0/18:4(6Z,9Z,12Z,15Z))	LMGP03010949
		PS(16:0/22:4(7Z,10Z,13Z,16Z))	LMGP03010965

MxP® Quant 500 XL kit lipid annotation	Potential isobars within ± 0.5 Da	Potential isomers	Data base ID
PS 38:5	PS 38:5	PS(16:1(9Z)/22:4(7Z,10Z,13Z,16Z))	LMGP03010225
		PS(18:2(9Z,12Z)/20:3(8Z,11Z,14Z))	LMGP03010359
		PS(18:3(6Z,9Z,12Z)/20:2(11Z,14Z))	LMGP03010387
		PS(18:3(9Z,12Z,15Z)/20:2(11Z,14Z))	LMGP03010415
		PS(18:4(6Z,9Z,12Z,15Z)/20:1(11Z))	LMGP03010445
		PS(20:1(11Z)/18:4(6Z,9Z,12Z,15Z))	LMGP03010544
		PS(20:2(11Z,14Z)/18:3(6Z,9Z,12Z))	LMGP03010572
		PS(20:2(11Z,14Z)/18:3(9Z,12Z,15Z))	LMGP03010573
		PS(20:3(8Z,11Z,14Z)/18:2(9Z,12Z))	LMGP03010602
		PS(20:4(5Z,8Z,11Z,14Z)/18:1(9Z))	LMGP03010631
		PS(20:5(5Z,8Z,11Z,14Z,17Z)/18:0)	LMGP03010659
		PS(22:4(7Z,10Z,13Z,16Z)/16:1(9Z))	LMGP03010794
		PS(18:1(9Z)/20:4(5Z,8Z,11Z,14Z))	LMGP03010879
		PS(18:0/20:5(5Z,8Z,11Z,14Z,17Z))	LMGP03010884
PS 38:6	PS 38:6	PS(16:0/22:6(4Z,7Z,10Z,13Z,16Z,19Z))	LMGP03010043
		PS(18:3(6Z,9Z,12Z)/20:3(8Z,11Z,14Z))	LMGP03010388
		PS(18:3(9Z,12Z,15Z)/20:3(8Z,11Z,14Z))	LMGP03010416
		PS(18:4(6Z,9Z,12Z,15Z)/20:2(11Z,14Z))	LMGP03010446
		PS(20:2(11Z,14Z)/18:4(6Z,9Z,12Z,15Z))	LMGP03010574
		PS(20:3(8Z,11Z,14Z)/18:3(6Z,9Z,12Z))	LMGP03010603
		PS(20:3(8Z,11Z,14Z)/18:3(9Z,12Z,15Z))	LMGP03010604
		PS(20:4(5Z,8Z,11Z,14Z)/18:2(9Z,12Z))	LMGP03010632
		PS(22:6(4Z,7Z,10Z,13Z,16Z,19Z)/16:0)	LMGP03010846
		PS(20:5(5Z,8Z,11Z,14Z,17Z)/18:1(9Z))	LMGP03010857
		PS(18:2(9Z,12Z)/20:4(5Z,8Z,11Z,14Z))	LMGP03010875
		PS(18:1(9Z)/20:5(5Z,8Z,11Z,14Z,17Z))	LMGP03010878

MxP® Quant 500 XL kit lipid annotation	Potential isobars within ± 0.5 Da	Potential isomers	Data base ID
PS 38:7	PS 37:0	PS(15:0/22:0)	LMGP03010156
		PS(16:0/21:0)	LMGP03010198
		PS(18:0/19:0)	LMGP03010317
		PS(20:0/17:0)	LMGP03010515
		PS(21:0/16:0)	LMGP03010680
		PS(22:0/15:0)	LMGP03010703
		PS(19:0/18:0)	LMGP03010869
		PS(17:0/20:0)	LMGP03010963
	PS 38:7	PS(16:1(9Z)/22:6(4Z,7Z,10Z,13Z,16Z,19Z))	LMGP03010044
		PS(18:2(9Z,12Z)/20:5(5Z,8Z,11Z,14Z,17Z))	LMGP03010360
		PS(18:3(6Z,9Z,12Z)/20:4(5Z,8Z,11Z,14Z))	LMGP03010389
		PS(18:3(9Z,12Z,15Z)/20:4(5Z,8Z,11Z,14Z))	LMGP03010417
		PS(18:4(6Z,9Z,12Z,15Z)/20:3(8Z,11Z,14Z))	LMGP03010447
		PS(20:3(8Z,11Z,14Z)/18:4(6Z,9Z,12Z,15Z))	LMGP03010605
		PS(20:4(5Z,8Z,11Z,14Z)/18:3(6Z,9Z,12Z))	LMGP03010633
		PS(20:4(5Z,8Z,11Z,14Z)/18:3(9Z,12Z,15Z))	LMGP03010634
		PS(20:5(5Z,8Z,11Z,14Z,17Z)/18:2(9Z,12Z))	LMGP03010660
		PS(22:6(4Z,7Z,10Z,13Z,16Z,19Z)/16:1(9Z))	LMGP03010823
	PS O-38:0	PS(O-20:0/18:0)	LMGP03020051
		PS(O-18:0/20:0)	LMGP03020086
		PS(O-16:0/22:0)	LMGP03020089
PS 40:4	PS 40:4	PS(18:2(9Z,12Z)/22:2(13Z,16Z))	LMGP03010364
		PS(18:3(6Z,9Z,12Z)/22:1(11Z))	LMGP03010393
		PS(18:3(9Z,12Z,15Z)/22:1(11Z))	LMGP03010421
		PS(18:4(6Z,9Z,12Z,15Z)/22:0)	LMGP03010451
		PS(20:1(11Z)/20:3(8Z,11Z,14Z))	LMGP03010549
		PS(20:2(11Z,14Z)/20:2(11Z,14Z))	LMGP03010579
		PS(20:3(8Z,11Z,14Z)/20:1(11Z))	LMGP03010609
		PS(20:4(5Z,8Z,11Z,14Z)/20:0)	LMGP03010638
		PS(22:0/18:4(6Z,9Z,12Z,15Z))	LMGP03010713
		PS(22:1(11Z)/18:3(6Z,9Z,12Z))	LMGP03010739
		PS(22:1(11Z)/18:3(9Z,12Z,15Z))	LMGP03010740
		PS(22:2(13Z,16Z)/18:2(9Z,12Z))	LMGP03010769
		PS(22:4(7Z,10Z,13Z,16Z)/18:0)	LMGP03010798
		PS(20:0/20:4(5Z,8Z,11Z,14Z))	LMGP03010862
		PS(18:0/22:4(7Z,10Z,13Z,16Z))	LMGP03010883

MxP® Quant 500 XL kit lipid annotation	Potential isobars within ± 0.5 Da	Potential isomers	Data base ID
PS 40:5	PS 40:5	PS(18:1(9Z)/22:4(7Z,10Z,13Z,16Z))	LMGP03010339
		PS(18:3(6Z,9Z,12Z)/22:2(13Z,16Z))	LMGP03010394
		PS(18:3(9Z,12Z,15Z)/22:2(13Z,16Z))	LMGP03010422
		PS(18:4(6Z,9Z,12Z,15Z)/22:1(11Z))	LMGP03010452
		PS(20:0/20:5(5Z,8Z,11Z,14Z,17Z))	LMGP03010523
		PS(20:1(11Z)/20:4(5Z,8Z,11Z,14Z))	LMGP03010550
		PS(20:2(11Z,14Z)/20:3(8Z,11Z,14Z))	LMGP03010580
		PS(20:3(8Z,11Z,14Z)/20:2(11Z,14Z))	LMGP03010610
		PS(20:4(5Z,8Z,11Z,14Z)/20:1(11Z))	LMGP03010639
		PS(20:5(5Z,8Z,11Z,14Z,17Z)/20:0)	LMGP03010666
		PS(22:1(11Z)/18:4(6Z,9Z,12Z,15Z))	LMGP03010741
		PS(22:2(13Z,16Z)/18:3(6Z,9Z,12Z))	LMGP03010770
		PS(22:2(13Z,16Z)/18:3(9Z,12Z,15Z))	LMGP03010771
		PS(22:4(7Z,10Z,13Z,16Z)/18:1(9Z))	LMGP03010799
PS 40:6	PS 40:6	PS(18:0/22:6(4Z,7Z,10Z,13Z,16Z,19Z))	LMGP03010040
		PS(18:2(9Z,12Z)/22:4(7Z,10Z,13Z,16Z))	LMGP03010365
		PS(18:4(6Z,9Z,12Z,15Z)/22:2(13Z,16Z))	LMGP03010453
		PS(20:1(11Z)/20:5(5Z,8Z,11Z,14Z,17Z))	LMGP03010551
		PS(20:2(11Z,14Z)/20:4(5Z,8Z,11Z,14Z))	LMGP03010581
		PS(20:3(8Z,11Z,14Z)/20:3(8Z,11Z,14Z))	LMGP03010611
		PS(20:4(5Z,8Z,11Z,14Z)/20:2(11Z,14Z))	LMGP03010640
		PS(20:5(5Z,8Z,11Z,14Z,17Z)/20:1(11Z))	LMGP03010667
		PS(22:2(13Z,16Z)/18:4(6Z,9Z,12Z,15Z))	LMGP03010772
		PS(22:4(7Z,10Z,13Z,16Z)/18:2(9Z,12Z))	LMGP03010800
		PS(22:6(4Z,7Z,10Z,13Z,16Z,19Z)/18:0)	LMGP03010827

MxP® Quant 500 XL kit lipid annotation	Potential isobars within ± 0.5 Da	Potential isomers	Data base ID
PS 40:7	PS 39:0	PS(17:0/22:0)	LMGP03010244
		PS(18:0/21:0)	LMGP03010319
		PS(19:0/20:0)	LMGP03010468
		PS(20:0/19:0)	LMGP03010520
		PS(22:0/17:0)	LMGP03010707
		PS(21:0/18:0)	LMGP03010852
	PS 40:7	PS(18:1(9Z)/22:6(4Z,7Z,10Z,13Z,16Z,19Z))	LMGP03010045
		PS(18:3(6Z,9Z,12Z)/22:4(7Z,10Z,13Z,16Z))	LMGP03010395
		PS(18:3(9Z,12Z,15Z)/22:4(7Z,10Z,13Z,16Z))	LMGP03010423
		PS(20:2(11Z,14Z)/20:5(5Z,8Z,11Z,14Z,17Z))	LMGP03010582
		PS(20:3(8Z,11Z,14Z)/20:4(5Z,8Z,11Z,14Z))	LMGP03010612
		PS(20:4(5Z,8Z,11Z,14Z)/20:3(8Z,11Z,14Z))	LMGP03010641
		PS(20:5(5Z,8Z,11Z,14Z,17Z)/20:2(11Z,14Z))	LMGP03010668
		PS(22:4(7Z,10Z,13Z,16Z)/18:3(6Z,9Z,12Z))	LMGP03010801
		PS(22:4(7Z,10Z,13Z,16Z)/18:3(9Z,12Z,15Z))	LMGP03010802
		PS(22:6(4Z,7Z,10Z,13Z,16Z,19Z)/18:1(9Z))	LMGP03010828
	PS O-40:0	PS(O-20:0/20:0)	LMGP03020059
		PS(O-18:0/22:0)	LMGP03020084
PS 40:8	PS 39:1	PS(17:0/22:1(11Z))	LMGP03010245
		PS(17:1(9Z)/22:0)	LMGP03010273
		PS(18:1(9Z)/21:0)	LMGP03010336
		PS(19:0/20:1(11Z))	LMGP03010469
		PS(19:1(9Z)/20:0)	LMGP03010499
		PS(20:0/19:1(9Z))	LMGP03010521
		PS(20:1(11Z)/19:0)	LMGP03010545
		PS(21:0/18:1(9Z))	LMGP03010685
		PS(22:0/17:1(9Z))	LMGP03010708
		PS(22:1(11Z)/17:0)	LMGP03010733
	PS 40:8	PS(18:4(6Z,9Z,12Z,15Z)/22:4(7Z,10Z,13Z,16Z))	LMGP03010454
		PS(20:3(8Z,11Z,14Z)/20:5(5Z,8Z,11Z,14Z,17Z))	LMGP03010613
		PS(20:5(5Z,8Z,11Z,14Z,17Z)/20:3(8Z,11Z,14Z))	LMGP03010669
		PS(22:4(7Z,10Z,13Z,16Z)/18:4(6Z,9Z,12Z,15Z))	LMGP03010803
		PS(22:6(4Z,7Z,10Z,13Z,16Z,19Z)/18:2(9Z,12Z))	LMGP03010829
		PS(20:4(5Z,8Z,11Z,14Z)/20:4(5Z,8Z,11Z,14Z))	LMGP03010858
		PS(18:2(9Z,12Z)/22:6(4Z,7Z,10Z,13Z,16Z,19Z))	LMGP03010979
	PS O-40:1	PS(O-18:0/22:1(11Z))	LMGP03020038
		PS(O-20:0/20:1(11Z))	LMGP03020060
		PS(P-18:0/22:0)	LMGP03030052
		PS(P-20:0/20:0)	LMGP03030075

Sphinganines and sphingosines (8)			
MxP® Quant 500 XL kit lipid annotation	Potential isobars within ± 0.5 Da	Potential isomers	Data base ID
SPB d14:0	SPB 14:0;2O	2-Aminotetradecane-1,3-diol	PubChem:12590333
SPB d14:1	SPB 14:1;O2	C14 sphingosine	LMSP01040006
SPB d16:0	SPB 16:0;O2	C16 Sphinganine	LMSP01040001
SPB d16:1	SPB 16:1;O2	C16 Sphingosine	LMSP01040008
SPB d17:0	SPB 17:0;O2	C17 Sphinganine	LMSP01040003
		Isoheptadecasphinganine	LMSP01020003
SPB d17:1	SPB 17:1;O2	C17 Sphingosine	LMSP01040002
		iso (4E,15-methyl-d16:1) sphingosine	LMSP01080005
		anteiso (4E,14-methyl-d16:1) sphingosine	LMSP01080006
SPB d18:0	SPB 18:0;O2	Shinganine	LMSP01020001
SPB d18:1	SPB 18:1;O2	Sphingosine	LMSP01010001
		(8Z,d18:1) sphingosine	LMSP01080008
		5-hydroxy,3E-sphingosine	LMSP01080004

Sphinganine and sphingosine phosphates (8)			
MxP® Quant 500 XL kit lipid annotation	Potential isobars within ± 0.5 Da	Potential isomers	Data base ID
SPBP d14:0	SPBP d14:0	-	-
SPBP d14:1	SPBP d14:1	-	-
SPBP d16:0	SPBP 16:0;O2	C16 Sphinganine-1-phosphate	LMSP01050006
SPBP d16:1	SPBP 16:1;O2	C16 Sphingosine-1-phosphate	LMSP01050005
SPBP d17:0	SPBP 17:0;O2	C17 Sphinganine-1-phosphate	LMSP01050008
SPBP d17:1	SPBP 17:1;O2	C17 Sphingosine-1-phosphate	LMSP01050007
SPBP d18:0	SPBP 18:0;O2	Sphinganine-phosphate	LMSP01050002
SPBP d18:1	SPBP 18:1;O2	Sphingosine-1-phosphate	LMSP01050001

Sphingomyelins (15)			
MxP® Quant 500 XL kit lipid annotation	Potential isobars within ± 0.5 Da	Potential isomers	Data base ID
SM 33:1	SM 33:1	SM(d16:1/17:0)	LMSP03010037
		SM(d18:1/15:0)	LMSP03010038
SM 34:1	SM 34:1	SM(d16:1/18:0)	LMSP03010042
		SM(d17:1/17:0)	LMSP03010043
		SM(d18:1/16:0)	LMSP03010003
SM 34:2	SM 34:2	SM(d16:1/18:1)	LMSP03010040
		SM(d18:1/16:1)	LMSP03010041
		SM(d18:2/16:0)	LMSP03010090
SM 35:1	SM 35:1	SM(d18:1/17:0)	LMSP03010044
		SM(d19:1/16:0)	LMSP03010045
SM 36:1	SM 36:1	SM(d16:1/20:0)	LMSP03010052
		SM(d18:0/18:1)	LMSP03010031
		SM(d18:1/18:0)	LMSP03010001
	[^{13}C] PC 32:2	-	-

MxP® Quant 500 XL kit lipid annotation	Potential isobars within ± 0.5 Da	Potential isomers	Data base ID
SM 36:2	SM 36:2	SM(d16:1/20:1)	LMSP03010048
		SM(d18:0/18:2)	LMSP03010049
		SM(d18:1/18:1)	LMSP03010029
		SM(d18:2/18:0)	LMSP03010050
		SM(d19:1/17:1)	LMSP03010051
SM 38:3	SM 38:3	SM(d18:1/20:2)	-
		SM(d18:2/20:1)	LMSP03010057
SM 40:4	SM 40:4	SM(d18:1/22:3)	-
		SM(d18:2/22:2)	-
SM 41:1	SM 41:1	SM(d16:1/25:0)	LMSP03010076
		SM(d17:1/24:0)	LMSP03010077
		SM(d18:1/23:0)	LMSP03010078
SM 41:2	SM 41:2	SM(d17:1/24:1)	LMSP03010074
		SM(d18:2/23:0)	LMSP03010075
SM 42:1	SM 42:1	SM(d18:0/24:1)	LMSP03010023
		SM(d18:1/24:0)	LMSP03010008
	[^{13}C] PC 38:2	-	-
SM 42:2	SM 42:2	SM(d18:1/24:1)	LMSP03010007
		SM(d18:2/24:0)	LMSP03010081
SM 43:1	SM 43:1	SM(d18:1/25:0)	LMSP03010027
		SM(d19:0/24:1)	LMSP03010085
		SM(d19:1/24:0)	LMSP03010086
		SM(d20:1/23:0)	LMSP03010087
SM 44:1	SM 44:1	SM(d18:1/26:0)	LMSP03010010
		SM(d18:0/26:1)	LMSP03010025
		SM(d20:0/24:1)	LMSP03010088
SM 44:2	SM 44:2	SM(d18:1/26:1)	LMSP03010009

Ceramides (29)		
MxP® Quant 500 XL kit lipid annotation	Potential isomers	Data base ID
Cer d16:1/18:0	Cer(d16:1/18:0)	LMSP02010046
Cer d16:1/20:0	Cer(d16:1/20:0)	LMSP02010047
Cer d16:1/22:0	Cer(d16:1/22:0)	LMSP02010016
Cer d16:1/23:0	Cer(d16:1/23:0)	LMSP02010017
Cer d16:1/24:0	Cer(d16:1/24:0)	LMSP02010050
Cer d18:0/16:0	Cer(d18:0/16:0)	LMSP02020001
Cer d18:1/14:0	Cer(d18:1/14:0)	LMSP02010001
Cer d18:1/16:0	Cer(d18:1/16:0)	LMSP02010004
Cer d18:1/18:0-OH	Cer(d18:1/18:0(2OH))	CHEBI 76754
Cer d18:1/18:0	Cer(d18:1/18:0)	LMSP02010006
Cer d18:1/18:1	Cer(d18:1/18:1(9Z))	LMSP02010003
Cer d18:1/20:0-OH	Cer(d18:1(8Z)/20:0(2OH[R]))	LMSP020101001
	Cer(d18:1(8E)/20:0(2OH[R]))	LMSP02010102
Cer d18:1/20:0	Cer(d18:1/20:0)	LMSP02010007
Cer d18:1/22:0	Cer(d18:1/22:0)	LMSP02010008
Cer d18:1/23:0	Cer(d18:1/23:0)	LMSP02010021
Cer d18:1/24:0	Cer(d18:1/24:0)	LMSP02010012
Cer d18:1/24:1	Cer(d18:1/24:1(15Z))	LMSP02010009
Cer d18:1/25:0	Cer(d18:1/25:0)	LMSP02010013
Cer d18:1/26:0	Cer(d18:1/26:0)	LMSP02010011
Cer d18:1/26:1	Cer(d18:1/26:1(17Z))	LMSP02010010
Cer d18:2/14:0	Cer(d18:2/14:0)	LMSP02010022

MxP® Quant 500 XL kit lipid annotation	Potential isomers	Data base ID
Cer d18:2/16:0	Cer(d18:2/16:0)	LMSP02010024
Cer d18:2/18:0	Cer(d18:2/18:0)	CHEBI 136461
Cer d18:2/18:1	Cer(d18:2/18:1)	LMSP02010025
Cer d18:2/20:0	Cer(d18:2/20:0)	LMSP02010026
Cer d18:2/22:0	Cer(d18:2/22:0)	LMSP02010029
Cer d18:2/23:0	Cer(d18:2/23:0)	LMSP02010030
Cer d18:2/24:0	Cer(d18:2/24:0)	PubChem 134783616
Cer d18:2/24:1	Cer(d18:2/24:1)	PubChem 134765416
CerP d18:1/16:0	CerP(d18:1/16:0)	LMSP02050002

Dihydroceramides (8)		
MxP® Quant 500 XL kit lipid annotation	Potential isomers	Data base ID
Cer d18:0/18:0-OH	Cer(d18:0/18:0(2OH))	LMSP02020030
Cer d18:0/18:0	Cer(d18:0/18:0)	LMSP02020008
Cer d18:0/20:0	Cer(d18:0/20:0)	LMSP02020009
Cer d18:0/22:0	Cer(d18:0/22:0)	LMSP02020010
Cer d18:0/24:0	Cer(d18:0/24:0)	LMSP02020012
Cer d18:0/24:1	Cer(d18:0/24:1(15Z))	LMSP02020011
Cer d18:0/26:1-OH	-	-
Cer d18:0/26:1	Cer(d18:0/26:1(17Z))	LMSP02020013

Hexosylceramides (19)		
MxP® Quant 500 XL kit lipid annotation	Potential isomers	Data base ID
Hex-Cer d16:1/20:0	GlcCer(d16:1/20:0)	LMSP0501AA50
Hex-Cer d16:1/22:0	GlcCer(d16:1/22:0)	LMSP0501AA30
	GalCer(d16:1/22:0)	LMSP0501AC09
Hex-Cer d16:1/24:0	GlcCer(d16:1/24:0)	LMSP0501AA51
Hex-Cer d18:1/14:0	GlcCer(d18:1/14:0)	LMSP0501AA26
Hex-Cer d18:1/16:0	GlcCer(d18:1/16:0)	LMSP0501AA03
	GalCer(d18:1/16:0)	LMSP0501AC01
Hex-Cer d18:1/18:0	GlcCer(d18:1/18:0)	LMSP0501AA05
	GalCer(d18:1/18:0)	LMSP0501AC02
Hex-Cer d18:1/18:1	GlcCer(d18:1/18:1(9Z))	LMSP0501AA27
Hex-Cer d18:1/20:0	GlcCer(d18:1/20:0)	LMSP0501AA06
	GalCer(d18:1/20:0)	LMSP0501AC03
Hex-Cer d18:1/22:0	GlcCer(d18:1/22:0)	LMSP0501AA07
	GalCer(d18:1/22:0)	LMSP0501AC04
Hex-Cer d18:1/23:0	GlcCer(d18:1/23:0)	LMSP0501AA32
	GalCer(d18:1/23:0)	LMSP0501AC17
Hex-Cer d18:1/24:0	GlcCer(d18:1/24:0)	LMSP0501AA09
	GalCer(d18:1/24:0)	LMSP0501AC05
Hex-Cer d18:1/24:1	GlcCer(d18:1/24:1(15Z))	LMSP0501AA08
	GalCer(d18:1/24:1(15Z))	LMSP0501AC07
Hex-Cer d18:1/26:0	GlcCer(d18:1/26:0)	LMSP0501AA11
	GalCer(d18:1/26:0)	LMSP0501AC06
Hex-Cer d18:1/26:1	GlcCer(d18:1/26:1(17Z))	LMSP0501AA10
	GalCer(d18:1/26:1(17Z))	LMSP0501AC08
Hex-Cer d18:2/16:0	GlcCer(d18:2/16:0)	LMSP0501AA33
	GalCer(d18:2/16:0)	LMSP0501AC18

MxP® Quant 500 XL kit lipid annotation	Potential isomers	Data base ID
Hex-Cer d18:2/18:0	N-[(2S,3R,4E,8E)-3-hydroxy-1-[(2R,3R,4S,5S,6R)-3,4,5-trihydroxy-6-(hydroxymethyl)oxan-2-yl]oxyoctadeca-4,8-dien-2-yl]octadecanamide	CID 10146923
Hex-Cer d18:2/20:0	GlcCer(d18:2/20:0)	LMSP0501AA34
	GalCer(d18:2/20:0)	LMSP0501AC19
Hex-Cer d18:2/22:0	GlcCer(d18:2/22:0)	LMSP0501AA37
	GalCer(d18:2/22:0)	LMSP0501AC22
Hex-Cer d18:2/23:0	GlcCer(d18:2/23:0)	LMSP0501AA38
	GalCer(d18:2/23:0)	LMSP0501AC23
Hex-Cer d18:2/24:0	N-[(2S,3R,4E,8E)-3-hydroxy-1-[(2S,5R,6S)-3,4,5-trihydroxy-6-(hydroxymethyl)oxan-2-yl]oxyoctadeca-4,8-dien-2-yl]tetracosanamide	CID 134765308

Dihexosylceramides (9)		
MxP® Quant 500 XL kit lipid annotation	Potential isomers	Data base ID
Hex2Cer d18:1/14:0	LacCer(d18:1/14:0)	LMSP0501AB12
Hex2Cer d18:1/16:0	LacCer(d18:1/16:0)	LMSP0501AB03
	Manβ1-4Glcβ-Cer(d18:1/16:0)	LMSP0501AD01
	Galα1-4Galβ-Cer(d18:1/16:0)	LMSP0509AA01
Hex2Cer d18:1/18:0	LacCer(d18:1/18:0)	LMSP0501AB04
	Manβ1-4Glcβ-Cer(d18:1/18:0)	LMSP0501AD02
	Galα1-4Galβ-Cer(d18:1/18:0)	LMSP0509AA02
Hex2Cer d18:1/20:0	LacCer(d18:1/20:0)	LMSP0501AB05
	Manβ1-4Glcβ-Cer(d18:1/20:0)	LMSP0501AD03
	Galα1-4Galβ-Cer(d18:1/20:0)	LMSP0509AA03
Hex2Cer d18:1/22:0	LacCer(d18:1/22:0)	LMSP0501AB06
	Manβ1-4Glcβ-Cer(d18:1/22:0)	LMSP0501AD04
	Galα1-4Galβ-Cer(d18:1/22:0)	LMSP0509AA04
Hex2Cer d18:1/24:0	LacCer(d18:1/24:0)	LMSP0501AB07
	Manβ1-4Glcβ-Cer(d18:1/24:0)	LMSP0501AD05
	Galα1-4Galβ-Cer(d18:1/24:0)	LMSP0509AA05
Hex2Cer d18:1/24:1	LacCer(d18:1/24:1(15Z))	LMSP0501AB09
	Manβ1-4Glcβ-Cer(d18:1/24:1(15Z))	LMSP0501AD07
	Galα1-4Galβ-Cer(d18:1/24:1(15Z))	LMSP0509AA07
Hex2Cer d18:1/26:0	LacCer(d18:1/26:0)	LMSP0501AB08
	Manβ1-4Glcβ-Cer(d18:1/26:0)	LMSP0501AD06
	Galα1-4Galβ-Cer(d18:1/26:0)	LMSP0509AA06
Hex2Cer d18:1/26:1	LacCer(d18:1/26:1(17Z))	LMSP0501AB10
	Manβ1-4Glcβ-Cer(d18:1/26:1(17Z))	LMSP0501AD08
	Galα1-4Galβ-Cer(d18:1/26:1(17Z))	LMSP0509AA08

Trihexosylceramides (6)		
MxP® Quant 500 XL kit lipid annotation	Potential isomers	Data base ID
Hex3Cer d18:1/16:0	Galα1-4Galβ1-4Glcβ-Cer(d18:1/16:0)	LMSP0502AA01
	Galα1-3Galβ1-4Glcβ-Cer(d18:1/16:0)	LMSP0505CZ01
	Manα1-3Manβ1-4Glcβ-Cer(d18:1/16:0)	LMSP0507AA01
Hex3Cer d18:1/18:0	Galα1-4Galβ1-4Glcβ-Cer(d18:1/18:0)	LMSP0502AA02
	Galα1-3Galβ1-4Glcβ-Cer(d18:1/18:0)	LMSP0505CZ02
	Manα1-3Manβ1-4Glcβ-Cer(d18:1/18:0)	LMSP0507AA02

MxP® Quant 500 XL kit lipid annotation	Potential isomers	Data base ID
Hex3Cer d18:1/20:0	Galα1-4Galβ1-4Glcβ-Cer(d18:1/20:0)	LMSP0502AA03
	Galα1-3Galβ1-4Glcβ-Cer(d18:1/20:0)	LMSP0505CZ03
	Manα1-3Manβ1-4Glcβ-Cer(d18:1/20:0)	LMSP0507AA03
Hex3Cer d18:1/22:0	Galα1-4Galβ1-4Glcβ-Cer(d18:1/22:0)	LMSP0502AA04
	Galα1-3Galβ1-4Glcβ-Cer(d18:1/22:0)	LMSP0505CZ04
	Manα1-3Manβ1-4Glcβ-Cer(d18:1/22:0)	LMSP0507AA04
Hex3Cer d18:1/24:1	Galα1-4Galβ1-4Glcβ-Cer(d18:1/24:1(15Z))	LMSP0502AA07
	Galα1-3Galβ1-4Glcβ-Cer(d18:1/24:1(15Z))	LMSP0505CZ07
	Manα1-3Manβ1-4Glcβ-Cer(d18:1/24:1(15Z))	LMSP0507AA07
Hex3Cer d18:1/26:1	Galα1-4Galβ1-4Glcβ-Cer(d18:1/26:1(17Z))	LMSP0502AA08
	Galα1-3Galβ1-4Glcβ-Cer(d18:1/26:1(17Z))	LMSP0505CZ08
	Manα1-3Manβ1-4Glcβ-Cer(d18:1/26:1(17Z))	LMSP0507AA08

Cholesteryl esters (22)		
MxP® Quant 500 XL kit lipid annotation	Potential isomers	Data base ID
CE 14:0	14:0 Cholesteryl ester	LMST01020004
CE 14:1	14:1 Cholesteryl ester	LMST01020021
CE 15:0	15:0 Cholesteryl ester	LMST01020027
CE 15:1	15:1 Cholesteryl ester	LMST01020022
CE 16:0	16:0 Cholesteryl ester	LMST01020005
CE 16:1	16:1 Cholesteryl ester	LMST01020006
	16:1 Cholesteryl ester	LMST01020041
CE 17:0	17:0 Cholesteryl ester	LMST01020026
CE 17:1	17:1 Cholesteryl ester	LMST01020023
CE 18:0	18:0 Cholesteryl ester	LMST01020007
CE 18:1	18:1 Cholesteryl ester	LMST01020003
CE 18:2	18:2 Cholesteryl ester	LMST01020008
CE 18:3	18:3 Cholesteryl ester	LMST01020009
CE 20:0	20:0 Cholesteryl ester	LMST01020010
CE 20:1	20:1 Cholesteryl ester	LMST01020011
CE 20:3	20:3 Cholesteryl ester	LMST01020013
CE 20:4	20:4 Cholesteryl ester	LMST01020014
CE 20:5	20:5 Cholesteryl ester	LMST01020015
CE 22:0	22:0 Cholesteryl ester	LMST01020016
CE 22:1	22:1 Cholesteryl ester	LMST01020025
CE 22:2	22:2 Cholesteryl ester	LMST01020017
CE 22:5	22:5 Cholesteryl ester	LMST01020031
CE 22:6	22:6 Cholesteryl ester	LMST01020019

Monoglycerides (12)			
MxP® Quant 500 XL kit lipid annotation	Potential isobars within ± 0.5 Da	Potential isomers	Data base ID
MG 16:1	MG 16:1	MG(16:1(9Z)/0:0/0:0)	PubChem:53480976
		MG(16:1(9Z)/0:0/0:0)	PubChem:9883914
		MG(0:0/16:1(9Z)/0:0)	PubChem:53480960
MG 18:1	MG 18:1	MG(18:1(11E)/0:0/0:0)[rac]	LMGL01010004
		MG(0:0/18:1(9Z)/0:0)	LMGL01010024
		MG(18:1(9Z)/0:0/0:0)[rac]	LMGL01010005
MG 18:2	MG 18:2	MG(18:2(9Z,12Z)/0:0/0:0)[rac]	LMGL01010006

MxP® Quant 500 XL kit lipid annotation	Potential isobars within ± 0.5 Da	Potential isomers	Data base ID
MG 18:3	MG 18:3	MG(18:3(6Z,9Z,12Z)/0:0/0:0)	PubChem:53480978
MG 20:1	MG 20:1	MAG(20:1n9/0:0)	PubChem:53480982
		MG(0:0/20:1(11Z)/0:0)	PubChem:53480963
MG 20:3	MG 20:3	Sciadonoyl-2-glycerol	LMGL01010036
MG 20:4	MG 20:4	MG(0:0/20:4(5Z,8Z,11Z,14Z)/0:0)	LMGL01010023
MG 20:5	MG 20:5	MG(0:0/20:5(5Z,8Z,11Z,14Z,17Z)/0:0)	LMGL01010026
MG 22:1	MG 22:1	MG(21:1(5Z)20Me))	LMGP01050153
MG 22:2	MG 22:2	MG 22:2	PubChem:134769740
		MG(22:2(13Z,16Z)/0:0/0:0)	PubChem:53480991
		MG(0:0/22:2(13Z,16Z)/0:0)	PubChem:53480968
MG 22:4	MG 22:4	MG(22:4(7Z,10Z,13Z,16Z)/0:0/0:0)	PubChem:53480992
		MG(0:0/22:4(7Z,10Z,13Z,16Z)/0:0)	PubChem:53480969
		MG 22:4	PubChem:134769856
MG 22:6	MG 22:6	MG(0:0/22:6(4Z,7Z,10Z,13Z,16Z,19Z)/0:0)	LMGL01010027

Diglycerides (44)		
**MxP® Quant 500 XL kit lipid annotation**	**Potential isomers**	**Data base ID**
DG 14:0_14:0	1,2-dimyristoyl-sn-glycerol	LMGL02010321
DG 14:0_18:1	DG(14:0/18:1(9Z)/0:0)[iso2]	LMGL02010384
DG 14:0_18:2	DG(14:0/18:2(9Z,12Z)/0:0)[iso2]	LMGL02010385
DG 14:0_20:0	DG(14:0/20:0/0:0)[iso2]	LMGL02010388
DG 14:1_18:1	DG(14:1(9Z)/18:1(9Z)/0:0)[iso2]	LMGL02010408
DG 14:1_20:2	DG(14:1(9Z)/20:2(11Z,14Z)/0:0)[iso2]	LMGL02010414
DG 16:0_16:0	DG(16:0/16:0/0:0)[rac]	LMGL02010001
	DG(16:0/16:0/0:0)	LMGL02010009
DG 16:0_16:1	DG(16:0/16:1(9Z)/0:0)[iso2]	LMGL02010010
DG 16:0_18:1	DG(18:1(11E)/16:0/0:0)	LMGL02010004
	DG(18:1(9Z)/16:0/0:0)	LMGL02010005
	DG(16:0/18:1(9Z)/0:0)	LMGL02010006
	DG(16:0/18:1(11Z)/0:0)	LMGL02010307
DG 16:0_18:2	DG(16:0/18:2(9Z,12Z)/0:0)[iso2]	LMGL02010027
DG 16:0_20:0	DG(16:0/20:0/0:0)[iso2]	LMGL02010042
DG 16:0_20:3	DG(16:0/20:3(8Z,11Z,14Z)/0:0)[iso2]	LMGL02010062
DG 16:0_20:4	DG(16:0/20:4(5Z,8Z,11Z,14Z)/0:0)[iso2]	LMGL02010070
DG 16:1_18:0	DG(16:1(9Z)/18:0/0:0)[iso2]	LMGL02010023
DG 16:1_18:1	DG(16:1(9Z)/18:1(9Z)/0:0)[iso2]	LMGL02010026
DG 16:1_18:2	DG(16:1(9Z)/18:2(9Z,12Z)/0:0)[iso2]	LMGL02010031
DG 16:1_20:0	DG(16:1(9Z)/20:0/0:0)[iso2]	LMGL02010047
DG 17:0_17:1	DG(17:0/17:1(9Z)/0:0)[iso2]	LMGL02010018
DG 17:0_18:1	DG(17:0/18:1(9Z)/0:0)[iso2]	LMGL02010030
DG 18:0_20:0	DG(20:0/18:0)	LMGL02010008
	DG(18:0/20:0/0:0)[iso2]	LMGL02010073
DG 18:0_20:4	DG(18:0/20:4(5Z,8Z,11Z,14Z)/0:0)[iso2]	LMGL02010111
DG 18:1_18:1	DG(18:1(9Z)/18:1(9Z)/0:0)	LMGL02010049
DG 18:1_18:2	DG(18:1(9Z)/18:2(9Z,12Z)/0:0)[iso2]	LMGL02010056
DG 18:1_18:3	DG(18:1(9Z)/18:3(9Z,12Z,15Z)/0:0)[iso2]	LMGL02010064
	DG(18:3(6Z,9Z,12Z)/18:1(9Z)/0:0)[iso2]	LMGL02010480
DG 18:1_18:4	DG(18:4(6Z,9Z,12Z,15Z)/18:1(9Z)/0:0)[iso2]	LMGL02010504

MxP® Quant 500 XL kit lipid annotation	Potential isomers	Data base ID
DG 18:1_20:0	DG(18:1(9Z)/20:0/0:0)[iso2]	LMGL02010081
DG 18:1_20:1	DG(18:1(9Z)/20:1(11Z)/0:0)[iso2]	LMGL02010090
DG 18:1_20:2	DG(18:1(9Z)/20:2(11Z,14Z)/0:0)[iso2]	LMGL02010100
DG 18:1_20:3	DG(18:1(9Z)/20:3(8Z,11Z,14Z)/0:0)[iso2]	LMGL02010110
DG 18:1_20:4	DG(18:1(9Z)/20:4(5Z,8Z,11Z,14Z)/0:0)[iso2]	LMGL02010121
DG 18:1_22:5	DG(18:1(9Z)/22:5(7Z,10Z,13Z,16Z,19Z)/0:0)[iso2]	LMGL02010215
DG 18:1_22:6	DG(18:1(9Z)/22:6(4Z,7Z,10Z,13Z,16Z,19Z)/0:0)[iso2]	LMGL02010225
DG 18:2_18:2	DG(18:2(9Z,12Z)/18:2(9Z,12Z)/0:0)	LMGL02010063
DG 18:2_18:3	DG(18:2(9Z,12Z)/18:3(9Z,12Z,15Z)/0:0)[iso2]	LMGL02010071
	DG(18:3(6Z,9Z,12Z)/18:2(9Z,12Z)/0:0)[iso2]	LMGL02010481
DG 18:2_18:4	DG(18:4(6Z,9Z,12Z,15Z)/18:2(9Z,12Z)/0:0)[iso2]	LMGL02010505
DG 18:2_20:0	DG(18:2(9Z,12Z)/20:0/0:0)[iso2]	LMGL02010089
DG 18:2_20:4	DG(18:2(9Z,12Z)/20:4(5Z,8Z,11Z,14Z)/0:0)[iso2]	LMGL02010131
DG 18:3_18:3	DG(18:3(9Z,12Z,15Z)/18:3(9Z,12Z,15Z)/0:0)	LMGL02010079
	DG(18:3(6Z,9Z,12Z)/18:3(6Z,9Z,12Z)/0:0)	LMGL02010328
	DG(18:3(6Z,9Z,12Z)/18:3(9Z,12Z,15Z)/0:0)[iso2]	LMGL02010482
DG 18:3_20:2	DG(18:3(9Z,12Z,15Z)/20:2(11Z,14Z)/0:0)[iso2]	LMGL02010119
	DG(18:3(6Z,9Z,12Z)/20:2(11Z,14Z)/0:0)[iso2]	LMGL02010486
DG 21:0_22:6	DG(21:0/22:6(4Z,7Z,10Z,13Z,16Z,19Z)/0:0)[iso2]	LMGL02010290
DG 22:1_22:2	DG(22:1(13Z)/22:2(13Z,16Z)/0:0)[iso2]	LMGL02010283
DG O-14:0_18:2	DG(P-14:0/18:1(9Z))	LMGL02040001
DG O-16:0_18:1	DG(O-16:0/18:1(9Z))	LMGL02020001
DG O-16:0_20:4	1-(14-methyl-pentadecanoyl)-2-(8-[3]-ladderane-octanyl)-sn-glycerol	LMGL02070004
DG O-18:2_18:2	-	-

Triglycerides (242)		
MxP® Quant 500 XL kit lipid annotation	Potential isomers	Data base ID
TG 14:0_32:2	TG(14:0/14:0/18:2(9Z,12Z))[iso3]	LMGL03012763
	TG(14:0/16:1(9Z)/16:1(9Z))[iso3]	LMGL03012787
	TG(12:0/14:0/20:2(11Z,14Z))[iso6]	LMGL03013226
	TG(14:0/14:1(9Z)/18:1(9Z))[iso6]	LMGL03014145
	TG(14:0/15:0/17:2(9Z,12Z))[iso6]	LMGL03014171
	TG(14:0/15:1(9Z)/17:1(9Z))[iso6]	LMGL03014197
TG 14:0_34:0	TG(14:0/14:0/20:0)[iso3]	LMGL03012769
	TG(14:0/17:0/17:0)[iso3]	LMGL03012788
	TG(12:0/14:0/22:0)[iso6]	LMGL03013231
	TG(13:0/14:0/21:0)[iso6]	LMGL03013694
	TG(14:0/15:0/19:0)[iso6]	LMGL03014178
	TG(14:0/16:0/18:0)[iso6]	LMGL03014225
TG 14:0_34:1	TG(14:0/14:0/20:1(11Z))[iso3]	LMGL03012770
	TG(12:0/14:0/22:1(11Z))[iso6]	LMGL03013232
	TG(14:0/14:1(9Z)/20:0)[iso6]	LMGL03014152
	TG(14:0/15:0/19:1(9Z))[iso6]	LMGL03014179
	TG(14:0/15:1(9Z)/19:0)[iso6]	LMGL03014205
	TG(14:0/16:0/18:1(9Z))[iso6]	LMGL03014226
	TG(14:0/16:1(9Z)/18:0)[iso6]	LMGL03014250
	TG(14:0/17:0/17:1(9Z))[iso6]	LMGL03014272

MxP® Quant 500 XL kit lipid annotation	Potential isomers	Data base ID
TG 14:0_34:2	TG(14:0/14:0/20:2(11Z,14Z))[iso3]	LMGL03012771
	TG(14:0/17:1(9Z)/17:1(9Z))[iso3]	LMGL03012789
	TG(12:0/14:0/22:2(13Z,16Z))[iso6]	LMGL03013233
	TG(14:0/14:1(9Z)/20:1(11Z))[iso6]	LMGL03014153
	TG(14:0/15:1(9Z)/19:1(9Z))[iso6]	LMGL03014206
	TG(14:0/16:0/18:2(9Z,12Z))[iso6]	LMGL03014227
	TG(14:0/16:1(9Z)/18:1(9Z))[iso6]	LMGL03014251
	TG(14:0/17:0/17:2(9Z,12Z))[iso6]	LMGL03014273
TG 14:0_34:3	TG(14:0/14:0/20:3(8Z,11Z,14Z))[iso3]	LMGL03012772
	TG(12:0/14:0/22:3(10Z,13Z,16Z))[iso6]	LMGL03013234
	TG(14:0/14:1(9Z)/20:2(11Z,14Z))[iso6]	LMGL03014154
	TG(14:0/16:0/18:3(6Z,9Z,12Z))[iso6]	LMGL03014228
	TG(14:0/16:0/18:3(9Z,12Z,15Z))[iso6]	LMGL03014229
	TG(14:0/16:1(9Z)/18:2(9Z,12Z))[iso6]	LMGL03014252
	TG(14:0/17:1(9Z)/17:2(9Z,12Z))[iso6]	LMGL03014296
TG 14:0_35:1	TG(14:0/18:4(6Z,9Z,12Z,15Z)/18:4(6Z,9Z,12Z,15Z))[iso3]	LMGL03012796
	TG(13:0/14:0/22:1(11Z))[iso6]	LMGL03013696
	TG(14:0/14:1(9Z)/21:0)[iso6]	LMGL03014158
	TG(14:0/15:0/20:1(11Z))[iso6]	LMGL03014181
	TG(14:0/15:1(9Z)/20:0)[iso6]	LMGL03014207
	TG(14:0/16:0/19:1(9Z))[iso6]	LMGL03014232
	TG(14:0/16:1(9Z)/19:0)[iso6]	LMGL03014256
	TG(14:0/17:0/18:1(9Z))[iso6]	LMGL03014275
	TG(14:0/17:1(9Z)/18:0)[iso6]	LMGL03014297
TG 14:0_35:2	TG(13:0/14:0/22:2(13Z,16Z))[iso6]	LMGL03013697
	TG(14:0/15:0/20:2(11Z,14Z))[iso6]	LMGL03014182
	TG(14:0/15:1(9Z)/20:1(11Z))[iso6]	LMGL03014208
	TG(14:0/16:1(9Z)/19:1(9Z))[iso6]	LMGL03014257
	TG(14:0/17:0/18:2(9Z,12Z))[iso6]	LMGL03014276
	TG(14:0/17:1(9Z)/18:1(9Z))[iso6]	LMGL03014298
	TG(14:0/17:2(9Z,12Z)/18:0)[iso6]	LMGL03014319
TG 14:0_36:1	TG(14:0/14:0/22:1(11Z))[iso3]	LMGL03012777
	TG(14:0/14:1(9Z)/22:0)[iso6]	LMGL03014159
	TG(14:0/15:1(9Z)/21:0)[iso6]	LMGL03014213
	TG(14:0/16:0/20:1(11Z))[iso6]	LMGL03014234
	TG(14:0/16:1(9Z)/20:0)[iso6]	LMGL03014258
	TG(14:0/17:0/19:1(9Z))[iso6]	LMGL03014281
	TG(14:0/17:1(9Z)/19:0)[iso6]	LMGL03014303
	TG(14:0/18:0/18:1(9Z))[iso6]	LMGL03014341
TG 14:0_36:2	TG(14:0/14:0/22:2(13Z,16Z))[iso3]	LMGL03012778
	TG(14:0/18:1(9Z)/18:1(9Z))[iso3]	LMGL03012792
	TG(14:0/14:1(9Z)/22:1(11Z))[iso6]	LMGL03014160
	TG(14:0/16:0/20:2(11Z,14Z))[iso6]	LMGL03014235
	TG(14:0/16:1(9Z)/20:1(11Z))[iso6]	LMGL03014259
	TG(14:0/17:1(9Z)/19:1(9Z))[iso6]	LMGL03014304
	TG(14:0/17:2(9Z,12Z)/19:0)[iso6]	LMGL03014325
	TG(14:0/18:0/18:2(9Z,12Z))[iso6]	LMGL03014342
TG 14:0_36:3	TG(14:0/14:0/22:3(10Z,13Z,16Z))[iso3]	LMGL03012779
	TG(14:0/14:1(9Z)/22:2(13Z,16Z))[iso6]	LMGL03014161
	TG(14:0/16:0/20:3(8Z,11Z,14Z))[iso6]	LMGL03014236
	TG(14:0/16:1(9Z)/20:2(11Z,14Z))[iso6]	LMGL03014260
	TG(14:0/17:2(9Z,12Z)/19:1(9Z))[iso6]	LMGL03014326
	TG(14:0/18:0/18:3(6Z,9Z,12Z))[iso6]	LMGL03014343
	TG(14:0/18:0/18:3(9Z,12Z,15Z))[iso6]	LMGL03014344
	TG(14:0/18:1(9Z)/18:2(9Z,12Z))[iso6]	LMGL03014362

MxP® Quant 500 XL kit lipid annotation	Potential isomers	Data base ID
TG 14:0_36:4	TG(14:0/14:0/22:4(7Z,10Z,13Z,16Z))[iso3]	LMGL03012780
	TG(14:0/18:2(9Z,12Z)/18:2(9Z,12Z))[iso3]	LMGL03012793
	TG(14:0/14:1(9Z)/22:3(10Z,13Z,16Z))[iso6]	LMGL03014162
	TG(14:0/16:0/20:4(5Z,8Z,11Z,14Z))[iso6]	LMGL03014237
	TG(14:0/16:1(9Z)/20:3(8Z,11Z,14Z))[iso6]	LMGL03014261
	TG(14:0/18:0/18:4(6Z,9Z,12Z,15Z))[iso6]	LMGL03014345
	TG(14:0/18:1(9Z)/18:3(6Z,9Z,12Z))[iso6]	LMGL03014363
	TG(14:0/18:1(9Z)/18:3(9Z,12Z,15Z))[iso6]	LMGL03014364
TG 14:0_38:4	TG(14:0/16:0/22:4(7Z,10Z,13Z,16Z))[iso6]	LMGL03014244
	TG(14:0/16:1(9Z)/22:3(10Z,13Z,16Z))[iso6]	LMGL03014268
	TG(14:0/18:0/20:4(5Z,8Z,11Z,14Z))[iso6]	LMGL03014352
	TG(14:0/18:1(9Z)/20:3(8Z,11Z,14Z))[iso6]	LMGL03014371
	TG(14:0/18:2(9Z,12Z)/20:2(11Z,14Z))[iso6]	LMGL03014389
	TG(14:0/18:3(6Z,9Z,12Z)/20:1(11Z))[iso6]	LMGL03014406
	TG(14:0/18:3(9Z,12Z,15Z)/20:1(11Z))[iso6]	LMGL03014423
	TG(14:0/18:4(6Z,9Z,12Z,15Z)/20:0)[iso6]	LMGL03014438
TG 14:0_38:5	TG(14:0/16:0/22:5(7Z,10Z,13Z,16Z,19Z))[iso6]	LMGL03014245
	TG(14:0/16:1(9Z)/22:4(7Z,10Z,13Z,16Z))[iso6]	LMGL03014269
	TG(14:0/18:0/20:5(5Z,8Z,11Z,14Z,17Z))[iso6]	LMGL03014353
	TG(14:0/18:1(9Z)/20:4(5Z,8Z,11Z,14Z))[iso6]	LMGL03014372
	TG(14:0/18:2(9Z,12Z)/20:3(8Z,11Z,14Z))[iso6]	LMGL03014390
	TG(14:0/18:3(6Z,9Z,12Z)/20:2(11Z,14Z))[iso6]	LMGL03014407
	TG(14:0/18:3(9Z,12Z,15Z)/20:2(11Z,14Z))[iso6]	LMGL03014424
	TG(14:0/18:4(6Z,9Z,12Z,15Z)/20:1(11Z))[iso6]	LMGL03014439
TG 14:0_39:3	TG(14:0/20:5(5Z,8Z,11Z,14Z,17Z)/20:5(5Z,8Z,11Z,14Z,17Z))[iso3]	LMGL03012804
	TG(14:0/17:0/22:3(10Z,13Z,16Z))[iso6]	LMGL03014292
	TG(14:0/17:1(9Z)/22:2(13Z,16Z))[iso6]	LMGL03014314
	TG(14:0/17:2(9Z,12Z)/22:1(11Z))[iso6]	LMGL03014335
	TG(14:0/18:3(6Z,9Z,12Z)/21:0)[iso6]	LMGL03014411
	TG(14:0/18:3(9Z,12Z,15Z)/21:0)[iso6]	LMGL03014428
	TG(14:0/18:4(6Z,9Z,12Z,15Z)/22:6(4Z,7Z,10Z,13Z,16Z,19Z))[iso6]	LMGL03014451
	TG(14:0/19:0/20:3(8Z,11Z,14Z))[iso6]	LMGL03014456
	TG(14:0/19:1(9Z)/20:2(11Z,14Z))[iso6]	LMGL03014469
TG 14:0_40:5	TG(14:0/18:0/22:5(7Z,10Z,13Z,16Z,19Z))[iso6]	LMGL03014360
	TG(14:0/18:1(9Z)/22:4(7Z,10Z,13Z,16Z))[iso6]	LMGL03014379
	TG(14:0/18:2(9Z,12Z)/22:3(10Z,13Z,16Z))[iso6]	LMGL03014397
	TG(14:0/18:4(6Z,9Z,12Z,15Z)/22:1(11Z))[iso6]	LMGL03014446
	TG(14:0/20:0/20:5(5Z,8Z,11Z,14Z,17Z))[iso6]	LMGL03014485
	TG(14:0/20:1(11Z)/20:4(5Z,8Z,11Z,14Z))[iso6]	LMGL03014496
	TG(14:0/20:2(11Z,14Z)/20:3(8Z,11Z,14Z))[iso6]	LMGL03014506
TG 16:0_28:1	TG(12:0/16:0/16:1(9Z))[iso6]	LMGL03013322
	TG(13:0/15:1(9Z)/16:0)[iso6]	LMGL03013759
	TG(14:0/14:1(9Z)/16:0)[iso6]	LMGL03014139
	PG(O-16:0/19:1(9Z))	LMGP04020011
	PG(P-16:0/19:0)	LMGP04030016
TG 16:0_28:2	TG(14:1(9Z)/14:1(9Z)/16:0)[iso3]	LMGL03012815
	PG(P-16:0/19:1(9Z))	LMGP04030017
TG 16:0_30:2	TG(15:1(9Z)/15:1(9Z)/16:0)[iso3]	LMGL03012927
	TG(12:0/16:0/18:2(9Z,12Z))[iso6]	LMGL03013327
	TG(13:0/16:0/17:2(9Z,12Z))[iso6]	LMGL03013789
	TG(14:1(9Z)/16:0/16:1(9Z))[iso6]	LMGL03014627
TG 16:0_32:0	TG(16:0/16:0/16:0)	LMGL03010001
	TG(12:0/16:0/20:0)[iso6]	LMGL03013333
	TG(13:0/16:0/19:0)[iso6]	LMGL03013796
	TG(14:0/16:0/18:0)[iso6]	LMGL03014225
	TG(15:0/16:0/17:0)[iso6]	LMGL03015006

MxP® Quant 500 XL kit lipid annotation	Potential isomers	Data base ID
TG 16:0_32:1	TG(16:0/16:0/16:1(9Z))[iso3]	LMGL03010017
	TG(12:0/16:0/20:1(11Z))[iso6]	LMGL03013334
	TG(13:0/16:0/19:1(9Z))[iso6]	LMGL03013797
	TG(14:0/16:0/18:1(9Z))[iso6]	LMGL03014226
	TG(14:1(9Z)/16:0/18:0)[iso6]	LMGL03014631
	TG(15:0/16:0/17:1(9Z))[iso6]	LMGL03015007
	TG(15:1(9Z)/16:0/17:0)[iso6]	LMGL03015357
TG 16:0_32:2	TG(16:0/16:1(9Z)/16:1(9Z))[iso3]	LMGL03010018
	TG(12:0/16:0/20:2(11Z,14Z))[iso6]	LMGL03013335
	TG(14:0/16:0/18:2(9Z,12Z))[iso6]	LMGL03014227
	TG(14:1(9Z)/16:0/18:1(9Z))[iso6]	LMGL03014632
	TG(15:0/16:0/17:2(9Z,12Z))[iso6]	LMGL03015008
	TG(15:1(9Z)/16:0/17:1(9Z))[iso6]	LMGL03015358
TG 16:0_32:3	TG(12:0/16:0/20:3(8Z,11Z,14Z))[iso6]	LMGL03013336
	TG(14:0/16:0/18:3(6Z,9Z,12Z))[iso6]	LMGL03014228
	TG(14:0/16:0/18:3(9Z,12Z,15Z))[iso6]	LMGL03014229
	TG(14:1(9Z)/16:0/18:2(9Z,12Z))[iso6]	LMGL03014633
	TG(15:1(9Z)/16:0/17:2(9Z,12Z))[iso6]	LMGL03015359
	16:0-Glc-Campesterol	LMST01031127
TG 16:0_33:1	TG(16:0/16:1(9Z)/17:0)[iso6]	LMGL03010021
	TG(16:0/16:0/17:1(9Z))[iso3]	LMGL03010022
	TG(13:0/16:0/20:1(11Z))[iso6]	LMGL03013799
	TG(14:0/16:0/19:1(9Z))[iso6]	LMGL03014232
	TG(14:1(9Z)/16:0/19:0)[iso6]	LMGL03014637
	TG(15:0/16:0/18:1(9Z))[iso6]	LMGL03015010
	TG(15:1(9Z)/16:0/18:0)[iso6]	LMGL03015360
TG 16:0_33:2	TG(16:0/16:1(9Z)/17:1(9Z))[iso6]	LMGL03010025
	TG(16:0/16:0/17:2(9Z,12Z))[iso3]	LMGL03010026
	TG(13:0/16:0/20:2(11Z,14Z))[iso6]	LMGL03013800
	TG(14:1(9Z)/16:0/19:1(9Z))[iso6]	LMGL03014638
	TG(15:0/16:0/18:2(9Z,12Z))[iso6]	LMGL03015011
	TG(15:1(9Z)/16:0/18:1(9Z))[iso6]	LMGL03015361
TG 16:0_34:0	TG(16:0/16:0/18:0)	LMGL03010004
	TG(16:0/17:0/17:0)[iso3]	LMGL03010023
	TG(12:0/16:0/22:0)[iso6]	LMGL03013340
	TG(13:0/16:0/21:0)[iso6]	LMGL03013804
	TG(14:0/16:0/20:0)[iso6]	LMGL03014233
	TG(15:0/16:0/19:0)[iso6]	LMGL03015015
TG 16:0_34:1	TG(16:0/16:0/18:1(11E))	LMGL03010005
	TG(16:0/16:0/18:1(9Z))	LMGL03010006
	TG(16:0/17:0/17:1(9Z))[iso6]	LMGL03010028
	TG(16:0/16:1(9Z)/18:0)[iso6]	LMGL03010036
	TG(12:0/16:0/22:1(11Z))[iso6]	LMGL03013341
	TG(14:0/16:0/20:1(11Z))[iso6]	LMGL03014234
	TG(14:1(9Z)/16:0/20:0)[iso6]	LMGL03014639
	TG(15:0/16:0/19:1(9Z))[iso6]	LMGL03015016
	TG(15:1(9Z)/16:0/19:0)[iso6]	LMGL03015366
TG 16:0_34:2	TG(16:0/17:1(9Z)/17:1(9Z))[iso3]	LMGL03010032
	TG(16:0/17:0/17:2(9Z,12Z))[iso6]	LMGL03010034
	TG(16:0/16:1(9Z)/18:1(9Z))[iso6]	LMGL03010043
	TG(16:0/16:0/18:2(9Z,12Z))[iso3]	LMGL03010044
	TG(12:0/16:0/22:2(13Z,16Z))[iso6]	LMGL03013342
	TG(14:0/16:0/20:2(11Z,14Z))[iso6]	LMGL03014235
	TG(14:1(9Z)/16:0/20:1(11Z))[iso6]	LMGL03014640
	TG(15:1(9Z)/16:0/19:1(9Z))[iso6]	LMGL03015367

MxP® Quant 500 XL kit lipid annotation	Potential isomers	Data base ID
TG 16:0_34:3	TG(16:0/17:1(9Z)/17:2(9Z,12Z))[iso6]	LMGL03010039
	TG(16:0/16:1(9Z)/18:2(9Z,12Z))[iso6]	LMGL03010053
	TG(16:0/16:0/18:3(9Z,12Z,15Z))[iso3]	LMGL03010054
	TG(16:0/16:0/18:3(6Z,9Z,12Z))[iso3]	LMGL03012981
	TG(12:0/16:0/22:3(10Z,13Z,16Z))[iso6]	LMGL03013343
	TG(14:0/16:0/20:3(8Z,11Z,14Z))[iso6]	LMGL03014236
	TG(14:1(9Z)/16:0/20:2(11Z,14Z))[iso6]	LMGL03014641
TG 16:0_34:4	TG(16:0/17:2(9Z,12Z)/17:2(9Z,12Z))[iso3]	LMGL03010046
	TG(16:0/16:1(9Z)/18:3(9Z,12Z,15Z))[iso6]	LMGL03010065
	TG(16:0/16:0/18:4(6Z,9Z,12Z,15Z))[iso3]	LMGL03012982
	TG(12:0/16:0/22:4(7Z,10Z,13Z,16Z))[iso6]	LMGL03013344
	TG(14:0/16:0/20:4(5Z,8Z,11Z,14Z))[iso6]	LMGL03014237
	TG(14:1(9Z)/16:0/20:3(8Z,11Z,14Z))[iso6]	LMGL03014642
	TG(16:0/16:1(9Z)/18:3(6Z,9Z,12Z))[iso6]	LMGL03015707
TG 16:0_35:1	TG(16:0/17:1(9Z)/18:0)[iso6]	LMGL03010049
	TG(16:0/17:0/18:1(9Z))[iso6]	LMGL03010051
	TG(16:0/16:1(9Z)/19:0)[iso6]	LMGL03010079
	TG(16:0/16:0/19:1(9Z))[iso3]	LMGL03012983
	TG(16:0/18:4(6Z,9Z,12Z,15Z)/18:4(6Z,9Z,12Z,15Z))[iso3]	LMGL03012986
	TG(13:0/16:0/22:1(11Z))[iso6]	LMGL03013806
	TG(14:1(9Z)/16:0/21:0)[iso6]	LMGL03014645
	TG(15:0/16:0/20:1(11Z))[iso6]	LMGL03015018
	TG(15:1(9Z)/16:0/20:0)[iso6]	LMGL03015368
TG 16:0_35:2	TG(16:0/17:2(9Z,12Z)/18:0)[iso6]	LMGL03010058
	TG(16:0/17:1(9Z)/18:1(9Z))[iso6]	LMGL03010061
	TG(16:0/17:0/18:2(9Z,12Z))[iso6]	LMGL03010063
	TG(13:0/16:0/22:2(13Z,16Z))[iso6]	LMGL03013807
	TG(15:0/16:0/20:2(11Z,14Z))[iso6]	LMGL03015019
	TG(15:1(9Z)/16:0/20:1(11Z))[iso6]	LMGL03015369
	TG(16:0/16:1(9Z)/19:1(9Z))[iso6]	LMGL03015709
TG 16:0_35:3	TG(16:0/17:2(9Z,12Z)/18:1(9Z))[iso6]	LMGL03010072
	TG(16:0/17:1(9Z)/18:2(9Z,12Z))[iso6]	LMGL03010075
	TG(16:0/17:0/18:3(9Z,12Z,15Z))[iso6]	LMGL03010077
	TG(13:0/16:0/22:3(10Z,13Z,16Z))[iso6]	LMGL03013808
	TG(15:0/16:0/20:3(8Z,11Z,14Z))[iso6]	LMGL03015020
	TG(15:1(9Z)/16:0/20:2(11Z,14Z))[iso6]	LMGL03015370
	TG(16:0/17:0/18:3(6Z,9Z,12Z))[iso6]	LMGL03015711
TG 16:0_36:2	TG(16:0/18:1(9Z)/18:1(9Z))[iso3]	LMGL03010100
	TG(16:0/18:0/18:2(9Z,12Z))[iso6]	LMGL03010104
	TG(16:0/16:1(9Z)/20:1(11Z))[iso6]	LMGL03010114
	TG(16:0/16:0/20:2(11Z,14Z))[iso3]	LMGL03010115
	TG(16:0/17:2(9Z,12Z)/19:0)[iso6]	LMGL03010128
	TG(14:0/16:0/22:2(13Z,16Z))[iso6]	LMGL03014242
	TG(14:1(9Z)/16:0/22:1(11Z))[iso6]	LMGL03014647
	TG(16:0/17:1(9Z)/19:1(9Z))[iso6]	LMGL03015717
TG 16:0_36:3	TG(16:0/18:1(9Z)/18:2(9Z,12Z))[iso6]	LMGL03010121
	TG(16:0/18:0/18:3(9Z,12Z,15Z))[iso6]	LMGL03010125
	TG(16:0/16:1(9Z)/20:2(11Z,14Z))[iso6]	LMGL03010135
	TG(16:0/16:0/20:3(8Z,11Z,14Z))[iso3]	LMGL03010136
	TG(14:0/16:0/22:3(10Z,13Z,16Z))[iso6]	LMGL03014243
	TG(14:1(9Z)/16:0/22:2(13Z,16Z))[iso6]	LMGL03014648
	TG(16:0/17:2(9Z,12Z)/19:1(9Z))[iso6]	LMGL03015721
	TG(16:0/18:0/18:3(6Z,9Z,12Z))[iso6]	LMGL03015723

MxP® Quant 500 XL kit lipid annotation	Potential isomers	Data base ID
TG 16:0_36:4	TG(16:0/18:2(9Z,12Z)/18:2(9Z,12Z))[iso3]	LMGL03010141
	TG(16:0/18:1(9Z)/18:3(9Z,12Z,15Z))[iso6]	LMGL03010145
	TG(16:0/16:1(9Z)/20:3(8Z,11Z,14Z))[iso6]	LMGL03010159
	TG(16:0/16:0/20:4(5Z,8Z,11Z,14Z))[iso3]	LMGL03010160
	TG(14:0/16:0/22:4(7Z,10Z,13Z,16Z))[iso6]	LMGL03014244
	TG(14:1(9Z)/16:0/22:3(10Z,13Z,16Z))[iso6]	LMGL03014649
	TG(16:0/18:0/18:4(6Z,9Z,12Z,15Z))[iso6]	LMGL03015724
	TG(16:0/18:1(9Z)/18:3(6Z,9Z,12Z))[iso6]	LMGL03015727
TG 16:0_36:5	TG(16:0/18:2(9Z,12Z)/18:3(9Z,12Z,15Z))[iso6]	LMGL03010167
	TG(16:0/16:1(9Z)/20:4(5Z,8Z,11Z,14Z))[iso6]	LMGL03010185
	TG(16:0/16:0/20:5(5Z,8Z,11Z,14Z,17Z))[iso3]	LMGL03010186
	TG(14:0/16:0/22:5(7Z,10Z,13Z,16Z,19Z))[iso6]	LMGL03014245
	TG(14:1(9Z)/16:0/22:4(7Z,10Z,13Z,16Z))[iso6]	LMGL03014650
	TG(16:0/18:1(9Z)/18:4(6Z,9Z,12Z,15Z))[iso6]	LMGL03015728
	TG(16:0/18:2(9Z,12Z)/18:3(6Z,9Z,12Z))[iso6]	LMGL03015731
TG 16:0_36:6	TG(16:0/18:3(9Z,12Z,15Z)/18:3(9Z,12Z,15Z))[iso3]	LMGL03010192
	TG(16:0/16:1(9Z)/20:5(5Z,8Z,11Z,14Z,17Z))[iso6]	LMGL03010215
	TG(16:0/18:3(6Z,9Z,12Z)/18:3(6Z,9Z,12Z))[iso3]	LMGL03012985
	TG(14:0/16:0/22:6(4Z,7Z,10Z,13Z,16Z,19Z))[iso6]	LMGL03014246
	TG(14:1(9Z)/16:0/22:5(7Z,10Z,13Z,16Z,19Z))[iso6]	LMGL03014651
	TG(16:0/18:2(9Z,12Z)/18:4(6Z,9Z,12Z,15Z))[iso6]	LMGL03015732
	TG(16:0/18:3(6Z,9Z,12Z)/18:3(9Z,12Z,15Z))[iso6]	LMGL03015735
TG 16:0_37:3	TG(16:0/17:2(9Z,12Z)/20:1(11Z))[iso6]	LMGL03010178
	TG(16:0/17:1(9Z)/20:2(11Z,14Z))[iso6]	LMGL03010181
	TG(16:0/17:0/20:3(8Z,11Z,14Z))[iso6]	LMGL03010183
	TG(16:0/18:3(9Z,12Z,15Z)/19:0)[iso6]	LMGL03010225
	TG(15:0/16:0/22:3(10Z,13Z,16Z))[iso6]	LMGL03015027
	TG(15:1(9Z)/16:0/22:2(13Z,16Z))[iso6]	LMGL03015377
	TG(16:0/18:2(9Z,12Z)/19:1(9Z))[iso6]	LMGL03015733
	TG(16:0/18:3(6Z,9Z,12Z)/19:0)[iso6]	LMGL03015737
TG 16:0_38:1	TG(16:0/18:1(9Z)/20:0)[iso6]	LMGL03010201
	TG(16:0/18:0/20:1(11Z))[iso6]	LMGL03010205
	TG(16:0/16:1(9Z)/22:0)[iso6]	LMGL03010285
	TG(16:0/16:0/22:1(13Z))[iso3]	LMGL03010286
	TG(16:0/17:1(9Z)/21:0)[iso6]	LMGL03010321
	TG(16:0/17:2(9Z,12Z)/22:6(4Z,7Z,10Z,13Z,16Z,19Z))[iso6]	LMGL03010782
	TG(16:0/16:0/22:1(11Z))[iso3]	LMGL03012984
	TG(16:0/19:0/19:1(9Z))[iso6]	LMGL03015772
TG 16:0_38:2	TG(16:0/18:2(9Z,12Z)/20:0)[iso6]	LMGL03010230
	TG(16:0/18:1(9Z)/20:1(11Z))[iso6]	LMGL03010234
	TG(16:0/18:0/20:2(11Z,14Z))[iso6]	LMGL03010238
	TG(16:0/16:1(9Z)/22:1(13Z))[iso6]	LMGL03010325
	TG(16:0/17:2(9Z,12Z)/21:0)[iso6]	LMGL03010362
	TG(16:0/16:0/22:2(13Z,16Z))[iso3]	LMGL03010370
	TG(16:0/19:1(9Z)/19:1(9Z))[iso3]	LMGL03012987
	TG(16:0/16:1(9Z)/22:1(11Z))[iso6]	LMGL03015710
TG 16:0_38:3	TG(16:0/18:3(9Z,12Z,15Z)/20:0)[iso6]	LMGL03010262
	TG(16:0/18:2(9Z,12Z)/20:1(11Z))[iso6]	LMGL03010267
	TG(16:0/18:1(9Z)/20:2(11Z,14Z))[iso6]	LMGL03010271
	TG(16:0/18:0/20:3(8Z,11Z,14Z))[iso6]	LMGL03010275
	TG(16:0/16:0/22:3(10Z,13Z,16Z))[iso3]	LMGL03010326
	TG(16:0/16:1(9Z)/22:2(13Z,16Z))[iso6]	LMGL03010417
	TG(16:0/18:3(6Z,9Z,12Z)/20:0)[iso6]	LMGL03015739

MxP® Quant 500 XL kit lipid annotation	Potential isomers	Data base ID
TG 16:0_38:4	TG(16:0/18:3(9Z,12Z,15Z)/20:1(11Z))[iso6]	LMGL03010302
	TG(16:0/18:2(9Z,12Z)/20:2(11Z,14Z))[iso6]	LMGL03010307
	TG(16:0/18:1(9Z)/20:3(8Z,11Z,14Z))[iso6]	LMGL03010311
	TG(16:0/18:0/20:4(5Z,8Z,11Z,14Z))[iso6]	LMGL03010315
	TG(16:0/16:1(9Z)/22:3(10Z,13Z,16Z))[iso6]	LMGL03010369
	TG(16:0/16:0/22:4(7Z,10Z,13Z,16Z))[iso3]	LMGL03010418
	TG(16:0/18:3(6Z,9Z,12Z)/20:1(11Z))[iso6]	LMGL03015740
	TG(16:0/18:4(6Z,9Z,12Z,15Z)/20:0)[iso6]	LMGL03015758
TG 16:0_38:5	TG(16:0/18:3(9Z,12Z,15Z)/20:2(11Z,14Z))[iso6]	LMGL03010346
	TG(16:0/18:2(9Z,12Z)/20:3(8Z,11Z,14Z))[iso6]	LMGL03010351
	TG(16:0/18:1(9Z)/20:4(5Z,8Z,11Z,14Z))[iso6]	LMGL03010355
	TG(16:0/18:0/20:5(5Z,8Z,11Z,14Z,17Z))[iso6]	LMGL03010359
	TG(16:0/16:1(9Z)/22:4(7Z,10Z,13Z,16Z))[iso6]	LMGL03010469
	TG(16:0/16:0/22:5(7Z,10Z,13Z,16Z,19Z))[iso3]	LMGL03010470
	TG(16:0/18:3(6Z,9Z,12Z)/20:2(11Z,14Z))[iso6]	LMGL03015741
	TG(16:0/18:4(6Z,9Z,12Z,15Z)/20:1(11Z))[iso6]	LMGL03015759
TG 16:0_38:6	TG(16:0/18:3(9Z,12Z,15Z)/20:3(8Z,11Z,14Z))[iso6]	LMGL03010394
	TG(16:0/18:2(9Z,12Z)/20:4(5Z,8Z,11Z,14Z))[iso6]	LMGL03010399
	TG(16:0/18:1(9Z)/20:5(5Z,8Z,11Z,14Z,17Z))[iso6]	LMGL03010403
	TG(16:0/16:1(9Z)/22:5(7Z,10Z,13Z,16Z,19Z))[iso6]	LMGL03010525
	TG(16:0/16:0/22:6(4Z,7Z,10Z,13Z,16Z,19Z))[iso3]	LMGL03010526
	TG(16:0/18:3(6Z,9Z,12Z)/20:3(8Z,11Z,14Z))[iso6]	LMGL03015742
	TG(16:0/18:4(6Z,9Z,12Z,15Z)/20:2(11Z,14Z))[iso6]	LMGL03015760
TG 16:0_38:7	TG(16:0/17:0/20:0)[iso6]	LMGL03010112
	TG(16:0/18:0/19:0)[iso6]	LMGL03010149
	TG(16:0/16:0/21:0)[iso3]	LMGL03010216
	TG(16:0/18:3(9Z,12Z,15Z)/20:4(5Z,8Z,11Z,14Z))[iso6]	LMGL03010446
	TG(16:0/18:2(9Z,12Z)/20:5(5Z,8Z,11Z,14Z,17Z))[iso6]	LMGL03010451
	TG(16:0/16:1(9Z)/22:6(4Z,7Z,10Z,13Z,16Z,19Z))[iso6]	LMGL03010586
	TG(15:0/16:0/22:0)[iso6]	LMGL03015024
	TG(16:0/18:3(6Z,9Z,12Z)/20:4(5Z,8Z,11Z,14Z))[iso6]	LMGL03015743
	TG(16:0/18:4(6Z,9Z,12Z,15Z)/20:3(8Z,11Z,14Z))[iso6]	LMGL03015761
TG 16:0_40:6	TG(16:0/20:3(8Z,11Z,14Z)/20:3(8Z,11Z,14Z))[iso3]	LMGL03010664
	TG(16:0/20:2(11Z,14Z)/20:4(5Z,8Z,11Z,14Z))[iso6]	LMGL03010671
	TG(16:0/20:1(11Z)/20:5(5Z,8Z,11Z,14Z,17Z))[iso6]	LMGL03010678
	TG(16:0/18:3(9Z,12Z,15Z)/22:3(10Z,13Z,16Z))[iso6]	LMGL03010766
	TG(16:0/18:2(9Z,12Z)/22:4(7Z,10Z,13Z,16Z))[iso6]	LMGL03010845
	TG(16:0/18:1(9Z)/22:5(7Z,10Z,13Z,16Z,19Z))[iso6]	LMGL03010849
	TG(16:0/18:0/22:6(4Z,7Z,10Z,13Z,16Z,19Z))[iso6]	LMGL03010853
	TG(16:0/18:3(6Z,9Z,12Z)/22:3(10Z,13Z,16Z))[iso6]	LMGL03015749
	TG(16:0/18:4(6Z,9Z,12Z,15Z)/22:2(13Z,16Z))[iso6]	LMGL03015767
TG 16:0_40:7	TG(16:0/19:0/20:0)[iso6]	LMGL03010297
	TG(16:0/17:0/22:0)[iso6]	LMGL03010323
	TG(16:0/18:0/21:0)[iso6]	LMGL03010407
	TG(16:0/20:3(8Z,11Z,14Z)/20:4(5Z,8Z,11Z,14Z))[iso6]	LMGL03010735
	TG(16:0/20:2(11Z,14Z)/20:5(5Z,8Z,11Z,14Z,17Z))[iso6]	LMGL03010742
	TG(16:0/18:3(9Z,12Z,15Z)/22:4(7Z,10Z,13Z,16Z))[iso6]	LMGL03010916
	TG(16:0/18:2(9Z,12Z)/22:5(7Z,10Z,13Z,16Z,19Z))[iso6]	LMGL03010921
	TG(16:0/18:1(9Z)/22:6(4Z,7Z,10Z,13Z,16Z,19Z))[iso6]	LMGL03010925
	TG(16:0/18:3(6Z,9Z,12Z)/22:4(7Z,10Z,13Z,16Z))[iso6]	LMGL03015750
	TG(16:0/18:4(6Z,9Z,12Z,15Z)/22:3(10Z,13Z,16Z))[iso6]	LMGL03015768

MxP® Quant 500 XL kit lipid annotation	Potential isomers	Data base ID
TG 16:0_40:8	TG(16:0/19:0/20:1(11Z))[iso6]	LMGL03010341
	TG(16:0/17:1(9Z)/22:0)[iso6]	LMGL03010365
	TG(16:0/17:0/22:1(13Z))[iso6]	LMGL03010367
	TG(16:0/18:1(9Z)/21:0)[iso6]	LMGL03010455
	TG(16:0/20:4(5Z,8Z,11Z,14Z)/20:4(5Z,8Z,11Z,14Z))[iso3]	LMGL03010801
	TG(16:0/20:3(8Z,11Z,14Z)/20:5(5Z,8Z,11Z,14Z,17Z))[iso6]	LMGL03010809
	TG(16:0/18:3(9Z,12Z,15Z)/22:5(7Z,10Z,13Z,16Z,19Z))[iso6]	LMGL03010994
	TG(16:0/18:2(9Z,12Z)/22:6(4Z,7Z,10Z,13Z,16Z,19Z))[iso6]	LMGL03010999
	TG(16:0/17:0/22:1(11Z))[iso6]	LMGL03015714
	TG(16:0/18:3(6Z,9Z,12Z)/22:5(7Z,10Z,13Z,16Z,19Z))[iso6]	LMGL03015751
	TG(16:0/18:4(6Z,9Z,12Z,15Z)/22:4(7Z,10Z,13Z,16Z))[iso6]	LMGL03015769
	TG(16:0/19:1(9Z)/20:0)[iso6]	LMGL03015774
TG 16:1_28:0	TG(14:0/14:0/16:1(9Z))[iso3]	LMGL03012757
	TG(12:0/16:0/16:1(9Z))[iso6]	LMGL03013322
	TG(13:0/15:0/16:1(9Z))[iso6]	LMGL03013733
TG 16:1_30:1	TG(14:0/16:1(9Z)/16:1(9Z))[iso3]	LMGL03012787
	TG(12:0/16:1(9Z)/18:1(9Z))[iso6]	LMGL03013351
	TG(13:0/16:1(9Z)/17:1(9Z))[iso6]	LMGL03013813
	TG(14:1(9Z)/16:0/16:1(9Z))[iso6]	LMGL03014627
	TG(15:0/15:1(9Z)/16:1(9Z))[iso6]	LMGL03014979
TG 16:1_32:0	TG(16:0/16:0/16:1(9Z))[iso3]	LMGL03010017
	TG(12:0/16:1(9Z)/20:0)[iso6]	LMGL03013358
	TG(13:0/16:1(9Z)/19:0)[iso6]	LMGL03013821
	TG(14:0/16:1(9Z)/18:0)[iso6]	LMGL03014250
	TG(15:0/16:1(9Z)/17:0)[iso6]	LMGL03015031
TG 16:1_32:1	TG(16:0/16:1(9Z)/16:1(9Z))[iso3]	LMGL03010018
	TG(12:0/16:1(9Z)/20:1(11Z))[iso6]	LMGL03013359
	TG(13:0/16:1(9Z)/19:1(9Z))[iso6]	LMGL03013822
	TG(14:0/16:1(9Z)/18:1(9Z))[iso6]	LMGL03014251
	TG(14:1(9Z)/16:1(9Z)/18:0)[iso6]	LMGL03014656
	TG(15:0/16:1(9Z)/17:1(9Z))[iso6]	LMGL03015032
	TG(15:1(9Z)/16:1(9Z)/17:0)[iso6]	LMGL03015382
TG 16:1_32:2	TG(16:1(9Z)/16:1(9Z)/16:1(9Z))	LMGL03010020
	TG(12:0/16:1(9Z)/20:2(11Z,14Z))[iso6]	LMGL03013360
	TG(14:0/16:1(9Z)/18:2(9Z,12Z))[iso6]	LMGL03014252
	TG(14:1(9Z)/16:1(9Z)/18:1(9Z))[iso6]	LMGL03014657
	TG(15:0/16:1(9Z)/17:2(9Z,12Z))[iso6]	LMGL03015033
	TG(15:1(9Z)/16:1(9Z)/17:1(9Z))[iso6]	LMGL03015383
TG 16:1_33:1	TG(16:1(9Z)/16:1(9Z)/17:0)[iso3]	LMGL03010024
	TG(16:0/16:1(9Z)/17:1(9Z))[iso6]	LMGL03010025
	TG(13:0/16:1(9Z)/20:1(11Z))[iso6]	LMGL03013824
	TG(14:0/16:1(9Z)/19:1(9Z))[iso6]	LMGL03014257
	TG(14:1(9Z)/16:1(9Z)/19:0)[iso6]	LMGL03014662
	TG(15:0/16:1(9Z)/18:1(9Z))[iso6]	LMGL03015035
	TG(15:1(9Z)/16:1(9Z)/18:0)[iso6]	LMGL03015385
TG 16:1_34:0	TG(16:1(9Z)/17:0/17:0)[iso3]	LMGL03010027
	TG(16:0/16:1(9Z)/18:0)[iso6]	LMGL03010036
	TG(12:0/16:1(9Z)/22:0)[iso6]	LMGL03013365
	TG(13:0/16:1(9Z)/21:0)[iso6]	LMGL03013829
	TG(14:0/16:1(9Z)/20:0)[iso6]	LMGL03014258
	TG(15:0/16:1(9Z)/19:0)[iso6]	LMGL03015040

MxP® Quant 500 XL kit lipid annotation	Potential isomers	Data base ID
TG 16:1_34:1	TG(16:1(9Z)/17:0/17:1(9Z))[iso6]	LMGL03010033
	TG(16:1(9Z)/16:1(9Z)/18:0)[iso3]	LMGL03010042
	TG(16:0/16:1(9Z)/18:1(9Z))[iso6]	LMGL03010043
	TG(12:0/16:1(9Z)/22:1(11Z))[iso6]	LMGL03013366
	TG(14:0/16:1(9Z)/20:1(11Z))[iso6]	LMGL03014259
	TG(14:1(9Z)/16:1(9Z)/20:0)[iso6]	LMGL03014664
	TG(15:0/16:1(9Z)/19:1(9Z))[iso6]	LMGL03015041
	TG(15:1(9Z)/16:1(9Z)/19:0)[iso6]	LMGL03015391
TG 16:1_34:2	TG(16:1(9Z)/17:1(9Z)/17:1(9Z))[iso3]	LMGL03010037
	TG(16:1(9Z)/17:0/17:2(9Z,12Z))[iso6]	LMGL03010040
	TG(16:1(9Z)/16:1(9Z)/18:1(9Z))[iso3]	LMGL03010052
	TG(16:0/16:1(9Z)/18:2(9Z,12Z))[iso6]	LMGL03010053
	TG(12:0/16:1(9Z)/22:2(13Z,16Z))[iso6]	LMGL03013367
	TG(14:0/16:1(9Z)/20:2(11Z,14Z))[iso6]	LMGL03014260
	TG(14:1(9Z)/16:1(9Z)/20:1(11Z))[iso6]	LMGL03014665
	TG(15:1(9Z)/16:1(9Z)/19:1(9Z))[iso6]	LMGL03015392
TG 16:1_34:3	TG(16:1(9Z)/17:1(9Z)/17:2(9Z,12Z))[iso6]	LMGL03010047
	TG(16:1(9Z)/16:1(9Z)/18:2(9Z,12Z))[iso3]	LMGL03010064
	TG(16:0/16:1(9Z)/18:3(9Z,12Z,15Z))[iso6]	LMGL03010065
	TG(12:0/16:1(9Z)/22:3(10Z,13Z,16Z))[iso6]	LMGL03013368
	TG(14:0/16:1(9Z)/20:3(8Z,11Z,14Z))[iso6]	LMGL03014261
	TG(14:1(9Z)/16:1(9Z)/20:2(11Z,14Z))[iso6]	LMGL03014666
	TG(16:0/16:1(9Z)/18:3(6Z,9Z,12Z))[iso6]	LMGL03015707
TG 16:1_36:1	TG(16:1(9Z)/18:0/18:1(9Z))[iso6]	LMGL03010101
	TG(16:1(9Z)/16:1(9Z)/20:0)[iso3]	LMGL03010113
	TG(16:0/16:1(9Z)/20:1(11Z))[iso6]	LMGL03010114
	TG(16:1(9Z)/17:1(9Z)/19:0)[iso6]	LMGL03010129
	TG(14:0/16:1(9Z)/22:1(11Z))[iso6]	LMGL03014266
	TG(14:1(9Z)/16:1(9Z)/22:0)[iso6]	LMGL03014671
	TG(15:1(9Z)/16:1(9Z)/21:0)[iso6]	LMGL03015399
	TG(16:1(9Z)/17:0/19:1(9Z))[iso6]	LMGL03015804
TG 16:1_36:2	TG(16:1(9Z)/18:1(9Z)/18:1(9Z))[iso3]	LMGL03010118
	TG(16:1(9Z)/18:0/18:2(9Z,12Z))[iso6]	LMGL03010122
	TG(16:1(9Z)/16:1(9Z)/20:1(11Z))[iso3]	LMGL03010134
	TG(16:0/16:1(9Z)/20:2(11Z,14Z))[iso6]	LMGL03010135
	TG(16:1(9Z)/17:2(9Z,12Z)/19:0)[iso6]	LMGL03010150
	TG(14:0/16:1(9Z)/22:2(13Z,16Z))[iso6]	LMGL03014267
	TG(14:1(9Z)/16:1(9Z)/22:1(11Z))[iso6]	LMGL03014672
	TG(16:1(9Z)/17:1(9Z)/19:1(9Z))[iso6]	LMGL03015808
TG 16:1_36:3	TG(16:1(9Z)/18:1(9Z)/18:2(9Z,12Z))[iso6]	LMGL03010142
	TG(16:1(9Z)/18:0/18:3(9Z,12Z,15Z))[iso6]	LMGL03010146
	TG(16:1(9Z)/16:1(9Z)/20:2(11Z,14Z))[iso3]	LMGL03010158
	TG(16:0/16:1(9Z)/20:3(8Z,11Z,14Z))[iso6]	LMGL03010159
	TG(14:0/16:1(9Z)/22:3(10Z,13Z,16Z))[iso6]	LMGL03014268
	TG(14:1(9Z)/16:1(9Z)/22:2(13Z,16Z))[iso6]	LMGL03014673
	TG(16:1(9Z)/17:2(9Z,12Z)/19:1(9Z))[iso6]	LMGL03015812
	TG(16:1(9Z)/18:0/18:3(6Z,9Z,12Z))[iso6]	LMGL03015814
TG 16:1_36:4	TG(16:1(9Z)/18:2(9Z,12Z)/18:2(9Z,12Z))[iso3]	LMGL03010163
	TG(16:1(9Z)/18:1(9Z)/18:3(9Z,12Z,15Z))[iso6]	LMGL03010168
	TG(16:1(9Z)/16:1(9Z)/20:3(8Z,11Z,14Z))[iso3]	LMGL03010184
	TG(16:0/16:1(9Z)/20:4(5Z,8Z,11Z,14Z))[iso6]	LMGL03010185
	TG(14:0/16:1(9Z)/22:4(7Z,10Z,13Z,16Z))[iso6]	LMGL03014269
	TG(14:1(9Z)/16:1(9Z)/22:3(10Z,13Z,16Z))[iso6]	LMGL03014674
	TG(16:1(9Z)/18:0/18:4(6Z,9Z,12Z,15Z))[iso6]	LMGL03015815
	TG(16:1(9Z)/18:1(9Z)/18:3(6Z,9Z,12Z))[iso6]	LMGL03015818

MxP® Quant 500 XL kit lipid annotation	Potential isomers	Data base ID
TG 16:1_36:5	TG(16:1(9Z)/18:2(9Z,12Z)/18:3(9Z,12Z,15Z))[iso6]	LMGL03010193
	TG(16:1(9Z)/16:1(9Z)/20:4(5Z,8Z,11Z,14Z))[iso3]	LMGL03010214
	TG(16:0/16:1(9Z)/20:5(5Z,8Z,11Z,14Z,17Z))[iso6]	LMGL03010215
	TG(14:0/16:1(9Z)/22:5(7Z,10Z,13Z,16Z,19Z))[iso6]	LMGL03014270
	TG(14:1(9Z)/16:1(9Z)/22:4(7Z,10Z,13Z,16Z))[iso6]	LMGL03014675
	TG(16:1(9Z)/18:1(9Z)/18:4(6Z,9Z,12Z,15Z))[iso6]	LMGL03015819
	TG(16:1(9Z)/18:2(9Z,12Z)/18:3(6Z,9Z,12Z))[iso6]	LMGL03015822
TG 16:1_38:3	TG(16:1(9Z)/18:3(9Z,12Z,15Z)/20:0)[iso6]	LMGL03010298
	TG(16:1(9Z)/18:2(9Z,12Z)/20:1(11Z))[iso6]	LMGL03010303
	TG(16:1(9Z)/18:1(9Z)/20:2(11Z,14Z))[iso6]	LMGL03010308
	TG(16:1(9Z)/18:0/20:3(8Z,11Z,14Z))[iso6]	LMGL03010312
	TG(16:0/16:1(9Z)/22:3(10Z,13Z,16Z))[iso6]	LMGL03010369
	TG(16:1(9Z)/16:1(9Z)/22:2(13Z,16Z))[iso3]	LMGL03010468
	TG(16:1(9Z)/18:3(6Z,9Z,12Z)/20:0)[iso6]	LMGL03015830
TG 16:1_38:4	TG(16:1(9Z)/18:3(9Z,12Z,15Z)/20:1(11Z))[iso6]	LMGL03010342
	TG(16:1(9Z)/18:2(9Z,12Z)/20:2(11Z,14Z))[iso6]	LMGL03010347
	TG(16:1(9Z)/18:1(9Z)/20:3(8Z,11Z,14Z))[iso6]	LMGL03010352
	TG(16:1(9Z)/18:0/20:4(5Z,8Z,11Z,14Z))[iso6]	LMGL03010356
	TG(16:1(9Z)/16:1(9Z)/22:3(10Z,13Z,16Z))[iso3]	LMGL03010416
	TG(16:0/16:1(9Z)/22:4(7Z,10Z,13Z,16Z))[iso6]	LMGL03010469
	TG(16:1(9Z)/18:3(6Z,9Z,12Z)/20:1(11Z))[iso6]	LMGL03015831
	TG(16:1(9Z)/18:4(6Z,9Z,12Z,15Z)/20:0)[iso6]	LMGL03015849
TG 16:1_38:5	TG(16:1(9Z)/18:3(9Z,12Z,15Z)/20:2(11Z,14Z))[iso6]	LMGL03010390
	TG(16:1(9Z)/18:2(9Z,12Z)/20:3(8Z,11Z,14Z))[iso6]	LMGL03010395
	TG(16:1(9Z)/18:1(9Z)/20:4(5Z,8Z,11Z,14Z))[iso6]	LMGL03010400
	TG(16:1(9Z)/18:0/20:5(5Z,8Z,11Z,14Z,17Z))[iso6]	LMGL03010404
	TG(16:1(9Z)/16:1(9Z)/22:4(7Z,10Z,13Z,16Z))[iso3]	LMGL03010524
	TG(16:0/16:1(9Z)/22:5(7Z,10Z,13Z,16Z,19Z))[iso6]	LMGL03010525
	TG(16:1(9Z)/18:3(6Z,9Z,12Z)/20:2(11Z,14Z))[iso6]	LMGL03015832
	TG(16:1(9Z)/18:4(6Z,9Z,12Z,15Z)/20:1(11Z))[iso6]	LMGL03015850
TG 17:0_32:1	TG(16:0/16:1(9Z)/17:0)[iso6]	LMGL03010021
	TG(15:1(9Z)/17:0/17:0)[iso3]	LMGL03012956
	TG(12:0/17:0/20:1(11Z))[iso6]	LMGL03013383
	TG(14:0/17:0/18:1(9Z))[iso6]	LMGL03014275
	TG(14:1(9Z)/17:0/18:0)[iso6]	LMGL03014680
	TG(15:0/17:0/17:1(9Z))[iso6]	LMGL03015056
TG 17:0_34:1	TG(17:0/17:0/17:1(9Z))[iso3]	LMGL03010038
	TG(16:1(9Z)/17:0/18:0)[iso6]	LMGL03010050
	TG(16:0/17:0/18:1(9Z))[iso6]	LMGL03010051
	TG(12:0/17:0/22:1(11Z))[iso6]	LMGL03013390
	TG(14:0/17:0/20:1(11Z))[iso6]	LMGL03014283
	TG(14:1(9Z)/17:0/20:0)[iso6]	LMGL03014688
	TG(15:0/17:0/19:1(9Z))[iso6]	LMGL03015065
	TG(15:1(9Z)/17:0/19:0)[iso6]	LMGL03015415
TG 17:0_34:2	TG(17:0/17:1(9Z)/17:1(9Z))[iso3]	LMGL03010045
	TG(17:0/17:0/17:2(9Z,12Z))[iso3]	LMGL03010048
	TG(16:1(9Z)/17:0/18:1(9Z))[iso6]	LMGL03010062
	TG(16:0/17:0/18:2(9Z,12Z))[iso6]	LMGL03010063
	TG(12:0/17:0/22:2(13Z,16Z))[iso6]	LMGL03013391
	TG(14:0/17:0/20:2(11Z,14Z))[iso6]	LMGL03014284
	TG(14:1(9Z)/17:0/20:1(11Z))[iso6]	LMGL03014689
	TG(15:1(9Z)/17:0/19:1(9Z))[iso6]	LMGL03015416

MxP® Quant 500 XL kit lipid annotation	Potential isomers	Data base ID
TG 17:0_34:3	TG(17:0/17:1(9Z)/17:2(9Z,12Z))[iso6]	LMGL03010057
	TG(16:1(9Z)/17:0/18:2(9Z,12Z))[iso6]	LMGL03010076
	TG(16:0/17:0/18:3(9Z,12Z,15Z))[iso6]	LMGL03010077
	TG(12:0/17:0/22:3(10Z,13Z,16Z))[iso6]	LMGL03013392
	TG(14:0/17:0/20:3(8Z,11Z,14Z))[iso6]	LMGL03014285
	TG(14:1(9Z)/17:0/20:2(11Z,14Z))[iso6]	LMGL03014690
	TG(16:0/17:0/18:3(6Z,9Z,12Z))[iso6]	LMGL03015711
TG 17:0_36:3	TG(17:0/18:1(9Z)/18:2(9Z,12Z))[iso6]	LMGL03010164
	TG(17:0/18:0/18:3(9Z,12Z,15Z))[iso6]	LMGL03010169
	TG(16:1(9Z)/17:0/20:2(11Z,14Z))[iso6]	LMGL03010182
	TG(16:0/17:0/20:3(8Z,11Z,14Z))[iso6]	LMGL03010183
	TG(14:0/17:0/22:3(10Z,13Z,16Z))[iso6]	LMGL03014292
	TG(14:1(9Z)/17:0/22:2(13Z,16Z))[iso6]	LMGL03014697
	TG(17:0/17:2(9Z,12Z)/19:1(9Z))[iso6]	LMGL03015899
	TG(17:0/18:0/18:3(6Z,9Z,12Z))[iso6]	LMGL03015901
TG 17:0_36:4	TG(17:0/18:2(9Z,12Z)/18:2(9Z,12Z))[iso3]	LMGL03010189
	TG(17:0/18:1(9Z)/18:3(9Z,12Z,15Z))[iso6]	LMGL03010194
	TG(16:1(9Z)/17:0/20:3(8Z,11Z,14Z))[iso6]	LMGL03010212
	TG(16:0/17:0/20:4(5Z,8Z,11Z,14Z))[iso6]	LMGL03010213
	TG(14:0/17:0/22:4(7Z,10Z,13Z,16Z))[iso6]	LMGL03014293
	TG(14:1(9Z)/17:0/22:3(10Z,13Z,16Z))[iso6]	LMGL03014698
	TG(17:0/18:0/18:4(6Z,9Z,12Z,15Z))[iso6]	LMGL03015902
	TG(17:0/18:1(9Z)/18:3(6Z,9Z,12Z))[iso6]	LMGL03015905
TG 17:1_32:1	TG(16:0/16:1(9Z)/17:1(9Z))[iso6]	LMGL03010025
	TG(15:0/17:1(9Z)/17:1(9Z))[iso3]	LMGL03012903
	TG(12:0/17:1(9Z)/20:1(11Z))[iso6]	LMGL03013406
	TG(13:0/17:1(9Z)/19:1(9Z))[iso6]	LMGL03013869
	TG(14:0/17:1(9Z)/18:1(9Z))[iso6]	LMGL03014298
	TG(14:1(9Z)/17:1(9Z)/18:0)[iso6]	LMGL03014703
	TG(15:1(9Z)/17:0/17:1(9Z))[iso6]	LMGL03015407
TG 17:1_34:1	TG(17:0/17:1(9Z)/17:1(9Z))[iso3]	LMGL03010045
	TG(16:1(9Z)/17:1(9Z)/18:0)[iso6]	LMGL03010059
	TG(16:0/17:1(9Z)/18:1(9Z))[iso6]	LMGL03010061
	TG(12:0/17:1(9Z)/22:1(11Z))[iso6]	LMGL03013413
	TG(14:0/17:1(9Z)/20:1(11Z))[iso6]	LMGL03014306
	TG(14:1(9Z)/17:1(9Z)/20:0)[iso6]	LMGL03014711
	TG(15:0/17:1(9Z)/19:1(9Z))[iso6]	LMGL03015088
	TG(15:1(9Z)/17:1(9Z)/19:0)[iso6]	LMGL03015438
TG 17:1_34:2	TG(17:1(9Z)/17:1(9Z)/17:1(9Z))	LMGL03010055
	TG(17:0/17:1(9Z)/17:2(9Z,12Z))[iso6]	LMGL03010057
	TG(16:1(9Z)/17:1(9Z)/18:1(9Z))[iso6]	LMGL03010073
	TG(16:0/17:1(9Z)/18:2(9Z,12Z))[iso6]	LMGL03010075
	TG(12:0/17:1(9Z)/22:2(13Z,16Z))[iso6]	LMGL03013414
	TG(14:0/17:1(9Z)/20:2(11Z,14Z))[iso6]	LMGL03014307
	TG(14:1(9Z)/17:1(9Z)/20:1(11Z))[iso6]	LMGL03014712
	TG(15:1(9Z)/17:1(9Z)/19:1(9Z))[iso6]	LMGL03015439
TG 17:1_34:3	TG(17:1(9Z)/17:1(9Z)/17:2(9Z,12Z))[iso3]	LMGL03010068
	TG(16:1(9Z)/17:1(9Z)/18:2(9Z,12Z))[iso6]	LMGL03010089
	TG(16:0/17:1(9Z)/18:3(9Z,12Z,15Z))[iso6]	LMGL03010091
	TG(12:0/17:1(9Z)/22:3(10Z,13Z,16Z))[iso6]	LMGL03013415
	TG(14:0/17:1(9Z)/20:3(8Z,11Z,14Z))[iso6]	LMGL03014308
	TG(14:1(9Z)/17:1(9Z)/20:2(11Z,14Z))[iso6]	LMGL03014713
	TG(16:0/17:1(9Z)/18:3(6Z,9Z,12Z))[iso6]	LMGL03015715

MxP® Quant 500 XL kit lipid annotation	Potential isomers	Data base ID
TG 17:1_36:3	TG(17:1(9Z)/18:1(9Z)/18:2(9Z,12Z))[iso6]	LMGL03010190
	TG(17:1(9Z)/18:0/18:3(9Z,12Z,15Z))[iso6]	LMGL03010195
	TG(16:1(9Z)/17:1(9Z)/20:2(11Z,14Z))[iso6]	LMGL03010209
	TG(16:0/17:1(9Z)/20:3(8Z,11Z,14Z))[iso6]	LMGL03010211
	TG(14:0/17:1(9Z)/22:3(10Z,13Z,16Z))[iso6]	LMGL03014315
	TG(14:1(9Z)/17:1(9Z)/22:2(13Z,16Z))[iso6]	LMGL03014720
	TG(17:1(9Z)/17:2(9Z,12Z)/19:1(9Z))[iso6]	LMGL03015982
	TG(17:1(9Z)/18:0/18:3(6Z,9Z,12Z))[iso6]	LMGL03015984
TG 17:1_36:4	TG(17:1(9Z)/18:2(9Z,12Z)/18:2(9Z,12Z))[iso3]	LMGL03010218
	TG(17:1(9Z)/18:1(9Z)/18:3(9Z,12Z,15Z))[iso6]	LMGL03010223
	TG(16:1(9Z)/17:1(9Z)/20:3(8Z,11Z,14Z))[iso6]	LMGL03010242
	TG(16:0/17:1(9Z)/20:4(5Z,8Z,11Z,14Z))[iso6]	LMGL03010244
	TG(14:0/17:1(9Z)/22:4(7Z,10Z,13Z,16Z))[iso6]	LMGL03014316
	TG(14:1(9Z)/17:1(9Z)/22:3(10Z,13Z,16Z))[iso6]	LMGL03014721
	TG(17:1(9Z)/18:0/18:4(6Z,9Z,12Z,15Z))[iso6]	LMGL03015985
	TG(17:1(9Z)/18:1(9Z)/18:3(6Z,9Z,12Z))[iso6]	LMGL03015988
TG 17:1_36:5	TG(17:1(9Z)/18:2(9Z,12Z)/18:3(9Z,12Z,15Z))[iso6]	LMGL03010254
	TG(16:1(9Z)/17:1(9Z)/20:4(5Z,8Z,11Z,14Z))[iso6]	LMGL03010279
	TG(16:0/17:1(9Z)/20:5(5Z,8Z,11Z,14Z,17Z))[iso6]	LMGL03010281
	TG(14:0/17:1(9Z)/22:5(7Z,10Z,13Z,16Z,19Z))[iso6]	LMGL03014317
	TG(14:1(9Z)/17:1(9Z)/22:4(7Z,10Z,13Z,16Z))[iso6]	LMGL03014722
	TG(17:1(9Z)/18:1(9Z)/18:4(6Z,9Z,12Z,15Z))[iso6]	LMGL03015989
	TG(17:1(9Z)/18:2(9Z,12Z)/18:3(6Z,9Z,12Z))[iso6]	LMGL03015992
TG 17:1_38:5	TG(17:1(9Z)/18:3(9Z,12Z,15Z)/20:2(11Z,14Z))[iso6]	LMGL03010488
	TG(17:1(9Z)/18:2(9Z,12Z)/20:3(8Z,11Z,14Z))[iso6]	LMGL03010494
	TG(17:1(9Z)/18:1(9Z)/20:4(5Z,8Z,11Z,14Z))[iso6]	LMGL03010500
	TG(17:1(9Z)/18:0/20:5(5Z,8Z,11Z,14Z,17Z))[iso6]	LMGL03010505
	TG(16:1(9Z)/17:1(9Z)/22:4(7Z,10Z,13Z,16Z))[iso6]	LMGL03010644
	TG(16:0/17:1(9Z)/22:5(7Z,10Z,13Z,16Z,19Z))[iso6]	LMGL03010646
	TG(17:1(9Z)/18:3(6Z,9Z,12Z)/20:2(11Z,14Z))[iso6]	LMGL03016002
	TG(17:1(9Z)/18:4(6Z,9Z,12Z,15Z)/20:1(11Z))[iso6]	LMGL03016020
TG 17:1_38:6	TG(17:1(9Z)/18:3(9Z,12Z,15Z)/20:3(8Z,11Z,14Z))[iso6]	LMGL03010549
	TG(17:1(9Z)/18:2(9Z,12Z)/20:4(5Z,8Z,11Z,14Z))[iso6]	LMGL03010555
	TG(17:1(9Z)/18:1(9Z)/20:5(5Z,8Z,11Z,14Z,17Z))[iso6]	LMGL03010561
	TG(16:1(9Z)/17:1(9Z)/22:5(7Z,10Z,13Z,16Z,19Z))[iso6]	LMGL03010712
	TG(16:0/17:1(9Z)/22:6(4Z,7Z,10Z,13Z,16Z,19Z))[iso6]	LMGL03010714
	TG(17:1(9Z)/18:3(6Z,9Z,12Z)/20:3(8Z,11Z,14Z))[iso6]	LMGL03016003
	TG(17:1(9Z)/18:4(6Z,9Z,12Z,15Z)/20:2(11Z,14Z))[iso6]	LMGL03016021
TG 17:1_38:7	TG(17:0/17:1(9Z)/20:0)[iso6]	LMGL03010177
	TG(17:1(9Z)/18:0/19:0)[iso6]	LMGL03010228
	TG(16:0/17:1(9Z)/21:0)[iso6]	LMGL03010321
	TG(17:1(9Z)/18:3(9Z,12Z,15Z)/20:4(5Z,8Z,11Z,14Z))[iso6]	LMGL03010613
	TG(17:1(9Z)/18:2(9Z,12Z)/20:5(5Z,8Z,11Z,14Z,17Z))[iso6]	LMGL03010619
	TG(16:1(9Z)/17:1(9Z)/22:6(4Z,7Z,10Z,13Z,16Z,19Z))[iso6]	LMGL03010783
	TG(15:0/17:1(9Z)/22:0)[iso6]	LMGL03015096
	TG(17:1(9Z)/18:3(6Z,9Z,12Z)/20:4(5Z,8Z,11Z,14Z))[iso6]	LMGL03016004
	TG(17:1(9Z)/18:4(6Z,9Z,12Z,15Z)/20:3(8Z,11Z,14Z))[iso6]	LMGL03016022
TG 17:2_34:2	TG(17:0/17:2(9Z,12Z)/17:2(9Z,12Z))[iso3]	LMGL03010067
	TG(17:1(9Z)/17:1(9Z)/17:2(9Z,12Z))[iso3]	LMGL03010068
	TG(16:1(9Z)/17:2(9Z,12Z)/18:1(9Z))[iso6]	LMGL03010086
	TG(16:0/17:2(9Z,12Z)/18:2(9Z,12Z))[iso6]	LMGL03010088
	TG(12:0/17:2(9Z,12Z)/22:2(13Z,16Z))[iso6]	LMGL03013436
	TG(14:0/17:2(9Z,12Z)/20:2(11Z,14Z))[iso6]	LMGL03014329
	TG(14:1(9Z)/17:2(9Z,12Z)/20:1(11Z))[iso6]	LMGL03014734
	TG(15:1(9Z)/17:2(9Z,12Z)/19:1(9Z))[iso6]	LMGL03015461

MxP® Quant 500 XL kit lipid annotation	Potential isomers	Data base ID
TG 17:2_34:3	TG(17:1(9Z)/17:2(9Z,12Z)/17:2(9Z,12Z))[iso3]	LMGL03010081
	TG(16:1(9Z)/17:2(9Z,12Z)/18:2(9Z,12Z))[iso6]	LMGL03010105
	TG(16:0/17:2(9Z,12Z)/18:3(9Z,12Z,15Z))[iso6]	LMGL03010107
	TG(12:0/17:2(9Z,12Z)/22:3(10Z,13Z,16Z))[iso6]	LMGL03013437
	TG(14:0/17:2(9Z,12Z)/20:3(8Z,11Z,14Z))[iso6]	LMGL03014330
	TG(14:1(9Z)/17:2(9Z,12Z)/20:2(11Z,14Z))[iso6]	LMGL03014735
	TG(16:0/17:2(9Z,12Z)/18:3(6Z,9Z,12Z))[iso6]	LMGL03015719
TG 17:2_36:2	TG(17:2(9Z,12Z)/18:1(9Z)/18:1(9Z))[iso3]	LMGL03010187
	TG(17:2(9Z,12Z)/18:0/18:2(9Z,12Z))[iso6]	LMGL03010191
	TG(16:1(9Z)/17:2(9Z,12Z)/20:1(11Z))[iso6]	LMGL03010206
	TG(16:0/17:2(9Z,12Z)/20:2(11Z,14Z))[iso6]	LMGL03010208
	TG(17:2(9Z,12Z)/17:2(9Z,12Z)/19:0)[iso3]	LMGL03010229
	TG(14:0/17:2(9Z,12Z)/22:2(13Z,16Z))[iso6]	LMGL03014336
	TG(14:1(9Z)/17:2(9Z,12Z)/22:1(11Z))[iso6]	LMGL03014741
	TG(17:1(9Z)/17:2(9Z,12Z)/19:1(9Z))[iso6]	LMGL03015982
TG 17:2_36:3	TG(17:2(9Z,12Z)/18:1(9Z)/18:2(9Z,12Z))[iso6]	LMGL03010219
	TG(17:2(9Z,12Z)/18:0/18:3(9Z,12Z,15Z))[iso6]	LMGL03010224
	TG(16:1(9Z)/17:2(9Z,12Z)/20:2(11Z,14Z))[iso6]	LMGL03010239
	TG(16:0/17:2(9Z,12Z)/20:3(8Z,11Z,14Z))[iso6]	LMGL03010241
	TG(17:2(9Z,12Z)/17:2(9Z,12Z)/19:1(9Z))[iso3]	LMGL03013015
	TG(14:0/17:2(9Z,12Z)/22:3(10Z,13Z,16Z))[iso6]	LMGL03014337
	TG(14:1(9Z)/17:2(9Z,12Z)/22:2(13Z,16Z))[iso6]	LMGL03014742
	TG(17:2(9Z,12Z)/18:0/18:3(6Z,9Z,12Z))[iso6]	LMGL03016063
TG 17:2_36:4	TG(17:2(9Z,12Z)/18:2(9Z,12Z)/18:2(9Z,12Z))[iso3]	LMGL03010251
	TG(17:2(9Z,12Z)/18:1(9Z)/18:3(9Z,12Z,15Z))[iso6]	LMGL03010255
	TG(16:1(9Z)/17:2(9Z,12Z)/20:3(8Z,11Z,14Z))[iso6]	LMGL03010276
	TG(16:0/17:2(9Z,12Z)/20:4(5Z,8Z,11Z,14Z))[iso6]	LMGL03010278
	TG(14:0/17:2(9Z,12Z)/22:4(7Z,10Z,13Z,16Z))[iso6]	LMGL03014338
	TG(14:1(9Z)/17:2(9Z,12Z)/22:3(10Z,13Z,16Z))[iso6]	LMGL03014743
	TG(17:2(9Z,12Z)/18:0/18:4(6Z,9Z,12Z,15Z))[iso6]	LMGL03016064
	TG(17:2(9Z,12Z)/18:1(9Z)/18:3(6Z,9Z,12Z))[iso6]	LMGL03016067
TG 17:2_38:5	TG(17:2(9Z,12Z)/18:3(9Z,12Z,15Z)/20:2(11Z,14Z))[iso6]	LMGL03010543
	TG(17:2(9Z,12Z)/18:2(9Z,12Z)/20:3(8Z,11Z,14Z))[iso6]	LMGL03010550
	TG(17:2(9Z,12Z)/18:1(9Z)/20:4(5Z,8Z,11Z,14Z))[iso6]	LMGL03010556
	TG(17:2(9Z,12Z)/18:0/20:5(5Z,8Z,11Z,14Z,17Z))[iso6]	LMGL03010562
	TG(16:1(9Z)/17:2(9Z,12Z)/22:4(7Z,10Z,13Z,16Z))[iso6]	LMGL03010709
	TG(16:0/17:2(9Z,12Z)/22:5(7Z,10Z,13Z,16Z,19Z))[iso6]	LMGL03010711
	TG(17:2(9Z,12Z)/18:3(6Z,9Z,12Z)/20:2(11Z,14Z))[iso6]	LMGL03016081
	TG(17:2(9Z,12Z)/18:4(6Z,9Z,12Z,15Z)/20:1(11Z))[iso6]	LMGL03016099
TG 17:2_38:6	TG(17:2(9Z,12Z)/18:3(9Z,12Z,15Z)/20:3(8Z,11Z,14Z))[iso6]	LMGL03010607
	TG(17:2(9Z,12Z)/18:2(9Z,12Z)/20:4(5Z,8Z,11Z,14Z))[iso6]	LMGL03010614
	TG(17:2(9Z,12Z)/18:1(9Z)/20:5(5Z,8Z,11Z,14Z,17Z))[iso6]	LMGL03010620
	TG(16:1(9Z)/17:2(9Z,12Z)/22:5(7Z,10Z,13Z,16Z,19Z))[iso6]	LMGL03010780
	TG(16:0/17:2(9Z,12Z)/22:6(4Z,7Z,10Z,13Z,16Z,19Z))[iso6]	LMGL03010782
	TG(17:2(9Z,12Z)/18:3(6Z,9Z,12Z)/20:3(8Z,11Z,14Z))[iso6]	LMGL03016082
	TG(17:2(9Z,12Z)/18:4(6Z,9Z,12Z,15Z)/20:2(11Z,14Z))[iso6]	LMGL03016100
TG 17:2_38:7	TG(17:0/17:2(9Z,12Z)/20:0)[iso6]	LMGL03010203
	TG(17:2(9Z,12Z)/18:0/19:0)[iso6]	LMGL03010261
	TG(16:0/17:2(9Z,12Z)/21:0)[iso6]	LMGL03010362
	TG(17:2(9Z,12Z)/18:3(9Z,12Z,15Z)/20:4(5Z,8Z,11Z,14Z))[iso6]	LMGL03010675
	TG(17:2(9Z,12Z)/18:2(9Z,12Z)/20:5(5Z,8Z,11Z,14Z,17Z))[iso6]	LMGL03010682
	TG(16:1(9Z)/17:2(9Z,12Z)/22:6(4Z,7Z,10Z,13Z,16Z,19Z))[iso6]	LMGL03010854
	TG(15:0/17:2(9Z,12Z)/22:0)[iso6]	LMGL03015118
	TG(17:2(9Z,12Z)/18:3(6Z,9Z,12Z)/20:4(5Z,8Z,11Z,14Z))[iso6]	LMGL03016083
	TG(17:2(9Z,12Z)/18:4(6Z,9Z,12Z,15Z)/20:3(8Z,11Z,14Z))[iso6]	LMGL03016101

MxP® Quant 500 XL kit lipid annotation	Potential isomers	Data base ID
TG 18:0_30:0	TG(12:0/18:0/18:0)[iso3]	LMGL03012669
	TG(15:0/15:0/18:0)[iso3]	LMGL03012877
	TG(13:0/17:0/18:0)[iso6]	LMGL03013839
	TG(14:0/16:0/18:0)[iso6]	LMGL03014225
TG 18:0_30:1	TG(12:0/18:0/18:1(9Z))[iso6]	LMGL03013441
	TG(13:0/17:1(9Z)/18:0)[iso6]	LMGL03013862
	TG(14:0/16:1(9Z)/18:0)[iso6]	LMGL03014250
	TG(14:1(9Z)/16:0/18:0)[iso6]	LMGL03014631
	TG(15:0/15:1(9Z)/18:0)[iso6]	LMGL03014983
TG 18:0_32:0	TG(16:0/16:1(9Z)/18:0)[iso6]	LMGL03010036
	TG(14:1(9Z)/18:0/18:0)[iso3]	LMGL03012849
	TG(12:0/18:0/20:1(11Z))[iso6]	LMGL03013449
	TG(13:0/18:0/19:1(9Z))[iso6]	LMGL03013912
	TG(14:0/18:0/18:1(9Z))[iso6]	LMGL03014341
	TG(15:0/17:1(9Z)/18:0)[iso6]	LMGL03015081
	TG(15:1(9Z)/17:0/18:0)[iso6]	LMGL03015409
TG 18:0_32:1	TG(16:0/16:1(9Z)/18:0)[iso6]	LMGL03010036
	TG(14:1(9Z)/18:0/18:0)[iso3]	LMGL03012849
	TG(12:0/18:0/20:1(11Z))[iso6]	LMGL03013449
	TG(13:0/18:0/19:1(9Z))[iso6]	LMGL03013912
	TG(14:0/18:0/18:1(9Z))[iso6]	LMGL03014341
	TG(15:0/17:1(9Z)/18:0)[iso6]	LMGL03015081
	TG(15:1(9Z)/17:0/18:0)[iso6]	LMGL03015409
TG 18:0_32:2	TG(16:1(9Z)/16:1(9Z)/18:0)[iso3]	LMGL03010042
	TG(12:0/18:0/20:2(11Z,14Z))[iso6]	LMGL03013450
	TG(14:0/18:0/18:2(9Z,12Z))[iso6]	LMGL03014342
	TG(14:1(9Z)/18:0/18:1(9Z))[iso6]	LMGL03014747
	TG(15:0/17:2(9Z,12Z)/18:0)[iso6]	LMGL03015103
	TG(15:1(9Z)/17:1(9Z)/18:0)[iso6]	LMGL03015432
TG 18:0_34:2	TG(17:0/17:2(9Z,12Z)/18:0)[iso6]	LMGL03010083
	TG(17:1(9Z)/17:1(9Z)/18:0)[iso3]	LMGL03010084
	TG(16:1(9Z)/18:0/18:1(9Z))[iso6]	LMGL03010101
	TG(16:0/18:0/18:2(9Z,12Z))[iso6]	LMGL03010104
	TG(12:0/18:0/22:2(13Z,16Z))[iso6]	LMGL03013457
	TG(14:0/18:0/20:2(11Z,14Z))[iso6]	LMGL03014350
	TG(14:1(9Z)/18:0/20:1(11Z))[iso6]	LMGL03014755
	TG(15:1(9Z)/18:0/19:1(9Z))[iso6]	LMGL03015482
TG 18:0_34:3	TG(17:1(9Z)/17:2(9Z,12Z)/18:0)[iso6]	LMGL03010099
	TG(16:1(9Z)/18:0/18:2(9Z,12Z))[iso6]	LMGL03010122
	TG(16:0/18:0/18:3(9Z,12Z,15Z))[iso6]	LMGL03010125
	TG(12:0/18:0/22:3(10Z,13Z,16Z))[iso6]	LMGL03013458
	TG(14:0/18:0/20:3(8Z,11Z,14Z))[iso6]	LMGL03014351
	TG(14:1(9Z)/18:0/20:2(11Z,14Z))[iso6]	LMGL03014756
	TG(16:0/18:0/18:3(6Z,9Z,12Z))[iso6]	LMGL03015723
TG 18:0_36:1	TG(18:0/18:0/18:1(9Z))[iso3]	LMGL03010188
	TG(16:1(9Z)/18:0/20:0)[iso6]	LMGL03010202
	TG(16:0/18:0/20:1(11Z))[iso6]	LMGL03010205
	TG(17:1(9Z)/18:0/19:0)[iso6]	LMGL03010228
	TG(14:0/18:0/22:1(11Z))[iso6]	LMGL03014356
	TG(14:1(9Z)/18:0/22:0)[iso6]	LMGL03014761
	TG(15:1(9Z)/18:0/21:0)[iso6]	LMGL03015489
	TG(17:0/18:0/19:1(9Z))[iso6]	LMGL03015903

MxP® Quant 500 XL kit lipid annotation	Potential isomers	Data base ID
TG 18:0_36:2	TG(18:0/18:1(9Z)/18:1(9Z))[iso3]	LMGL03010217
	TG(18:0/18:0/18:2(9Z,12Z))[iso3]	LMGL03010220
	TG(16:1(9Z)/18:0/20:1(11Z))[iso6]	LMGL03010235
	TG(16:0/18:0/20:2(11Z,14Z))[iso6]	LMGL03010238
	TG(17:2(9Z,12Z)/18:0/19:0)[iso6]	LMGL03010261
	TG(14:0/18:0/22:2(13Z,16Z))[iso6]	LMGL03014357
	TG(14:1(9Z)/18:0/22:1(11Z))[iso6]	LMGL03014762
	TG(17:1(9Z)/18:0/19:1(9Z))[iso6]	LMGL03015986
TG 18:0_36:3	TG(18:0/18:1(9Z)/18:2(9Z,12Z))[iso6]	LMGL03010252
	TG(18:0/18:0/18:3(9Z,12Z,15Z))[iso3]	LMGL03010256
	TG(16:1(9Z)/18:0/20:2(11Z,14Z))[iso6]	LMGL03010272
	TG(16:0/18:0/20:3(8Z,11Z,14Z))[iso6]	LMGL03010275
	TG(18:0/18:0/18:3(6Z,9Z,12Z))[iso3]	LMGL03013021
	TG(14:0/18:0/22:3(10Z,13Z,16Z))[iso6]	LMGL03014358
	TG(14:1(9Z)/18:0/22:2(13Z,16Z))[iso6]	LMGL03014763
	TG(17:2(9Z,12Z)/18:0/19:1(9Z))[iso6]	LMGL03016065
TG 18:0_36:4	TG(18:0/18:2(9Z,12Z)/18:2(9Z,12Z))[iso3]	LMGL03010287
	TG(18:0/18:1(9Z)/18:3(9Z,12Z,15Z))[iso6]	LMGL03010291
	TG(16:1(9Z)/18:0/20:3(8Z,11Z,14Z))[iso6]	LMGL03010312
	TG(16:0/18:0/20:4(5Z,8Z,11Z,14Z))[iso6]	LMGL03010315
	TG(18:0/18:0/18:4(6Z,9Z,12Z,15Z))[iso3]	LMGL03013022
	TG(14:0/18:0/22:4(7Z,10Z,13Z,16Z))[iso6]	LMGL03014359
	TG(14:1(9Z)/18:0/22:3(10Z,13Z,16Z))[iso6]	LMGL03014764
	TG(18:0/18:1(9Z)/18:3(6Z,9Z,12Z))[iso6]	LMGL03016142
TG 18:0_36:5	TG(18:0/18:2(9Z,12Z)/18:3(9Z,12Z,15Z))[iso6]	LMGL03010329
	TG(16:1(9Z)/18:0/20:4(5Z,8Z,11Z,14Z))[iso6]	LMGL03010356
	TG(16:0/18:0/20:5(5Z,8Z,11Z,14Z,17Z))[iso6]	LMGL03010359
	TG(14:0/18:0/22:5(7Z,10Z,13Z,16Z,19Z))[iso6]	LMGL03014360
	TG(14:1(9Z)/18:0/22:4(7Z,10Z,13Z,16Z))[iso6]	LMGL03014765
	TG(18:0/18:1(9Z)/18:4(6Z,9Z,12Z,15Z))[iso6]	LMGL03016143
	TG(18:0/18:2(9Z,12Z)/18:3(6Z,9Z,12Z))[iso6]	LMGL03016146
TG 18:0_38:6	TG(18:0/18:3(9Z,12Z,15Z)/20:3(8Z,11Z,14Z))[iso6]	LMGL03010669
	TG(18:0/18:2(9Z,12Z)/20:4(5Z,8Z,11Z,14Z))[iso6]	LMGL03010676
	TG(18:0/18:1(9Z)/20:5(5Z,8Z,11Z,14Z,17Z))[iso6]	LMGL03010683
	TG(16:1(9Z)/18:0/22:5(7Z,10Z,13Z,16Z,19Z))[iso6]	LMGL03010850
	TG(16:0/18:0/22:6(4Z,7Z,10Z,13Z,16Z,19Z))[iso6]	LMGL03010853
	TG(18:0/18:3(6Z,9Z,12Z)/20:3(8Z,11Z,14Z))[iso6]	LMGL03016157
	TG(18:0/18:4(6Z,9Z,12Z,15Z)/20:2(11Z,14Z))[iso6]	LMGL03016175
TG 18:0_38:7	TG(17:0/18:0/20:0)[iso6]	LMGL03010232
	TG(18:0/18:0/19:0)[iso3]	LMGL03010296
	TG(16:0/18:0/21:0)[iso6]	LMGL03010407
	TG(18:0/18:3(9Z,12Z,15Z)/20:4(5Z,8Z,11Z,14Z))[iso6]	LMGL03010740
	TG(18:0/18:2(9Z,12Z)/20:5(5Z,8Z,11Z,14Z,17Z))[iso6]	LMGL03010747
	TG(16:1(9Z)/18:0/22:6(4Z,7Z,10Z,13Z,16Z,19Z))[iso6]	LMGL03010926
	TG(15:0/18:0/22:0)[iso6]	LMGL03015139
	TG(18:0/18:3(6Z,9Z,12Z)/20:4(5Z,8Z,11Z,14Z))[iso6]	LMGL03016158
	TG(18:0/18:4(6Z,9Z,12Z,15Z)/20:3(8Z,11Z,14Z))[iso6]	LMGL03016176
TG 18:1_26:0	TG(13:0/13:0/18:1(9Z))[iso3]	LMGL03012701
	TG(12:0/14:0/18:1(9Z))[iso6]	LMGL03013217
TG 18:1_28:1	TG(12:0/16:1(9Z)/18:1(9Z))[iso6]	LMGL03013351
	TG(13:0/15:1(9Z)/18:1(9Z))[iso6]	LMGL03013765
	TG(14:0/14:1(9Z)/18:1(9Z))[iso6]	LMGL03014145
TG 18:1_30:0	TG(15:0/15:0/18:1(9Z))[iso3]	LMGL03012878
	TG(12:0/18:0/18:1(9Z))[iso6]	LMGL03013441
	TG(13:0/17:0/18:1(9Z))[iso6]	LMGL03013840
	TG(14:0/16:0/18:1(9Z))[iso6]	LMGL03014226

MxP® Quant 500 XL kit lipid annotation	Potential isomers	Data base ID
TG 18:1_30:1	TG(12:0/18:1(9Z)/18:1(9Z))[iso3]	LMGL03012670
	TG(13:0/17:1(9Z)/18:1(9Z))[iso6]	LMGL03013863
	TG(14:0/16:1(9Z)/18:1(9Z))[iso6]	LMGL03014251
	TG(14:1(9Z)/16:0/18:1(9Z))[iso6]	LMGL03014632
	TG(15:0/15:1(9Z)/18:1(9Z))[iso6]	LMGL03014984
TG 18:1_30:2	TG(15:1(9Z)/15:1(9Z)/18:1(9Z))[iso3]	LMGL03012933
	TG(12:0/18:1(9Z)/18:2(9Z,12Z))[iso6]	LMGL03013462
	TG(13:0/17:2(9Z,12Z)/18:1(9Z))[iso6]	LMGL03013885
	TG(14:1(9Z)/16:1(9Z)/18:1(9Z))[iso6]	LMGL03014657
TG 18:1_31:0	TG(12:0/18:1(9Z)/19:0)[iso6]	LMGL03013466
	TG(13:0/18:0/18:1(9Z))[iso6]	LMGL03013906
	TG(14:0/17:0/18:1(9Z))[iso6]	LMGL03014275
	TG(15:0/16:0/18:1(9Z))[iso6]	LMGL03015010
TG 18:1_32:0	TG(16:0/16:0/18:1(11E))	LMGL03010005
	TG(16:0/16:0/18:1(9Z))	LMGL03010006
	TG(12:0/18:1(9Z)/20:0)[iso6]	LMGL03013468
	TG(13:0/18:1(9Z)/19:0)[iso6]	LMGL03013931
	TG(14:0/18:0/18:1(9Z))[iso6]	LMGL03014341
	TG(15:0/17:0/18:1(9Z))[iso6]	LMGL03015059
TG 18:1_32:1	TG(16:0/16:1(9Z)/18:1(9Z))[iso6]	LMGL03010043
	TG(14:0/18:1(9Z)/18:1(9Z))[iso3]	LMGL03012792
	TG(12:0/18:1(9Z)/20:1(11Z))[iso6]	LMGL03013469
	TG(13:0/18:1(9Z)/19:1(9Z))[iso6]	LMGL03013932
	TG(14:1(9Z)/18:0/18:1(9Z))[iso6]	LMGL03014747
	TG(15:0/17:1(9Z)/18:1(9Z))[iso6]	LMGL03015082
	TG(15:1(9Z)/17:0/18:1(9Z))[iso6]	LMGL03015410
TG 18:1_32:2	TG(16:1(9Z)/16:1(9Z)/18:1(9Z))[iso3]	LMGL03010052
	TG(14:1(9Z)/18:1(9Z)/18:1(9Z))[iso3]	LMGL03012850
	TG(12:0/18:1(9Z)/20:2(11Z,14Z))[iso6]	LMGL03013470
	TG(14:0/18:1(9Z)/18:2(9Z,12Z))[iso6]	LMGL03014362
	TG(15:0/17:2(9Z,12Z)/18:1(9Z))[iso6]	LMGL03015104
	TG(15:1(9Z)/17:1(9Z)/18:1(9Z))[iso6]	LMGL03015433
TG 18:1_32:3	TG(12:0/18:1(9Z)/20:3(8Z,11Z,14Z))[iso6]	LMGL03013471
	TG(14:0/18:1(9Z)/18:3(6Z,9Z,12Z))[iso6]	LMGL03014363
	TG(14:0/18:1(9Z)/18:3(9Z,12Z,15Z))[iso6]	LMGL03014364
	TG(14:1(9Z)/18:1(9Z)/18:2(9Z,12Z))[iso6]	LMGL03014768
	TG(15:1(9Z)/17:2(9Z,12Z)/18:1(9Z))[iso6]	LMGL03015455
TG 18:1_33:0	TG(16:0/17:0/18:1(9Z))[iso6]	LMGL03010051
	TG(12:0/18:1(9Z)/21:0)[iso6]	LMGL03013474
	TG(13:0/18:1(9Z)/20:0)[iso6]	LMGL03013933
	TG(14:0/18:1(9Z)/19:0)[iso6]	LMGL03014366
	TG(15:0/18:0/18:1(9Z))[iso6]	LMGL03015125
TG 18:1_33:1	TG(16:0/17:1(9Z)/18:1(9Z))[iso6]	LMGL03010061
	TG(16:1(9Z)/17:0/18:1(9Z))[iso6]	LMGL03010062
	TG(15:0/18:1(9Z)/18:1(9Z))[iso3]	LMGL03012906
	TG(13:0/18:1(9Z)/20:1(11Z))[iso6]	LMGL03013934
	TG(14:0/18:1(9Z)/19:1(9Z))[iso6]	LMGL03014367
	TG(14:1(9Z)/18:1(9Z)/19:0)[iso6]	LMGL03014772
	TG(15:1(9Z)/18:0/18:1(9Z))[iso6]	LMGL03015476
TG 18:1_33:2	TG(16:0/17:2(9Z,12Z)/18:1(9Z))[iso6]	LMGL03010072
	TG(16:1(9Z)/17:1(9Z)/18:1(9Z))[iso6]	LMGL03010073
	TG(15:1(9Z)/18:1(9Z)/18:1(9Z))[iso3]	LMGL03012960
	TG(13:0/18:1(9Z)/20:2(11Z,14Z))[iso6]	LMGL03013935
	TG(14:1(9Z)/18:1(9Z)/19:1(9Z))[iso6]	LMGL03014773
	TG(15:0/18:1(9Z)/18:2(9Z,12Z))[iso6]	LMGL03015146

MxP® Quant 500 XL kit lipid annotation	Potential isomers	Data base ID
TG 18:1_33:3	TG(16:1(9Z)/17:2(9Z,12Z)/18:1(9Z))[iso6]	LMGL03010086
	TG(13:0/18:1(9Z)/20:3(8Z,11Z,14Z))[iso6]	LMGL03013936
	TG(15:0/18:1(9Z)/18:3(6Z,9Z,12Z))[iso6]	LMGL03015147
	TG(15:0/18:1(9Z)/18:3(9Z,12Z,15Z))[iso6]	LMGL03015148
	TG(15:1(9Z)/18:1(9Z)/18:2(9Z,12Z))[iso6]	LMGL03015497
TG 18:1_34:1	TG(17:0/17:1(9Z)/18:1(9Z))[iso6]	LMGL03010087
	TG(16:0/18:1(9Z)/18:1(9Z))[iso3]	LMGL03010100
	TG(16:1(9Z)/18:0/18:1(9Z))[iso6]	LMGL03010101
	TG(12:0/18:1(9Z)/22:1(11Z))[iso6]	LMGL03013476
	TG(14:0/18:1(9Z)/20:1(11Z))[iso6]	LMGL03014369
	TG(14:1(9Z)/18:1(9Z)/20:0)[iso6]	LMGL03014774
	TG(15:0/18:1(9Z)/19:1(9Z))[iso6]	LMGL03015151
	TG(15:1(9Z)/18:1(9Z)/19:0)[iso6]	LMGL03015501
TG 18:1_34:2	TG(17:0/17:2(9Z,12Z)/18:1(9Z))[iso6]	LMGL03010102
	TG(17:1(9Z)/17:1(9Z)/18:1(9Z))[iso3]	LMGL03010103
	TG(16:1(9Z)/18:1(9Z)/18:1(9Z))[iso3]	LMGL03010118
	TG(16:0/18:1(9Z)/18:2(9Z,12Z))[iso6]	LMGL03010121
	TG(12:0/18:1(9Z)/22:2(13Z,16Z))[iso6]	LMGL03013477
	TG(14:0/18:1(9Z)/20:2(11Z,14Z))[iso6]	LMGL03014370
	TG(14:1(9Z)/18:1(9Z)/20:1(11Z))[iso6]	LMGL03014775
	TG(15:1(9Z)/18:1(9Z)/19:1(9Z))[iso6]	LMGL03015502
TG 18:1_34:3	TG(17:1(9Z)/17:2(9Z,12Z)/18:1(9Z))[iso6]	LMGL03010120
	TG(16:1(9Z)/18:1(9Z)/18:2(9Z,12Z))[iso6]	LMGL03010142
	TG(16:0/18:1(9Z)/18:3(9Z,12Z,15Z))[iso6]	LMGL03010145
	TG(12:0/18:1(9Z)/22:3(10Z,13Z,16Z))[iso6]	LMGL03013478
	TG(14:0/18:1(9Z)/20:3(8Z,11Z,14Z))[iso6]	LMGL03014371
	TG(14:1(9Z)/18:1(9Z)/20:2(11Z,14Z))[iso6]	LMGL03014776
	TG(16:0/18:1(9Z)/18:3(6Z,9Z,12Z))[iso6]	LMGL03015727
TG 18:1_34:4	TG(17:2(9Z,12Z)/17:2(9Z,12Z)/18:1(9Z))[iso3]	LMGL03010140
	TG(16:1(9Z)/18:1(9Z)/18:3(9Z,12Z,15Z))[iso6]	LMGL03010168
	TG(12:0/18:1(9Z)/22:4(7Z,10Z,13Z,16Z))[iso6]	LMGL03013479
	TG(14:0/18:1(9Z)/20:4(5Z,8Z,11Z,14Z))[iso6]	LMGL03014372
	TG(14:1(9Z)/18:1(9Z)/20:3(8Z,11Z,14Z))[iso6]	LMGL03014777
	TG(16:0/18:1(9Z)/18:4(6Z,9Z,12Z,15Z))[iso6]	LMGL03015728
	TG(16:1(9Z)/18:1(9Z)/18:3(6Z,9Z,12Z))[iso6]	LMGL03015818
TG 18:1_35:2	TG(17:1(9Z)/18:1(9Z)/18:1(9Z))[iso3]	LMGL03010161
	TG(17:2(9Z,12Z)/18:0/18:1(9Z))[iso6]	LMGL03010162
	TG(17:0/18:1(9Z)/18:2(9Z,12Z))[iso6]	LMGL03010164
	TG(13:0/18:1(9Z)/22:2(13Z,16Z))[iso6]	LMGL03013942
	TG(15:0/18:1(9Z)/20:2(11Z,14Z))[iso6]	LMGL03015154
	TG(15:1(9Z)/18:1(9Z)/20:1(11Z))[iso6]	LMGL03015504
	TG(16:1(9Z)/18:1(9Z)/19:1(9Z))[iso6]	LMGL03015820
TG 18:1_35:3	TG(17:2(9Z,12Z)/18:1(9Z)/18:1(9Z))[iso3]	LMGL03010187
	TG(17:1(9Z)/18:1(9Z)/18:2(9Z,12Z))[iso6]	LMGL03010190
	TG(17:0/18:1(9Z)/18:3(9Z,12Z,15Z))[iso6]	LMGL03010194
	TG(13:0/18:1(9Z)/22:3(10Z,13Z,16Z))[iso6]	LMGL03013943
	TG(15:0/18:1(9Z)/20:3(8Z,11Z,14Z))[iso6]	LMGL03015155
	TG(15:1(9Z)/18:1(9Z)/20:2(11Z,14Z))[iso6]	LMGL03015505
	TG(17:0/18:1(9Z)/18:3(6Z,9Z,12Z))[iso6]	LMGL03015905
TG 18:1_36:0	TG(18:0/18:0/18:1(9Z))[iso3]	LMGL03010188
	TG(16:0/18:1(9Z)/20:0)[iso6]	LMGL03010201
	TG(17:0/18:1(9Z)/19:0)[iso6]	LMGL03010227
	TG(17:2(9Z,12Z)/18:1(9Z)/20:5(5Z,8Z,11Z,14Z,17Z))[iso6]	LMGL03010620
	TG(14:0/18:1(9Z)/22:0)[iso6]	LMGL03014375
	TG(15:0/18:1(9Z)/21:0)[iso6]	LMGL03015158
	TG(15:1(9Z)/18:1(9Z)/22:6(4Z,7Z,10Z,13Z,16Z,19Z))[iso6]	LMGL03015516

MxP® Quant 500 XL kit lipid annotation	Potential isomers	Data base ID
TG 18:1_36:1	TG(18:0/18:1(9Z)/18:1(9Z))[iso3]	LMGL03010217
	TG(16:1(9Z)/18:1(9Z)/20:0)[iso6]	LMGL03010231
	TG(16:0/18:1(9Z)/20:1(11Z))[iso6]	LMGL03010234
	TG(17:1(9Z)/18:1(9Z)/19:0)[iso6]	LMGL03010260
	TG(14:0/18:1(9Z)/22:1(11Z))[iso6]	LMGL03014376
	TG(14:1(9Z)/18:1(9Z)/22:0)[iso6]	LMGL03014781
	TG(15:1(9Z)/18:1(9Z)/21:0)[iso6]	LMGL03015509
	TG(17:0/18:1(9Z)/19:1(9Z))[iso6]	LMGL03015907
TG 18:1_36:2	TG(18:1(9Z)/18:1(9Z)/18:1(9Z))	LMGL03010250
	TG(18:0/18:1(9Z)/18:2(9Z,12Z))[iso6]	LMGL03010252
	TG(16:1(9Z)/18:1(9Z)/20:1(11Z))[iso6]	LMGL03010268
	TG(16:0/18:1(9Z)/20:2(11Z,14Z))[iso6]	LMGL03010271
	TG(17:2(9Z,12Z)/18:1(9Z)/19:0)[iso6]	LMGL03010295
	TG(18:1(9Z)/18:1(6Z)/18:1(9Z))	LMGL03012612
	TG(18:1(6Z)/18:1(9Z)/18:1(6Z))	LMGL03012613
	TG(14:0/18:1(9Z)/22:2(13Z,16Z))[iso6]	LMGL03014377
	TG(14:1(9Z)/18:1(9Z)/22:1(11Z))[iso6]	LMGL03014782
	TG(17:1(9Z)/18:1(9Z)/19:1(9Z))[iso6]	LMGL03015990
TG 18:1_36:3	TG(18:1(9Z)/18:1(9Z)/18:2(9Z,12Z))[iso3]	LMGL03010288
	TG(18:0/18:1(9Z)/18:3(9Z,12Z,15Z))[iso6]	LMGL03010291
	TG(16:1(9Z)/18:1(9Z)/20:2(11Z,14Z))[iso6]	LMGL03010308
	TG(16:0/18:1(9Z)/20:3(8Z,11Z,14Z))[iso6]	LMGL03010311
	TG(14:0/18:1(9Z)/22:3(10Z,13Z,16Z))[iso6]	LMGL03014378
	TG(14:1(9Z)/18:1(9Z)/22:2(13Z,16Z))[iso6]	LMGL03014783
	TG(17:2(9Z,12Z)/18:1(9Z)/19:1(9Z))[iso6]	LMGL03016069
	TG(18:0/18:1(9Z)/18:3(6Z,9Z,12Z))[iso6]	LMGL03016142
TG 18:1_36:4	TG(18:1(9Z)/18:2(9Z,12Z)/18:2(9Z,12Z))[iso3]	LMGL03010327
	TG(18:1(9Z)/18:1(9Z)/18:3(9Z,12Z,15Z))[iso3]	LMGL03010330
	TG(16:1(9Z)/18:1(9Z)/20:3(8Z,11Z,14Z))[iso6]	LMGL03010352
	TG(16:0/18:1(9Z)/20:4(5Z,8Z,11Z,14Z))[iso6]	LMGL03010355
	TG(18:1(9Z)/18:1(9Z)/18:3(6Z,9Z,12Z))[iso3]	LMGL03013029
	TG(14:0/18:1(9Z)/22:4(7Z,10Z,13Z,16Z))[iso6]	LMGL03014379
	TG(14:1(9Z)/18:1(9Z)/22:3(10Z,13Z,16Z))[iso6]	LMGL03014784
	TG(18:0/18:1(9Z)/18:4(6Z,9Z,12Z,15Z))[iso6]	LMGL03016143
TG 18:1_36:5	TG(18:1(9Z)/18:2(9Z,12Z)/18:3(9Z,12Z,15Z))[iso6]	LMGL03010373
	TG(16:1(9Z)/18:1(9Z)/20:4(5Z,8Z,11Z,14Z))[iso6]	LMGL03010400
	TG(16:0/18:1(9Z)/20:5(5Z,8Z,11Z,14Z,17Z))[iso6]	LMGL03010403
	TG(18:1(9Z)/18:1(9Z)/18:4(6Z,9Z,12Z,15Z))[iso3]	LMGL03013030
	TG(14:0/18:1(9Z)/22:5(7Z,10Z,13Z,16Z,19Z))[iso6]	LMGL03014380
	TG(14:1(9Z)/18:1(9Z)/22:4(7Z,10Z,13Z,16Z))[iso6]	LMGL03014785
	TG(18:1(9Z)/18:2(9Z,12Z)/18:3(6Z,9Z,12Z))[iso6]	LMGL03016217
TG 18:1_36:6	TG(18:1(9Z)/18:3(9Z,12Z,15Z)/18:3(9Z,12Z,15Z))[iso3]	LMGL03010419
	TG(16:1(9Z)/18:1(9Z)/20:5(5Z,8Z,11Z,14Z,17Z))[iso6]	LMGL03010452
	TG(18:1(9Z)/18:3(6Z,9Z,12Z)/18:3(6Z,9Z,12Z))[iso3]	LMGL03013033
	TG(14:0/18:1(9Z)/22:6(4Z,7Z,10Z,13Z,16Z,19Z))[iso6]	LMGL03014381
	TG(14:1(9Z)/18:1(9Z)/22:5(7Z,10Z,13Z,16Z,19Z))[iso6]	LMGL03014786
	TG(18:1(9Z)/18:2(9Z,12Z)/18:4(6Z,9Z,12Z,15Z))[iso6]	LMGL03016218
	TG(18:1(9Z)/18:3(6Z,9Z,12Z)/18:3(9Z,12Z,15Z))[iso6]	LMGL03016221
TG 18:1_38:5	TG(18:1(9Z)/18:3(9Z,12Z,15Z)/20:2(11Z,14Z))[iso6]	LMGL03010662
	TG(18:1(9Z)/18:2(9Z,12Z)/20:3(8Z,11Z,14Z))[iso6]	LMGL03010670
	TG(18:1(9Z)/18:1(9Z)/20:4(5Z,8Z,11Z,14Z))[iso3]	LMGL03010677
	TG(18:0/18:1(9Z)/20:5(5Z,8Z,11Z,14Z,17Z))[iso6]	LMGL03010683
	TG(16:1(9Z)/18:1(9Z)/22:4(7Z,10Z,13Z,16Z))[iso6]	LMGL03010846
	TG(16:0/18:1(9Z)/22:5(7Z,10Z,13Z,16Z,19Z))[iso6]	LMGL03010849
	TG(18:1(9Z)/18:3(6Z,9Z,12Z)/20:2(11Z,14Z))[iso6]	LMGL03016227
	TG(18:1(9Z)/18:4(6Z,9Z,12Z,15Z)/20:1(11Z))[iso6]	LMGL03016245

MxP® Quant 500 XL kit lipid annotation	Potential isomers	Data base ID
TG 18:1_38:6	TG(18:1(9Z)/18:3(9Z,12Z,15Z)/20:3(8Z,11Z,14Z))[iso6]	LMGL03010733
	TG(18:1(9Z)/18:2(9Z,12Z)/20:4(5Z,8Z,11Z,14Z))[iso6]	LMGL03010741
	TG(18:1(9Z)/18:1(9Z)/20:5(5Z,8Z,11Z,14Z,17Z))[iso3]	LMGL03010748
	TG(16:1(9Z)/18:1(9Z)/22:5(7Z,10Z,13Z,16Z,19Z))[iso6]	LMGL03010922
	TG(16:0/18:1(9Z)/22:6(4Z,7Z,10Z,13Z,16Z,19Z))[iso6]	LMGL03010925
	TG(18:1(9Z)/18:3(6Z,9Z,12Z)/20:3(8Z,11Z,14Z))[iso6]	LMGL03016228
	TG(18:1(9Z)/18:4(6Z,9Z,12Z,15Z)/20:2(11Z,14Z))[iso6]	LMGL03016246
TG 18:1_38:7	TG(17:0/18:1(9Z)/20:0)[iso6]	LMGL03010264
	TG(18:0/18:1(9Z)/19:0)[iso6]	LMGL03010334
	TG(16:0/18:1(9Z)/21:0)[iso6]	LMGL03010455
	TG(18:1(9Z)/18:3(9Z,12Z,15Z)/20:4(5Z,8Z,11Z,14Z))[iso6]	LMGL03010807
	TG(18:1(9Z)/18:2(9Z,12Z)/20:5(5Z,8Z,11Z,14Z,17Z))[iso6]	LMGL03010815
	TG(16:1(9Z)/18:1(9Z)/22:6(4Z,7Z,10Z,13Z,16Z,19Z))[iso6]	LMGL03011000
	TG(15:0/18:1(9Z)/22:0)[iso6]	LMGL03015159
	TG(18:1(9Z)/18:3(6Z,9Z,12Z)/20:4(5Z,8Z,11Z,14Z))[iso6]	LMGL03016229
	TG(18:1(9Z)/18:4(6Z,9Z,12Z,15Z)/20:3(8Z,11Z,14Z))[iso6]	LMGL03016247
TG 18:2_28:0	TG(14:0/14:0/18:2(9Z,12Z))[iso3]	LMGL03012763
	TG(12:0/16:0/18:2(9Z,12Z))[iso6]	LMGL03013327
	TG(13:0/15:0/18:2(9Z,12Z))[iso6]	LMGL03013739
TG 18:2_30:0	TG(15:0/15:0/18:2(9Z,12Z))[iso3]	LMGL03012879
	TG(12:0/18:0/18:2(9Z,12Z))[iso6]	LMGL03013442
	TG(13:0/17:0/18:2(9Z,12Z))[iso6]	LMGL03013841
	TG(14:0/16:0/18:2(9Z,12Z))[iso6]	LMGL03014227
TG 18:2_30:1	TG(12:0/18:1(9Z)/18:2(9Z,12Z))[iso6]	LMGL03013462
	TG(13:0/17:1(9Z)/18:2(9Z,12Z))[iso6]	LMGL03013864
	TG(14:0/16:1(9Z)/18:2(9Z,12Z))[iso6]	LMGL03014252
	TG(14:1(9Z)/16:0/18:2(9Z,12Z))[iso6]	LMGL03014633
	TG(15:0/15:1(9Z)/18:2(9Z,12Z))[iso6]	LMGL03014985
TG 18:2_31:0	TG(12:0/18:2(9Z,12Z)/19:0)[iso6]	LMGL03013485
	TG(13:0/18:0/18:2(9Z,12Z))[iso6]	LMGL03013907
	TG(14:0/17:0/18:2(9Z,12Z))[iso6]	LMGL03014276
	TG(15:0/16:0/18:2(9Z,12Z))[iso6]	LMGL03015011
TG 18:2_32:0	TG(16:0/16:0/18:2(9Z,12Z))[iso3]	LMGL03010044
	TG(12:0/18:2(9Z,12Z)/20:0)[iso6]	LMGL03013487
	TG(13:0/18:2(9Z,12Z)/19:0)[iso6]	LMGL03013950
	TG(14:0/18:0/18:2(9Z,12Z))[iso6]	LMGL03014342
	TG(15:0/17:0/18:2(9Z,12Z))[iso6]	LMGL03015060
TG 18:2_32:1	TG(16:0/16:1(9Z)/18:2(9Z,12Z))[iso6]	LMGL03010053
	TG(12:0/18:2(9Z,12Z)/20:1(11Z))[iso6]	LMGL03013488
	TG(13:0/18:2(9Z,12Z)/19:1(9Z))[iso6]	LMGL03013951
	TG(14:0/18:1(9Z)/18:2(9Z,12Z))[iso6]	LMGL03014362
	TG(14:1(9Z)/18:0/18:2(9Z,12Z))[iso6]	LMGL03014748
	TG(15:0/17:1(9Z)/18:2(9Z,12Z))[iso6]	LMGL03015083
	TG(15:1(9Z)/17:0/18:2(9Z,12Z))[iso6]	LMGL03015411
TG 18:2_32:2	TG(16:1(9Z)/16:1(9Z)/18:2(9Z,12Z))[iso3]	LMGL03010064
	TG(14:0/18:2(9Z,12Z)/18:2(9Z,12Z))[iso3]	LMGL03012793
	TG(12:0/18:2(9Z,12Z)/20:2(11Z,14Z))[iso6]	LMGL03013489
	TG(14:1(9Z)/18:1(9Z)/18:2(9Z,12Z))[iso6]	LMGL03014768
	TG(15:0/17:2(9Z,12Z)/18:2(9Z,12Z))[iso6]	LMGL03015105
	TG(15:1(9Z)/17:1(9Z)/18:2(9Z,12Z))[iso6]	LMGL03015434
TG 18:2_33:0	TG(16:0/17:0/18:2(9Z,12Z))[iso6]	LMGL03010063
	TG(12:0/18:2(9Z,12Z)/21:0)[iso6]	LMGL03013493
	TG(13:0/18:2(9Z,12Z)/20:0)[iso6]	LMGL03013952
	TG(14:0/18:2(9Z,12Z)/19:0)[iso6]	LMGL03014385
	TG(15:0/18:0/18:2(9Z,12Z))[iso6]	LMGL03015126

MxP® Quant 500 XL kit lipid annotation	Potential isomers	Data base ID
TG 18:2_33:1	TG(16:0/17:1(9Z)/18:2(9Z,12Z))[iso6]	LMGL03010075
	TG(16:1(9Z)/17:0/18:2(9Z,12Z))[iso6]	LMGL03010076
	TG(13:0/18:2(9Z,12Z)/20:1(11Z))[iso6]	LMGL03013953
	TG(14:0/18:2(9Z,12Z)/19:1(9Z))[iso6]	LMGL03014386
	TG(14:1(9Z)/18:2(9Z,12Z)/19:0)[iso6]	LMGL03014791
	TG(15:0/18:1(9Z)/18:2(9Z,12Z))[iso6]	LMGL03015146
	TG(15:1(9Z)/18:0/18:2(9Z,12Z))[iso6]	LMGL03015477
TG 18:2_33:2	TG(16:0/17:2(9Z,12Z)/18:2(9Z,12Z))[iso6]	LMGL03010088
	TG(16:1(9Z)/17:1(9Z)/18:2(9Z,12Z))[iso6]	LMGL03010089
	TG(15:0/18:2(9Z,12Z)/18:2(9Z,12Z))[iso3]	LMGL03012907
	TG(13:0/18:2(9Z,12Z)/20:2(11Z,14Z))[iso6]	LMGL03013954
	TG(14:1(9Z)/18:2(9Z,12Z)/19:1(9Z))[iso6]	LMGL03014792
	TG(15:1(9Z)/18:1(9Z)/18:2(9Z,12Z))[iso6]	LMGL03015497
TG 18:2_34:0	TG(17:0/17:0/18:2(9Z,12Z))[iso3]	LMGL03010090
	TG(16:0/18:0/18:2(9Z,12Z))[iso6]	LMGL03010104
	TG(12:0/18:2(9Z,12Z)/22:0)[iso6]	LMGL03013494
	TG(13:0/18:2(9Z,12Z)/21:0)[iso6]	LMGL03013958
	TG(14:0/18:2(9Z,12Z)/20:0)[iso6]	LMGL03014387
	TG(15:0/18:2(9Z,12Z)/19:0)[iso6]	LMGL03015169
TG 18:2_34:1	TG(17:0/17:1(9Z)/18:2(9Z,12Z))[iso6]	LMGL03010106
	TG(16:0/18:1(9Z)/18:2(9Z,12Z))[iso6]	LMGL03010121
	TG(16:1(9Z)/18:0/18:2(9Z,12Z))[iso6]	LMGL03010122
	TG(12:0/18:2(9Z,12Z)/22:1(11Z))[iso6]	LMGL03013495
	TG(14:0/18:2(9Z,12Z)/20:1(11Z))[iso6]	LMGL03014388
	TG(14:1(9Z)/18:2(9Z,12Z)/20:0)[iso6]	LMGL03014793
	TG(15:0/18:2(9Z,12Z)/19:1(9Z))[iso6]	LMGL03015170
	TG(15:1(9Z)/18:2(9Z,12Z)/19:0)[iso6]	LMGL03015520
TG 18:2_34:2	TG(17:0/17:2(9Z,12Z)/18:2(9Z,12Z))[iso6]	LMGL03010123
	TG(17:1(9Z)/17:1(9Z)/18:2(9Z,12Z))[iso3]	LMGL03010124
	TG(16:0/18:2(9Z,12Z)/18:2(9Z,12Z))[iso3]	LMGL03010141
	TG(16:1(9Z)/18:1(9Z)/18:2(9Z,12Z))[iso6]	LMGL03010142
	TG(12:0/18:2(9Z,12Z)/22:2(13Z,16Z))[iso6]	LMGL03013496
	TG(14:0/18:2(9Z,12Z)/20:2(11Z,14Z))[iso6]	LMGL03014389
	TG(14:1(9Z)/18:2(9Z,12Z)/20:1(11Z))[iso6]	LMGL03014794
	TG(15:1(9Z)/18:2(9Z,12Z)/19:1(9Z))[iso6]	LMGL03015521
TG 18:2_34:3	TG(17:1(9Z)/17:2(9Z,12Z)/18:2(9Z,12Z))[iso6]	LMGL03010144
	TG(16:1(9Z)/18:2(9Z,12Z)/18:2(9Z,12Z))[iso3]	LMGL03010163
	TG(16:0/18:2(9Z,12Z)/18:3(9Z,12Z,15Z))[iso6]	LMGL03010167
	TG(12:0/18:2(9Z,12Z)/22:3(10Z,13Z,16Z))[iso6]	LMGL03013497
	TG(14:0/18:2(9Z,12Z)/20:3(8Z,11Z,14Z))[iso6]	LMGL03014390
	TG(14:1(9Z)/18:2(9Z,12Z)/20:2(11Z,14Z))[iso6]	LMGL03014795
	TG(16:0/18:2(9Z,12Z)/18:3(6Z,9Z,12Z))[iso6]	LMGL03015731
TG 18:2_34:4	TG(17:2(9Z,12Z)/17:2(9Z,12Z)/18:2(9Z,12Z))[iso3]	LMGL03010166
	TG(16:1(9Z)/18:2(9Z,12Z)/18:3(9Z,12Z,15Z))[iso6]	LMGL03010193
	TG(12:0/18:2(9Z,12Z)/22:4(7Z,10Z,13Z,16Z))[iso6]	LMGL03013498
	TG(14:0/18:2(9Z,12Z)/20:4(5Z,8Z,11Z,14Z))[iso6]	LMGL03014391
	TG(14:1(9Z)/18:2(9Z,12Z)/20:3(8Z,11Z,14Z))[iso6]	LMGL03014796
	TG(16:0/18:2(9Z,12Z)/18:4(6Z,9Z,12Z,15Z))[iso6]	LMGL03015732
	TG(16:1(9Z)/18:2(9Z,12Z)/18:3(6Z,9Z,12Z))[iso6]	LMGL03015822

MxP® Quant 500 XL kit lipid annotation	Potential isomers	Data base ID
TG 18:2_35:1	TG(17:0/18:1(9Z)/18:2(9Z,12Z))[iso6]	LMGL03010164
	TG(17:1(9Z)/18:0/18:2(9Z,12Z))[iso6]	LMGL03010165
	TG(16:1(9Z)/18:2(9Z,12Z)/19:0)[iso6]	LMGL03010226
	TG(18:2(9Z,12Z)/18:4(6Z,9Z,12Z,15Z)/18:4(6Z,9Z,12Z,15Z))[iso3]	LMGL03013042
	TG(13:0/18:2(9Z,12Z)/22:1(11Z))[iso6]	LMGL03013960
	TG(14:1(9Z)/18:2(9Z,12Z)/21:0)[iso6]	LMGL03014799
	TG(15:0/18:2(9Z,12Z)/20:1(11Z))[iso6]	LMGL03015172
	TG(15:1(9Z)/18:2(9Z,12Z)/20:0)[iso6]	LMGL03015522
	TG(16:0/18:2(9Z,12Z)/19:1(9Z))[iso6]	LMGL03015733
TG 18:2_35:2	TG(17:0/18:2(9Z,12Z)/18:2(9Z,12Z))[iso3]	LMGL03010189
	TG(17:1(9Z)/18:1(9Z)/18:2(9Z,12Z))[iso6]	LMGL03010190
	TG(17:2(9Z,12Z)/18:0/18:2(9Z,12Z))[iso6]	LMGL03010191
	TG(13:0/18:2(9Z,12Z)/22:2(13Z,16Z))[iso6]	LMGL03013961
	TG(15:0/18:2(9Z,12Z)/20:2(11Z,14Z))[iso6]	LMGL03015173
	TG(15:1(9Z)/18:2(9Z,12Z)/20:1(11Z))[iso6]	LMGL03015523
	TG(16:1(9Z)/18:2(9Z,12Z)/19:1(9Z))[iso6]	LMGL03015824
TG 18:2_35:3	TG(17:1(9Z)/18:2(9Z,12Z)/18:2(9Z,12Z))[iso3]	LMGL03010218
	TG(17:2(9Z,12Z)/18:1(9Z)/18:2(9Z,12Z))[iso6]	LMGL03010219
	TG(17:0/18:2(9Z,12Z)/18:3(9Z,12Z,15Z))[iso6]	LMGL03010222
	TG(13:0/18:2(9Z,12Z)/22:3(10Z,13Z,16Z))[iso6]	LMGL03013962
	TG(15:0/18:2(9Z,12Z)/20:3(8Z,11Z,14Z))[iso6]	LMGL03015174
	TG(15:1(9Z)/18:2(9Z,12Z)/20:2(11Z,14Z))[iso6]	LMGL03015524
	TG(17:0/18:2(9Z,12Z)/18:3(6Z,9Z,12Z))[iso6]	LMGL03015909
TG 18:2_36:0	TG(18:0/18:0/18:2(9Z,12Z))[iso3]	LMGL03010220
	TG(16:0/18:2(9Z,12Z)/20:0)[iso6]	LMGL03010230
	TG(17:0/18:2(9Z,12Z)/19:0)[iso6]	LMGL03010259
	TG(17:2(9Z,12Z)/18:2(9Z,12Z)/20:5(5Z,8Z,11Z,14Z,17Z))[iso6]	LMGL03010682
	TG(14:0/18:2(9Z,12Z)/22:0)[iso6]	LMGL03014394
	TG(15:0/18:2(9Z,12Z)/21:0)[iso6]	LMGL03015177
	TG(15:1(9Z)/18:2(9Z,12Z)/22:6(4Z,7Z,10Z,13Z,16Z,19Z))[iso6]	LMGL03015535
TG 18:2_36:1	TG(18:0/18:1(9Z)/18:2(9Z,12Z))[iso6]	LMGL03010252
	TG(16:1(9Z)/18:2(9Z,12Z)/20:0)[iso6]	LMGL03010263
	TG(16:0/18:2(9Z,12Z)/20:1(11Z))[iso6]	LMGL03010267
	TG(17:1(9Z)/18:2(9Z,12Z)/19:0)[iso6]	LMGL03010294
	TG(14:0/18:2(9Z,12Z)/22:1(11Z))[iso6]	LMGL03014395
	TG(14:1(9Z)/18:2(9Z,12Z)/22:0)[iso6]	LMGL03014800
	TG(15:1(9Z)/18:2(9Z,12Z)/21:0)[iso6]	LMGL03015528
	TG(17:0/18:2(9Z,12Z)/19:1(9Z))[iso6]	LMGL03015911
TG 18:2_36:2	TG(18:0/18:2(9Z,12Z)/18:2(9Z,12Z))[iso3]	LMGL03010287
	TG(18:1(9Z)/18:1(9Z)/18:2(9Z,12Z))[iso3]	LMGL03010288
	TG(16:1(9Z)/18:2(9Z,12Z)/20:1(11Z))[iso6]	LMGL03010303
	TG(16:0/18:2(9Z,12Z)/20:2(11Z,14Z))[iso6]	LMGL03010307
	TG(17:2(9Z,12Z)/18:2(9Z,12Z)/19:0)[iso6]	LMGL03010333
	TG(14:0/18:2(9Z,12Z)/22:2(13Z,16Z))[iso6]	LMGL03014396
	TG(14:1(9Z)/18:2(9Z,12Z)/22:1(11Z))[iso6]	LMGL03014801
	TG(17:1(9Z)/18:2(9Z,12Z)/19:1(9Z))[iso6]	LMGL03015994
TG 18:2_36:3	TG(18:1(9Z)/18:2(9Z,12Z)/18:2(9Z,12Z))[iso3]	LMGL03010327
	TG(18:0/18:2(9Z,12Z)/18:3(9Z,12Z,15Z))[iso6]	LMGL03010329
	TG(16:1(9Z)/18:2(9Z,12Z)/20:2(11Z,14Z))[iso6]	LMGL03010347
	TG(16:0/18:2(9Z,12Z)/20:3(8Z,11Z,14Z))[iso6]	LMGL03010351
	TG(14:0/18:2(9Z,12Z)/22:3(10Z,13Z,16Z))[iso6]	LMGL03014397
	TG(14:1(9Z)/18:2(9Z,12Z)/22:2(13Z,16Z))[iso6]	LMGL03014802
	TG(17:2(9Z,12Z)/18:2(9Z,12Z)/19:1(9Z))[iso6]	LMGL03016073
	TG(18:0/18:2(9Z,12Z)/18:3(6Z,9Z,12Z))[iso6]	LMGL03016146

MxP® Quant 500 XL kit lipid annotation	Potential isomers	Data base ID
TG 18:2_36:4	TG(18:2(9Z,12Z)/18:2(9Z,12Z)/18:2(9Z,12Z))	LMGL03010371
	TG(18:1(9Z)/18:2(9Z,12Z)/18:3(9Z,12Z,15Z))[iso6]	LMGL03010373
	TG(16:1(9Z)/18:2(9Z,12Z)/20:3(8Z,11Z,14Z))[iso6]	LMGL03010395
	TG(16:0/18:2(9Z,12Z)/20:4(5Z,8Z,11Z,14Z))[iso6]	LMGL03010399
	TG(14:0/18:2(9Z,12Z)/22:4(7Z,10Z,13Z,16Z))[iso6]	LMGL03014398
	TG(14:1(9Z)/18:2(9Z,12Z)/22:3(10Z,13Z,16Z))[iso6]	LMGL03014803
	TG(18:0/18:2(9Z,12Z)/18:4(6Z,9Z,12Z,15Z))[iso6]	LMGL03016147
	TG(18:1(9Z)/18:2(9Z,12Z)/18:3(6Z,9Z,12Z))[iso6]	LMGL03016217
TG 18:2_36:5	TG(18:2(9Z,12Z)/18:2(9Z,12Z)/18:3(9Z,12Z,15Z))[iso3]	LMGL03010420
	TG(16:1(9Z)/18:2(9Z,12Z)/20:4(5Z,8Z,11Z,14Z))[iso6]	LMGL03010447
	TG(16:0/18:2(9Z,12Z)/20:5(5Z,8Z,11Z,14Z,17Z))[iso6]	LMGL03010451
	TG(18:2(9Z,12Z)/18:2(9Z,12Z)/18:3(6Z,9Z,12Z))[iso3]	LMGL03013037
	TG(14:0/18:2(9Z,12Z)/22:5(7Z,10Z,13Z,16Z,19Z))[iso6]	LMGL03014399
	TG(14:1(9Z)/18:2(9Z,12Z)/22:4(7Z,10Z,13Z,16Z))[iso6]	LMGL03014804
	TG(18:1(9Z)/18:2(9Z,12Z)/18:4(6Z,9Z,12Z,15Z))[iso6]	LMGL03016218
TG 18:2_38:4	TG(18:2(9Z,12Z)/18:3(9Z,12Z,15Z)/20:1(11Z))[iso6]	LMGL03010657
	TG(18:2(9Z,12Z)/18:2(9Z,12Z)/20:2(11Z,14Z))[iso3]	LMGL03010663
	TG(18:1(9Z)/18:2(9Z,12Z)/20:3(8Z,11Z,14Z))[iso6]	LMGL03010670
	TG(18:0/18:2(9Z,12Z)/20:4(5Z,8Z,11Z,14Z))[iso6]	LMGL03010676
	TG(16:1(9Z)/18:2(9Z,12Z)/22:3(10Z,13Z,16Z))[iso6]	LMGL03010767
	TG(16:0/18:2(9Z,12Z)/22:4(7Z,10Z,13Z,16Z))[iso6]	LMGL03010845
	TG(18:2(9Z,12Z)/18:3(6Z,9Z,12Z)/20:1(11Z))[iso6]	LMGL03016293
	TG(18:2(9Z,12Z)/18:4(6Z,9Z,12Z,15Z)/20:0)[iso6]	LMGL03016311
TG 18:2_38:5	TG(18:2(9Z,12Z)/18:3(9Z,12Z,15Z)/20:2(11Z,14Z))[iso6]	LMGL03010727
	TG(18:2(9Z,12Z)/18:2(9Z,12Z)/20:3(8Z,11Z,14Z))[iso3]	LMGL03010734
	TG(18:1(9Z)/18:2(9Z,12Z)/20:4(5Z,8Z,11Z,14Z))[iso6]	LMGL03010741
	TG(18:0/18:2(9Z,12Z)/20:5(5Z,8Z,11Z,14Z,17Z))[iso6]	LMGL03010747
	TG(16:1(9Z)/18:2(9Z,12Z)/22:4(7Z,10Z,13Z,16Z))[iso6]	LMGL03010917
	TG(16:0/18:2(9Z,12Z)/22:5(7Z,10Z,13Z,16Z,19Z))[iso6]	LMGL03010921
	TG(18:2(9Z,12Z)/18:3(6Z,9Z,12Z)/20:2(11Z,14Z))[iso6]	LMGL03016294
	TG(18:2(9Z,12Z)/18:4(6Z,9Z,12Z,15Z)/20:1(11Z))[iso6]	LMGL03016312
TG 18:2_38:6	TG(18:2(9Z,12Z)/18:3(9Z,12Z,15Z)/20:3(8Z,11Z,14Z))[iso6]	LMGL03010800
	TG(18:2(9Z,12Z)/18:2(9Z,12Z)/20:4(5Z,8Z,11Z,14Z))[iso3]	LMGL03010808
	TG(18:1(9Z)/18:2(9Z,12Z)/20:5(5Z,8Z,11Z,14Z,17Z))[iso6]	LMGL03010815
	TG(16:1(9Z)/18:2(9Z,12Z)/22:5(7Z,10Z,13Z,16Z,19Z))[iso6]	LMGL03010995
	TG(16:0/18:2(9Z,12Z)/22:6(4Z,7Z,10Z,13Z,16Z,19Z))[iso6]	LMGL03010999
	TG(18:2(9Z,12Z)/18:3(6Z,9Z,12Z)/20:3(8Z,11Z,14Z))[iso6]	LMGL03016295
	TG(18:2(9Z,12Z)/18:4(6Z,9Z,12Z,15Z)/20:2(11Z,14Z))[iso6]	LMGL03016313
TG 18:3_30:0	TG(15:0/15:0/18:3(6Z,9Z,12Z))[iso3]	LMGL03012880
	TG(15:0/15:0/18:3(9Z,12Z,15Z))[iso3]	LMGL03012881
	TG(12:0/18:0/18:3(6Z,9Z,12Z))[iso6]	LMGL03013443
	TG(12:0/18:0/18:3(9Z,12Z,15Z))[iso6]	LMGL03013444
	TG(13:0/17:0/18:3(6Z,9Z,12Z))[iso6]	LMGL03013842
	TG(13:0/17:0/18:3(9Z,12Z,15Z))[iso6]	LMGL03013843
	TG(14:0/16:0/18:3(6Z,9Z,12Z))[iso6]	LMGL03014228
	TG(14:0/16:0/18:3(9Z,12Z,15Z))[iso6]	LMGL03014229
TG 18:3_32:0	TG(16:0/16:0/18:3(9Z,12Z,15Z))[iso3]	LMGL03010054
	TG(16:0/16:0/18:3(6Z,9Z,12Z))[iso3]	LMGL03012981
	TG(12:0/18:3(6Z,9Z,12Z)/20:0)[iso6]	LMGL03013505
	TG(12:0/18:3(9Z,12Z,15Z)/20:0)[iso6]	LMGL03013522
	TG(13:0/18:3(6Z,9Z,12Z)/19:0)[iso6]	LMGL03013968
	TG(13:0/18:3(9Z,12Z,15Z)/19:0)[iso6]	LMGL03013985
	TG(14:0/18:0/18:3(6Z,9Z,12Z))[iso6]	LMGL03014343
	TG(14:0/18:0/18:3(9Z,12Z,15Z))[iso6]	LMGL03014344
	TG(15:0/17:0/18:3(6Z,9Z,12Z))[iso6]	LMGL03015061
	TG(15:0/17:0/18:3(9Z,12Z,15Z))[iso6]	LMGL03015062

MxP® Quant 500 XL kit lipid annotation	Potential isomers	Data base ID
TG 18:3_32:1	TG(16:0/16:1(9Z)/18:3(9Z,12Z,15Z))[iso6]	LMGL03010065
	TG(12:0/18:3(6Z,9Z,12Z)/20:1(11Z))[iso6]	LMGL03013506
	TG(12:0/18:3(9Z,12Z,15Z)/20:1(11Z))[iso6]	LMGL03013523
	TG(13:0/18:3(6Z,9Z,12Z)/19:1(9Z))[iso6]	LMGL03013969
	TG(13:0/18:3(9Z,12Z,15Z)/19:1(9Z))[iso6]	LMGL03013986
	TG(14:0/18:1(9Z)/18:3(6Z,9Z,12Z))[iso6]	LMGL03014363
	TG(14:0/18:1(9Z)/18:3(9Z,12Z,15Z))[iso6]	LMGL03014364
	TG(14:1(9Z)/18:0/18:3(6Z,9Z,12Z))[iso6]	LMGL03014749
	TG(14:1(9Z)/18:0/18:3(9Z,12Z,15Z))[iso6]	LMGL03014750
	TG(15:0/17:1(9Z)/18:3(6Z,9Z,12Z))[iso6]	LMGL03015084
	TG(15:0/17:1(9Z)/18:3(9Z,12Z,15Z))[iso6]	LMGL03015085
	TG(15:1(9Z)/17:0/18:3(6Z,9Z,12Z))[iso6]	LMGL03015412
	TG(15:1(9Z)/17:0/18:3(9Z,12Z,15Z))[iso6]	LMGL03015413
	TG(16:0/16:1(9Z)/18:3(6Z,9Z,12Z))[iso6]	LMGL03015707
TG 18:3_33:2	TG(16:0/17:2(9Z,12Z)/18:3(9Z,12Z,15Z))[iso6]	LMGL03010107
	TG(16:1(9Z)/17:1(9Z)/18:3(9Z,12Z,15Z))[iso6]	LMGL03010108
	TG(13:0/18:3(6Z,9Z,12Z)/20:2(11Z,14Z))[iso6]	LMGL03013972
	TG(13:0/18:3(9Z,12Z,15Z)/20:2(11Z,14Z))[iso6]	LMGL03013989
	TG(14:1(9Z)/18:3(6Z,9Z,12Z)/19:1(9Z))[iso6]	LMGL03014810
	TG(14:1(9Z)/18:3(9Z,12Z,15Z)/19:1(9Z))[iso6]	LMGL03014827
	TG(15:0/18:2(9Z,12Z)/18:3(6Z,9Z,12Z))[iso6]	LMGL03015166
	TG(15:0/18:2(9Z,12Z)/18:3(9Z,12Z,15Z))[iso6]	LMGL03015167
	TG(15:1(9Z)/18:1(9Z)/18:3(6Z,9Z,12Z))[iso6]	LMGL03015498
	TG(15:1(9Z)/18:1(9Z)/18:3(9Z,12Z,15Z))[iso6]	LMGL03015499
	TG(16:0/17:2(9Z,12Z)/18:3(6Z,9Z,12Z))[iso6]	LMGL03015719
	TG(16:1(9Z)/17:1(9Z)/18:3(6Z,9Z,12Z))[iso6]	LMGL03015806
TG 18:3_34:0	TG(17:0/17:0/18:3(9Z,12Z,15Z))[iso3]	LMGL03010109
	TG(16:0/18:0/18:3(9Z,12Z,15Z))[iso6]	LMGL03010125
	TG(17:0/17:0/18:3(6Z,9Z,12Z))[iso3]	LMGL03012997
	TG(12:0/18:3(6Z,9Z,12Z)/22:0)[iso6]	LMGL03013512
	TG(12:0/18:3(9Z,12Z,15Z)/22:0)[iso6]	LMGL03013529
	TG(13:0/18:3(6Z,9Z,12Z)/21:0)[iso6]	LMGL03013976
	TG(13:0/18:3(9Z,12Z,15Z)/21:0)[iso6]	LMGL03013993
	TG(14:0/18:3(6Z,9Z,12Z)/20:0)[iso6]	LMGL03014405
	TG(14:0/18:3(9Z,12Z,15Z)/20:0)[iso6]	LMGL03014422
	TG(15:0/18:3(6Z,9Z,12Z)/19:0)[iso6]	LMGL03015187
	TG(15:0/18:3(9Z,12Z,15Z)/19:0)[iso6]	LMGL03015204
	TG(16:0/18:0/18:3(6Z,9Z,12Z))[iso6]	LMGL03015723
TG 18:3_34:1	TG(17:0/17:1(9Z)/18:3(9Z,12Z,15Z))[iso6]	LMGL03010127
	TG(16:0/18:1(9Z)/18:3(9Z,12Z,15Z))[iso6]	LMGL03010145
	TG(16:1(9Z)/18:0/18:3(9Z,12Z,15Z))[iso6]	LMGL03010146
	TG(12:0/18:3(6Z,9Z,12Z)/22:1(11Z))[iso6]	LMGL03013513
	TG(12:0/18:3(9Z,12Z,15Z)/22:1(11Z))[iso6]	LMGL03013530
	TG(14:0/18:3(6Z,9Z,12Z)/20:1(11Z))[iso6]	LMGL03014406
	TG(14:0/18:3(9Z,12Z,15Z)/20:1(11Z))[iso6]	LMGL03014423
	TG(14:1(9Z)/18:3(6Z,9Z,12Z)/20:0)[iso6]	LMGL03014811
	TG(14:1(9Z)/18:3(9Z,12Z,15Z)/20:0)[iso6]	LMGL03014828
	TG(15:0/18:3(6Z,9Z,12Z)/19:1(9Z))[iso6]	LMGL03015188
	TG(15:0/18:3(9Z,12Z,15Z)/19:1(9Z))[iso6]	LMGL03015205
	TG(15:1(9Z)/18:3(6Z,9Z,12Z)/19:0)[iso6]	LMGL03015538
	TG(15:1(9Z)/18:3(9Z,12Z,15Z)/19:0)[iso6]	LMGL03015555
	TG(16:0/18:1(9Z)/18:3(6Z,9Z,12Z))[iso6]	LMGL03015727
	TG(16:1(9Z)/18:0/18:3(6Z,9Z,12Z))[iso6]	LMGL03015814
	TG(17:0/17:1(9Z)/18:3(6Z,9Z,12Z))[iso6]	LMGL03015893

MxP® Quant 500 XL kit lipid annotation	Potential isomers	Data base ID
TG 18:3_34:2	TG(17:0/17:2(9Z,12Z)/18:3(9Z,12Z,15Z))[iso6]	LMGL03010147
	TG(17:1(9Z)/17:1(9Z)/18:3(9Z,12Z,15Z))[iso3]	LMGL03010148
	TG(16:0/18:2(9Z,12Z)/18:3(9Z,12Z,15Z))[iso6]	LMGL03010167
	TG(16:1(9Z)/18:1(9Z)/18:3(9Z,12Z,15Z))[iso6]	LMGL03010168
	TG(17:1(9Z)/17:1(9Z)/18:3(6Z,9Z,12Z))[iso3]	LMGL03013005
	TG(12:0/18:3(6Z,9Z,12Z)/22:2(13Z,16Z))[iso6]	LMGL03013514
	TG(12:0/18:3(9Z,12Z,15Z)/22:2(13Z,16Z))[iso6]	LMGL03013531
	TG(14:0/18:3(6Z,9Z,12Z)/20:2(11Z,14Z))[iso6]	LMGL03014407
	TG(14:0/18:3(9Z,12Z,15Z)/20:2(11Z,14Z))[iso6]	LMGL03014424
	TG(14:1(9Z)/18:3(6Z,9Z,12Z)/20:1(11Z))[iso6]	LMGL03014812
	TG(14:1(9Z)/18:3(9Z,12Z,15Z)/20:1(11Z))[iso6]	LMGL03014829
	TG(15:1(9Z)/18:3(6Z,9Z,12Z)/19:1(9Z))[iso6]	LMGL03015539
	TG(15:1(9Z)/18:3(9Z,12Z,15Z)/19:1(9Z))[iso6]	LMGL03015556
	TG(16:0/18:2(9Z,12Z)/18:3(6Z,9Z,12Z))[iso6]	LMGL03015731
	TG(16:1(9Z)/18:1(9Z)/18:3(6Z,9Z,12Z))[iso6]	LMGL03015818
	TG(17:0/17:2(9Z,12Z)/18:3(6Z,9Z,12Z))[iso6]	LMGL03015897
TG 18:3_34:3	TG(17:1(9Z)/17:2(9Z,12Z)/18:3(9Z,12Z,15Z))[iso6]	LMGL03010170
	TG(16:0/18:3(9Z,12Z,15Z)/18:3(9Z,12Z,15Z))[iso3]	LMGL03010192
	TG(16:1(9Z)/18:2(9Z,12Z)/18:3(9Z,12Z,15Z))[iso6]	LMGL03010193
	TG(16:0/18:3(6Z,9Z,12Z)/18:3(6Z,9Z,12Z))[iso3]	LMGL03012985
	TG(12:0/18:3(6Z,9Z,12Z)/22:3(10Z,13Z,16Z))[iso6]	LMGL03013515
	TG(12:0/18:3(9Z,12Z,15Z)/22:3(10Z,13Z,16Z))[iso6]	LMGL03013532
	TG(14:0/18:3(6Z,9Z,12Z)/20:3(8Z,11Z,14Z))[iso6]	LMGL03014408
	TG(14:0/18:3(9Z,12Z,15Z)/20:3(8Z,11Z,14Z))[iso6]	LMGL03014425
	TG(14:1(9Z)/18:3(6Z,9Z,12Z)/20:2(11Z,14Z))[iso6]	LMGL03014813
	TG(14:1(9Z)/18:3(9Z,12Z,15Z)/20:2(11Z,14Z))[iso6]	LMGL03014830
	TG(16:0/18:3(6Z,9Z,12Z)/18:3(9Z,12Z,15Z))[iso6]	LMGL03015735
	TG(16:1(9Z)/18:2(9Z,12Z)/18:3(6Z,9Z,12Z))[iso6]	LMGL03015822
	TG(17:1(9Z)/17:2(9Z,12Z)/18:3(6Z,9Z,12Z))[iso6]	LMGL03015980
TG 18:3_35:2	TG(17:0/18:2(9Z,12Z)/18:3(9Z,12Z,15Z))[iso6]	LMGL03010222
	TG(17:1(9Z)/18:1(9Z)/18:3(9Z,12Z,15Z))[iso6]	LMGL03010223
	TG(17:2(9Z,12Z)/18:0/18:3(9Z,12Z,15Z))[iso6]	LMGL03010224
	TG(13:0/18:3(6Z,9Z,12Z)/22:2(13Z,16Z))[iso6]	LMGL03013979
	TG(13:0/18:3(9Z,12Z,15Z)/22:2(13Z,16Z))[iso6]	LMGL03013996
	TG(15:0/18:3(6Z,9Z,12Z)/20:2(11Z,14Z))[iso6]	LMGL03015191
	TG(15:0/18:3(9Z,12Z,15Z)/20:2(11Z,14Z))[iso6]	LMGL03015208
	TG(15:1(9Z)/18:3(6Z,9Z,12Z)/20:1(11Z))[iso6]	LMGL03015541
	TG(15:1(9Z)/18:3(9Z,12Z,15Z)/20:1(11Z))[iso6]	LMGL03015558
	TG(16:1(9Z)/18:3(6Z,9Z,12Z)/19:1(9Z))[iso6]	LMGL03015829
	TG(16:1(9Z)/18:3(9Z,12Z,15Z)/19:1(9Z))[iso6]	LMGL03015845
	TG(17:0/18:2(9Z,12Z)/18:3(6Z,9Z,12Z))[iso6]	LMGL03015909
	TG(17:1(9Z)/18:1(9Z)/18:3(6Z,9Z,12Z))[iso6]	LMGL03015988
	TG(17:2(9Z,12Z)/18:0/18:3(6Z,9Z,12Z))[iso6]	LMGL03016063

MxP® Quant 500 XL kit lipid annotation	Potential isomers	Data base ID
TG 18:3_36:1	TG(18:0/18:1(9Z)/18:3(9Z,12Z,15Z))[iso6]	LMGL03010291
	TG(16:1(9Z)/18:3(9Z,12Z,15Z)/20:0)[iso6]	LMGL03010298
	TG(16:0/18:3(9Z,12Z,15Z)/20:1(11Z))[iso6]	LMGL03010302
	TG(17:1(9Z)/18:3(9Z,12Z,15Z)/19:0)[iso6]	LMGL03010332
	TG(14:0/18:3(6Z,9Z,12Z)/22:1(11Z))[iso6]	LMGL03014413
	TG(14:0/18:3(9Z,12Z,15Z)/22:1(11Z))[iso6]	LMGL03014430
	TG(14:1(9Z)/18:3(6Z,9Z,12Z)/22:0)[iso6]	LMGL03014818
	TG(14:1(9Z)/18:3(9Z,12Z,15Z)/22:0)[iso6]	LMGL03014835
	TG(15:1(9Z)/18:3(6Z,9Z,12Z)/21:0)[iso6]	LMGL03015546
	TG(15:1(9Z)/18:3(9Z,12Z,15Z)/21:0)[iso6]	LMGL03015563
	TG(16:0/18:3(6Z,9Z,12Z)/20:1(11Z))[iso6]	LMGL03015740
	TG(16:1(9Z)/18:3(6Z,9Z,12Z)/20:0)[iso6]	LMGL03015830
	TG(17:0/18:3(6Z,9Z,12Z)/19:1(9Z))[iso6]	LMGL03015916
	TG(17:0/18:3(9Z,12Z,15Z)/19:1(9Z))[iso6]	LMGL03015932
	TG(17:1(9Z)/18:3(6Z,9Z,12Z)/19:0)[iso6]	LMGL03015998
	TG(18:0/18:1(9Z)/18:3(6Z,9Z,12Z))[iso6]	LMGL03016142
TG 18:3_36:2	TG(18:0/18:2(9Z,12Z)/18:3(9Z,12Z,15Z))[iso6]	LMGL03010329
	TG(18:1(9Z)/18:1(9Z)/18:3(9Z,12Z,15Z))[iso3]	LMGL03010330
	TG(16:1(9Z)/18:3(9Z,12Z,15Z)/20:1(11Z))[iso6]	LMGL03010342
	TG(16:0/18:3(9Z,12Z,15Z)/20:2(11Z,14Z))[iso6]	LMGL03010346
	TG(17:2(9Z,12Z)/18:3(9Z,12Z,15Z)/19:0)[iso6]	LMGL03010375
	TG(18:1(9Z)/18:1(9Z)/18:3(6Z,9Z,12Z))[iso3]	LMGL03013029
	TG(14:0/18:3(6Z,9Z,12Z)/22:2(13Z,16Z))[iso6]	LMGL03014414
	TG(14:0/18:3(9Z,12Z,15Z)/22:2(13Z,16Z))[iso6]	LMGL03014431
	TG(14:1(9Z)/18:3(6Z,9Z,12Z)/22:1(11Z))[iso6]	LMGL03014819
	TG(14:1(9Z)/18:3(9Z,12Z,15Z)/22:1(11Z))[iso6]	LMGL03014836
	TG(16:0/18:3(6Z,9Z,12Z)/20:2(11Z,14Z))[iso6]	LMGL03015741
	TG(16:1(9Z)/18:3(6Z,9Z,12Z)/20:1(11Z))[iso6]	LMGL03015831
	TG(17:1(9Z)/18:3(6Z,9Z,12Z)/19:1(9Z))[iso6]	LMGL03015999
	TG(17:1(9Z)/18:3(9Z,12Z,15Z)/19:1(9Z))[iso6]	LMGL03016015
	TG(17:2(9Z,12Z)/18:3(6Z,9Z,12Z)/19:0)[iso6]	LMGL03016077
	TG(18:0/18:2(9Z,12Z)/18:3(6Z,9Z,12Z))[iso6]	LMGL03016146
TG 18:3_36:3	TG(18:0/18:3(9Z,12Z,15Z)/18:3(9Z,12Z,15Z))[iso3]	LMGL03010372
	TG(18:1(9Z)/18:2(9Z,12Z)/18:3(9Z,12Z,15Z))[iso6]	LMGL03010373
	TG(16:1(9Z)/18:3(9Z,12Z,15Z)/20:2(11Z,14Z))[iso6]	LMGL03010390
	TG(16:0/18:3(9Z,12Z,15Z)/20:3(8Z,11Z,14Z))[iso6]	LMGL03010394
	TG(18:0/18:3(6Z,9Z,12Z)/18:3(6Z,9Z,12Z))[iso3]	LMGL03013025
	TG(14:0/18:3(6Z,9Z,12Z)/22:3(10Z,13Z,16Z))[iso6]	LMGL03014415
	TG(14:0/18:3(9Z,12Z,15Z)/22:3(10Z,13Z,16Z))[iso6]	LMGL03014432
	TG(14:1(9Z)/18:3(6Z,9Z,12Z)/22:2(13Z,16Z))[iso6]	LMGL03014820
	TG(14:1(9Z)/18:3(9Z,12Z,15Z)/22:2(13Z,16Z))[iso6]	LMGL03014837
	TG(16:0/18:3(6Z,9Z,12Z)/20:3(8Z,11Z,14Z))[iso6]	LMGL03015742
	TG(16:1(9Z)/18:3(6Z,9Z,12Z)/20:2(11Z,14Z))[iso6]	LMGL03015832
	TG(17:2(9Z,12Z)/18:3(6Z,9Z,12Z)/19:1(9Z))[iso6]	LMGL03016078
	TG(17:2(9Z,12Z)/18:3(9Z,12Z,15Z)/19:1(9Z))[iso6]	LMGL03016094
	TG(18:0/18:3(6Z,9Z,12Z)/18:3(9Z,12Z,15Z))[iso6]	LMGL03016150
	TG(18:1(9Z)/18:2(9Z,12Z)/18:3(6Z,9Z,12Z))[iso6]	LMGL03016217

MxP® Quant 500 XL kit lipid annotation	Potential isomers	Data base ID
TG 18:3_36:4	TG(18:1(9Z)/18:3(9Z,12Z,15Z)/18:3(9Z,12Z,15Z))[iso3]	LMGL03010419
	TG(18:2(9Z,12Z)/18:2(9Z,12Z)/18:3(9Z,12Z,15Z))[iso3]	LMGL03010420
	TG(16:1(9Z)/18:3(9Z,12Z,15Z)/20:3(8Z,11Z,14Z))[iso6]	LMGL03010442
	TG(16:0/18:3(9Z,12Z,15Z)/20:4(5Z,8Z,11Z,14Z))[iso6]	LMGL03010446
	TG(18:1(9Z)/18:3(6Z,9Z,12Z)/18:3(6Z,9Z,12Z))[iso3]	LMGL03013033
	TG(18:2(9Z,12Z)/18:2(9Z,12Z)/18:3(6Z,9Z,12Z))[iso3]	LMGL03013037
	TG(14:0/18:3(6Z,9Z,12Z)/22:4(7Z,10Z,13Z,16Z))[iso6]	LMGL03014416
	TG(14:0/18:3(9Z,12Z,15Z)/22:4(7Z,10Z,13Z,16Z))[iso6]	LMGL03014433
	TG(14:1(9Z)/18:3(6Z,9Z,12Z)/22:3(10Z,13Z,16Z))[iso6]	LMGL03014821
	TG(14:1(9Z)/18:3(9Z,12Z,15Z)/22:3(10Z,13Z,16Z))[iso6]	LMGL03014838
	TG(16:0/18:3(6Z,9Z,12Z)/20:4(5Z,8Z,11Z,14Z))[iso6]	LMGL03015743
	TG(16:1(9Z)/18:3(6Z,9Z,12Z)/20:3(8Z,11Z,14Z))[iso6]	LMGL03015833
	TG(18:0/18:3(6Z,9Z,12Z)/18:4(6Z,9Z,12Z,15Z))[iso6]	LMGL03016151
	TG(18:0/18:3(9Z,12Z,15Z)/18:4(6Z,9Z,12Z,15Z))[iso6]	LMGL03016168
	TG(18:1(9Z)/18:3(6Z,9Z,12Z)/18:3(9Z,12Z,15Z))[iso6]	LMGL03016221
TG 18:3_38:5	TG(18:3(9Z,12Z,15Z)/18:3(9Z,12Z,15Z)/20:2(11Z,14Z))[iso3]	LMGL03010794
	TG(18:2(9Z,12Z)/18:3(9Z,12Z,15Z)/20:3(8Z,11Z,14Z))[iso6]	LMGL03010800
	TG(18:1(9Z)/18:3(9Z,12Z,15Z)/20:4(5Z,8Z,11Z,14Z))[iso6]	LMGL03010807
	TG(18:0/18:3(9Z,12Z,15Z)/20:5(5Z,8Z,11Z,14Z,17Z))[iso6]	LMGL03010814
	TG(16:1(9Z)/18:3(9Z,12Z,15Z)/22:4(7Z,10Z,13Z,16Z))[iso6]	LMGL03010990
	TG(16:0/18:3(9Z,12Z,15Z)/22:5(7Z,10Z,13Z,16Z,19Z))[iso6]	LMGL03010994
	TG(18:3(6Z,9Z,12Z)/18:3(6Z,9Z,12Z)/20:2(11Z,14Z))[iso3]	LMGL03013051
	TG(16:0/18:3(6Z,9Z,12Z)/22:5(7Z,10Z,13Z,16Z,19Z))[iso6]	LMGL03015751
	TG(16:1(9Z)/18:3(6Z,9Z,12Z)/22:4(7Z,10Z,13Z,16Z))[iso6]	LMGL03015841
	TG(18:0/18:3(6Z,9Z,12Z)/20:5(5Z,8Z,11Z,14Z,17Z))[iso6]	LMGL03016159
	TG(18:1(9Z)/18:3(6Z,9Z,12Z)/20:4(5Z,8Z,11Z,14Z))[iso6]	LMGL03016229
	TG(18:2(9Z,12Z)/18:3(6Z,9Z,12Z)/20:3(8Z,11Z,14Z))[iso6]	LMGL03016295
	TG(18:3(6Z,9Z,12Z)/18:3(9Z,12Z,15Z)/20:2(11Z,14Z))[iso6]	LMGL03016360
	TG(18:3(6Z,9Z,12Z)/18:4(6Z,9Z,12Z,15Z)/20:1(11Z))[iso6]	LMGL03016375
	TG(18:3(9Z,12Z,15Z)/18:4(6Z,9Z,12Z,15Z)/20:1(11Z))[iso6]	LMGL03016511
TG 18:3_38:6	TG(18:3(9Z,12Z,15Z)/18:3(9Z,12Z,15Z)/20:3(8Z,11Z,14Z))[iso3]	LMGL03010869
	TG(18:2(9Z,12Z)/18:3(9Z,12Z,15Z)/20:4(5Z,8Z,11Z,14Z))[iso6]	LMGL03010876
	TG(18:1(9Z)/18:3(9Z,12Z,15Z)/20:5(5Z,8Z,11Z,14Z,17Z))[iso6]	LMGL03010883
	TG(16:1(9Z)/18:3(9Z,12Z,15Z)/22:5(7Z,10Z,13Z,16Z,19Z))[iso6]	LMGL03011070
	TG(16:0/18:3(9Z,12Z,15Z)/22:6(4Z,7Z,10Z,13Z,16Z,19Z))[iso6]	LMGL03011074
	TG(18:3(6Z,9Z,12Z)/18:3(6Z,9Z,12Z)/20:3(8Z,11Z,14Z))[iso3]	LMGL03013052
	TG(16:0/18:3(6Z,9Z,12Z)/22:6(4Z,7Z,10Z,13Z,16Z,19Z))[iso6]	LMGL03015752
	TG(16:1(9Z)/18:3(6Z,9Z,12Z)/22:5(7Z,10Z,13Z,16Z,19Z))[iso6]	LMGL03015842
	TG(18:1(9Z)/18:3(6Z,9Z,12Z)/20:5(5Z,8Z,11Z,14Z,17Z))[iso6]	LMGL03016230
	TG(18:2(9Z,12Z)/18:3(6Z,9Z,12Z)/20:4(5Z,8Z,11Z,14Z))[iso6]	LMGL03016296
	TG(18:3(6Z,9Z,12Z)/18:3(9Z,12Z,15Z)/20:3(8Z,11Z,14Z))[iso6]	LMGL03016361
	TG(18:3(6Z,9Z,12Z)/18:4(6Z,9Z,12Z,15Z)/20:2(11Z,14Z))[iso6]	LMGL03016376
	TG(18:3(9Z,12Z,15Z)/18:4(6Z,9Z,12Z,15Z)/20:2(11Z,14Z))[iso6]	LMGL03016512
TG 20:0_32:3	TG(12:0/20:0/20:3(8Z,11Z,14Z))[iso6]	LMGL03013583
	TG(14:0/18:3(6Z,9Z,12Z)/20:0)[iso6]	LMGL03014405
	TG(14:0/18:3(9Z,12Z,15Z)/20:0)[iso6]	LMGL03014422
	TG(14:1(9Z)/18:2(9Z,12Z)/20:0)[iso6]	LMGL03014793
	TG(15:1(9Z)/17:2(9Z,12Z)/20:0)[iso6]	LMGL03015462
TG 20:0_32:4	TG(12:0/20:0/20:4(5Z,8Z,11Z,14Z))[iso6]	LMGL03013584
	TG(14:0/18:4(6Z,9Z,12Z,15Z)/20:0)[iso6]	LMGL03014438
	TG(14:1(9Z)/18:3(6Z,9Z,12Z)/20:0)[iso6]	LMGL03014811
	TG(14:1(9Z)/18:3(9Z,12Z,15Z)/20:0)[iso6]	LMGL03014828

MxP® Quant 500 XL kit lipid annotation	Potential isomers	Data base ID
TG 20:0_34:1	TG(17:0/17:1(9Z)/20:0)[iso6]	LMGL03010177
	TG(16:0/18:1(9Z)/20:0)[iso6]	LMGL03010201
	TG(16:1(9Z)/18:0/20:0)[iso6]	LMGL03010202
	TG(14:1(9Z)/20:0/20:0)[iso3]	LMGL03012857
	TG(12:0/20:0/22:1(11Z))[iso6]	LMGL03013588
	TG(14:0/20:0/20:1(11Z))[iso6]	LMGL03014481
	TG(15:0/19:1(9Z)/20:0)[iso6]	LMGL03015251
	TG(15:1(9Z)/19:0/20:0)[iso6]	LMGL03015588
TG 20:1_24:3	PA(19:1(9Z)/20:1(11Z))	LMGP10010498
	PA(20:1(11Z)/19:1(9Z))	LMGP10010544
	PA(P-20:0/20:1(11Z))	LMGP10030076
TG 20:1_26:1	TG(12:0/14:1(9Z)/20:1(11Z))[iso6]	LMGL03013254
TG 20:1_30:1	TG(12:0/18:1(9Z)/20:1(11Z))[iso6]	LMGL03013469
	TG(13:0/17:1(9Z)/20:1(11Z))[iso6]	LMGL03013871
	TG(14:0/16:1(9Z)/20:1(11Z))[iso6]	LMGL03014259
	TG(14:1(9Z)/16:0/20:1(11Z))[iso6]	LMGL03014640
	TG(15:0/15:1(9Z)/20:1(11Z))[iso6]	LMGL03014992
TG 20:1_31:0	TG(12:0/19:0/20:1(11Z))[iso6]	LMGL03013554
	TG(13:0/18:0/20:1(11Z))[iso6]	LMGL03013914
	TG(14:0/17:0/20:1(11Z))[iso6]	LMGL03014283
	TG(15:0/16:0/20:1(11Z))[iso6]	LMGL03015018
TG 20:1_32:0	TG(16:0/16:0/20:1(11Z))[iso3]	LMGL03010096
	TG(12:0/20:0/20:1(11Z))[iso6]	LMGL03013581
	TG(13:0/19:0/20:1(11Z))[iso6]	LMGL03014019
	TG(14:0/18:0/20:1(11Z))[iso6]	LMGL03014349
	TG(15:0/17:0/20:1(11Z))[iso6]	LMGL03015067
TG 20:1_32:1	TG(16:0/16:1(9Z)/20:1(11Z))[iso6]	LMGL03010114
	TG(12:0/20:1(11Z)/20:1(11Z))[iso3]	LMGL03012678
	TG(13:0/19:1(9Z)/20:1(11Z))[iso6]	LMGL03014033
	TG(14:0/18:1(9Z)/20:1(11Z))[iso6]	LMGL03014369
	TG(14:1(9Z)/18:0/20:1(11Z))[iso6]	LMGL03014755
	TG(15:0/17:1(9Z)/20:1(11Z))[iso6]	LMGL03015090
	TG(15:1(9Z)/17:0/20:1(11Z))[iso6]	LMGL03015418
TG 20:1_32:2	TG(16:1(9Z)/16:1(9Z)/20:1(11Z))[iso3]	LMGL03010134
	TG(12:0/20:1(11Z)/20:2(11Z,14Z))[iso6]	LMGL03013594
	TG(14:0/18:2(9Z,12Z)/20:1(11Z))[iso6]	LMGL03014388
	TG(14:1(9Z)/18:1(9Z)/20:1(11Z))[iso6]	LMGL03014775
	TG(15:0/17:2(9Z,12Z)/20:1(11Z))[iso6]	LMGL03015112
	TG(15:1(9Z)/17:1(9Z)/20:1(11Z))[iso6]	LMGL03015441
TG 20:1_32:3	TG(12:0/20:1(11Z)/20:3(8Z,11Z,14Z))[iso6]	LMGL03013595
	TG(14:0/18:3(6Z,9Z,12Z)/20:1(11Z))[iso6]	LMGL03014406
	TG(14:0/18:3(9Z,12Z,15Z)/20:1(11Z))[iso6]	LMGL03014423
	TG(14:1(9Z)/18:2(9Z,12Z)/20:1(11Z))[iso6]	LMGL03014794
	TG(15:1(9Z)/17:2(9Z,12Z)/20:1(11Z))[iso6]	LMGL03015463
TG 20:1_34:0	TG(17:0/17:0/20:1(11Z))[iso3]	LMGL03010180
	TG(16:0/18:0/20:1(11Z))[iso6]	LMGL03010205
	TG(12:0/20:1(11Z)/22:0)[iso6]	LMGL03013599
	TG(13:0/20:1(11Z)/21:0)[iso6]	LMGL03014063
	TG(14:0/20:0/20:1(11Z))[iso6]	LMGL03014481
	TG(15:0/19:0/20:1(11Z))[iso6]	LMGL03015238

MxP® Quant 500 XL kit lipid annotation	Potential isomers	Data base ID
TG 20:1_34:1	TG(17:0/17:1(9Z)/20:1(11Z))[iso6]	LMGL03010207
	TG(16:0/18:1(9Z)/20:1(11Z))[iso6]	LMGL03010234
	TG(16:1(9Z)/18:0/20:1(11Z))[iso6]	LMGL03010235
	TG(14:0/20:1(11Z)/20:1(11Z))[iso3]	LMGL03012800
	TG(12:0/20:1(11Z)/22:1(11Z))[iso6]	LMGL03013600
	TG(14:1(9Z)/20:0/20:1(11Z))[iso6]	LMGL03014887
	TG(15:0/19:1(9Z)/20:1(11Z))[iso6]	LMGL03015252
	TG(15:1(9Z)/19:0/20:1(11Z))[iso6]	LMGL03015589
TG 20:1_34:2	TG(17:0/17:2(9Z,12Z)/20:1(11Z))[iso6]	LMGL03010236
	TG(17:1(9Z)/17:1(9Z)/20:1(11Z))[iso3]	LMGL03010237
	TG(16:0/18:2(9Z,12Z)/20:1(11Z))[iso6]	LMGL03010267
	TG(16:1(9Z)/18:1(9Z)/20:1(11Z))[iso6]	LMGL03010268
	TG(14:1(9Z)/20:1(11Z)/20:1(11Z))[iso3]	LMGL03012858
	TG(12:0/20:1(11Z)/22:2(13Z,16Z))[iso6]	LMGL03013601
	TG(14:0/20:1(11Z)/20:2(11Z,14Z))[iso6]	LMGL03014494
	TG(15:1(9Z)/19:1(9Z)/20:1(11Z))[iso6]	LMGL03015603
TG 20:1_34:3	TG(17:1(9Z)/17:2(9Z,12Z)/20:1(11Z))[iso6]	LMGL03010270
	TG(16:0/18:3(9Z,12Z,15Z)/20:1(11Z))[iso6]	LMGL03010302
	TG(16:1(9Z)/18:2(9Z,12Z)/20:1(11Z))[iso6]	LMGL03010303
	TG(12:0/20:1(11Z)/22:3(10Z,13Z,16Z))[iso6]	LMGL03013602
	TG(14:0/20:1(11Z)/20:3(8Z,11Z,14Z))[iso6]	LMGL03014495
	TG(14:1(9Z)/20:1(11Z)/20:2(11Z,14Z))[iso6]	LMGL03014900
	TG(16:0/18:3(6Z,9Z,12Z)/20:1(11Z))[iso6]	LMGL03015740
TG 20:2_32:0	TG(16:0/16:0/20:2(11Z,14Z))[iso3]	LMGL03010115
	TG(12:0/20:0/20:2(11Z,14Z))[iso6]	LMGL03013582
	TG(13:0/19:0/20:2(11Z,14Z))[iso6]	LMGL03014020
	TG(14:0/18:0/20:2(11Z,14Z))[iso6]	LMGL03014350
	TG(15:0/17:0/20:2(11Z,14Z))[iso6]	LMGL03015068
TG 20:2_32:1	TG(16:0/16:1(9Z)/20:2(11Z,14Z))[iso6]	LMGL03010135
	TG(12:0/20:1(11Z)/20:2(11Z,14Z))[iso6]	LMGL03013594
	TG(13:0/19:1(9Z)/20:2(11Z,14Z))[iso6]	LMGL03014034
	TG(14:0/18:1(9Z)/20:2(11Z,14Z))[iso6]	LMGL03014370
	TG(14:1(9Z)/18:0/20:2(11Z,14Z))[iso6]	LMGL03014756
	TG(15:0/17:1(9Z)/20:2(11Z,14Z))[iso6]	LMGL03015091
	TG(15:1(9Z)/17:0/20:2(11Z,14Z))[iso6]	LMGL03015419
TG 20:2_34:1	TG(17:0/17:1(9Z)/20:2(11Z,14Z))[iso6]	LMGL03010240
	TG(16:0/18:1(9Z)/20:2(11Z,14Z))[iso6]	LMGL03010271
	TG(16:1(9Z)/18:0/20:2(11Z,14Z))[iso6]	LMGL03010272
	TG(12:0/20:2(11Z,14Z)/22:1(11Z))[iso6]	LMGL03013611
	TG(14:0/20:1(11Z)/20:2(11Z,14Z))[iso6]	LMGL03014494
	TG(14:1(9Z)/20:0/20:2(11Z,14Z))[iso6]	LMGL03014888
	TG(15:0/19:1(9Z)/20:2(11Z,14Z))[iso6]	LMGL03015253
	TG(15:1(9Z)/19:0/20:2(11Z,14Z))[iso6]	LMGL03015590
TG 20:2_34:2	TG(17:0/17:2(9Z,12Z)/20:2(11Z,14Z))[iso6]	LMGL03010273
	TG(17:1(9Z)/17:1(9Z)/20:2(11Z,14Z))[iso3]	LMGL03010274
	TG(16:0/18:2(9Z,12Z)/20:2(11Z,14Z))[iso6]	LMGL03010307
	TG(16:1(9Z)/18:1(9Z)/20:2(11Z,14Z))[iso6]	LMGL03010308
	TG(14:0/20:2(11Z,14Z)/20:2(11Z,14Z))[iso3]	LMGL03012801
	TG(12:0/20:2(11Z,14Z)/22:2(13Z,16Z))[iso6]	LMGL03013612
	TG(14:1(9Z)/20:1(11Z)/20:2(11Z,14Z))[iso6]	LMGL03014900
	TG(15:1(9Z)/19:1(9Z)/20:2(11Z,14Z))[iso6]	LMGL03015604

MxP® Quant 500 XL kit lipid annotation	Potential isomers	Data base ID
TG 20:2_34:3	TG(17:1(9Z)/17:2(9Z,12Z)/20:2(11Z,14Z))[iso6]	LMGL03010310
	TG(16:0/18:3(9Z,12Z,15Z)/20:2(11Z,14Z))[iso6]	LMGL03010346
	TG(16:1(9Z)/18:2(9Z,12Z)/20:2(11Z,14Z))[iso6]	LMGL03010347
	TG(14:1(9Z)/20:2(11Z,14Z)/20:2(11Z,14Z))[iso3]	LMGL03012859
	TG(12:0/20:2(11Z,14Z)/22:3(10Z,13Z,16Z))[iso6]	LMGL03013613
	TG(14:0/20:2(11Z,14Z)/20:3(8Z,11Z,14Z))[iso6]	LMGL03014506
	TG(16:0/18:3(6Z,9Z,12Z)/20:2(11Z,14Z))[iso6]	LMGL03015741
TG 20:2_34:4	TG(17:2(9Z,12Z)/17:2(9Z,12Z)/20:2(11Z,14Z))[iso3]	LMGL03010350
	TG(16:1(9Z)/18:3(9Z,12Z,15Z)/20:2(11Z,14Z))[iso6]	LMGL03010390
	TG(12:0/20:2(11Z,14Z)/22:4(7Z,10Z,13Z,16Z))[iso6]	LMGL03013614
	TG(14:0/20:2(11Z,14Z)/20:4(5Z,8Z,11Z,14Z))[iso6]	LMGL03014507
	TG(14:1(9Z)/20:2(11Z,14Z)/20:3(8Z,11Z,14Z))[iso6]	LMGL03014912
	TG(16:0/18:4(6Z,9Z,12Z,15Z)/20:2(11Z,14Z))[iso6]	LMGL03015760
	TG(16:1(9Z)/18:3(6Z,9Z,12Z)/20:2(11Z,14Z))[iso6]	LMGL03015832
TG 20:2_36:5	TG(18:2(9Z,12Z)/18:3(9Z,12Z,15Z)/20:2(11Z,14Z))[iso6]	LMGL03010727
	TG(16:1(9Z)/20:2(11Z,14Z)/20:4(5Z,8Z,11Z,14Z))[iso6]	LMGL03010736
	TG(16:0/20:2(11Z,14Z)/20:5(5Z,8Z,11Z,14Z,17Z))[iso6]	LMGL03010742
	TG(14:0/20:2(11Z,14Z)/22:5(7Z,10Z,13Z,16Z,19Z))[iso6]	LMGL03014515
	TG(14:1(9Z)/20:2(11Z,14Z)/22:4(7Z,10Z,13Z,16Z))[iso6]	LMGL03014920
	TG(18:1(9Z)/18:4(6Z,9Z,12Z,15Z)/20:2(11Z,14Z))[iso6]	LMGL03016246
	TG(18:2(9Z,12Z)/18:3(6Z,9Z,12Z)/20:2(11Z,14Z))[iso6]	LMGL03016294
TG 20:3_32:0	TG(16:0/16:0/20:3(8Z,11Z,14Z))[iso3]	LMGL03010136
	TG(12:0/20:0/20:3(8Z,11Z,14Z))[iso6]	LMGL03013583
	TG(13:0/19:0/20:3(8Z,11Z,14Z))[iso6]	LMGL03014021
	TG(14:0/18:0/20:3(8Z,11Z,14Z))[iso6]	LMGL03014351
	TG(15:0/17:0/20:3(8Z,11Z,14Z))[iso6]	LMGL03015069
TG 20:3_32:1	TG(16:0/16:1(9Z)/20:3(8Z,11Z,14Z))[iso6]	LMGL03010159
	TG(12:0/20:1(11Z)/20:3(8Z,11Z,14Z))[iso6]	LMGL03013595
	TG(13:0/19:1(9Z)/20:3(8Z,11Z,14Z))[iso6]	LMGL03014035
	TG(14:0/18:1(9Z)/20:3(8Z,11Z,14Z))[iso6]	LMGL03014371
	TG(14:1(9Z)/18:0/20:3(8Z,11Z,14Z))[iso6]	LMGL03014757
	TG(15:0/17:1(9Z)/20:3(8Z,11Z,14Z))[iso6]	LMGL03015092
	TG(15:1(9Z)/17:0/20:3(8Z,11Z,14Z))[iso6]	LMGL03015420
TG 20:3_32:2	TG(16:1(9Z)/16:1(9Z)/20:3(8Z,11Z,14Z))[iso3]	LMGL03010184
	TG(12:0/20:2(11Z,14Z)/20:3(8Z,11Z,14Z))[iso6]	LMGL03013606
	TG(14:0/18:2(9Z,12Z)/20:3(8Z,11Z,14Z))[iso6]	LMGL03014390
	TG(14:1(9Z)/18:1(9Z)/20:3(8Z,11Z,14Z))[iso6]	LMGL03014777
	TG(15:0/17:2(9Z,12Z)/20:3(8Z,11Z,14Z))[iso6]	LMGL03015114
	TG(15:1(9Z)/17:1(9Z)/20:3(8Z,11Z,14Z))[iso6]	LMGL03015443
TG 20:3_34:0	TG(17:0/17:0/20:3(8Z,11Z,14Z))[iso3]	LMGL03010243
	TG(16:0/18:0/20:3(8Z,11Z,14Z))[iso6]	LMGL03010275
	TG(12:0/20:3(8Z,11Z,14Z)/22:0)[iso6]	LMGL03013620
	TG(13:0/20:3(8Z,11Z,14Z)/21:0)[iso6]	LMGL03014084
	TG(14:0/20:0/20:3(8Z,11Z,14Z))[iso6]	LMGL03014483
	TG(15:0/19:0/20:3(8Z,11Z,14Z))[iso6]	LMGL03015240
TG 20:3_34:1	TG(17:0/17:1(9Z)/20:3(8Z,11Z,14Z))[iso6]	LMGL03010277
	TG(16:0/18:1(9Z)/20:3(8Z,11Z,14Z))[iso6]	LMGL03010311
	TG(16:1(9Z)/18:0/20:3(8Z,11Z,14Z))[iso6]	LMGL03010312
	TG(12:0/20:3(8Z,11Z,14Z)/22:1(11Z))[iso6]	LMGL03013621
	TG(14:0/20:1(11Z)/20:3(8Z,11Z,14Z))[iso6]	LMGL03014495
	TG(14:1(9Z)/20:0/20:3(8Z,11Z,14Z))[iso6]	LMGL03014889
	TG(15:0/19:1(9Z)/20:3(8Z,11Z,14Z))[iso6]	LMGL03015254
	TG(15:1(9Z)/19:0/20:3(8Z,11Z,14Z))[iso6]	LMGL03015591

MxP® Quant 500 XL kit lipid annotation	Potential isomers	Data base ID
TG 20:3_34:2	TG(17:0/17:2(9Z,12Z)/20:3(8Z,11Z,14Z))[iso6]	LMGL03010313
	TG(17:1(9Z)/17:1(9Z)/20:3(8Z,11Z,14Z))[iso3]	LMGL03010314
	TG(16:0/18:2(9Z,12Z)/20:3(8Z,11Z,14Z))[iso6]	LMGL03010351
	TG(16:1(9Z)/18:1(9Z)/20:3(8Z,11Z,14Z))[iso6]	LMGL03010352
	TG(12:0/20:3(8Z,11Z,14Z)/22:2(13Z,16Z))[iso6]	LMGL03013622
	TG(14:0/20:2(11Z,14Z)/20:3(8Z,11Z,14Z))[iso6]	LMGL03014506
	TG(14:1(9Z)/20:1(11Z)/20:3(8Z,11Z,14Z))[iso6]	LMGL03014901
	TG(15:1(9Z)/19:1(9Z)/20:3(8Z,11Z,14Z))[iso6]	LMGL03015605
TG 20:3_34:3	TG(17:1(9Z)/17:2(9Z,12Z)/20:3(8Z,11Z,14Z))[iso6]	LMGL03010354
	TG(16:0/18:3(9Z,12Z,15Z)/20:3(8Z,11Z,14Z))[iso6]	LMGL03010394
	TG(16:1(9Z)/18:2(9Z,12Z)/20:3(8Z,11Z,14Z))[iso6]	LMGL03010395
	TG(14:0/20:3(8Z,11Z,14Z)/20:3(8Z,11Z,14Z))[iso3]	LMGL03012802
	TG(12:0/20:3(8Z,11Z,14Z)/22:3(10Z,13Z,16Z))[iso6]	LMGL03013623
	TG(14:1(9Z)/20:2(11Z,14Z)/20:3(8Z,11Z,14Z))[iso6]	LMGL03014912
	TG(16:0/18:3(6Z,9Z,12Z)/20:3(8Z,11Z,14Z))[iso6]	LMGL03015742
TG 20:3_36:3	TG(16:0/20:3(8Z,11Z,14Z)/20:3(8Z,11Z,14Z))[iso3]	LMGL03010664
	TG(16:1(9Z)/20:2(11Z,14Z)/20:3(8Z,11Z,14Z))[iso6]	LMGL03010665
	TG(18:0/18:3(9Z,12Z,15Z)/20:3(8Z,11Z,14Z))[iso6]	LMGL03010669
	TG(18:1(9Z)/18:2(9Z,12Z)/20:3(8Z,11Z,14Z))[iso6]	LMGL03010670
	TG(14:0/20:3(8Z,11Z,14Z)/22:3(10Z,13Z,16Z))[iso6]	LMGL03014523
	TG(14:1(9Z)/20:3(8Z,11Z,14Z)/22:2(13Z,16Z))[iso6]	LMGL03014928
	TG(17:2(9Z,12Z)/19:1(9Z)/20:3(8Z,11Z,14Z))[iso6]	LMGL03016117
	TG(18:0/18:3(6Z,9Z,12Z)/20:3(8Z,11Z,14Z))[iso6]	LMGL03016157
TG 20:3_36:4	TG(16:1(9Z)/20:3(8Z,11Z,14Z)/20:3(8Z,11Z,14Z))[iso3]	LMGL03010728
	TG(18:1(9Z)/18:3(9Z,12Z,15Z)/20:3(8Z,11Z,14Z))[iso6]	LMGL03010733
	TG(18:2(9Z,12Z)/18:2(9Z,12Z)/20:3(8Z,11Z,14Z))[iso3]	LMGL03010734
	TG(16:0/20:3(8Z,11Z,14Z)/20:4(5Z,8Z,11Z,14Z))[iso6]	LMGL03010735
	TG(14:0/20:3(8Z,11Z,14Z)/22:4(7Z,10Z,13Z,16Z))[iso6]	LMGL03014524
	TG(14:1(9Z)/20:3(8Z,11Z,14Z)/22:3(10Z,13Z,16Z))[iso6]	LMGL03014929
	TG(18:0/18:4(6Z,9Z,12Z,15Z)/20:3(8Z,11Z,14Z))[iso6]	LMGL03016176
	TG(18:1(9Z)/18:3(6Z,9Z,12Z)/20:3(8Z,11Z,14Z))[iso6]	LMGL03016228
TG 20:3_36:5	TG(18:2(9Z,12Z)/18:3(9Z,12Z,15Z)/20:3(8Z,11Z,14Z))[iso6]	LMGL03010800
	TG(16:1(9Z)/20:3(8Z,11Z,14Z)/20:4(5Z,8Z,11Z,14Z))[iso6]	LMGL03010802
	TG(16:0/20:3(8Z,11Z,14Z)/20:5(5Z,8Z,11Z,14Z,17Z))[iso6]	LMGL03010809
	TG(14:0/20:3(8Z,11Z,14Z)/22:5(7Z,10Z,13Z,16Z,19Z))[iso6]	LMGL03014525
	TG(14:1(9Z)/20:3(8Z,11Z,14Z)/22:4(7Z,10Z,13Z,16Z))[iso6]	LMGL03014930
	TG(18:1(9Z)/18:4(6Z,9Z,12Z,15Z)/20:3(8Z,11Z,14Z))[iso6]	LMGL03016247
	TG(18:2(9Z,12Z)/18:3(6Z,9Z,12Z)/20:3(8Z,11Z,14Z))[iso6]	LMGL03016295
TG 20:4_30:0	TG(15:0/15:0/20:4(5Z,8Z,11Z,14Z))[iso3]	LMGL03012889
	TG(12:0/18:0/20:4(5Z,8Z,11Z,14Z))[iso6]	LMGL03013452
	TG(13:0/17:0/20:4(5Z,8Z,11Z,14Z))[iso6]	LMGL03013851
	TG(14:0/16:0/20:4(5Z,8Z,11Z,14Z))[iso6]	LMGL03014237
TG 20:4_32:0	TG(16:0/16:0/20:4(5Z,8Z,11Z,14Z))[iso3]	LMGL03010160
	TG(12:0/20:0/20:4(5Z,8Z,11Z,14Z))[iso6]	LMGL03013584
	TG(13:0/19:0/20:4(5Z,8Z,11Z,14Z))[iso6]	LMGL03014022
	TG(14:0/18:0/20:4(5Z,8Z,11Z,14Z))[iso6]	LMGL03014352
	TG(15:0/17:0/20:4(5Z,8Z,11Z,14Z))[iso6]	LMGL03015070
TG 20:4_32:1	TG(16:0/16:1(9Z)/20:4(5Z,8Z,11Z,14Z))[iso6]	LMGL03010185
	TG(12:0/20:1(11Z)/20:4(5Z,8Z,11Z,14Z))[iso6]	LMGL03013596
	TG(13:0/19:1(9Z)/20:4(5Z,8Z,11Z,14Z))[iso6]	LMGL03014036
	TG(14:0/18:1(9Z)/20:4(5Z,8Z,11Z,14Z))[iso6]	LMGL03014372
	TG(14:1(9Z)/18:0/20:4(5Z,8Z,11Z,14Z))[iso6]	LMGL03014758
	TG(15:0/17:1(9Z)/20:4(5Z,8Z,11Z,14Z))[iso6]	LMGL03015093
	TG(15:1(9Z)/17:0/20:4(5Z,8Z,11Z,14Z))[iso6]	LMGL03015421

MxP® Quant 500 XL kit lipid annotation	Potential isomers	Data base ID
TG 20:4_32:2	TG(16:1(9Z)/16:1(9Z)/20:4(5Z,8Z,11Z,14Z))[iso3]	LMGL03010214
	TG(12:0/20:2(11Z,14Z)/20:4(5Z,8Z,11Z,14Z))[iso6]	LMGL03013607
	TG(14:0/18:2(9Z,12Z)/20:4(5Z,8Z,11Z,14Z))[iso6]	LMGL03014391
	TG(14:1(9Z)/18:1(9Z)/20:4(5Z,8Z,11Z,14Z))[iso6]	LMGL03014778
	TG(15:0/17:2(9Z,12Z)/20:4(5Z,8Z,11Z,14Z))[iso6]	LMGL03015115
	TG(15:1(9Z)/17:1(9Z)/20:4(5Z,8Z,11Z,14Z))[iso6]	LMGL03015444
TG 20:4_33:2	TG(16:0/17:2(9Z,12Z)/20:4(5Z,8Z,11Z,14Z))[iso6]	LMGL03010278
	TG(16:1(9Z)/17:1(9Z)/20:4(5Z,8Z,11Z,14Z))[iso6]	LMGL03010279
	TG(13:0/20:2(11Z,14Z)/20:4(5Z,8Z,11Z,14Z))[iso6]	LMGL03014072
	TG(14:1(9Z)/19:1(9Z)/20:4(5Z,8Z,11Z,14Z))[iso6]	LMGL03014877
	TG(15:0/18:2(9Z,12Z)/20:4(5Z,8Z,11Z,14Z))[iso6]	LMGL03015175
	TG(15:1(9Z)/18:1(9Z)/20:4(5Z,8Z,11Z,14Z))[iso6]	LMGL03015507
TG 20:4_34:0	TG(17:0/17:0/20:4(5Z,8Z,11Z,14Z))[iso3]	LMGL03010280
	TG(16:0/18:0/20:4(5Z,8Z,11Z,14Z))[iso6]	LMGL03010315
	TG(12:0/20:4(5Z,8Z,11Z,14Z)/22:0)[iso6]	LMGL03013629
	TG(13:0/20:4(5Z,8Z,11Z,14Z)/21:0)[iso6]	LMGL03014093
	TG(14:0/20:0/20:4(5Z,8Z,11Z,14Z))[iso6]	LMGL03014484
	TG(15:0/19:0/20:4(5Z,8Z,11Z,14Z))[iso6]	LMGL03015241
TG 20:4_34:1	TG(17:0/17:1(9Z)/20:4(5Z,8Z,11Z,14Z))[iso6]	LMGL03010317
	TG(16:0/18:1(9Z)/20:4(5Z,8Z,11Z,14Z))[iso6]	LMGL03010355
	TG(16:1(9Z)/18:0/20:4(5Z,8Z,11Z,14Z))[iso6]	LMGL03010356
	TG(12:0/20:4(5Z,8Z,11Z,14Z)/22:1(11Z))[iso6]	LMGL03013630
	TG(14:0/20:1(11Z)/20:4(5Z,8Z,11Z,14Z))[iso6]	LMGL03014496
	TG(14:1(9Z)/20:0/20:4(5Z,8Z,11Z,14Z))[iso6]	LMGL03014890
	TG(15:0/19:1(9Z)/20:4(5Z,8Z,11Z,14Z))[iso6]	LMGL03015255
	TG(15:1(9Z)/19:0/20:4(5Z,8Z,11Z,14Z))[iso6]	LMGL03015592
TG 20:4_34:2	TG(17:0/17:2(9Z,12Z)/20:4(5Z,8Z,11Z,14Z))[iso6]	LMGL03010357
	TG(17:1(9Z)/17:1(9Z)/20:4(5Z,8Z,11Z,14Z))[iso3]	LMGL03010358
	TG(16:0/18:2(9Z,12Z)/20:4(5Z,8Z,11Z,14Z))[iso6]	LMGL03010399
	TG(16:1(9Z)/18:1(9Z)/20:4(5Z,8Z,11Z,14Z))[iso6]	LMGL03010400
	TG(12:0/20:4(5Z,8Z,11Z,14Z)/22:2(13Z,16Z))[iso6]	LMGL03013631
	TG(14:0/20:2(11Z,14Z)/20:4(5Z,8Z,11Z,14Z))[iso6]	LMGL03014507
	TG(14:1(9Z)/20:1(11Z)/20:4(5Z,8Z,11Z,14Z))[iso6]	LMGL03014902
TG 20:4_34:2	TG(15:1(9Z)/19:1(9Z)/20:4(5Z,8Z,11Z,14Z))[iso6]	LMGL03015606
	TG(17:1(9Z)/17:2(9Z,12Z)/20:4(5Z,8Z,11Z,14Z))[iso6]	LMGL03010402
	TG(16:0/18:3(9Z,12Z,15Z)/20:4(5Z,8Z,11Z,14Z))[iso6]	LMGL03010446
	TG(16:1(9Z)/18:2(9Z,12Z)/20:4(5Z,8Z,11Z,14Z))[iso6]	LMGL03010447
	TG(12:0/20:4(5Z,8Z,11Z,14Z)/22:3(10Z,13Z,16Z))[iso6]	LMGL03013632
	TG(14:0/20:3(8Z,11Z,14Z)/20:4(5Z,8Z,11Z,14Z))[iso6]	LMGL03014517
	TG(14:1(9Z)/20:2(11Z,14Z)/20:4(5Z,8Z,11Z,14Z))[iso6]	LMGL03014913
	TG(16:0/18:3(6Z,9Z,12Z)/20:4(5Z,8Z,11Z,14Z))[iso6]	LMGL03015743
TG 20:4_35:3	TG(17:0/18:3(9Z,12Z,15Z)/20:4(5Z,8Z,11Z,14Z))[iso6]	LMGL03010554
	TG(17:1(9Z)/18:2(9Z,12Z)/20:4(5Z,8Z,11Z,14Z))[iso6]	LMGL03010555
	TG(17:2(9Z,12Z)/18:1(9Z)/20:4(5Z,8Z,11Z,14Z))[iso6]	LMGL03010556
	TG(13:0/20:4(5Z,8Z,11Z,14Z)/22:3(10Z,13Z,16Z))[iso6]	LMGL03014097
	TG(15:0/20:3(8Z,11Z,14Z)/20:4(5Z,8Z,11Z,14Z))[iso6]	LMGL03015301
	TG(15:1(9Z)/20:2(11Z,14Z)/20:4(5Z,8Z,11Z,14Z))[iso6]	LMGL03015642
	TG(17:0/18:3(6Z,9Z,12Z)/20:4(5Z,8Z,11Z,14Z))[iso6]	LMGL03015921
TG 20:4_36:2	TG(16:0/20:2(11Z,14Z)/20:4(5Z,8Z,11Z,14Z))[iso6]	LMGL03010671
	TG(16:1(9Z)/20:1(11Z)/20:4(5Z,8Z,11Z,14Z))[iso6]	LMGL03010672
	TG(18:0/18:2(9Z,12Z)/20:4(5Z,8Z,11Z,14Z))[iso6]	LMGL03010676
	TG(18:1(9Z)/18:1(9Z)/20:4(5Z,8Z,11Z,14Z))[iso3]	LMGL03010677
	TG(17:2(9Z,12Z)/19:0/20:4(5Z,8Z,11Z,14Z))[iso6]	LMGL03010739
	TG(14:0/20:4(5Z,8Z,11Z,14Z)/22:2(13Z,16Z))[iso6]	LMGL03014531
	TG(14:1(9Z)/20:4(5Z,8Z,11Z,14Z)/22:1(11Z))[iso6]	LMGL03014936
	TG(17:1(9Z)/19:1(9Z)/20:4(5Z,8Z,11Z,14Z))[iso6]	LMGL03016039

MxP® Quant 500 XL kit lipid annotation	Potential isomers	Data base ID
TG 20:4_36:3	TG(16:0/20:3(8Z,11Z,14Z)/20:4(5Z,8Z,11Z,14Z))[iso6]	LMGL03010735
	TG(16:1(9Z)/20:2(11Z,14Z)/20:4(5Z,8Z,11Z,14Z))[iso6]	LMGL03010736
	TG(18:0/18:3(9Z,12Z,15Z)/20:4(5Z,8Z,11Z,14Z))[iso6]	LMGL03010740
	TG(18:1(9Z)/18:2(9Z,12Z)/20:4(5Z,8Z,11Z,14Z))[iso6]	LMGL03010741
	TG(14:0/20:4(5Z,8Z,11Z,14Z)/22:3(10Z,13Z,16Z))[iso6]	LMGL03014532
	TG(14:1(9Z)/20:4(5Z,8Z,11Z,14Z)/22:2(13Z,16Z))[iso6]	LMGL03014937
	TG(17:2(9Z,12Z)/19:1(9Z)/20:4(5Z,8Z,11Z,14Z))[iso6]	LMGL03016118
	TG(18:0/18:3(6Z,9Z,12Z)/20:4(5Z,8Z,11Z,14Z))[iso6]	LMGL03016158
TG 20:4_36:4	TG(16:0/20:4(5Z,8Z,11Z,14Z)/20:4(5Z,8Z,11Z,14Z))[iso3]	LMGL03010801
	TG(16:1(9Z)/20:3(8Z,11Z,14Z)/20:4(5Z,8Z,11Z,14Z))[iso6]	LMGL03010802
	TG(18:1(9Z)/18:3(9Z,12Z,15Z)/20:4(5Z,8Z,11Z,14Z))[iso6]	LMGL03010807
	TG(18:2(9Z,12Z)/18:2(9Z,12Z)/20:4(5Z,8Z,11Z,14Z))[iso3]	LMGL03010808
	TG(14:0/20:4(5Z,8Z,11Z,14Z)/22:4(7Z,10Z,13Z,16Z))[iso6]	LMGL03014533
	TG(14:1(9Z)/20:4(5Z,8Z,11Z,14Z)/22:3(10Z,13Z,16Z))[iso6]	LMGL03014938
	TG(18:0/18:4(6Z,9Z,12Z,15Z)/20:4(5Z,8Z,11Z,14Z))[iso6]	LMGL03016177
	TG(18:1(9Z)/18:3(6Z,9Z,12Z)/20:4(5Z,8Z,11Z,14Z))[iso6]	LMGL03016229
TG 20:4_36:5	TG(16:1(9Z)/20:4(5Z,8Z,11Z,14Z)/20:4(5Z,8Z,11Z,14Z))[iso3]	LMGL03010870
	TG(18:2(9Z,12Z)/18:3(9Z,12Z,15Z)/20:4(5Z,8Z,11Z,14Z))[iso6]	LMGL03010876
	TG(16:0/20:4(5Z,8Z,11Z,14Z)/20:5(5Z,8Z,11Z,14Z,17Z))[iso6]	LMGL03010877
	TG(14:0/20:4(5Z,8Z,11Z,14Z)/22:5(7Z,10Z,13Z,16Z,19Z))[iso6]	LMGL03014534
	TG(14:1(9Z)/20:4(5Z,8Z,11Z,14Z)/22:4(7Z,10Z,13Z,16Z))[iso6]	LMGL03014939
	TG(18:1(9Z)/18:4(6Z,9Z,12Z,15Z)/20:4(5Z,8Z,11Z,14Z))[iso6]	LMGL03016248
	TG(18:2(9Z,12Z)/18:3(6Z,9Z,12Z)/20:4(5Z,8Z,11Z,14Z))[iso6]	LMGL03016296
TG 20:5_34:0	TG(17:0/17:0/20:5(5Z,8Z,11Z,14Z,17Z))[iso3]	LMGL03010320
	TG(16:0/18:0/20:5(5Z,8Z,11Z,14Z,17Z))[iso6]	LMGL03010359
	TG(12:0/20:5(5Z,8Z,11Z,14Z,17Z)/22:0)[iso6]	LMGL03013637
	TG(13:0/20:5(5Z,8Z,11Z,14Z,17Z)/21:0)[iso6]	LMGL03014101
	TG(14:0/20:0/20:5(5Z,8Z,11Z,14Z,17Z))[iso6]	LMGL03014485
	TG(15:0/19:0/20:5(5Z,8Z,11Z,14Z,17Z))[iso6]	LMGL03015242
TG 20:5_34:1	TG(17:0/17:1(9Z)/20:5(5Z,8Z,11Z,14Z,17Z))[iso6]	LMGL03010361
	TG(16:0/18:1(9Z)/20:5(5Z,8Z,11Z,14Z,17Z))[iso6]	LMGL03010403
	TG(16:1(9Z)/18:0/20:5(5Z,8Z,11Z,14Z,17Z))[iso6]	LMGL03010404
	TG(12:0/20:5(5Z,8Z,11Z,14Z,17Z)/22:1(11Z))[iso6]	LMGL03013638
	TG(14:0/20:1(11Z)/20:5(5Z,8Z,11Z,14Z,17Z))[iso6]	LMGL03014497
	TG(14:1(9Z)/20:0/20:5(5Z,8Z,11Z,14Z,17Z))[iso6]	LMGL03014891
	TG(15:0/19:1(9Z)/20:5(5Z,8Z,11Z,14Z,17Z))[iso6]	LMGL03015256
	TG(15:1(9Z)/19:0/20:5(5Z,8Z,11Z,14Z,17Z))[iso6]	LMGL03015593
TG 20:5_34:2	TG(17:0/17:2(9Z,12Z)/20:5(5Z,8Z,11Z,14Z,17Z))[iso6]	LMGL03010405
	TG(17:1(9Z)/17:1(9Z)/20:5(5Z,8Z,11Z,14Z,17Z))[iso3]	LMGL03010406
	TG(16:0/18:2(9Z,12Z)/20:5(5Z,8Z,11Z,14Z,17Z))[iso6]	LMGL03010451
	TG(16:1(9Z)/18:1(9Z)/20:5(5Z,8Z,11Z,14Z,17Z))[iso6]	LMGL03010452
	TG(12:0/20:5(5Z,8Z,11Z,14Z,17Z)/22:2(13Z,16Z))[iso6]	LMGL03013639
	TG(14:0/20:2(11Z,14Z)/20:5(5Z,8Z,11Z,14Z,17Z))[iso6]	LMGL03014508
	TG(14:1(9Z)/20:1(11Z)/20:5(5Z,8Z,11Z,14Z,17Z))[iso6]	LMGL03014903
	TG(15:1(9Z)/19:1(9Z)/20:5(5Z,8Z,11Z,14Z,17Z))[iso6]	LMGL03015607
TG 20:5_36:2	TG(16:0/20:2(11Z,14Z)/20:5(5Z,8Z,11Z,14Z,17Z))[iso6]	LMGL03010742
	TG(16:1(9Z)/20:1(11Z)/20:5(5Z,8Z,11Z,14Z,17Z))[iso6]	LMGL03010743
	TG(18:0/18:2(9Z,12Z)/20:5(5Z,8Z,11Z,14Z,17Z))[iso6]	LMGL03010747
	TG(18:1(9Z)/18:1(9Z)/20:5(5Z,8Z,11Z,14Z,17Z))[iso3]	LMGL03010748
	TG(17:2(9Z,12Z)/19:0/20:5(5Z,8Z,11Z,14Z,17Z))[iso6]	LMGL03010813
	TG(14:0/20:5(5Z,8Z,11Z,14Z,17Z)/22:2(13Z,16Z))[iso6]	LMGL03014539
	TG(14:1(9Z)/20:5(5Z,8Z,11Z,14Z,17Z)/22:1(11Z))[iso6]	LMGL03014944
	TG(17:1(9Z)/19:1(9Z)/20:5(5Z,8Z,11Z,14Z,17Z))[iso6]	LMGL03016040

MxP® Quant 500 XL kit lipid annotation	Potential isomers	Data base ID
TG 20:5_36:3	TG(16:0/20:3(8Z,11Z,14Z)/20:5(5Z,8Z,11Z,14Z,17Z))[iso6]	LMGL03010809
	TG(16:1(9Z)/20:2(11Z,14Z)/20:5(5Z,8Z,11Z,14Z,17Z))[iso6]	LMGL03010810
	TG(18:0/18:3(9Z,12Z,15Z)/20:5(5Z,8Z,11Z,14Z,17Z))[iso6]	LMGL03010814
	TG(18:1(9Z)/18:2(9Z,12Z)/20:5(5Z,8Z,11Z,14Z,17Z))[iso6]	LMGL03010815
	TG(14:0/20:5(5Z,8Z,11Z,14Z,17Z)/22:3(10Z,13Z,16Z))[iso6]	LMGL03014540
	TG(14:1(9Z)/20:5(5Z,8Z,11Z,14Z,17Z)/22:2(13Z,16Z))[iso6]	LMGL03014945
	TG(17:2(9Z,12Z)/19:1(9Z)/20:5(5Z,8Z,11Z,14Z,17Z))[iso6]	LMGL03016119
	TG(18:0/18:3(6Z,9Z,12Z)/20:5(5Z,8Z,11Z,14Z,17Z))[iso6]	LMGL03016159
TG 22:0_32:4	TG(12:0/20:4(5Z,8Z,11Z,14Z)/22:0)[iso6]	LMGL03013629
	TG(14:0/18:4(6Z,9Z,12Z,15Z)/22:0)[iso6]	LMGL03014445
	TG(14:1(9Z)/18:3(6Z,9Z,12Z)/22:0)[iso6]	LMGL03014818
	TG(14:1(9Z)/18:3(9Z,12Z,15Z)/22:0)[iso6]	LMGL03014835
TG 22:1_32:5	TG(12:0/20:5(5Z,8Z,11Z,14Z,17Z)/22:1(11Z))[iso6]	LMGL03013638
	TG(14:1(9Z)/18:4(6Z,9Z,12Z,15Z)/22:1(11Z))[iso6]	LMGL03014852
TG 22:2_32:4	TG(12:0/20:4(5Z,8Z,11Z,14Z)/22:2(13Z,16Z))[iso6]	LMGL03013631
	TG(14:0/18:4(6Z,9Z,12Z,15Z)/22:2(13Z,16Z))[iso6]	LMGL03014447
	TG(14:1(9Z)/18:3(6Z,9Z,12Z)/22:2(13Z,16Z))[iso6]	LMGL03014820
	TG(14:1(9Z)/18:3(9Z,12Z,15Z)/22:2(13Z,16Z))[iso6]	LMGL03014837
TG 22:3_30:2	TG(15:1(9Z)/15:1(9Z)/22:3(10Z,13Z,16Z))[iso3]	LMGL03012950
	TG(12:0/18:2(9Z,12Z)/22:3(10Z,13Z,16Z))[iso6]	LMGL03013497
	TG(13:0/17:2(9Z,12Z)/22:3(10Z,13Z,16Z))[iso6]	LMGL03013902
	TG(14:1(9Z)/16:1(9Z)/22:3(10Z,13Z,16Z))[iso6]	LMGL03014674
TG 22:4_32:0	TG(16:0/16:0/22:4(7Z,10Z,13Z,16Z))[iso3]	LMGL03010418
	TG(12:0/20:0/22:4(7Z,10Z,13Z,16Z))[iso6]	LMGL03013591
	TG(13:0/19:0/22:4(7Z,10Z,13Z,16Z))[iso6]	LMGL03014029
	TG(14:0/18:0/22:4(7Z,10Z,13Z,16Z))[iso6]	LMGL03014359
	TG(15:0/17:0/22:4(7Z,10Z,13Z,16Z))[iso6]	LMGL03015077
TG 22:4_32:2	TG(16:1(9Z)/16:1(9Z)/22:4(7Z,10Z,13Z,16Z))[iso3]	LMGL03010524
	TG(12:0/20:2(11Z,14Z)/22:4(7Z,10Z,13Z,16Z))[iso6]	LMGL03013614
	TG(14:0/18:2(9Z,12Z)/22:4(7Z,10Z,13Z,16Z))[iso6]	LMGL03014398
	TG(14:1(9Z)/18:1(9Z)/22:4(7Z,10Z,13Z,16Z))[iso6]	LMGL03014785
	TG(15:0/17:2(9Z,12Z)/22:4(7Z,10Z,13Z,16Z))[iso6]	LMGL03015122
	TG(15:1(9Z)/17:1(9Z)/22:4(7Z,10Z,13Z,16Z))[iso6]	LMGL03015451
TG 22:4_34:2	TG(17:0/17:2(9Z,12Z)/22:4(7Z,10Z,13Z,16Z))[iso6]	LMGL03010777
	TG(17:1(9Z)/17:1(9Z)/22:4(7Z,10Z,13Z,16Z))[iso3]	LMGL03010778
	TG(16:0/18:2(9Z,12Z)/22:4(7Z,10Z,13Z,16Z))[iso6]	LMGL03010845
	TG(16:1(9Z)/18:1(9Z)/22:4(7Z,10Z,13Z,16Z))[iso6]	LMGL03010846
	TG(12:0/22:2(13Z,16Z)/22:4(7Z,10Z,13Z,16Z))[iso6]	LMGL03013663
	TG(14:0/20:2(11Z,14Z)/22:4(7Z,10Z,13Z,16Z))[iso6]	LMGL03014514
	TG(14:1(9Z)/20:1(11Z)/22:4(7Z,10Z,13Z,16Z))[iso6]	LMGL03014909
	TG(15:1(9Z)/19:1(9Z)/22:4(7Z,10Z,13Z,16Z))[iso6]	LMGL03015613
TG 22:5_32:0	TG(16:0/16:0/22:5(7Z,10Z,13Z,16Z,19Z))[iso3]	LMGL03010470
	TG(12:0/20:0/22:5(7Z,10Z,13Z,16Z,19Z))[iso6]	LMGL03013592
	TG(13:0/19:0/22:5(7Z,10Z,13Z,16Z,19Z))[iso6]	LMGL03014030
	TG(14:0/18:0/22:5(7Z,10Z,13Z,16Z,19Z))[iso6]	LMGL03014360
	TG(15:0/17:0/22:5(7Z,10Z,13Z,16Z,19Z))[iso6]	LMGL03015078
TG 22:5_32:1	TG(16:0/16:1(9Z)/22:5(7Z,10Z,13Z,16Z,19Z))[iso6]	LMGL03010525
	TG(12:0/20:1(11Z)/22:5(7Z,10Z,13Z,16Z,19Z))[iso6]	LMGL03013604
	TG(13:0/19:1(9Z)/22:5(7Z,10Z,13Z,16Z,19Z))[iso6]	LMGL03014044
	TG(14:0/18:1(9Z)/22:5(7Z,10Z,13Z,16Z,19Z))[iso6]	LMGL03014380
	TG(14:1(9Z)/18:0/22:5(7Z,10Z,13Z,16Z,19Z))[iso6]	LMGL03014766
	TG(15:0/17:1(9Z)/22:5(7Z,10Z,13Z,16Z,19Z))[iso6]	LMGL03015101
	TG(15:1(9Z)/17:0/22:5(7Z,10Z,13Z,16Z,19Z))[iso6]	LMGL03015429

MxP® Quant 500 XL kit lipid annotation	Potential isomers	Data base ID
TG 22:5_34:1	TG(17:0/17:1(9Z)/22:5(7Z,10Z,13Z,16Z,19Z))[iso6]	LMGL03010781
	TG(16:0/18:1(9Z)/22:5(7Z,10Z,13Z,16Z,19Z))[iso6]	LMGL03010849
	TG(16:1(9Z)/18:0/22:5(7Z,10Z,13Z,16Z,19Z))[iso6]	LMGL03010850
	TG(12:0/22:1(11Z)/22:5(7Z,10Z,13Z,16Z,19Z))[iso6]	LMGL03013660
	TG(14:0/20:1(11Z)/22:5(7Z,10Z,13Z,16Z,19Z))[iso6]	LMGL03014504
	TG(14:1(9Z)/20:0/22:5(7Z,10Z,13Z,16Z,19Z))[iso6]	LMGL03014898
	TG(15:0/19:1(9Z)/22:5(7Z,10Z,13Z,16Z,19Z))[iso6]	LMGL03015263
	TG(15:1(9Z)/19:0/22:5(7Z,10Z,13Z,16Z,19Z))[iso6]	LMGL03015600
TG 22:5_34:2	TG(17:0/17:2(9Z,12Z)/22:5(7Z,10Z,13Z,16Z,19Z))[iso6]	LMGL03010851
	TG(17:1(9Z)/17:1(9Z)/22:5(7Z,10Z,13Z,16Z,19Z))[iso3]	LMGL03010852
	TG(16:0/18:2(9Z,12Z)/22:5(7Z,10Z,13Z,16Z,19Z))[iso6]	LMGL03010921
	TG(16:1(9Z)/18:1(9Z)/22:5(7Z,10Z,13Z,16Z,19Z))[iso6]	LMGL03010922
	TG(12:0/22:2(13Z,16Z)/22:5(7Z,10Z,13Z,16Z,19Z))[iso6]	LMGL03013664
	TG(14:0/20:2(11Z,14Z)/22:5(7Z,10Z,13Z,16Z,19Z))[iso6]	LMGL03014515
	TG(14:1(9Z)/20:1(11Z)/22:5(7Z,10Z,13Z,16Z,19Z))[iso6]	LMGL03014910
	TG(15:1(9Z)/19:1(9Z)/22:5(7Z,10Z,13Z,16Z,19Z))[iso6]	LMGL03015614
TG 22:5_34:3	TG(17:1(9Z)/17:2(9Z,12Z)/22:5(7Z,10Z,13Z,16Z,19Z))[iso6]	LMGL03010924
	TG(16:0/18:3(9Z,12Z,15Z)/22:5(7Z,10Z,13Z,16Z,19Z))[iso6]	LMGL03010994
	TG(16:1(9Z)/18:2(9Z,12Z)/22:5(7Z,10Z,13Z,16Z,19Z))[iso6]	LMGL03010995
	TG(12:0/22:3(10Z,13Z,16Z)/22:5(7Z,10Z,13Z,16Z,19Z))[iso6]	LMGL03013667
	TG(14:0/20:3(8Z,11Z,14Z)/22:5(7Z,10Z,13Z,16Z,19Z))[iso6]	LMGL03014525
	TG(14:1(9Z)/20:2(11Z,14Z)/22:5(7Z,10Z,13Z,16Z,19Z))[iso6]	LMGL03014921
	TG(16:0/18:3(6Z,9Z,12Z)/22:5(7Z,10Z,13Z,16Z,19Z))[iso6]	LMGL03015751
TG 22:6_32:0	TG(16:0/16:0/22:6(4Z,7Z,10Z,13Z,16Z,19Z))[iso3]	LMGL03010526
	TG(12:0/20:0/22:6(4Z,7Z,10Z,13Z,16Z,19Z))[iso6]	LMGL03013593
	TG(13:0/19:0/22:6(4Z,7Z,10Z,13Z,16Z,19Z))[iso6]	LMGL03014031
	TG(14:0/18:0/22:6(4Z,7Z,10Z,13Z,16Z,19Z))[iso6]	LMGL03014361
	TG(15:0/17:0/22:6(4Z,7Z,10Z,13Z,16Z,19Z))[iso6]	LMGL03015079
TG 22:6_32:1	TG(16:0/16:1(9Z)/22:6(4Z,7Z,10Z,13Z,16Z,19Z))[iso6]	LMGL03010586
	TG(12:0/20:1(11Z)/22:6(4Z,7Z,10Z,13Z,16Z,19Z))[iso6]	LMGL03013605
	TG(13:0/19:1(9Z)/22:6(4Z,7Z,10Z,13Z,16Z,19Z))[iso6]	LMGL03014045
	TG(14:0/18:1(9Z)/22:6(4Z,7Z,10Z,13Z,16Z,19Z))[iso6]	LMGL03014381
	TG(14:1(9Z)/18:0/22:6(4Z,7Z,10Z,13Z,16Z,19Z))[iso6]	LMGL03014767
	TG(15:0/17:1(9Z)/22:6(4Z,7Z,10Z,13Z,16Z,19Z))[iso6]	LMGL03015102
	TG(15:1(9Z)/17:0/22:6(4Z,7Z,10Z,13Z,16Z,19Z))[iso6]	LMGL03015430
TG 22:6_34:1	TG(17:0/17:1(9Z)/22:6(4Z,7Z,10Z,13Z,16Z,19Z))[iso6]	LMGL03010855
	TG(16:0/18:1(9Z)/22:6(4Z,7Z,10Z,13Z,16Z,19Z))[iso6]	LMGL03010925
	TG(16:1(9Z)/18:0/22:6(4Z,7Z,10Z,13Z,16Z,19Z))[iso6]	LMGL03010926
	TG(12:0/22:1(11Z)/22:6(4Z,7Z,10Z,13Z,16Z,19Z))[iso6]	LMGL03013661
	TG(14:0/20:1(11Z)/22:6(4Z,7Z,10Z,13Z,16Z,19Z))[iso6]	LMGL03014505
	TG(14:1(9Z)/20:0/22:6(4Z,7Z,10Z,13Z,16Z,19Z))[iso6]	LMGL03014899
	TG(15:0/19:1(9Z)/22:6(4Z,7Z,10Z,13Z,16Z,19Z))[iso6]	LMGL03015264
	TG(15:1(9Z)/19:0/22:6(4Z,7Z,10Z,13Z,16Z,19Z))[iso6]	LMGL03015601
TG 22:6_34:2	TG(17:0/17:2(9Z,12Z)/22:6(4Z,7Z,10Z,13Z,16Z,19Z))[iso6]	LMGL03010927
	TG(17:1(9Z)/17:1(9Z)/22:6(4Z,7Z,10Z,13Z,16Z,19Z))[iso3]	LMGL03010928
	TG(16:0/18:2(9Z,12Z)/22:6(4Z,7Z,10Z,13Z,16Z,19Z))[iso6]	LMGL03010999
	TG(16:1(9Z)/18:1(9Z)/22:6(4Z,7Z,10Z,13Z,16Z,19Z))[iso6]	LMGL03011000
	TG(12:0/22:2(13Z,16Z)/22:6(4Z,7Z,10Z,13Z,16Z,19Z))[iso6]	LMGL03013665
	TG(14:0/20:2(11Z,14Z)/22:6(4Z,7Z,10Z,13Z,16Z,19Z))[iso6]	LMGL03014516
	TG(14:1(9Z)/20:1(11Z)/22:6(4Z,7Z,10Z,13Z,16Z,19Z))[iso6]	LMGL03014911
	TG(15:1(9Z)/19:1(9Z)/22:6(4Z,7Z,10Z,13Z,16Z,19Z))[iso6]	LMGL03015615

MxP® Quant 500 XL kit lipid annotation	Potential isomers	Data base ID
TG 22:6_34:3	TG(17:1(9Z)/17:2(9Z,12Z)/22:6(4Z,7Z,10Z,13Z,16Z,19Z))[iso6]	LMGL03011002
	TG(16:0/18:3(9Z,12Z,15Z)/22:6(4Z,7Z,10Z,13Z,16Z,19Z))[iso6]	LMGL03011074
	TG(16:1(9Z)/18:2(9Z,12Z)/22:6(4Z,7Z,10Z,13Z,16Z,19Z))[iso6]	LMGL03011075
	TG(12:0/22:3(10Z,13Z,16Z)/22:6(4Z,7Z,10Z,13Z,16Z,19Z))[iso6]	LMGL03013668
	TG(14:0/20:3(8Z,11Z,14Z)/22:6(4Z,7Z,10Z,13Z,16Z,19Z))[iso6]	LMGL03014526
	TG(14:1(9Z)/20:2(11Z,14Z)/22:6(4Z,7Z,10Z,13Z,16Z,19Z))[iso6]	LMGL03014922
	TG(16:0/18:3(6Z,9Z,12Z)/22:6(4Z,7Z,10Z,13Z,16Z,19Z))[iso6]	LMGL03015752

biocrates
The future of research and health

Ordering

Webshop	shop.biocrates.com
Information request	biocrates.com/contact
E-mail	sales@biocrates.com

Technical support

FAQs	biocrates.com/support
E-mail	support@biocrates.com

For research use only | not for use in diagnostic procedures

biocrates.com

Video tutorials

Frequently Asked Questions (FAQ)

biocrates life sciences ag
Eduard-Bodem-Gasse 8 I 6020 Innsbruck I Austria
biocrates.com

EU | APAC | RoW +43 512 579 823
North America +1 774 424 81 50
office@biocrates.com
