# Supplementary material for: Fingolimod normalizes metabolic signatures associated with synaptic plasticity and memory in APP/PS1 model: Sphingosine-1-phosphate receptor a therapeutic target for Alzheimer’s
Source: Sci Rep. 2026 Mar 10;16:12835. doi: 10.1038/s41598-026-42518-8 (PMC13096423; doi:10.1038/s41598-026-42518-8)
Supplement: Supplementary file 5 — Supplementary Information 5. [file 41598_2026_42518_MOESM5_ESM.pdf]

| Name | Long name | Formula |
|---|---|---|
| **Fischer ratio** | Fischer ratio | (Ile + Leu + Val) / (Phe + Trp + Tyr) |
| **Glutaminolysis rate** | Glutaminolysis rate | (Ala + Asp + Glu + Lac + Suc) / Gln |
| **PKU** | Phenylketonuria | Tyr / Phe |
| **Sum of BCAAs** | Sum of branched-chain amino acids | Ile + Leu + Val |
| **Valinemia** | Valinemia | Val / Phe |
| **3-Met-His synthesis** | 3-Methylhistidine synthesis | 3-Met-His / (Anserine + Carnosine) |
| **AABA synthesis** | alpha-Aminobutyric acid synthesis | AABA / Thr |
| **Betaine synthesis** | Betaine synthesis | Betaine / Choline |
| **HArg synthesis** | Homoarginine synthesis | HArg / (Arg + Lys) |
| **IDO activity** | Indoleamine 2,3-dioxygenase activity | Kynurenine / Trp |
| **Ratio of SG to hexose** | Ratio of serine and glycine to hexose | (Ser + Gly) / Hexose |
| **Ratio of SGA to hexose** | Ratio of serine, glycine, and alanine to hexose | (Ser + Gly + Ala) / Hexose |
| **Sum of asym. and sym. Arg methylation** | Sum of asymmetrical and symmetrical arginine methylation | (ADMA + SDMA) / Arg |
| **Sum of betaine and related metabolites** | Sum of betaine and related metabolites | Betaine + PheAlaBetaine + ProBetaine + TrpBetaine |
| **Symmetrical Arg methylation** | Symmetrical arginine methylation | SDMA / Arg |
| **β-Ala synthesis** | beta-Alanine synthesis | beta-Ala / Carnosine |
| **Serotonin synthesis** | Serotonin synthesis | Serotonin / Trp |
| **Spermidine synthesis** | Spermidine synthesis | Spermidine / Putrescine |
| **Sum of neurotransmitters** | Sum of neurotransmitters | Dopamine + Histamine + Serotonin |
| **HipAcid synthesis** | Hippuric acid synthesis | HipAcid / Gly |
| **LDH activity** | Lactate dehydrogenase activity | Lac / Hexose |
| **MAGL activity** | Monoacylglycerol lipase activity | FA 20:4 / MG 20:4 |
| **Sum of purines** | Sum of purine derivatives | Hypoxanthine + Xanthine |
| **Sum of choline lipids** | Sum of choline and choline-based lipids | Choline + LPC xx:x + PC (O-)xx:x |
| **Carnitine uptake defect** | Carnitine uptake defect | (C0 + C2 + C3 + C16 + C18 + C18:1) / Cit |
| **Ratio of ACs to FAs** | Ratio of acylcarnitines to fatty acids | (C2-18 + C2-18:x + Cx-DC + Cx:x-DC + Cx-OH + Cx:x-OH + C5-M-DC) / FA xx:x |
| **Sum of ACs** | Sum of acylcarnitines | C2-18 + C2-18:x + Cx-DC + Cx:x-DC + Cx-OH + Cx:x-OH + C5-M-DC |
| **Sum of long-chain ACs** | Sum of long-chain acylcarnitines | C14-18 + C14-18:x + C14:1-OH + C14:2-OH + C16-OH + C16:1-OH + C16:2-OH + C18:1-OH |
| **Sum of MUFA-ACs** | Sum of monounsaturated fatty acid acylcarnitines | C5:1-DC + Cx:1 + Cx:1-OH |
| **Sum of SFA-ACs** | Sum of saturated fatty acid acylcarnitines | C2-18 + C5-M-DC + Cx-DC + Cx-OH |
| **Sum of short-chain ACs** | Sum of short-chain acylcarnitines | C2-5 + C2-5:x + C3-DC (C4-OH) + C3-OH + C5-DC (C6-OH) + C5-M-DC + C5-OH (C3-DC-M) + C5:1-DC |

| | | |
|---|---|---|
| **ω-Oxidation** | omega-Oxidation | (Cx-DC + Cx:x-DC) / (C2-18 + C2-18:x + C5-M-DC + Cx-DC + Cx:x-DC + Cx-OH + Cx:x-OH) |
| **ATX activity** | Autotaxin activity | (LPA xx:x + Choline) / LPC xx:x |
| **Ratio of MUFA-LPCs to SFA-LPCs** | Ratio of monounsaturated fatty acid lysophosphatidylcholines to saturated fatty acid lysophosphatidylcholines | LPC xx:1 / LPC xx:0 |
| **Ratio of UFA-LPCs to SFA-LPCs** | Ratio of unsaturated fatty acid lysophosphatidylcholines to saturated fatty acid lysophosphatidylcholines | LPC xx:1-4 / LPC xx:0 |
| **Sum of (L)PC (O)s** | Sum of phosphatidylcholines and lysophosphatidylcholines | PC (O-)xx:x + LPC xx:x |
| **Ratio of MUFA-LPEs to SFA-LPEs** | Ratio of monounsaturated fatty acid acyl-lysophosphatidylethanolamines to saturated fatty acid acyl-lysophosphatidylethanolamines | LPE xx:1 / LPE xx:0 |
| **Ratio of MUFA-LPEs P to SFA-LPEs P** | Ratio of monounsaturated fatty acid lysophosphatidylethanolamine plasmalogens to saturated fatty acid lysophosphatidylethanolamine plasmalogens | LPE P-xx:1 / LPE P-xx:0 |
| **Ratio of MUFA-LPE (P)s to SFA-LPE (P)s** | Ratio of monounsaturated fatty acid lysophosphatidylethanolamines to saturated fatty acid lysophosphatidylethanolamines | LPE (P-)xx:1 / LPE (P-)xx:0 |
| **Ratio of PUFA-LPEs to SFA-LPEs** | Ratio of polyunsaturated fatty acid acyl-lysophosphatidylethanolamines to saturated fatty acid acyl-lysophosphatidylethanolamines | LPE xx:2-6 / LPE xx:0 |
| **Ratio of PUFA-LPE (P)s to SFA-LPE (P)s** | Ratio of polyunsaturated fatty acid lysophosphatidylethanolamines to saturated fatty acid lysophosphatidylethanolamines | LPE (P-)xx:2-6 / LPE (P-)xx:0 |
| **Ratio of UFA-LPEs to SFA-LPEs** | Ratio of unsaturated fatty acid acyl-lysophosphatidylethanolamines to saturated fatty acid acyl-lysophosphatidylethanolamines | LPE xx:1-6 / LPE xx:0 |
| **Ratio of UFA-LPEs P to SFA-LPEs P** | Ratio of unsaturated fatty acid lysophosphatidylethanolamine plasmalogens to saturated fatty acid lysophosphatidylethanolamine plasmalogens | LPE P-xx:1-6 / LPE P-xx:0 |
| **Ratio of UFA-LPE (P)s to SFA-LPE (P)s** | Ratio of unsaturated fatty acid lysophosphatidylethanolamines to saturated fatty acid lysophosphatidylethanolamines | LPE (P-)xx:1-6 / LPE (P-)xx:0 |
| **Ratio of MUFA-PEs to SFA-PEs** | Ratio of monounsaturated fatty acid diacyl-phosphatidylethanolamines to saturated fatty acid diacyl-phosphatidylethanolamines | PE xx:1 / PE xx:0 |
| **Ratio of PUFA-PEs to MUFA-PEs** | Ratio of polyunsaturated fatty acid diacyl-phosphatidylethanolamines to | PE xx:3-12 / PE xx:1 |

| | | |
|---|---|---|
| | monounsaturated fatty acid diacyl-phosphatidylethanolamines | |
| **Ratio of PUFA-PEs to SFA-PEs** | Ratio of polyunsaturated fatty acid diacyl-phosphatidylethanolamines to saturated fatty acid diacyl-phosphatidylethanolamines | PE xx:3-12 / PE xx:0 |
| **Ratio of PUFA-PEs P to SFA-PEs P** | Ratio of polyunsaturated fatty acid phosphatidylethanolamine plasmalogens to saturated fatty acid phosphatidylethanolamine plasmalogens | PE P-xx:x/xx:2-6 / PE P-xx:0/xx:0 |
| **Ratio of PUFA-PE (P)s to SFA-PE (P)s** | Ratio of polyunsaturated fatty acid phosphatidylethanolamines to saturated fatty acid phosphatidylethanolamines | (PE xx:3-12 + PE P-xx:x/xx:2-6) / (PE xx:0 + PE P-xx:0/xx:0) |
| **Ratio of UFA-PEs to SFA-PEs** | Ratio of unsaturated fatty acid diacyl-phosphatidylethanolamines to saturated fatty acid diacyl-phosphatidylethanolamines | PE xx:1-12 / PE xx:0 |
| **Ratio of UFA-PEs P to SFA-PEs P** | Ratio of unsaturated fatty acid phosphatidylethanolamine plasmalogens to saturated fatty acid phosphatidylethanolamine plasmalogens | (PE P-xx:1-6/xx:x + PE P-xx:x/xx:1-6) / PE P-xx:0/xx:0 |
| **Sum of SFA-PEs** | Sum of saturated fatty acid diacyl-phosphatidylethanolamines | Sum of PE xx:0 |
| **Ratio of PUFA-PGs to MUFA-PGs** | Ratio of polyunsaturated fatty acid phosphatidylglycerols to monounsaturated fatty acid phosphatidylglycerols | (PG xx:2-6_xx:x + PG xx:x_xx:2-6) / (PG xx:1_xx:0-1 + PG xx:0-1_xx:1) |
| **Sum of UFA-LPIs** | Sum of unsaturated fatty acid lysophosphatidylinositols | Sum of LPI xx:1-4 |
| **Sum of PUFA-LPIs** | Sum of polyunsaturated fatty acid lysophosphatidylinositols | Sum of LPI xx:2-4 |
| **Ratio of PI 18:0_20:4 to PIs** | Ratio of PI 18:0_20:4 to phosphatidylinositols | PI 18:0_20:4 / PI xx:x_xx:x |
| **Sum of LCFA-LPSs** | Sum of long-chain fatty acid lysophosphatidylserines | Sum of LPS 16-20:x |
| **Sum of LPSs** | Sum of lysophosphatidylserines | Sum of LPS xx:x |
| **Sum of SFA-LPSs** | Sum of saturated fatty acid lysophosphatidylserines | Sum of LPS xx:0 |
| **Ratio of PUFA-PSs to MUFA-PSs** | Ratio of polyunsaturated fatty acid phosphatidylserines to monounsaturated fatty acid phosphatidylserines | PS xx:3-8 / PS xx:1 |
| **LPP3 activity (4)** | Lipid phosphate phosphatase 3 activity (4) | SPB d18:1 / SPBP d18:1 |
| **LPP3 activity (5)** | Lipid phosphate phosphatase 3 activity (5) | SPB dxx:1 / SPBP dxx:1 |
| **Ratio of SphoPs to SphaPs** | Ratio of sphingosine phosphates to sphinganine phosphates | SPBP dxx:1 / SPBP dxx:0 |
| **SphK activity (4)** | Sphingosine kinase activity (4) | SPBP d18:1 / SPB d18:1 |

| | | |
|---|---|---|
| **SphK activity (5)** | Sphingosine kinase activity (5) | SPBP dxx:1 / SPB dxx:1 |
| **Sum of SPBPs** | Sum of sphingoid base phosphates | Sum of SPBP dxx:x |
| **Sum of SphoPs** | Sum of sphingosine phosphates | Sum of SPBP dxx:1 |
| **Ratio of OC-FA SMs to EC-FA SMs** | Ratio of odd-chain fatty acid sphingomyelins to even-chain fatty acid sphingomyelins | (SM x1:x + SM x3:x + SM x5:x) / (SM x2:x + SM x4:x + SM x6:x + SM x8:x + SM x0:x) |
| **Ratio of SMs to Cer** | Ratio of sphingomyelins to ceramides | SM xx:x / Cer d1x:x/xx:x(-OH) |
| **SMase activity** | Sphingomyelinase activity | Cer d1x:x/xx:x(-OH) / SM xx:x |
| **Sum of Cer** | Sum of ceramides | Cer d1x:x/xx:x(-OH) |
| **Sum of VLCFA-Cer** | Sum of very long-chain fatty acid ceramides | Sum of Cer d1x:x/22-26:x |
| **Sum of SFA-TGs** | Sum of saturated fatty acid triglycerides | Sum of TG xx:0_xx:0 |
