# Supplementary material for: Fingolimod normalizes metabolic signatures associated with synaptic plasticity and memory in APP/PS1 model: Sphingosine-1-phosphate receptor a therapeutic target for Alzheimer’s
Source: Sci Rep. 2026 Mar 10;16:12835. doi: 10.1038/s41598-026-42518-8 (PMC13096423; doi:10.1038/s41598-026-42518-8)
Supplement: Supplementary file 6 — Supplementary Information 6. [file 41598_2026_42518_MOESM6_ESM.pdf]

Supplementary Figures

Supplementary Fig. S1: Metabolic effects of FTY720 treatment in APP/PS1 mice on individual lipid species

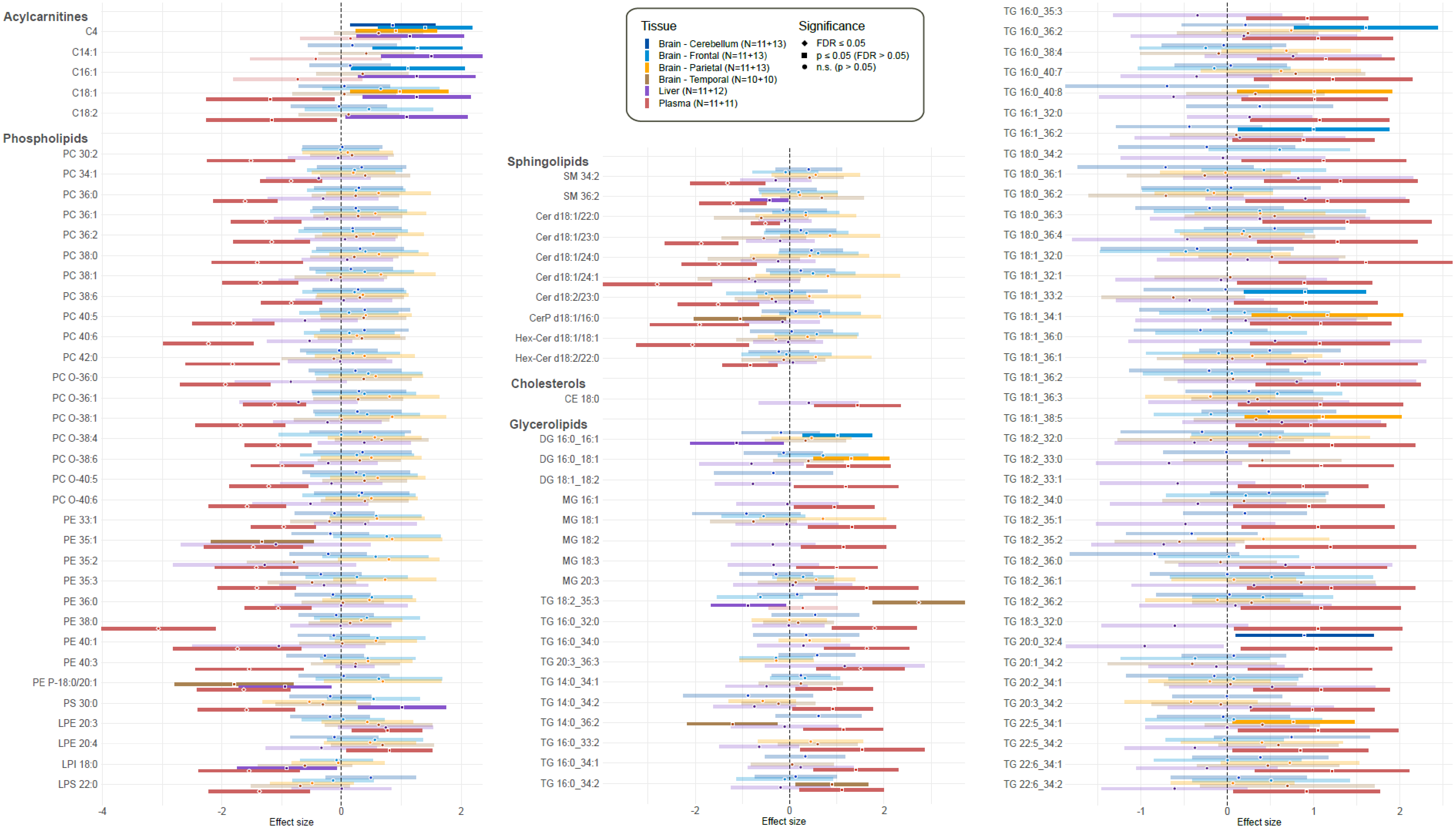

Forest plot with metabolic differences in APP/PS1-FTY720 mice that are statistically significant (FDR ≤ 0.05 for at least 1 tissue among APP/PS1 mice or APP/PS1 and WT combined) compared to APP/PS1-vehicle mice in a linear regression model (for details, see **Methods**, section Statistical analysis – Differential analysis). This figure shows results for individual lipid species. The normalized regression coefficients are shown on the x axis as points, with the shape denoting the statistical significance (diamond – FDR ≤ 0.05; square – FDR > 0.05 and p ≤ 0.05; circle – p > 0.05), located in the middle of a horizontal line depicting 95% confidence intervals, with lower opacity for insignificant results (p > 0.05). The vertical dashed line across 0 represents no group difference. Positive values mean an increase in APP/PS1-FTY720 group. Individual types of tissue are color-coded (dark blue – cerebellum; light blue – frontal cortex; orange – parietal cortex; brown – temporal cortex; purple – liver; red – plasma). Sample counts for each tissue and group are included as "N" with APP/PS1-vehicle group listed first.

Supplementary Fig. S2: Selection of top metabolic analytes best corresponding to FTY720-related correction of abnormal behavioral and electrophysiological tests in APP/PS1 mice

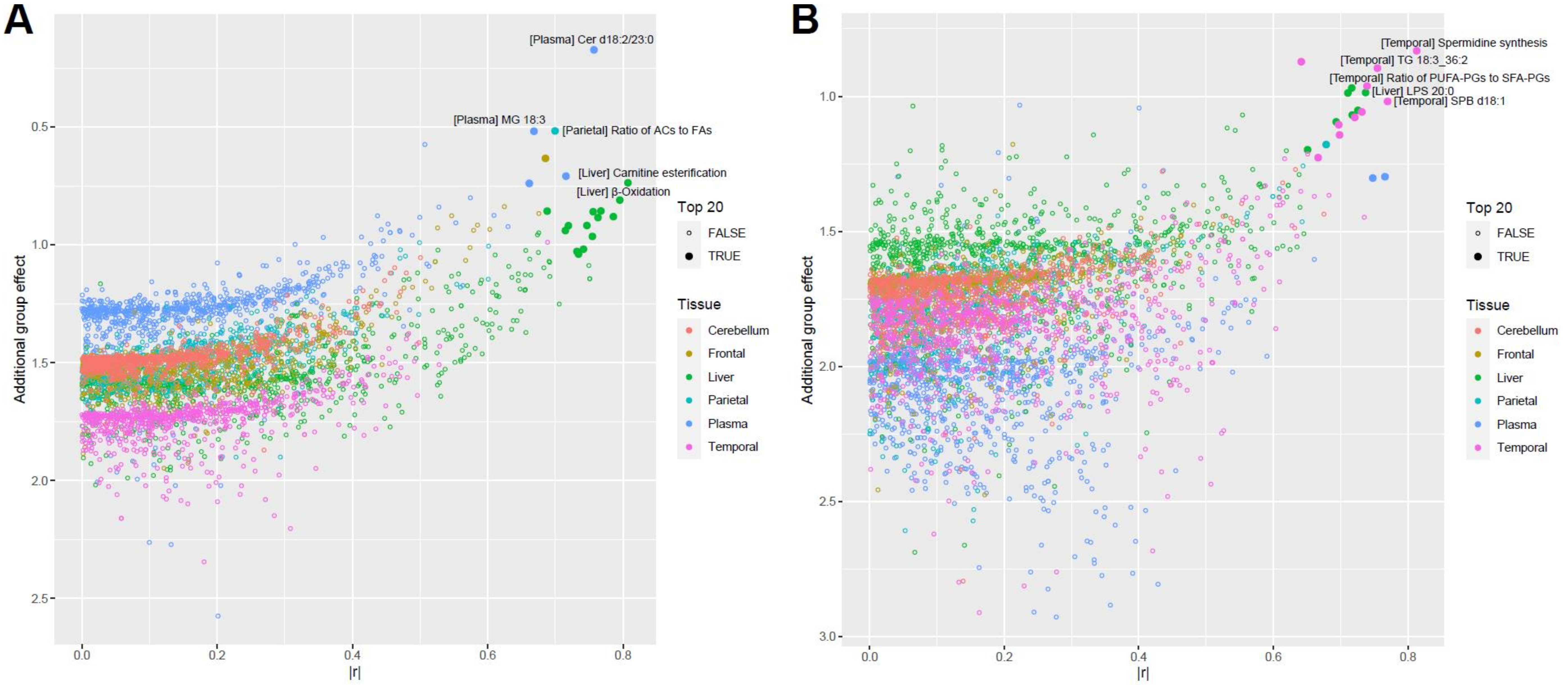

Scatter plots with distribution of components of the selection score (x-axis – magnitude of Pearson's correlation coefficient; y-axis – magnitude of normalized group effect from linear regression model; see **Methods**, section Statistical analysis – Integrative analysis) for **A)** Target hole time (Barnes maze), and **B)** LTP in CA3-CA1 hippocampal region, among APP/PS1 mice across tissues. Each point represents one analyte, with the top 5 labeled by text, top 20 showed as full points, and others depicted as circles. Individual types of tissue are color-coded (red – cerebellum; dark yellow – frontal cortex; green – liver; blue– parietal cortex; purple – plasma; pink – temporal cortex).

Supplementary Fig. S3: Experimental timeline

A

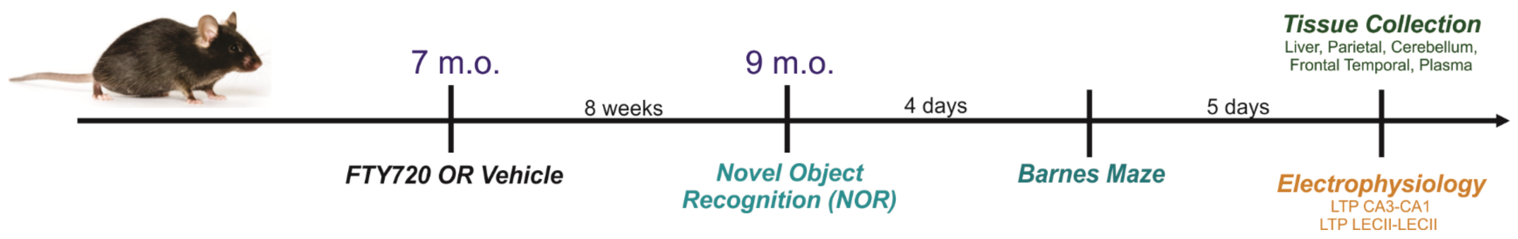

7-month-old animals (APP/PS1 or wild-type littermates) were treated with/without FTY720 for two months. At 9 months they were trained on the NOR (four days) and the Barnes (five days) behavioral tasks. After behavioral testing, electrophysiology and tissue collection for metabolomics were performed.
